# A machine learning approach for the identification of odorant binding proteins from sequence-derived properties

**DOI:** 10.1186/1471-2105-8-351

**Published:** 2007-09-19

**Authors:** Ganesan Pugalenthi, Ke Tang, PN Suganthan, G Archunan, R Sowdhamini

**Affiliations:** 1 School of Electrical and Electronic Engineering, Nanyang Technological University, 639798, Singapore; 2 Nature Inspired Computation and Applications Laboratory (NICAL), Department of Computer Science and Technology, University of Science and Technology of China, Hefei, Anhui, China; 3 Department of Animal Science, Bharathidasan University Trichirapalli, Tamilnadu, 620 024, India; 4 National Centre for Biological Sciences, UAS-GKVK campus, Bellary Road, Bangalore 560 065, India

## Abstract

**Background:**

Odorant binding proteins (OBPs) are believed to shuttle odorants from the environment to the underlying odorant receptors, for which they could potentially serve as odorant presenters. Although several sequence based search methods have been exploited for protein family prediction, less effort has been devoted to the prediction of OBPs from sequence data and this area is more challenging due to poor sequence identity between these proteins.

**Results:**

In this paper, we propose a new algorithm that uses Regularized Least Squares Classifier (RLSC) in conjunction with multiple physicochemical properties of amino acids to predict odorant-binding proteins. The algorithm was applied to the dataset derived from Pfam and GenDiS database and we obtained overall prediction accuracy of 97.7% (94.5% and 98.4% for positive and negative classes respectively).

**Conclusion:**

Our study suggests that RLSC is potentially useful for predicting the odorant binding proteins from sequence-derived properties irrespective of sequence similarity. Our method predicts 92.8% of 56 odorant binding proteins non-homologous to any protein in the swissprot database and 97.1% of the 414 independent dataset proteins, suggesting the usefulness of RLSC method for facilitating the prediction of odorant binding proteins from sequence information.

## Background

Olfaction is an important process to establish behavioural response and involves the binding of small, hydrophobic, volatile molecules to receptors of the nasal neuroepithelia [1]. The olfaction mechanism has been well studied and is generally similar in vertebrates, insects, crustaceans, and nematodes [2-4]. The first step in olfaction is the solubilization of the hydrophobic odorants in the hydrophilic nasal mucus.

Odorant Binding Proteins (OBPs) play a vital role in the olfaction. OBPs are small soluble polypeptides, which are thought to act as a carrier for odorants and carries odorant from the environment to the nasal epithelium in vertebrates and sensillar lymph in insects [5,6]. OBPs of vertebrate are members of large family lipocalin and shares eight stranded beta barrel [7]. Insects OBPs include the general odorant-binding proteins (GOBPs) and the pheromone-binding proteins (PBPs), which are completely different from their vertebrate counterpart both in sequence and three-dimensional folding [8]. Insect OBPs contains alpha helical barrel and six highly conserved cysteines [9]. Another class of putative OBPs, named chemosensory proteins (CSPs) has been reported in different orders of insects, including Lepidoptera [10-12]. These polypeptides, of about 12 kDa, do not exhibit significant homology to PBPs and GOBPs and contain four conserved cysteine residues all involved in intramolecular disulphide bridges. In spite of the sequence and structural difference, their general chemical properties indicate similar functions in olfactory transduction.

Previous reports have shown that OBPs are present in large number within a species [13]. This suggests that OBPs do play an active role in odorant recognition rather than merely serving as passive odorant shuttles [14,15]. Several reports have demonstrated selective binding of odorants to different OBPs derived from a given species [16-18]. OBPs are also suspected to participate in the deactivation of odorants and signal termination [19]. Presence of OBPs in non-sensory tissues of insect suggests their non-sensory roles [20]

Although many efforts have been made to study the role of OBPs, their physiological function is still unclear and more sequence data are required for the complete understanding of the odorant binding and transport mechanism. With the rapid increase in newly found protein sequences entering into databanks, an efficient method is needed to identify OBPs from the sequence databases. At present, prediction of the odorant binding proteins is primarily based on sequence similarity search methods [21,22] and these methods will not be employed efficiently due to the fact that OBPs show very low sequence similarity between species and within the same species [23,24]. So far, SVM and other statistical learning methods have not been explored for predicting odorant binding proteins. Here, we propose a method based on regularized least squares classifier (RLSC) method to predict odorant binding proteins from sequence-derived properties irrespective of sequence similarity.

## Results and discussion

The dataset used for the prediction was obtained from GenDiS [25] and Pfam [26] databases. Positive class consists of 476 odorant binding protein domains [see Additional file 1]. whereas the negative class has 2157 non-odorant binding protein domains [see Additional file 2]. A regularized least squares classifier (RLSC) [27,28] was used to conduct the training and testing on the dataset. First, the classification was carried out without feature selection, i.e. all the 1463 features were used. The confusion matrix achieved by RLSC is given in Table 1.

**Table 1:** Confusion matrix for RLSC on the training dataset

	**Predicted class**	
	
**Original class**	Positive	Negative
Positive	451	25
Negative	35	2122

To analyze the impact of the feature selection procedure on the classification performance, we selected eight feature subsets by decreasing the number of features. The performance of the method for discriminating between odorant binding proteins and non-odorant binding proteins is summarized in Table 2. In this Table, TP and TN stand for true positive (correctly predicted OBPs), and true negative (correctly predicted non-class-members). The results show that our method can distinguish odorant binding proteins from other protein sequences with an accuracy of >90% and Matthews Correlation Coefficient (MCC) of 0.922, when evaluated through leave one out cross validation. Using all the 1463 features, the RLSC achieved the TP rate of 94.5% and the TN rate of 98.4%. The overall Leave-one-out accuracy (LOOA), Balanced LOOA and MCC were 97.7%, 96.5% and 0.922 respectively. As seen in Table 2, feature selection generally does not deteriorate the classification performance much. The usage of smaller number of features only leads to a decrease of the TN rate. The TP rate is less influenced by the feature selection. In some cases, feature selection even leads to slight increase of the TP rates.

**Table 2:** Classification results achieved on different feature subsets. The optimal values of *σ *and *λ *are also given.

Features	*σ*	*λ*	LOOA	BLOOA	TP rates	TN rates	MCC
1463	2.614e-5	1e-009	0.977	0.965	0.945	0.984	0.922
450	4.714e-5	1e-009	0.975	0.963	0.945	0.981	0.915
250	6.325e-5	1e-008	0.970	0.961	0.948	0.975	0.901
100	1e-4	1e-008	0.970	0.962	0.950	0.975	0.903
50	1.414e-4	1e-008	0.967	0.958	0.945	0.971	0.891

LOOA – Leave-one-out accuracy (LOOA); BLOOA – Balanced LOOA MCC – Matthews Correlation Coefficient; *σ *– Kernel-parameter *λ *– Regularization parameter; TN – True negative; TP-True positive

To test the capability, our algorithm was evaluated by independent dataset obtained from NCBI database using keyword search. The keywords used for the search includes "odorant binding proteins", "pheromone binding proteins", "chemosensory proteins", "antennal protein" and "juvenile hormone binding proteins". The sequences that are present in the positive training dataset were removed from the list. After careful manual inspection, 414 odorant binding proteins were selected for independent testing [see Additional file 3]. The performance of our algorithm was compared with PSI-BLAST [29] and HMM [30]. PSI-BLAST search for each sequence was carried out against the database of positive training dataset. HMM analysis for each query sequence was performed against the HMM profile obtained from the positive training dataset. Our approach correctly predicts 402 proteins as odorant binding proteins whereas PSI-BLAST and HMM methods predict 369 and 360 proteins respectively [see Additional file 4]. The overall prediction accuracy for our approach, PSI-BLAST and HMM method is 97.1%, 89.1% and 86.9% respectively (Table 3).

**Table 3:** Prediction result of 414 odorant binding proteins by RLSC, PSI-BLAST and HMM methods

Method	Correctly predicted as odorant binding proteins	Incorrectly predicted as non odorant binding proteins	Classification accuracy
RLSC	402	12	97.1%
PSI-BLAST	369	45	89.1%
HMM	360	54	86.9%

Further analysis of 414 odorant binding proteins shows that 56 proteins have no single homologous protein in the SWISSPROT [31] database based on PSI-BLAST search result. A similarity E-value threshold of 0.01 was used for homologue search to ensure maximum exclusion of proteins that have a homologue. Our method correctly predicts 52 proteins as odorant binding proteins. This result shows the capability of our prediction systems for recognizing novel odorant binding proteins that are non-homologous to other proteins.

In this work, a total of nine physicochemical properties, secondary structural content and frequencies of di-peptides and tripeptides were used to represent each protein sequence. It has been reported that not all feature vectors contribute equally to the classification of proteins; some have been found to play a relatively more prominent role than others in specific aspects of proteins [32]. It is therefore of interest to examine which feature properties play more prominent roles in the classification of odorant-binding proteins. Our analysis suggests that molecular weight, hydrophobicity, hydration potential, average accessible surface area and refractivity play more prominent role. Hydrophobicity is an important factor for the formation of binding pocket and also for the interaction between OBP and odorant molecule. It is also observed that the tripeptides play significant role in our classification scheme than dipeptides.

## Conclusion

Overall prediction accuracy of 97.7% (94.5% and 98.4% for positive and negative classes respectively) shows that RLSC is a potentially useful tool for the prediction of odorant-binding proteins. It is also a computationally efficient method for the prediction of odorant binding proteins despite the low sequence identity. Further, the capability of our method is tested by an independent dataset consisting of 414 members and this method is able to predict 97.1% of 414 odorant binding proteins. This approach can be used to identify novel odorant binding proteins from genome sequence databases using sequence-derived properties.

## Methods

### Classification models

All results presented in this paper are acquired through a leave-one-out cross-validation (LOOCV) procedure. A regularized least squares classifier (RLSC) is used as the classification model. From the machine learning viewpoint, RLSC belongs to the large family of kernel methods and is closely related to the well-known support vector machines (SVM) [33,34]. The difference between RLSC and SVM is that they formulate the classification in different ways. However, both of them can achieve comparable classification performance [35]. Recall that our dataset is now represented as *S *= {(**x**_1_, *y*_2_),..., (**x**_*n*_, *y*_*n*_)}, where **x**_*i *_denotes the instance (i.e. the protein sequences) and *y*_*i *_is the corresponding class label. An RLSC (denoted as *f*) typically classifies a data points **x **by

f(x)=sign[∑i=1nαik(xi,x)]
 MathType@MTEF@5@5@+=feaafiart1ev1aaatCvAUfKttLearuWrP9MDH5MBPbIqV92AaeXatLxBI9gBaebbnrfifHhDYfgasaacH8akY=wiFfYdH8Gipec8Eeeu0xXdbba9frFj0=OqFfea0dXdd9vqai=hGuQ8kuc9pgc9s8qqaq=dirpe0xb9q8qiLsFr0=vr0=vr0dc8meaabaqaciaacaGaaeqabaqabeGadaaakeaacqWGMbGzcqGGOaakieqacqWF4baEcqGGPaqkcqGH9aqpcqWGZbWCcqWGPbqAcqWGNbWzcqWGUbGBdaWadaqaamaaqahabaacciGae4xSde2aaSbaaSqaaiabdMgaPbqabaGccqWGRbWAcqGGOaakcqWF4baEdaWgaaWcbaGaemyAaKgabeaakiabcYcaSiab=Hha4jabcMcaPaWcbaGaemyAaKMaeyypa0JaeGymaedabaGaemOBa4ganiabggHiLdaakiaawUfacaGLDbaaaaa@4C54@

where *k *is the so-called kernel function that models the relationship between data points **x**_*i *_and **x**, and the coefficients *α*_*i*_'s are to be computed by training. In practice, the kernel function is usually defined before training the RLSC. And the *α*_*i*_'s are computed through the training process, which involves solving a system of linear equations:

(**K **+ *λn***I**)**α **= **Y**

where **α **= [*α*_1_, *α*_2_,..., *α*_*n*_]^*T*^, **Y **= [*y*_1_, *y*_2_,..., *y*_*n*_]^*T *^and *λ *is a predefined positive constant called the regularization parameter. **I **is an identity matrix of size *n*. **K **is the kernel matrix, whose components can be computed as *K*_*ij *_= *k*(**x**_*i*_, **x**_*j*_).

In our experiment, a Gaussian kernel *k*(**x**_*i*_, **x**_*j*_) = exp(-*σ*^2 ^||**x**_*i *_- **x**_*j*_||^2^) is used for the RLSC since the Gaussian kernel is suggested as the first choice for most kernel methods. It is obvious that the values of the kernel-parameter *σ *and the regularization parameter *λ *are crucial to the RLSC's performance. Thus, both parameters are optimized to maximize the balanced leave-one-out accuracy. Due to the specific formulation of RLSC and our choice of LOOCV for fine tuning the parameters of a model, we can overcome the longer time problem by computing the training process only once.

### Datasets

All odorant binding proteins are obtained from GenDiS [25] and Pfam [26] databases. Sequences having more than 40% sequence identity are removed from the dataset. After careful manual examination, a total of 476 odorant binding proteins are considered for the construction of positive dataset which includes 40 vertebrate odorant binding proteins, 282 insect general odorant binding proteins, 46 pheromone binding proteins and 108 chemosensory proteins [see Additional file 1]. Due to the limitation in the number of known odorant binding proteins, the positive dataset could not be enhanced any further. However, in future, as more and more sequences are clarified to belong to the family, we can enrich the positive dataset. The negative samples are taken from seed proteins of Pfam protein families, which are unrelated to odorant binding proteins. Our final negative dataset consists of 2157 non-odorant binding domains [see Additional file 2].

### Derivation of physicochemical properties from protein sequence

Amino acid composition is one of the most basic characteristics of proteins and is extensively used in sequence based prediction studies [36]. Instead of using the conventional 20-D amino acid composition, another new concept called "pseudo amino acid composition" has been reported in order to include the sequence-order information which leads to a higher success rate in sequence based prediction studies [37-40]. Owing to the wide applications of PseAA (pseudo amino acid) composition, recently, a webserver called PseAA [41] was designed in a flexible manner to generate various kinds of PseAA composition for a given protein sequence [37,38] according to the needs of users. Apart from the amino acid composition, sequence-derived structural and physicochemical features have frequently been used for various prediction studies.

In this work, amino acid composition and nine physicochemical properties were employed to describe each protein. Given the sequence of a protein, its amino acid composition and the properties of every constituent amino acid are computed and then used to generate feature vector. The computed amino acid properties include molecular weight, hydrophobicity, hydrophilicity, hydration potential, refractivity, average and total accessible surface area, secondary structural content and propensity of amino acids at secondary structures [42]. Secondary structure for each sequence is predicted using PSIPRED [43]. Additionally, frequencies of dipeptides and tripeptides are used to represent protein sequences for classification [44]. To reduce the dimensionality of feature space, the amino acids are clustered into 11 groups with similar physicochemical or structural properties as shown in Table 4. All possible pairwise and triplet combinations are computed from the 11 groups and this gives rise to 66 dipeptide and 1331 triplet combinations. The dipeptide and tripeptide frequencies are computed from each sequence and are represented by one or more pairwise and triplet combinations respectively. As a feature space, 1463 feature vectors represent each protein sequence.

**Table 4:** Amino acid groupings (11 groups) according to their physical and chemical properties

**Attribute**	**Amino acids**
Hydrophobic (hb)	F, I, W, L, V, M, Y, C, A
Hydrophilic (hp)	R, K, N, D, E, P
Charged (Ch)	R, H, K, D, E
Neutral (Neu)	T, H, G, S, Q
Aliphatic (Ali)	I, L, V
Aromatic (Aro)	F, W, Y, H
Polar (Pol)	N, Q, R, E, D
Nonpolar (Npol)	F, M, I, L, V
Polar-Nonpolar (PN)	C, K, H, Y, W
Small (Sm)	P, V, A, G, T, S, N, D
Cysteine (cys)	C

### Feature selection

**Figure 1:**
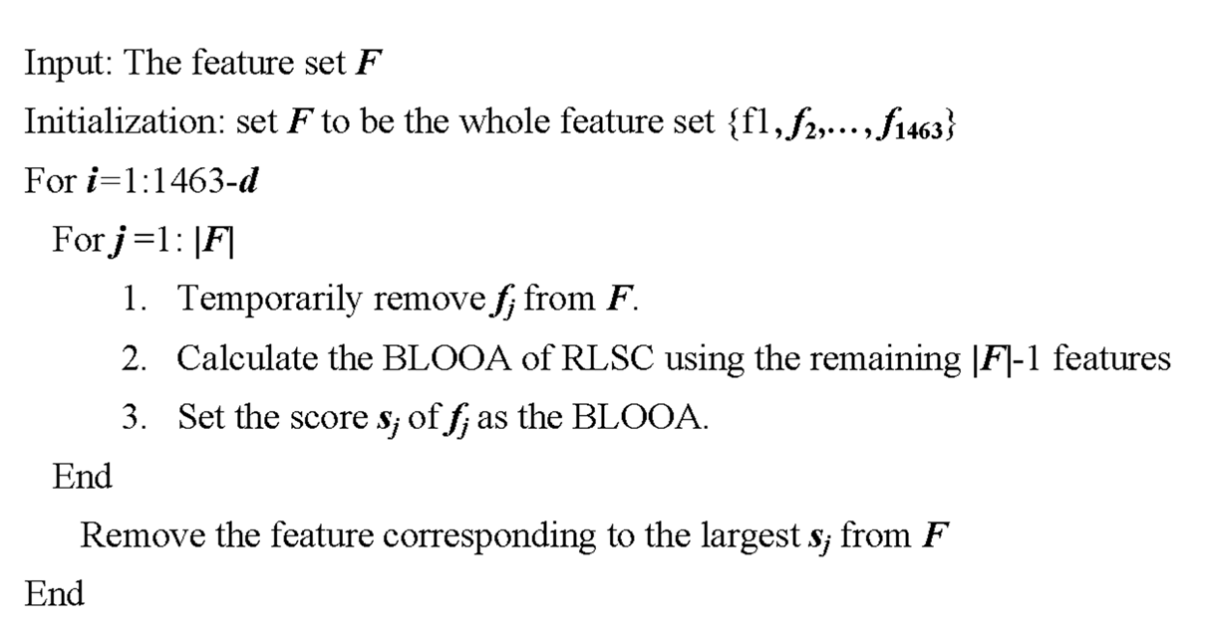
Description of the feature selection method. Redundant features are sequentially removed until the number of remaining features reaches a pre-defined number.

In this work, the main purpose of conducting feature selection is to remove possible redundant features from the original feature set. By redundancy, we mean that the feature has negligible influence on the final classification performance. We design a wrapper approach [45] to conduct feature selection for our dataset. In this method, we utilize the balanced leave-one-out accuracy (BLOOA) of RLSC as the selection criterion. The sequential backward elimination (or the recursive feature elimination) scheme is employed as the search scheme. To be specific, the feature selection procedure can be described as follows: We start from the whole feature subset (i.e. with all the 1463 features) and calculate the BLOOA. Then, features are iteratively pruned from the feature set. At each iteration, the feature whose omission leads to the largest BLOOA is pruned. Assume that we need to prune the number of features from 1463 to *d*, the feature selection (or redundant feature elimination) procedure is demonstrated in Figure 1, where |*F*| denotes the cardinality of *F*.

### Leave-one-out cross-validation

Among the independent test dataset, sub-sampling (e.g., 5 or 10-fold sub-sampling) test and jackknife test, which are often used for examining the accuracy of a statistical prediction method, the jackknife test is deemed the most rigorous and objective as analyzed by a comprehensive review [46] and has been increasingly adopted by leading investigators to test the power of various prediction methods [47-51].

In this paper, we have used Leave-one-out (i.e., jackknife) cross-validation approach to estimating generalization performance of a classifier. It involves removing one protein from the training set, training the classifier (in our case, the RLSC) on the remaining proteins and then predicting class label of the removed (left out) protein using the trained classifier. This process was repeated until all proteins had been left out. Then the leave-one-out accuracy is computed by counting the total number of correct predictions and divided it by *n *(i.e. the number of samples in the original dataset).

### Balanced LOOA for unbalanced population of classes

Although LOOA has been commonly used in the literature, it is also known that LOOA may not provide a precise evaluation on the performance of a classifier if a large unbalance in the population of different classes exists in the data of interest. To be specific, a good classifier is usually expected to provide high accuracy on both the positive and negative data. But LOOA will bias more to the True Positive rate if we have much more positive samples in the dataset and vice versa. Since our dataset contains much more negative instances than positive instances, alternative metrics needs to be used in addition to the LOOA. We resort to the balanced LOOA (BLOOA) [52], which can be computed as:

BLOOA=12(TP+TN)
 MathType@MTEF@5@5@+=feaafiart1ev1aaatCvAUfKttLearuWrP9MDH5MBPbIqV92AaeXatLxBI9gBaebbnrfifHhDYfgasaacH8akY=wiFfYdH8Gipec8Eeeu0xXdbba9frFj0=OqFfea0dXdd9vqai=hGuQ8kuc9pgc9s8qqaq=dirpe0xb9q8qiLsFr0=vr0=vr0dc8meaabaqaciaacaGaaeqabaqabeGadaaakeaacqWGcbGqcqWGmbatcqWGpbWtcqWGpbWtcqWGbbqqcqGH9aqpdaWcaaqaaiabigdaXaqaaiabikdaYaaacqGGOaakcqWGubavcqWGqbaucqGHRaWkcqWGubavcqWGobGtcqGGPaqkaaa@3C6F@

where *TP *and *TN *denote the true positive and true negative rate, respectively.

### Competing interests

The author(s) declares that there are no competing interests.

## Authors' contributions

GP and KT contributed equally for the analysis and manuscript preparation. RS and PNS coordinated the study, helped drafting the manuscript and critically revised its content. GA provided useful suggestions to improve the classification scheme. All authors read and approved the manuscript.

## Supplementary Material

Additional file 1 Positive training dataset. This data provides 476 protein sequences that are used for training. Click here for file

Additional file 2 Negative training dataset. This data provides 2157 protein sequences that are used for training. Click here for file

Additional file 3 Independent testing dataset. This data provides 414 protein sequences that are used for testing. Click here for file

Additional file 4 Prediction results of 414 odorant binding proteins. This table provides prediction results for 414 odorant binding proteins by our method, BLAST and HMM, where "+" represents proteins correctly predicted as odorant binding proteins, and "-" represents proteins incorrectly predicted as non odorant binding proteins. Click here for file
