# Supplementary material for: A machine learning approach for the identification of odorant binding proteins from sequence-derived properties
Source: BMC Bioinformatics. 2007 Sep 19;8:351. doi: 10.1186/1471-2105-8-351 (PMC2216042; doi:10.1186/1471-2105-8-351)
Supplement: Additional file 1 — Positive training dataset. This data provides 476 protein sequences that are used for training. [file 1471-2105-8-351-S1.doc]

>17738189-1

FSLVGPYNIQGKVLILPISGTGQSNMTMVNVRAIVSFSGKPLVKNGETYL

DVTDLKITMKPESSHYHFSNLFNGDKALGDNMNVFLNENSEAIYKETAKA

IDRSFGKLYLGVVKGVFSKLPYAKFFA

>17945746-1

IRVKAQYRSTGVLILVKASGAGDYWGEYEGVKAKIYFKAVANEGPDGRTY

LTTDSVKMDFNVKEIQMGVDNIANGNTVIQAALNLFINSNSQELLKEMKP

ALRTKLTLVIRNFMDRIFAKIPLDEWIN

>1841697-1

LEVRAKYDVDGKLLILPIVSKGDLTIRLNDVHTKVWITAEPVKRSDGHTY

LNITDYKTATKIKGSSPSGHFDLSNLFNDNKELRDSTLKVLNQEWSTLAL

DVQPKINEACAKAFSAIVQSLWANIPYDEFFE

>19528567-1

MFVEGLYKADIKLNDLKLNPKGAFNITMTDVAMRARPIGELYERDGHTYL

RLTKLETEPKVGDLKFYANGLVPDPVLNDVILDFINQYWRQLYQAMLPET

LDTWQPLILKSTNDFFAALPFDMLVT

>19921236-1

LKAGGHFKADVKFGGLRLVPKGPFNITIDNIKATILTDGHIEQLPSGQQR

LSLHRLNANVNIGDAKVVANGIFSDRNLNAMILNLVNENLPEITRVGIPA

TREQWAPILIAHINEFFAKVPIEKFLV

>19921294-1

VNVDTQYDLSVLLKKYGFTINLIGAGHAKFAIKDMVIWGTMKYSLGVISG

NLKLKSLEVRTHLGEVDSEIEGILGDGSINEKMNEYLAEAVELAINENED

LIADTIESIALPAVNSVLDDISIAEIIS

>20129495-1

LNIVGSYKADMQVNQLQLKPKGEFNVTLLDVEAITVTDGEVYEKDGHRFF

RLKNIDSKPKIKDLVIKANGIFADPELDKIALNVANQYWRDIYGIMLPET

RQFWQPLMLRMFNEAFELVPIDQFLK

>21355505-1

IEMIGDYEMSGRILLLPITGHGKANVTLINTKIEHRLIGEPFEKDGVKYM

RLKDYRVSFDPKRVYMNFENLFNDKTLSDGMNRFLNENWETVFNELKVGY

AKSFGIIFRELSNKLFEKVPFDNIFL

>21355625-1

LKIEAKYTSSGVLLILPASGGGDFHANFEGVSADLTGKTSIHAFKGANYL

HIDALSLVLDVKDVKMSISGAFNNNRILLEATNLFLRENSQVVLEAMQAQ

LQKKLASEFGKLANQLLKNVPVEQFYV

>24644107-1

LHGDGLYEINGNILALPIKGNGPFTGNFTNFVAYVRVQYDIKSVNDLEYL

HVKEFVLKIRTGKGNLKLENLFNGDKVLGDVINDTINQNFEVFTNDLIAP

IARALEAKFLVITTKILENFTYSELFP

>24647332-1

LRIRAKYNLKGNILLLPLVGSGDVAMALKNVHTTVYTRISLRNETRTGDE

IIHIDEMKVGFDVGAMRIHLKNLFNGNEILAASINSFLNQNGKEVIAELR

PDLELGLADIFHGLWNNVFSKMPTKLWLV

>24647334-1

LHMESDYSIKGKIMMMPLLGDGHCKVDLVNITMRTELIGQEYKKNGANFL

KINTVKVKYELSDVHIHLDNLFNGDKALGDRMNEFLNENWKALAEEVRPL

MTKALVDILRASVDKLFASFSYDDLLP

>24648601-1

LIHKGDYFSMGRVWIVQMNSTGESLSDFQNFRFVLKLKVIMEYRNNKRYL

KIYELTPFVTMDRWVFWLDNFFESNTDMTIAINQVFNLHWVEFWNELEPT

NLKIFAGVFRSVFEDIFKKVPYDDMFL

>24648603-1

LVYKGDYVAKGRMLWFVDIHSQGTSESDFLNFQFVLTLKVRVEYRNNKRY

LKIYELVPNIRLDRWIMWLDNFFPDNEDLTIAVNNLFNRNWVEFWNELEP

GILRLFETVFLSLFEDLFEKVPYDDLFL

>24648605-1

LKLNGTYEMRGSLLTMPIHGKGQAKVEIRECRVRCKVRVLEDLRDDGKLY

AGISKVKCLLDVQGMHLNLENLFNNPEMSDAMNVVANTKWLEIWHNLRRG

ITSAVDQLVESILQRLLGLKGEESGDL

>24648778-1

SLEGEYEFAAKMLGTEMNRKGHWNLTLYDYSQTTSVRRIGGPGSLIKVHV

EVDRIGGMELHIENLLQGQPLNQLADGVINSMWQLGLPFIKPMINELVST

AFTDIFNESFRHFPLEKFLA

>24649971-1

IVLKGKYVADGRILILPIRGDGDAEIVLHNPKFSVKFKPGTQQRNGRTYL

SVDKLKVLVEPQKMNIRLENLFNGDQALGTNLNQFLNDNWTEVWNELHPS

IHVAIAEIMKSVLSQLFKRFAYEDLFL

>24649974-1

IGVRGPYSVDGRILILPITGNGIADIRLTRTKVRAQIKLKRVSKGDHQTY

AEVMNIKVELDPSHVTYQLENLFNGQKDLSENMHALINENWKDIFNELKP

GIGEAFGLIAKSVVDRIFGKLPLEQLFV

>24650515-1

LRFNFDYKVKGHVSALNLNGHGKGYFEAENALLLLELAVKPLATSDGYFA

DVQSVKVNFREIKQFRIKLENLFGGNKDLEDTAHILFNENWRDFFEVLRP

AVEQTVGGVLLDRFKKTFVYVPATYLI

>24650517-1

MRLDGRYEMAGRILLIPLSGSGKIFIEIDDLDILLLTKIRLYEKGGFTFD

NVTAVQVQLNLSKVRTYLDNLFNGRSKEVERSTNEFFNENWRDFYEALKP

LIVETVENILYDVMSTVFHLIPANFFV

>24650521-1

MRIDGHYKMVGRILLVPLQGNGKIVMEIDDLDILMTTKTRLYEKGGYTFY

NVTSVKVKVDVGKVRTRLDNLFNGHSKEVEDSTNQFFNDNWKDVFEALRP

LVVETVERTLLDLLHKTFALFPASFFV

>24668608-1

MKLQADYSLFGRILLIPLNGKGQVFLDAENMTVTMHTKTRLYSKGGFTFY

NVTNLHVDFKMDGLKSYFSNLFNGNKQLEDSTNKFFNDNWRMLADALYTV

ITQTIEDILLDVLKKIFHFIPANFFV

>25012183-1

LNFTTDYELDMGSGYRIKRNGGAFFALEDLNIQGRISYSLGVFTSQLRVK

DVLIYPSVGNVNSQIENLSKYRIFNRKLNEIIEEFVTLTINENTDFVAAW

VSEQATPICNDLIGDRTLSDIIA

>28396156-1

MRLEGNYHMQGRILVIPLNGHGKCWFEPSGMDIIMRTSTDLYQKNGHVFY

NVTGTKVDYTISGLRLHMGNLFEGVKVLEDSTNQYLNDNWRPVSEALKPI

IAKTIEDILLAIMQNIFHQLPADYFV

>28396158-1

ASLVGSYKIKGKVLILPIQGEGTSNMTMVNCDFLMKWNGALEKRANGKEY

YQMNKIKATFDTTRFYMHLTNLFNGDKALGDNMNQFLNDNWEDILKELKP

AIIGAFTKIFRAIITNVFENVPYEELFL

>28572146-1

LVHEATYDMSGRVLLFFFNTTGRLISDFQNFRITLTIKALVEYRNDKRYL

KIYNLVPSLDLDRWIIWLDGLYKENTDVTIFMNKLFNENWVEFWNDLQPG

LVKAFTNAFTVLLNRVFDNVAYDDMFL

>29165392-1

LVLQSNYDLSGSIRNIPIEGRGRSNIVHEKYRIGMRGGFDVVLDLKGRPH

VRINHYDIDRLEVVGNIRSDFSNLYNGDVARSAVMHKYLNDNWKSIGFYT

QYPTMRSFMKRMMENLIKFLQVVPANELFF

>31241293-1

IKATAQYQSSGVLLLLQASGAGEYWGEYEGVKAKTYFKATPYQGDDGMTY

LTVDQTKMDFSVKEIKMGVENIANQNAIIHAAMNLFINTNAQELLKEMKP

QLRTKLTEHLQSFLQQLFDRIPVEQWLD

>31242725-1

LRTKGKYKTKLKLIGVLPVNRFGSFRFDLKGLTVKGGASVAISGDKLQVR

DLVITPTLKSVRSDVRGIFLNPVASFAFNRMVELAVPGLINNNQQAITQA

IEQQLKPAINEMLGDISLQDLID

>48097306-1

INVMTDYNVTGTLVDSFSIFGNGNIRVAIKGLNVTVDLKLGVKNNKLNIS

KLTFNVHLKEFDCAITGLYNDEEVSKLLSKAITEVLPGLLDDYQTEISNY

ASPIIANTLNNKFLNNLSLKDLID

>48109142-1

LLIDGDYKAEGSLATFKMGGKGFFNISMENIRTVWDISGHVVNDRWVVEH

FKTAPTISSMKVWFSDLFNGNEELNRAALVFINEYWPLVYRTMLPKLIEM

WDTYLSEFSNRFFSKIPFSTIFP

>48109165-1

LILDAHYSSSGVLIILPASGNGTFHARFDDVTAVVKGTVSTRVKDGKTYL

NVDNLDVVLDVKKVRMGVHKIFNNNRILTEATNLFLRENGQEVLKVMEPQ

LKRKLSVLFAGIVNQLLRHVPVEVFLL

>505621-1

LQMKGHYKLFSNSSLIRNLLDGDTLHGDGNGGLKFEKMKMSFDFRFKVEQ

RGGDNFLNIDSESSKFNVDILRGASFAADNLYLGDVEASATIMKVMNQHW

KLVMDGTALPFMYKIKGLFYDLTQKYFNNVPVKIISM

>52630376-1

VDFVADYKIEGKVLLLPVRGAGKSNITMYDLKSHNDIYCEKYEKNGETYL

RIKKHAVKFNPAKVKLRFENLFDGNKELGEQMNRFINENSELLFKELQAA

YEETFSLVFTKIDNEIFNRVPFDKIFP

>54639741-1

LAFKGPYSVDGKVLILPIVGKGRAEIVLKHCKIHSLITLRPISKGGHHTF

AEVTDIKLQVDPSHVSYKLEGLFHGQKDLSENMHILINENWQEIFNELKP

SISEAMGLIVKSVLNKTFGKTPLEELFI

>54639743-1

YSLVGEYSIKGQVLVLPISGSGHSNMTLIGTDLKVSFIGKPVEKNGEIYM

EATDLQYYLKPKSQHYHFSNLFNGDKILGDTMNTFLNENGAAIFSETKET

IEKAFAALYQPLITRVFSKYPYAKYFA

>55237501-1

IVMEGYFKGEGRFNSIKLASKGYFNNTMTDVTTTWKMSGHVKERDGEQYL

EIEDFDMSPEVGNMKIYATGLFPDPELNQIALEFVNQYWPMFYKEMLPGT

RQVWEPVMIELVNKIFLRVPYRRLLP

>55244195-1

MSILVLQINGDGDLQVNLTDTKVSLKLTFYTETVDGEEYFRFNPINLRVK

FGKARFYLKNLFNGDPTLEMIGNQAINENPDVLLDEVKGGIEENLAKLFT

KIASEVVKDALFEEVFP

>55244820-1

VQFAGNYTMDGRILVLPITGKGLANITLHRLKTHHELIGELVERNGEQYM

HIRKYLVHFEPKLVTFQFGNLFNGDERLGKTMHQVLNDNWEVVFRELRSS

YEDTFGYIFKKISNQIFLKVPMNKIFP

>57906452-1

ISANGFYDANGRLWGLIPIFGIGDFSVRPRDVVATGFATITDNGQGFLKL

SDFSISLQIGSLESNIQGLLLGGDLSDLLNAVIQDIVPSVLRNFPDGMTN

LLNALVVPIANRFLATRTMEDLMG

>58395197-1

SLDGGYDFQSKILGLPRHTRGVWNLTLYNYSQTTTVTRIGGPGGLLKVRV

EIDKIGDMKLHISDLFHGAKIIESIADFFINTMWQPSFPFIKPLINDLVS

VAFTDIFNESFRYFPLDQVIR

>58395199-1

LTIHAKYTSSGVLIIIPASGSGDFDAVFDGVTADVKGLVSTNEKPTGMHL

RVEKLDLNLSIKKPRLSVSKIFNNNRILTEATNLFLKENGHEVLRALQPQ

LQKKLSSEFTGIANQLLDNVPIHFFIV

>58395201-1

LYVEGRYVVDGRILLLPVKGSGKFTGNFTQGIGSVRIKGDRKRINGKDHL

SLAKLDIKIRVSDGRVKLENLFGGDRVLGEIINETINQNFNLLSTELIPL

IEKALQRIFKRTGNKILERFPEEVLFP

>58395203-1

LRIDATYNLKGNVLLLPLVGDGDVTMSLKNVKTTVVTKFSVRPLPEDAIF

IEEMKVTFLVGGMRIHLDNLFQGNQVLGASLNLFLNQNANEVIAELRSDL

EHGLADIFTGLWNELFNKLPLKLWIA

>58395205-1

VETRGKYEVIGNVLLLPVRSNGEFWTEFSDITAIAKIYGKAVERDGESFM

GIEKINVDFTMKNARFKVKDHVNTQNVLGEAINQFLNQNANELIQEMRPA

ASQSIGKLFRKFLNDAFTNLPTRLWLL

>61675576-1

AHFQGKYEVNGKIMSLPITGKGDFEATFDDLYAKYVTTYELQKMDDEEVY

LVPTIYNVIFETKSANVRMDNLFNGNKLLGDNMNKFLNENWQDVMKEAGQ

STYNALGLVIHNIFSGTTLTVAYKDVFD

>6560645-1

CTVKGHYTAAGRILVLPITGDGDMKLKLKNLNIKLYISYEVEKGADGKDH

IAPKKLDYDFEVVDNAQFSLTNLFNGNKELSDTMLKFLNENWKQISLEFG

KPLMDVAAKSIFKNVITFFAKEAIEDIAE

>66530058-1

IRAAAKYRSSGTLLLVKASGAGDYWGEYEGVKAKVFIRAKPFLVQDRRYL

RLQQLKMDFSVQNIKMGVENVRDSNAIILAALNLFINTNSQELLKEMKPD

LRRKLVQVMTTFVERIFAQVPYDAWIL

>66530060-1

LHFQGKYQIDARVLLLKLTGEGNITGTFTDYDSDVVLRARKVYRDNDIYL

NFERMKVKIRIGKAHLHLSNLFGGDTVLAAATHDLLNNNNALFLDEITPV

LETSLADLFTDVANKITKSFTYKELFP

>109109935-1

EGRMYVKAMVVDKDFPEDRKPRKVSPVKVTALGGGDLEATFTFMREDRCV

QKKILMWKTEEPGKFSAYGGRKLIYVQELPGRDHFVFYCKDQHRGGLFHM

GKLMGRNPDINMEALEEFKKFVQHKGLSEEDIFIPLQTG

>109110642-1

SGEWYSVLLASDCREKIEEDGSMRVFVEHIDYLGDSSLTFKLHEMTHYIP

PQHFCLGTQWVPFIFPSVWSPVPSFASMTNTQDMAVTVTLPTARTPDVSS

QLKERFVKYCEEHGIVKENIFDLTKVA

>109474951-1

NGDWFSIVVASNKREKIEENGSMRVFMQHIDVLENFLGFKFRIKVNGECR

ELYMVAYRTPKDGEYFVEFDGGNTVNILKTDYYKYLILQIINYKNGRTYL

VTKLWGRTKYLSSDIKEKFEKLCVAHGITRDNIIDLTNAD

>109514805-1

SGKWYTKATVSDRNLTVGNNPKRTLPMMVSNMDGGDLYVKIKIWVTGYCY

NIEVHLRKTNEPLKYTALKGTSVIYIVKIPVKDHCIFFCEGKRHRKRYCK

AKLVGRDSKDNPEAMEEFKKFVKSKGFRLEYIIVPERRD

>11277082-1

PGEWRIIYAAADNKDKIVEGGPLRNYYRRIECINDCESLSITFYLKDQGT

CLLLTEVAKRQEGYVYVLEFYGTNTLEVIHVSENMLVTYAENYDGERITK

MTEGLAKGTSFTPEELEKYQQLNSERGVPNENIENLIKTD

>1168469-1

QGKWYTIVIAADNLEKIEEGGPLRFYFRHIDCYKNCSEMEITFYVITNNQ

CSKTTVIGYLKGNGTYQTQFEGNNIFQPLYITSDKIFFTNKNMDRAGQET

NMIVVAGKGNALTPEENEILVQFAHEKKIPVENILNILATD

>12084609-1

SGKWITSYIGSSDLEKIGENAPFQVFMRSIEFDDKESKVYLNFFSKENGI

CEEFSLIGTKQEGNTYDVNYAGNNKFVVSYASETALIISNINVDEEGDKT

IMTGLLGKGTDIEDQDLEKFKEVTRENGIPEENIVNIIERD

>127526-1

NGEWHTIILASDKREKIEDNGNFRLFLEQIHVLENSLVLKFHTVRDEECS

ELSMVADKTEKAGEYSVTYDGFNTFTIPKTDYDNFLMAHLINEKDGETFQ

LMGLYGREPDLSSDIKERFAQLCEKHGILRENIIDLSNAN

>127532-1

SGYWFSIAEASYEREKIEEHGSMRAFVENITVLENSLVFKFHLIVNEECT

EMTAIGEQTEKAGIYYMNYDGFNTFSILKTDYDNYIMIHLINKKDGKTFQ

LMELYGREPDLSLDIKEKFAKLCEEHGIIRENIIDLTNVN

>12856407-1

NGDWRTLYIAADKVEKVKMNGDLRAYFEHMECNDDCGTLKVKFHVQMNGK

CQTHTVVGEKQEDGRYTTDYSGRNYFEVVMKEDGALFFHNVNVDESGQET

NVILVAGKGETLSKAQKQELGKLVKEYNIPKENIQHLAPTD

>12860854-1

SGTWYLDSIASDNMTRIEENGDLRLFIRNIKLLNNGSLQFDFHFMLQGEC

VAVTMVCEKTKNNGEFSVAYEGKNKVLLLETDYSMYIIFYMQNIKNGTKT

QVLALYGRSILLDKTHQREFENICNLYGLDSQNIIDMTKKD

>129022-1

SGPWRTVYIGSTNPEKIQENGPFRTYFRELVFDDEKGTVDFYFSVKRDGK

WKNVHVKATKQDDGTYVADYEGQNVFKIVSLSRTHLVAHNINVDKHGQTT

ELTELFVKLNVEDEDLEKFWKLTEDKGIDKKNVVNFLENE

>129658-1

EGNWRTVYLAASSVEKINEGSPLRTYFRRIECGKRCNRINLYFYIKKGAK

CQQFKIVGRRSQDVYYAKYEGSTAFMLKTVNEKILLFDYFNRNRRNDVTR

VAGVLAKGRQLTKDEMTEYMNFVEEMGIEDENVQRVMDTD

>13124669-1

DGKWHSLYIAADNKSKVSEGGPLRVYVKHLECSDECQTFTIKFYTKVENV

CQEHRVVGRKGKDGKYITDFSGQNYFHVVEKADDTMTFHNVNVDDSGKTN

VILVVGKGESSSIEQKQRFEKTAEKYDIPKENIEHLVTTD

>13185353-1

LGVTLGLAAALSFTLEEEDEGESVHPEENPDAEDGGAWQIQRLWGQEAHI

PAGAARDGRLRLLLQRPAPWGPALHGKACGICSLQGRAAVPTLAHLATSP

AGRNPNTNLEALEEFKKLVQRKGLSEEDIFMPLQTG

>13185357-1

TGTWYVKAMVVDKDFPEDRRPRKVSPVKVTALGGGKLEATFTFMREDRCI

QKKILMRKTEEPGKYSACLSAVEMDQITPALWEALAIDTLRKLRIGTRRP

RIRWGQEAHVPAGAAQEGPLHLLLQRP

>1835145-1

QGQWKTTAIMADNIDKIETSGPLELFVREITCDEGCQKMKVTFYVKQNGQ

CSLTTVTGYKQEDGKTFKNQYEGENNYKLLKATSENLVFYDENVDRASRK

TKLLYILGKGEALTHEQKERLTELATQKGIPAGNLRELAHED

>207551-1

SGKWYTKATVCDRNHTDGKRPMKVFPMTVTALEGGDLEVRITFRGKGHCH

LRRITMHKTDEPGKYTTFKGKKTFYTKEIPVKDHYIFYIKGQRHGKSYLK

GKLVGRDSKDNPEAMEEFKKFVKSKGFREENITVPELLD

>21465464-1

AGEWYSILLASDAKENIEENGSMRVFVEHIRVLDNSSLAFKFQRKVNGEC

TDFYAVCDKVGDGVYTVAYYGENKFRLLEVNYSDYVILHLVDVNGDKTFQ

LMEFYGRKPDVEPKLKDKFVEICQQYGIIKENIIDLTKID

>23305893-1

SGTWYTIYEASANIEVLSENSPLRGYFRLIKCHPDGETLLVIFYTKENGT

CQLYNKQGQRIDENGYTTNYEGKVDFSFIQQAKDFLLIHAINKNEEGNVF

EVVGALAREKDISEENYQAFLEFAVENGIPKENIVKVIDTD

>2598976-1

SGRWHSVALASNKSDLIKPWGHFRVFIHSMSAKDGNLHGDILIPQDGQCE

KVSLTAFKTATSNKFDLEYWGHNDLYLAEVDPKSYLILYMINQYNDDTSL

VAHLMVRDLSRQQDFLPAFESVCEDIGLHKDQIVVLSDDD

>3123036-1

SGVWFIKATVSQRREVEGETLVAFPIKFTCPEEGTLELRHTLASKGECIN

VGIRLQRTEEPGQYSAFWGHTLFYIYDLPVKDHYIIYCESHPFQKISQFG

YLIGKYPEENQDTLEVFKEFIQHKGFLQEKIGVPEQRD

>45775300-1

SGEWYSILLASDVKEKIEENGSMRVFVEHIKALDNSSLSFVFHTKENGKC

TEIFLVADKTKDGVYTVVYDGYNVFSIVETVYDEYILLHLLNFDKTRPFQ

LVEFYAREPDVSQKLKEKFVKYCQEHGIVNILDLTEVD

>14091480-1

YTDKYDNINLDEILENKRLLLAYVNCVMERGKCSPEGKELKEHLQDAIET

GCSKCTEAQEKGAYKVIEHLIKNELDIWRELAAKYDPKGDWRKKY

>21898556-1

YTDKYDNVDLDEILSNRRLLVPYVKCILDQGKCAPDAKELKEHIIEALEN

ECGKCTEAQKKGTRRVIGHLINNEADYWNELTAKFDPEKKYVQKY

>21898574-1

YTDKWDNINVDEILESQRLLKAYVDCLLDRGRCTPDGKALKETLPDALEN

ECSKCTEKQKAGSDKVIRYLVNKRQDLWKELSAKYDPNNIYQDRY

>21898673-1

YTTKYDNIDLDEILGSKRLLNNYFNCLLDKGPCTPDGKELRDHIPDALET

GCDKCSDKQKNGTRRVLKFLIDNEPDRYKELENKFDPEGTYRKKY

>24415096-1

YTSKFDNINVDEILHSDRLLNNYFKCLMDEGRCTAEGNELKRVLPDALAT

DCKKCTDKQREVIKKVIKFLVENKPELWDSLANKYDPDKKYRVKF

>2613100-1

YTTKYDNIDLDEILASDRLLANYHKCLIEEGKCTPDGEELKSHVSDALQN

DCAKCSDKQRAGAEKVINFLYNKKKPMWESLQKKYDPENTYVTKY

>27543474-1

YTTKYDNVNLDEILANDRLFDKYAQCLLEDGESNCTADGKELKKAVPDAL

SNECAKCNEKQKEGTKKVLKHLINHKPDVWQKLKAKYDPDGTYSKKY

>27543486-1

YTTKYDNIDLDEILNNERLLKKYYECLMSDSDASCTPDGKELKVSIPDAL

VTDCSKCNEKQKEGSNKVIRFLIQKKEDLWKPLQAKYDPEGTYLKKH

>31442896-1

KYDYIDPMEIVNNDRLRDQYYNCFMNTGPCVTPDAIYFKEHFPEAVVTKC

KKCTEIQKTNFEKLAIWYNENRPDEWTALIKKF

>36020870-1

YTTKYDNVNLEEILSNDRLRNKYVECLTSTSDEHCTPEGKELKSVVSDAL

TTDCAKCNEKQKNGTKYVVDTLLDKYPDDYAKLEKVYDADGAYRKKY

>38048069-1

YTSKFDNVNVDEVLNNNRVLNNYLKCLMEKGPCTPEGRELKRLLPDALQS

DCSKCTDVQRKNSEKVINFLRVQKPGEWKLLLDKYDPKGIYRAKH

>38048453-1

YTTKYDNIDVDEILKSDRLFGNYFKCLVDTGKCTPEGRELKKSLPDALKT

ECSKCSERQRQNTDKVIRYIIDNKPEEWKQLQAKFDPEEIYIKRY

>40287944-1

YTAENDDLDIDGIVKDPKKLQEWFGCFVDKSPCDNVQLSFKADMPEAIRE

ACAKCTTAQKGILKKFLVGLEEKAPADYEVFKKKYDSENKYIEPL

>40287946-1

YNNRYDNLNADSIVQNERVLLAYYKCVMDKGPCTKDGKNFKRVLPETLST

ACARCSPKQKGLVRTLLLGIRVKSEPRFNELLDKYDPDRSNRDDL

>454078-1

YDDKFDNVDLDEILNQERLLINYIKCLEGTGPCTPDAKMLKEILPDAIQT

DCTKCTEKQRYGAEKVTRHLIDNRPTDWERLEKIYDPEGTYRIKY

>47933944-1

DKLDSFNVDEVLNNERLLKSYIQCMLDADEGRCTNEGKEIKKRLPKFVAN

GCLDCTPSQLERAIKTLRHVTEKYPEEWTKLKAKFDPTGEYAKKH

>4836777-1

TKYDNVNLEEVFGNERLLESYRKCLMDEGLCAPDAEELKKAIPDALENEC

AKCSEKQKAGVETTIVFLIKNKPEIWESFKKKYDPTHKYEKIY

>4836779-1

TKYDNVDVPSLLQNERSANSYYNCLMSLGLCTPEGQFFKELLPDALATGC

SKCSDRQKAIVKAIVEFLKKNKPDDLQKLVNKFDPDGSYRAKY

>49532922-1

YTDRYDNLDLDEILNNSRLRVPYVKCLLGKGKCSPDGKELKSHVREALEN

QCGKCTPAQQAGTRKVIGYLINNEAGYWQELVALYDPQRKYVKQY

>51105598-1

YTTKYDGINLDEILKSDRLFNNYYKCLMDQGRCTPDGKELKARLPDALKT

ECSKCSEKQKEGTEKVLRYLIEKRPKQWTTLQQKYDPDNIYTRKY

>55237617-1

YTDKFDNIDVDRVLSNDRILNNYLKCLLDKGPCTQEGRELKKTLPDALKT

NCEKCSEKQRTSSRKVIAHLEERKPQEWKKLLDKYDPEGIYKSKF

>55978944-1

YTTKFDNINVQEILHNDRLLNNYVKCLLDQGRCTADAIELKKSLPDALET

ECSKCSPKQKEFAEEAMKFLSHNKKDIWEKLLAKYDPEKKYRSKF

>56462366-1

YTNKYDNTNLDEILGNDRLFNAHMECIMGEGKCTPEGRELKEHIGESLEN

ECEKCTDDQKKGAKKAIDYIIKHRPEAWKRLTDKFDPSGKYKQQY

>56713962-1

YNSKYDNFDVETLISNDRLLKSYVNCFLDKGRCTPEGTDFKKTLPDAVET

TCAKCTDKQKTNIKKVIKAIQTRHPRQWDELVKKNDPTGKHIVNF

>56805549-1

YSDMFDHINPDDILPNDELRNQYYNCFMDTGPCVTEDQKYFKEHAAEAFA

TKCRKCTEVQKKNVEKIVVWYTENRPQEWQAMVQK

>63020522-1

YTDKWDNIDLDEILNNKKILASYVKCCLDQGKCTPDAKELKSHIKEALEN

RCGKCTPAQKDGTRKVLTHLINHEPEMWNQLCEKYDAEGKYRKMY

>6560677-1

YTSRYDSMNVDDVIGNHRLLHAYIKCMLDEGRCTAEGRELKKHITDALQT

GCSRCTDAQKKAIRHVIKHLIEHEHDFWALLVEKYDPHRIYTTKY

>66560284-1

YSDKYDYVNIDEILANDRLRNQYYDCFIDAGSCLTPDSVFFKSHITEAFQ

TQCKKCTEIQKQNLDKLAEWFTTNEPEKWNHFLKSIGPKLVPFFKS

>66840187-1

YTTKYDNIDIDQILASKRLVNNYVQCLLDKKPCTPEGAELRKILPDALKT

QCSKCNPGQKNAALKVVDRLQKDYDAEWKQLLDKWDPKREHFQKF

>66840974-1

YTTKFDNFDVDKVLNNDRILTSYIKCLLDQGNCTNEGRELKRVLPDALKT

DCSKCTTVQKDRSEKVIKFLIKNRSTDFDHLTAKYDPSGEYKKKI

>77415556-1

YTTKYDNVDIDVVLNTERLLNAYVNCLLDQGPCTPDAAELKRNLPDALEN

ECSPCSEKQKKIADKVVQFLIDNKPEIWVLLEAKYDPTGAYKQHY

>77415560-1

YTTKYDDMDIDRILQNGRILTNYIKCMLDEGPCTNEGRELKKILPDALST

GCNKCNEKQKHTANKVVNYLKTKRPKDWERLSAKYDSTGEYKKRY

>77415562-1

YTDKYDTVDLDQLISNRRLLIPYVHCILEKGQCTAEGKELKSHIKEALET

NCAKCTKAQKGGTEKMIGHLINHEAEFWEELKAKYDPTNEFTKKY

>77415568-1

YTDKYDNIDVDEILENRKLLVPYIKCVLDEGRCTPDGKELKAHIKDGMQT

ACAKCTDKQKVSARKIVKHIKQHEADYWEQMKAKYDPKDEFKEIY

>77415570-1

DKYEPIDDSFDASEVLSNERLLKSYTKCLLNQGPCTAELKKIKDKIPEAL

ETHCAKCTDKQKQMAKQLAQGIKKTHPELWDEFITFYDPQGKYQTSF

>77415572-1

YDKKYDNFNVDEIIDNPRLLKAYTFCFNDKGKCTAEGNDFKKWIPESLQT

SCGKCSEKQKYLVAKFVHAIKDKMPDEFDILRKLHDPKGEYTENL

>77415574-1

YSSQYDNFDVEQLVGNLRLLKNYAKCFLDQGPCTAEGTEFKKRIPEALRT

KCAKCNPKQRHLIRTVVKAFQTKLPDLWEELAIKEDPKGQYKHEF

>77415580-1

SKYENFDVEPIVTSDRLLKAYINCFLDKGRCTPEASDFKKALPDTIATNC

GKCTEKQKANVRKVIKVIQQKHSTEWEKLVKKHDPSGKHRADF

>77415582-1

YDERYDYLDVDDIFRNKRLVRNYVDCLINAQRCTPEGKALKRILPEALRT

KCIRCTERQKRTSVKVIRRLKNEYPEEWAKLASRWDPTGDFTRY

>77415586-1

NIDVDAVLADPQRVDAAVKCFLSDADDDCNVRSKVIKSLIAEMLKTNCAE

CSEKQKAGVVKFMAHIAKNKPEEMKQLLAKYDPNGEALAKY

>77415598-1

DRLDNINVDEVLGNRRLLKTFVQCILDEGEGRCTKEGKDLKQELPRLVET

GCSDCSPRQLENGVKVLKHITENYPQEWAQMKAKYDPTGEYAKKY

>77415600-1

YPDTFDKLDLQELQGDKERVQAAIKCLVQEEDTECKPAAKLLKSVLAEIV

QTDCGKCTEAQKTKVAGFFAFVSQNYPQQMQQVLEKYDPSKEYREKY

>77415602-1

KYDHVDVERMLRNQRFVNAAIKCLLEEGPCTPEIRDLKKMLPDALKSDCS

KCSAKQKENVRKVVDFMMKQRAADWARLSRKYDPEGLHQKRI

>77415604-1

YTTKFDSVNLDDILSNDRLLNKYAQCLLDADDRNCTPDAKELKRAIPDAL

TNECAKCSEKQKAGTEKVAKFLLEKKPDLWKQLEAKYDPNGEYRKRY

>77415610-1

YPATFDSLDLQALLADEARVQAAVRCLLEDGDGACRPAGKALKEVLPEIV

RTDCAKCTETQHKKIGGFFGEISQRHPDLMKKLLDKYDPTGEFRKKY

>77415612-1

LGNDRLLHSYLECVMSDNDSKCTKEGKEVRSRIPGLVQTGCSDCTPKQLE

RAIKTLKHITEKHPEDWKKLKAKYDPTGEYTQKY

>77415614-1

YTTRYDNIDIESILKSERLLRNYFDCLMDRGTCTQEGCLLRAAIPDALQN

DCSKCSDVQKKQAGRVMAWILENKRNYWDELIAKYDPEGNFRKKY

>77415620-1

MASERLLNSYFRCLIEDTEEHCTADAKYLKEVVPDALTNGCARCRPNQRE

GAEKVIKFLMKNKPDMWSKLEAKYDPDGTYRKKY

>77415622-1

YTTRYDNIDLDEILHSTRLLNSYVNCLMERGPCTADGKELKDNLVDALQT

DCSKCSEKQKEGAQKVVDFLIDNRPAQWKELEAKYEPTGIYRRKY

>77415626-1

YPTRYDYIDIEAVMNNERIIKILFNCVMSRGPCTREGLELKRIVPDAIQT

ECAKCNERQRKQAGKVLAHLLQYKPEYWKMLVQKFDPNNVYLRKY

>1042146-1

AFVILAACNIRAELTKEEAITIATECKEEAGASDADFEAMVKHQPAESKE

GKCMRACTLKKFGVMSDDGKMIKDAAIELGKSLVKDDEKKDLVVEVIETC

DGLEVNDDPCEAAEEYGHCVKSEFE

>10798684-1

AAGIRLSMAEVMSHVTAHFGKALEECREESGLSAEVLEEFQHFWREDFEV

VHRELGCAIICMSNKFSLLQDDTRMHHVNMHDYVKGFPNGHVLSEKLVEL

IHNCEKRFDSMTDDCERVVKVAACFKVDAK

>1146410-1

FAVCVVLAQALTDEQKEKLKKHRSECLAETKPDEQLVNKLKTGDYKTENE

PLKKYALCMLIKSELMTKDGKFKKDVALAKVPNAADKPAVEKLIDACLAN

KGNTPHQTAWNYVKCYHEKDP

>1155060-1

SYMALAAHGQLHDEIAELAAMVRENCADESSVDLNLVEKVNAGTDLATIT

DGKLKCYIKCTMETAGMMSDGVVDVEAVLSLLPDSLKTKNEASLKKCDTQ

KGSDDCDTAYLTQICWQAANK

>1155062-1

LLQGALTDVYVMKDVTLGFGQALEQCREESQLTEEKMEEFFHFWNDDFKF

EHRELGCAIQCMSRHFNLLTDSSRMHHENTDKFIKSFPNGEILSQKMIDM

IHTCEKTFDSEPDHCWRILRVAECFKDACN

>118216-1

FTTFSVVASMGPFDPEEMLFIFTRCMEDNLEDGANRLPMLAKWKEWINEP

VDSPATQCFGKCVLVRTGLYDPVAQKFDASVIQEQFKAYPSLGEKSKVEA

YANAVKQLPSTNNDCAAVFKAYDPVHKAHK

>12247745-1

AVGNVDSSPEVMKNLCLNFGKALDECKAEMNLSDSIKDDFANFWVEGYEV

SNRDTGCAILCLSKKLDMIDPDGKLHHGNAMEFAKKHGADEAMAKQLLDI

IHNCENSTPPNDDACLKTLDIAKCFKKEIH

>1236279-1

AINLVHSSPEIMKNLSNNFGKAMDQCKDELSLPDSVVADLYNFWKDDYVM

TDRLAGCAINCLATKLDVVDPDGNLHHGNAKDFAMKHGADETMAQQLVDI

IHGCEKSAPPNDDKCMKTIDVAMCFKKEIH

>13235566-1

LGSIHALSSDEESSIKEALHPFVVECAEEYGITEEMFEEAKKKGSAEDID

PCFMSCFLKKAEFFDGAGKFDVEKTMSFAKSHITSEPAIKFLEAAGGACV

KINDEDVSDGDQGCDRAKLLFDCLMELKK

>13235568-1

SFSQAFASEEEKTAFREAIRPIVEECSKEHGVSHDELKSAQENQNADNIK

PCFLGCVYKKSEVFNSKGEYDVDKALEKLKGFVSNEAAYAKFAEVGKKCV

TVNDKPVSDGAAGCERGAMLTACFLEHKA

>15705901-1

GVWQVESSADVMKKLTTGFATALEKCRDELNLPDAVMQDFFNFWREDYEL

VNRDMGCAIMCMATKFDLVTEEQKLHHGNAHEFAKSHGADDSMAKQLVTM

LHECETQTASISDDCGRTLEIAKCFRTKIH

>15811612-1

LYQAVEPSQDVVKDMSLNFRKGLDACKKELNLPDTINSDFNRFWNDDHVV

TNRDTGCAIMCLSSKLELVSDTGLHHGNTLEYAKQHGADDTVAQQIVDLL

HSCAQAVPDLEDPCLKVLEWAKCFKAEIH

>159559-1

LTKGLYEKLGKDIRQKKKSYFEFCENKYYPAGSDKRQQLCKIRQYTVLDD

ALFKEHTDCVMKGIRYITKNNELDAEEVKRDFKLVNKDTKALEKVLNDCK

SKEPSNAKEKSWHYYKCLVESSV

>161691-1

SLILLVAVQAITEEDLELLRQTSAECKTESGVSEDVIKRARKGDLEDDPK

LKMQLLCIFKALEIVAESGEIEADTFKEKLTRVTNDDEESEKIVEKCTVT

EDTPEDTAFEVTKCVLKDKP

>16225958-1

TVDSVAKVYEAKPEIKKQEESFFAYCAKKALGANGKEGYKKIRDYELADS

AEFRNAMDCVFRGFRYMDDSGLKVDEVVRDFNLINKSDLEPEVRSVLASC

TGTQAYDYYSCLLNSSV

>16225961-1

LACGLFVIAQANTVKKCEKKMPASLKSQLCEIRKYKLLDTPDMDSHMDCV

MKALDFVRPDGTGDYHKLIKPLNAIEKDRKHDFNLEKCGGQTQHLPVGKR

ANAYYKCLVESTS

>16225965-1

LVWCLISLGQARKESTVEECEKNIGDSLKDRVCELRQYTPVSSDDMDKHM

QCVLEVVGFVDGNGEVKESVLLKLLQRVDSGVNHAANMKKCVTEASTSGS

DKKANTFYTCFLGTSS

>16225968-1

LVWCLFSLGQARQEETVEECERNIPASLKGRVCELRQYTPVQGKDMDSHM

QCVLEVLGFVEYNGELLFQELLGVLKMVDPDGDHAGSMKKCNAEAEKVDT

SSKANTFYTCFLGTSS

>16225974-1

TVEGAAKIYAAMPEIKQKGESFHAYCEKRAWKGNKQSEWKNGRRYKLTGS

PELKDAIDCIFRGLRYMDDTGLKVDEIVRDFNLINKSELEPEVRSVLASC

KGSEAYDYYVCLVNSRL

>16225977-1

STLCFVAAVQGGTVKECEDKMAASLKSKLCEVRQYKLFESQDMYNHIDCC

VKAVGFVNNDGSGDYHKLIKLLDKIKKSRKHGENLETCVGQSKRAGANQR

AYVYYKCLLNTNS

>16225980-1

SVALVTCGLLVIVQAAKKVEQCEKRIPDSLKPKLCQIRQYQLLEGADMEK

HIDCVMRALGFVHPDGSGNYHALIEPLNAIDKDRKHGFNLETCGGNRDKL

PKRKRAYAFYKCMLKSTS

>16225989-1

IYLAFFSLGFSEARFTNLGIEEFYIKKCEEKIVYTTDHGEICDMRSLEVV

MDTEENKNYIGCVFRELGYFNAKGQFDKQALIKDYHQAGVKNRDKAVLES

YQSCMQHYGPTTNPMKILDCVTQDKD

>16225995-1

LLGILFSLSMALEVSHFYICSTDYVARERNFLCHTANFKLVSLPPKGDEF

FDCCFQTSEWMDRGSKELKTNKFVSDMKKYGFDKRKAIEKVVQSCKTEMG

DKINGWAYFRCFVMDRK

>16519347-1

MSEEMEELAKQLHDDCVGQTGVDEAHITTVKDQKGFPDDEKFKCYLKCLM

TEMAIVGDDGIVDVEAAVGVIPDEYKAKAEPIMRKCGFKPGANPCDNVYQ

THKCYYDTDP

>16798386-1

CWMAFVQAGEPKTVEECEKNIPSSLKDRICELRQYTPDTSPDMDKHMQCV

LHVVGFVDRNGEVEFQELLGLLTIAEPRGKHAENIKKCVAESAKVNASKK

ANTFYTCFLTTDS

>17016228-1

LTVCLLALVQSETVQDCENKLPPSLKSRLCEIRRYEIIEGPEMDKHIHCV

MRALDFVYEDGRGDYHKLYDPLNIIELDKRHDVNLEKCIGECVQVPTSER

AHVFYKCLLKSTT

>17136918-1

GDVDDISTTTSAPREADYVDFDEVNRNCNASFITSMTNVLQFNNTGDLPD

DKDKVTSMCYFHCFFEKSGLMTDYKLNTDLVRKYVWPATGDSVEACEAEG

KDETNACMRGYAIVKCVFTRAL

>17473507-1

LLFCFMRGVHSADDLSKIPEIKGYELHCIEASGITESSAKKLRNGDDIAS

PDQSIKCYVQCFFSKLRLMNEKGVVQKDKVLSLLGKLMEEDKAKKLAEKC

DLRRTNPCDTAYAMYDCYRQNKA

>17647793-1

IVLLGAALVRAFDEKEALAKLMESAESCMPEVGATDADLQEMVKKQPAST

YAGKCLRACVMKNIGILDANGKLDTEAGHEKAKQYTGNDPAKLKIALEIG

DTCAAITVPDDHCEAAEAYGTCFRGEAK

>17737647-1

LLLPDPAVAMTMEQFLTSLDMIRSGCAPKFKLKTEDLDRLRVGDFNFPPS

QDLMCYTKCVSLMAGTVNKKGEFNAPKALAQLPHLVPPEMMEMSRKSVEA

CRDTHKQFKESCERVYQTAKCFSENAD

>17946714-1

ILAISAAELQLSDEQKAVAHANCALCAQQEGITKDQAIALRNGNFDDSDP

KVKCFANCFLEKIGFLINGEVQPDVVLAKLGPLAGEDAVKAVQAKCDATK

GADKCDTAYQLFECYYKNRA

>17981809-1

IFALVAFASASRDSAKKIGSQYEHYATCLTENDAAADDIFTILDITSGHH

KNENEHDKQHKNGCVMHCLLEKDGLMTGADYHEEKIREDYIKETGAQPGD

KRLEALDTCMNETKDMTDKCDKSLLLVACVLIAED

>18140725-1

TATAASQCFRDAGQLKRVVQAQEECVRYLRIPCARLAVYNKFIYPNDAET

QCMVRCMGLNLGWWNDTHGVQEASMRSFFHPDPNDCDYERRTYRCLHSQR

LDRPAPHDEACERAYESFRCYYEHYG

>18140729-1

ALMPLEVLSNDTKGLTIEKSFLQSVHDCAEYLQVPKHRLVQYLAYEFPPD

EETKCLIFCVGTDLRWWNNTCGLQVPEIMNYFQPVLGDRQYEKRTSECLE

RNVHTAELPNNCCQAYETFQCYFREFG

>18140731-1

FVYGAKNKPVFSEEIKEIIQTVHDECVGKTGVSEEDIANCENGIFKEDVK

LKCYMFCLLEVAGLADEDGTVDYDMLVSLIPEEYSERASKMIFACNHLDT

PEKDKCQRSFDVHKCTYEKDP

>18140735-1

LILFDASYAMSRQQLKNSGKMMKKSCIPKNDVTEDEVGQIEQGKFIEDRR

VMCYIACIYTMTQVVKNNKLSYDAIVKQVDMMFPPEMRTAVKTAAENCKD

IAKKYKDICEASYWTAKCMYDFDS

>18140737-1

FILADGVDSMSKQQLKNSGKMFKKQCMGKNKVTEDEIGEIDKGRFVEQQN

VMCYIACIYQMSQVVKNNKLNYEASLKQIDIMYPPELKDTAKGALEACKD

IAKKNKDLCEASFKTAKCMYEYSP

>18140739-1

SLLCVIYCALVHADTVAILCSQKAGFDLSDLKSMYEANSEEQMKKFGCFE

ACVFQKLHFMDGNTLNVEKLESGTRELTPDDFTEDVHEIIEQCVSKAADE

DECMVARKYIDCALEKMK

>18140741-1

TVVRGIDQDTVVAKYMEYLMPDIMPCADELHISEDIATNIQAAKNGADMS

QLGCLKACVMKRIEMLKGTELYVEPVYKMIEVVHAGNADDIQLVKGIANE

CIENAKGETDECNIGNKYTDCYIEKLF

>18140743-1

AIFVNAAPDWVPPEVFDLVAEDKARCMSEHGTTQAQIDDVDKGNLVNEPS

ITCYMYCLLEAFSLVDDEANVDEDIMLGLLPDQLQERAQSVMGKCLPTSG

SDNCNKIYNLAKCVQESAP

>18140747-1

AIEDTMSKKMTIEEAKKTIKNLRKVCSKKNDTPKELLDGQFRGEFPQDER

LMCYMKCIMIATKAMKNDVILWDFFVKNARMILLEEYIPRVESVVETCKK

EVTSTEGCEVAWQFGKCIYENDK

>18140749-1

FVALKPVKSMSADQVEKLAKNMRKSCLQKIAITEELVDGMRRGEFPDDHD

LQCYTTCIMKLLRTFKNGNFDFDMIVKQLEITMPPEEVVIGKEIVAVCRN

EEYTGDDCQKTYQYVQCHYKQNP

>18140751-1

IANVQRNDQGSMDDVDVEDIMNQCNETFKIEMAYLQALNESGSFPDETDK

TPKCFLLCVLDNTGVMTKDGDFDPERTAALFAGERAGKVMDGIQDMAAAC

ADRKEKCKCEKSYNYLKCLMT

>18140753-1

IAASPKKIYRIPPQASEKIVEEVLKCVQKMGLDSTVVNLLKEGKYTEDDR

VIETLMCSNQNVGNVNGDGKVNIDKVMNDIFSNKPEIRSALVACEKDGGK

SPLETFKNFILCFKEKVP

>18140755-1

VVGLNAHNVHLTDGQKEKANEPIAACIKETGIKPEVIAEAKKGHYSEDEA

MKKFILCFFHKAGIVNADGKLNLDVAIAKLPPGVDKTEATKTLEGCKDNG

GKDAADPAFAIFKCYKDATK

>1894778-1

VVLAQAADDDWVPKTPEEFNAIRRECHKEFPFSKELQKQEDELDFSDDET

VRKYEVCVFRKWGIIDADDTFHGERLVKQFEAVLDGVEGIEQKVNNCVDK

NEQGSPIDVYASRIQQCIDKTDIA

>19071272-1

DTTPRRDAEYPPPELLEALKPLHDICLGKTGVTEEAIKKFSDEEIHEDEK

LKCYMNCLFHEAKVVDDNGDVHLEKLHDSLPSSMHDIAMHMGKRCLYPEG

ETLCDKAFWLHKCWKQSDP

>19071274-1

RPAPRRDGQYPPPETLAFLRPLGKLCLEETGVSPEAVKRFSDADPFDDNR

ALKCYMDCMFRVTNVTDDRGELHMGKLLEHVPTEFEDIALRMGVRCTRPK

GKDVCERAFWFHKCWKTSDP

>19071278-1

CLIEHIDGAMTMKQLTNSMDMMRQACAPKFKVEEAELHGLRKSIFPANPD

KELKCYAMCIAQMAGTMTKKGEISFSKTMAQIEAMLPPEMKTMAKEALTH

CKDTQTSYKDPCDKAYFSAKCAADFTP

>19071280-1

IPFPSVECAMTRKQLINSMDMMRSACAPKFKVSTEMLDNLRGGIFAEDRE

LKCYTMCIAQMAGTMNKKGEINVQKTLAQMDAMLPPDMRDKAKEAIHSCR

DVQGRYKDSCDKTFYSTKCLAEYDR

>19071282-1

PALVHAQQSLTQADMDEIAKGMRKVCMSRPKISEEMANYPSQGIFPDDKE

FKCYVACLMDLTQTSKKGKLNYDAAVKQIEILPETYRQPFRLGLDSCRTA

ADDATDRCEVAYILLKCFFKASP

>19071284-1

MYIVLSAPFEIPDRYKKPAKMLHEICIAESGASEEQLRTCLDGTVPTAPA

AKCYIHCLFDKIDVVDEATGRILLDRLLYIIPDDVKAAVDHLTRECSHIV

TPDKCETAYETVKCYFNARD

>19921806-1

ALLGLASASDYKLRTAEDLQSARKECAASSKVTEALIAKYKTFDYPDDDI

TRNYIQCIFVKFDLFDEAKGFKVENLVAQLGQGKEDKAALKADIEKCADK

NEQKSPANEWAFRGFKCFLGKNL

>19922608-1

VTLAVGSSLNLSDEQKDLAKQHREQCAEEVKLTEEEKAKVNAKDFNNPTE

NIKCFANCFFEKVGTLKDGELQESVVLEKLGALIGEEKTKAALEKCRTIK

GENKCDTASKLYDCFESFKP

>19922612-1

AALSLASAVGLTDSQKAEAKQRAKACVKQEGITKEQAIALRSGNFADSDP

KVKCFANCFLEQTGLVANGQIKPDVVLAKLGPIAGEANVKEVQAKCDSTK

GADKCDTSYLLYKCYYENHA

>19922636-1

WLICILTVSVVSIQSLSLLEETNYVSDCLASNNISQAEFQELIDRNSSEE

DDLENTDRRYKCFIHCLAEKGNLLDTNGYLDVDKIDQIEPVSDELREILY

DCKKIYDEEEDHCEYAFKMVTCLTESFE

>20136465-1

VIAIVCYLFVATESFPSHSPELDTCSTKHHLSKDRARQLAIHSVQATDDN

EKCFLSCYLKERGYLVDGRVDFDKLLENHKKFQPTLFEELKKTFDECKKN

LDYNGKNECEVAYQAYSCAFT

>21064793-1

ATSAKPHEEINRDHAAELANESKAETGATDEDVEQLMSHDLPERHEAKCL

RACVMKKLQIMDESGKLNKEHAIELVKVMSKHDAEKEDAPAEVVAKCEAI

ETPEDHCDAAFAYEECIYEQMK

>21357379-1

IALCLCLSLNEGLALLEHEGETINRCIQNYGGLTAENAERLERFKEWSDS

YEEIPCFTRCYLSEMFDFYNNLTGFNKDGIVGVFGRPVYEACRKKLELPF

ESGESSCKHAYEGFHCITNME

>21483542-1

AVLWICLITMWQSAGRVNAEGCLKHHNLSSAQVQAVAPSTPVADVPVAVK

CYSRCLIQDYFGDDGKIDLQKVGKRGSQEDHVILSQCKQQFDGVTNLDTC

DYPYLILQCYFKGKQ

>21630066-1

VTLPTCFVQAGPIKDQCMAAAGITAQDVANRHETDDPGHSVKCFFRCFLE

NIGIIADNQIIPGAFDRVLGHIVTAEAVERMEATCNMIKSETTYDESCEF

AWQISECYEGVRL

>21630078-1

HLACIIFILEIQFRIADSNDPCPHNQGIDEDIAESILGDWPANVDLISVK

RSHKCYVTCILQYYNIVTTSGEIFLNKYYDTGVIDELAVAPKINRCRYEF

RMETDYCSRIFAIFNCLRQEIL

>21898675-1

SGDKIISDQQKSDMEECAKQYGVEKPAAGGPPKGGLEEMKKKMACAGQCL

GQKQGLLDSDNYVDVDKFSASVAAVVTDSDIKALAEETAKKCAQEANEKA

KASGEVDYNGTKCNL

>21898677-1

LAVATATLADSTQSYKDAMGPLVRECMGSVSATEDDFKTVLNRNPLESRT

AQCLLACALDKVGLISPEGAIYTGDDLMPVMNRLYGFNDFKTVMKAKAVN

DCANQVNGAYPDRCDLIKNFTDCVRNS

>22026979-1

CLLLNFLCANVLANTSVFNPCVSQNELSEYEAHQVMENWPVPPIDRAYKC

FLTCVLLDLGLIDERGNVQIDKYMKSGVVDWQWVAIELVTCRIEFSDERD

LCELSYGIFNCFKDVKL

>22138790-1

TLLEHGQKVMEQAVDAGAKCAEELGATPEDLDKLAKRELPETKAGRCVIT

CVNKIFGLQNADGTIKKDSTLANVEEVKDIDEDVYKKMASVWTTCSSNTV

NDSDECNTGVNLAKCMKESSE

>23320747-1

LLGVADLASGLTGRAFERAKEVDEKCRSENNVERAYFEKFIKARIDEIDP

PDNYKCFVKCVMVELMALNDEGDFNVDEELQNVPPEIVEEGHRIVKTCHG

TPGKDPCDKAYQVHKCYHKENP

>23320749-1

VLVNAVPASEAEVDVKEIMKKCNESNPIDAEYLDQLNMTGSFPDENVRSA

KCFIRCMFMETGVMDSDGKLVAAKLKEAFGKRQGPVAVKTADLEMFVDNC

IAADADVTCQCERAYRFSKCLMTEEL

>23320751-1

VFAARVHAYDSTLNDVRIQCNETFPISYEYDVHLMNFGSFPDESDQTSMC

FIHCVMDKTGMMDTEGTFHKKTVVEKLQGFPNDTEIPDLEEIVEHCVTET

DQEELCERAYGFGKCLMIEEI

>24158424-1

ATADEEEGSMTVDEVVELIEPFGDACTPKPSRENIVEMVLNKEDAKHETK

CFRHCMLEQFELMPEDQLQYNEDKTVDMINMMFPDREDDGRRIVKTCNEE

LKAEQDKCEAAHGIAMCMLREMR

>2444185-1

LVMRVDCSKEVMKQMTINFAKPMEACKQELNVPDAVMQDFFNFWKEGYQI

TNREAGCVILCLAKKLELLDQDMNLHHGKAMEFAMKHGADEAMAKQLLDI

KHSCEKVITIVADDPCQTMLNLAMCFKAEIH

>2444187-1

LVIPVEPSKDVMHQMALKFGKPIKLCQQELGADDSVVKEFLDFWKDGYVM

KDRQTGCMLICMAMKLELLDSAMEIHHGSTFAFAKAHGADEAMAQQIIDI

VHGCTTTYPAAETNDPCQRAVNVAMCFKAHVH

>24580976-1

FVLLLGLSVLATKEPEEVKIVSECAKENNVHRKKALDLLMSYRLKKKTHN

VMCFINCIFERTNILQKVKEKVVKENHNCDSIKDADKCAESFQKFQCLVK

IEMK

>24643503-1

GPIPQSEAGVTEEQMWSAGKLMRDVCLPKYPKVSVEVADNIRNGDIPNSK

DTNCYINCILEMMQAIKKGKFQLESTLKQMDIMLPDSYKDEYRKGINLCK

DSTVGLKNAPNCDPAHALLSCLKNNIK

>24643505-1

PIWAVIDRNLPQVQELVTAARMECIQKLQLPRDQRPLGKVTNPSEKEKCL

VECVLKKIKLMDADNKLNVGQVEKLTSLVTQDNKMAIAVSSSMAQACSRG

ISSKNPCEVAHLFNQCISRQ

>24644477-1

AQEPRRDGEWPPPAILKLGKHFHDICAPKTGVTDEAIKEFSDGQIHEDEA

LKCYMNCLFHEFEVVDDNGDVHMEKVLNAIPGEKLRNIMMEASKGCIHPE

GDTLCHKAWWFHQCWKKADP

>24644509-1

VATFLVAQTTAKFLLKDHADAEKAFEECREDYYVPDDIYEKYLNYEFPAH

RRTSCFVKCFLEKLELFSEKKGFDERAMIAQFTSKSSKDLSTVQHGLEKC

IDHNEAESDVCTWANRVFSCWLPINR

>24651098-1

ALCAVAHADDWTPKTGEEIRKIRVDCLKENPLSNDQISQLKNLIFPNEPD

VRQYLTCSAIKLGIFCDQQGYHADRLAKQFKMDLSEEEALQIAQSCVDDN

AQKNPTDVWAFRGHQCMMASKI

>24651100-1

MAVSTEAASVWKLPTAQMVYEDLEKCRQESQEEDAATLRCLVKKLGLWTD

ESGYNARRIAKIFAGHNQMEELMLVVEHCNRMEQDTSHLDDWAFLAYRCA

TS

>24651102-1

LADHHHHHHDYVVKTHEDLTNYRTQCVEKVHASEELVEKYKKWQYPDDAV

THCYLECIFQKFGFYDTEHGFDVHKIHIQLAGPGVEVHESDEVHQKIAHC

AETHSKEGDSCSKAYHAGMCFMNSNL

>24652587-1

INLGLTVADESPKTITEEMIRLCGDQTDISLRELNKLQREDFSDPSESVQ

CFTHCLYEQMGLMHDGVFVERDLFGLLSDVSNTDYWPERQCHAIRGNNKC

ETAYRIHQCQQQLKQ

>24653777-1

VFIGLVLLLAVTTLSSALFESEANECAKKLGITPDYFENFPHSSRVKCFY

HCQMEKLEIIANGVVTPFDLKVLNISPESYDKYGVKVKPCLKLSHRDKCE

LGYLVFQCLKREFN

>24655939-1

FLIFALSELVAGQSAAELAAYKQIQQACIKELNIAASDANLLTTDKEVAN

PSESVKCYHSCVYKKLGLLGDDGKPNTDKIVKLAQIRFSSLPVDKLKSLL

TSCGTTKSAATCDFVYNYEKCVVKGIS

>24655956-1

FLLFIFISAIWLQAFCMKSSEKIKACLKRQLGYTITENTKFDAKEDSLQS

KCFYHCLLEVKGVIANDAISSEQPRKVLEKKYGITDTDELEKAEEKCHSI

KASGKCELGYEILKCYQSITK

>24656242-1

SLAKARHPFDIFHWNWQDFQECLQVNNITIGEYEKYARHETLDYLLNEKV

DLRYKCNIKCQLERDSTKWLNAQGRMDLDLMNTTDKASKSITKCMEKAPE

ELCAYSFRLVMCAFKAGH

>24658435-1

HYCCKHPDGHNDLIEGCARETNFTLPNQNEEALVDITADRAIRGTCFGKC

VFSKLNLMKDNNLDMDAVRSLFTERFPDDPEYAKEMINAFDHCHGKSEEN

TSMFLSKPLFKQMS

>24663268-1

DLIPSNQGVEINPTIIKQVRKLRMRCLNQTGASVDVIDKSVKNRILPTDP

EIKCFLYCMFDMFGLIDSQNIMHLEALLEVLPEEIHKTINGLVSSCGTQK

GKDGCDTAYETVKCYIAVNG

>2597821-1

LFIFTVASLDGEMAELAKMIRDNCVDEIGVDVTLLEQVDAGANLMPDEKF

KCYLKCTLETAGMMSDGVVDIEIVLELLPEDLKTKNENLLRKCDTQKGSD

DCDTAFLTQVCWQNGNK

>2597955-1

AALGAARAVAMDEDMAELARMVRENCAAETGADVALVERVNAGADLMPDD

KLKCYIKCTMETAGMMADGEVDIEAVLALLPPELAEHNAPSLRACGTVRG

ADHCDTAFRTQQCWQNANK

>25990272-1

VASSVMASKELITKMSSGFTKVVDQCKNELNVGEHIMQDMYNFWREEYEL

LNRDLGCMVMCMANKLDLIGEDQKMHHGKAADFAKSHGADDDQAKQLVGI

VHDCENTHQGVEDACSRALEVAKCFRSKMH

>26007502-1

LFITVECSQEIMKNLSMNFAKPLEDCKKEMDLPDSVTTDFYNFWKEGYEF

TNRQTGCAILCLSSKLELLDQELKLHHGKAQEFAQKHGADEAMAKQLVDL

IHGCTQSTPDVAADPCMKALNVAMCSKTKVH

>27414019-1

SLVPPGECLDISKVTLDAAFYPLFGCARDLVVPEDLIELYKKRIFPDDQL

TCCVFRCLGMRLGIYDDVKGFDVDKQYERVKDRLSVDEDTYKRGVKNCIR

NVLRGRTLNNCEKAYLILNQCQGNTIT

>27414021-1

QQDPVPATSTFIVSDFLQFLQTAVTCFNKLRIPEERFPLYLAGVFPNCPE

TQCFVRCLSANLNLYCDETGSDIDRHYLQYGLGQDYNCFRQKAEQCLAAN

TSPCNDPCEAAYKQELCFLDEFR

>27414023-1

VCRVQAGSAEELEQAKEMLRGLAAECKTKEGATDEDVEGFVNDKMPESRT

QKCLAGCMQEQFGVSNGKAFQEDGFIEIAKMLMKGDETKIELAKEIAADC

KAVANDDRCELAVDIMNCLKESAE

>27414027-1

ATASANAPKSLSPELLQQMGQFRSECLRETGTTDEQIEQFNSPQSVQASH

ELQCYMYCMFRLHNVTRPNGELDLIDVYHAIPKQFNSIALKVLAKCNKST

GPIADACERAYSHHRCWKETEP

>27414039-1

SNYGIITQEQLEKTARTFRQVCQPKHKISDEVADAVNRGVFADTKDFKCY

VSCLLDIMQVARKGKVNYEKSLKQIDTMLPDHMKPAFRAGLEACKSAAQG

VKDHCEAATILLQCFYKNNP

>27414041-1

KIFPLRKEQMMKSGEMIRSVCLGKTKVAEELVNGLRESKFADVKELKCYV

NCVMEMMQTMKKGKLNYDASVKQIDTIMPDELAGPMRAALDICRTVADGI

KNNCDAAYVLLQCLSKNNP

>27414043-1

IVFVVLLAAVSTMEQHEIAKSLAEQCRAELGGELPEDFATKMRLGDLTLD

SETAKCTIQCMFAKVGFTLESGAANRDVLIAKLSKGNPTAKAEAFADVCE

NNEGETACDKAFSLYQCYHKNKS

>27414055-1

FVFPSPLQGARLEAEHVRRIHQNARECVKETGILPKNAFRVLSGDFSVDT

MKAKCFVKCFLDKAGFIDDDGVIQQDVIREKLTVGIEAGKVNELIKKCSV

EGTDACDTAYQMYKCFFSNHK

>27414057-1

AAAPPPDLEDVSKIANGEAFALECLIESGLKLDSLAALSAKELDTNGSKI

KCLVKCFFEKTGFMNKDGQLQEETITEQLSKFMPRERIESLVKNCNFQEA

DACETAYKVTECYFQNKA

>27414063-1

LLAVCAAAQPLTDDQMKKAEGFALGCLEQHKGLNKEHLVLLRDGDFSKVD

ADTKCFLRCFLQQANFMDAAGKLQNDYVIERLSLNREKSKVEALVKKCSA

GVEVEDSCETAFRAVECYHREKA

>27414067-1

LAFTAVVSAEFVVQTREDLLAYRAECVKSLGVSDELVEKYKSWNFPEDDT

TQCYIKCIFNKMQLFDDTNGPIVDNLVVQLAHGRDANEVREEIVKCAGSN

TDGNVCHWAFRGFQCFQKNNL

>27414069-1

GDNRTVHTLTIHSHMTVSLHCSLSLSLSLLSPSFSPIWQCFVQCFFQKLR

LMDEKGVVLKDKLEVFLTKLMDADKAKDYVQQCDLRRTNPCDTAYAVYDC

YLGKKA

>27414075-1

PFSIFQTHGAYVVRTFADATAYRDECVQQYAGRGSSLIDYMRQVALHTDN

ADSRWCIVRCILQKADLLDGEGAPHEANVHAQMQHSNAIVEDPDDIRSET

SRCLREPPAPDSGGGCLRAYTFFACIQSTE

>27414079-1

TILGVEAYATPPPTTANCTTVSTFDAALQECVVQLGIAPERLDQEYNLLL

YPADRDTMCLVRCIGVLLRFWNDTTGLREATIRQYYEPAPEDQDYQNRTR

SCLAALEPSVTDVCERAHRSFLCYH

>27414083-1

VFVPLDHLQLQHVTSRCMDVHQITTEQLMSLSAEAMDANDKLHCLVRCIG

LQTGVYSDREGVSIDRLNAQYGEGHCEKEFKTHAVECITKHRELAYGSPC

KRAYHLLYKCFENVRN

>27414089-1

SPLPHYFVRKSFPEAQAECAVYLQVPDDRLQRYMREGYPDEPEVHCLVLC

VLENLRAWENGTLHENVLANYFVPATEDCDNAKRTERCLVNLPQECNGEP

CVQAYRAFQCYYQNYG

>27414093-1

LAVGAVSVSASLQHYVVEKSFNQAQAECAEYQGVHDDDLLRYVKEGYPDV

EEVRCLLRCVAFNLRFWNHTTGLQKNMVAGHFVPYPDDFHNVERTEACLA

ENLYTCDDDLCTQVYKAFQCYYQYYG

>27414101-1

ATFGARDPPPPALREAQAACVKYLGICENRLHQYNNSVYPTDQDTMCMVR

CAGIMVGFWDDCQGLKLDGLANLFPALAANDRVRYQIMSCAEKRIATCPP

QDTCARAYNGFRCFLDAQK

>27414105-1

PTGCDASLDVPHLTLSKSFSRALQDCMEYLQVPGYRYAEYAANSFPDDPE

TKCLLRCVGLNLRWWNDTTGMQTAVIEGFFHPDPLDELYENRTAECLRKE

LSHADTTDCCCLAYDSFRCYLQHYG

>27464446-1

SVRKAEPSKDAMQYITSGFVKVLEECKHELDLNEQILADLFHFWKLEYSL

LGRDTGCAIICMSKKLDLLDANGRMHHGNAAEFAKKHGAGDEVASKIVTI

IHECEKKHEQDGDECLRVLEVAKCFRTGIH

>28571271-1

GPIPQSEAGVTEEQMWSAGKLMRDVCLPKYPKVSVEVADNIRNGDIPNSK

DTNCYINCILEMMQAVPGVDPQADGHHAAGQLQGRVPQGHQSVQGLHRRP

EERPQLRSRP

>28573568-1

LFCIALAAFLSMGQCNPDFRQIMQQCMETNQVTEADLKEFMASGMQSSAK

ENLKCYTKCLMEKQGHLTNGQFNAQAMLDTLKNVPQIKDKMDEISSGVNA

CKDIKGTNDCDTAFKVTMCLKEHKA

>29501378-1

VVQVHFKNSYSHFKTKKISTYQGGTIKECESKMAASLKKKLCQVRQYKLF

ETTDMYSHIDCCMKAVDFVEKDGTGDYHKLYELLNNIEKHRKHDINLETC

VGESMDAQANQRAYAFYKCLLKSTS

>31322218-1

DEMKELAQQLHNTCVSETGTTEDAITNARAGTFTDDEKFKCYLKCLLDQM

AIVDEEGRIDVEAMIAVLPEEFQDSLPPVIRECDTIIGANACDNVWLTQQ

CYYKENP

>31442894-1

SDIAVKKYLHAVPEPVLAKCLKESGLEADKDKLLSDESTVDQGKFSCLIA

CTLKDNGALVNGELKYDVLSELLSKLLTNKEDKLQERLELLKACIPEGAN

AKNDCEYIGKIMQCKLSKAK

>31747543-1

SQEVVASFSKGFTNVVEHCKAEVNAGEHIMQDIYNFWREEYQLVNRDLGC

MVLCMANKLGLIGEDQKMHHAKAEEFAKSHGADEAVAKQLVAILYECETS

TRPVEDECGWRLEIAKCFRTKMH

>33356124-1

LVLVALVAATYAETPREKLKQHSDACKAESGVSEESLNKVRNREEVDDPK

LKEHAFCILKRAGFIDASGEFQLDHIKTKFKENSEHPDKVDALVAKCAVK

KDTPQHSSADFFKCVHDNRS

>33356150-1

VVAFVAAVYAETPQQKLRQYSDACLSVSGVSQESLRKVRNREHVDDPKLW

EHAVCIVQKGEFIDSNGDFLVDNIKTKFKQDYDHPEKVDDLVAKCAVKKD

TLQNTCFEFVKCIHRNRS

>33356152-1

AQALTDEQIQKRNKISKECQQVSGVSQETIDKVRTGVLVDDPKMKKHVLC

FSKKTGVATEAGDTNVEVLKAKLKHVASDEEVDKIVQKCVVKKATPEETA

YDTFKCIYDSKP

>33356158-1

LVAAVATAQTLTDEQKANWKKWSDECREETGVSPEGINQLRTNHFDNVDD

KIKAQSLCFGRKSGLINEAGDVLADQIKIKLKRVAVDDEEVDKIANKCVV

KKDTPEETAFETFKCLREQKP

>33518701-1

LVIAYVNGAATQSPVKEKVAALAKKCSADHKATPEQTKLVFSQKVPSDEV

ERCLLECVYTGVGVIQGGEFSAEGSKKLATLRFSDPKERETVNKLVDTCT

KEVTKNKDEKCSLGKSVRECFVKH

>3644030-1

AALTCVMAGELPEEMREMAQGLHDGCVEETGVDNGLIGPCAKGNFADDQK

LKCYFKCVFGNLGVISDEGELDAEAFGSILPDNMQELLPTIRGCAGTTGA

DPCELAMNFNKCLQKVDP

>37778929-1

MAAQIKDNLELPEYYKRPAKILHNICLAESGAMESKLKQCMDGVLHDDRE

VKCYIHCLFDKVDVIDEATGQILLDRLAPLAPDNDVKDVFNHLTRECGHI

KLQDSCDTAYEVAKCYFAAHD

>38350655-1

ILLFKPITARFTPLGIDEFYIKPCERKIVYTTDKHDKCLMRRLEIEMDTG

ENQGYVKCVFKEFGYLNGEGQFNKQALLKDYHQAGFKNKDKAVLESYDGC

MKNYGPTPNAMKILDCVTKDKD

>39579195-1

SLSGVHATAEEKAAFIEAVKPHIQECSKEHGVTPEEIKSAKAAGNADGIN

SCFLSCVYKKAEVINDKGEYDADKALEKLKKFVSNEDDYAKFAEIGKKCA

SVNEKSVSDGDAGCERAALLTTCFLEHKS

>39579207-1

IYLLIALKVANGETLRESLRPVIVACSKEHGVTDEEIQAAKEAGSPASIK

PCFIACVFKKAGFLDDQGQIDIETGLKNLRQFVKDDEQYKKLEEVSKLCS

FVKDKVVSDGAAGCEKGALLAGCFLDHKT

>39840707-1

PAAAQRDENYPPPGILKMAKPFHDACVEKTGVSEAAIKEFSDGEIHEDEK

LKCYMNCFFHEIEVVDDKGDVHLEKLFATVPLSLRDKLVEMSKGCVHPEG

DTLCHKAWWFHQCWKKADP

>40019429-1

LVVLNVQFVTAADNNESVIESCSNAVQGAANDELKVHYRANEFPDDPVTH

CFVRCIGLELNLYDDKYGVDLQANWENLGNSDDADEEFVAKHRACLEAKN

LETIEDLCERAYSAFQCLREDYE

>40019433-1

GLTACSFAFTEHGAIVQSIVQAQHECVTYLNLPKHRLYQYLMYNYSNDAK

TKQMLRCVGLILQWWKSDGTLNEHVLAQYFMPDTSDSDYYNRTYRCIERK

APVDDDLCSRAFETFQCYLQQYG

>40204878-1

CTLQLESLALLDHETESIEKCIKNYGGLTSETAERLERFKEWSDGYEEIP

CFTQCYLAEMFEFYDNHTGFDESGVVQLFGRPVYNACRQRLELGGGRTQS

SCEHAYAGFHCITNLEG

>43439928-1

LTSAALGSQELMMKMTKGFTKVVDECKAELNAGEHIMQDMYNYWREDYQL

INRDLGCMILCMAKKLDLMEDQKMHHGKTEEFAKSHGADDEVAKKLVSII

HECEQQHAGIADDCMRVLEISKCFRTKIH

>45551127-1

TLVPDPPNGTENKLSQEMLRACMRRTEISMSQLKLFHMSLMNSDYNNDND

IAPTPVQSIGDCFVSCLYETLDLDRYNVLLEEAFKNQVQTIIQHEKAEIK

ECSDLQGKTRCEAAYKLHLCYNH

>45552755-1

TLVPDPPNGTENKLSQEMLRACMRRTEISMSQLKLFHMSLMNSDYNNDND

IAPTPVQSIGDVNNLGDLDFNGNSQMPYLDLKHNEPLQCFVSCLYETLDL

DRYNVLLEEAFKNQVQTIIQHEKAEIKECSDLQGKTRCEAAYKLHLCYNH

>4884922-1

KFDDSIISDDIKKLLKGLHDVCVGKIGVEEALIENLKNAEFTEDDKLKCY

VHCLLIQVGAMDLAGHIDAEAAIELIPEQIRVSVIQEANKCAKDKEKIEN

HCSRAFATIKCLHDVNP

>48994192-1

GAAMLADPATVKQVPEVTMQDAIAQCNRSFIIQPEYLAELNQTGSFPEET

DKIPLCFIRCYLKALGILTEDDKVNKEVALARNWATSGETVDECLEEMAG

SACEQAYFFTRCVMTRAL

>48994194-1

CPRTRSACAETFPSTRRRRGALCMHSEGAGKARLSKEFFGLVMVCFVKCF

LDKAGFIDDDGVIQQDVIREKLTVGIEAGKVNELIKKCSVEGTDACDTAY

QMYKCFFSNHK

>48994196-1

SAFIACAVATISEEQREAARQLAGKCMQQTGASEDDVNRLRSGDTEGADR

NTRCFVQCFFQGAGFVDQDGSVQTDELTQKLASEYGQEKADELVARCRNN

DGPDACERSFRLLQCYMENRA

>48994200-1

VVALIAGTFALTIDQKKKAEGYAAECVKTTGVPPETAAKLKGGDFAGADD

KTKCFAKCFLEKAGFMTDKGEIDEKTVIEKLSVDHDRAKVEGLVKKCNHK

EANPCETAFKAYQCIYAAKG

>48994202-1

FFLLVASVHAFTLRQQKMVSIFALECMAETGIGAESLTKLRDGDLTANDR

TAKCFMKCFFEKENFMDAEGKLQLEAIATALEKDYERAKIDEMLEKCGEQ

KEDACETAFNAYACYHDH

>48994204-1

SDEPRRDANYPPPELLEKMKPMHDACVAETGASEDAIKRFSDQEIHEDDK

LKCYMNCLFHQAGVVNDKGEFHYVKIQDFLPESMHLITLNWFKRCLYPEG

ENGCEKAFWLNKCWKTRDP

>5081559-1

AILGAECSQDVMKQMTINFGKALDTCRKELDLPDSINADFYNFWKEGYEL

SNRXTGCAIMCLSSKLDLVDPEGKLHHGNTHEFAKKHGADXXMAKQLVEL

IHKCEGSVADDPDACMKVLNIAKCFKAEIH

>51557689-1

VLCAPPSGDQYDTDNLLKVRECEEEKDLKEPEKTEWWAWKVPSNPTECYI

DCILQKYGWLSGSGGSVVNSAIEESYAAVGHSNPSLTQCNLTKTGCSKAD

ELYECLLNADG

>51557693-1

ALLLLNLIALSLAGTRGTSRCEKRNKLDRATIQKYRNWQIPTKFKNNNEK

CHLHCVFKQIGWMRGHYIMDAQIGNDIDAKKEFTQKTPHLRTLLFEDCNI

NERSLTDKCGKAIQLYTCLVQKFK

>51557724-1

YNLPNEQYFNTTKLWSIRKCERDLKLNDAERQIWWPWKVPDHPTKCFAQC

VFKSVGWVQDDGWRINFSQLHHSYRLMGHRILPTYKIMLKRCRKSPTRDN

CEEYTLRWKCLLQLNE

>51557740-1

TFIVEISCYNLPDESYYNTTRLWGIRDCERKFKLSDDERQFWWSWKVPPN

PTKCFAQCVFHAIGWYDSNGRFNLARVQWHYRQMGHRITPNKYKMRRCMR

RNDPCNNVTYLWKCFLDFNA

>51557760-1

VLLLTVAIAYIPNHVECSNMRTEVHHQCLAKVLPGKTIEEASWDQVKKEA

IDNGNRDYQCFILCELTNLNMLKSNGVVQTDESPLHPALGAKLTECANMK

VDADSCKNAKDSAQCIINVTA

>51557796-1

MVFILNSGLVSANKSANEQCVEKTLPGKTLSDVKWSKVQSEAFMKDNREY

QCFILCGLSNLKILKSTGAVETINNPLESELGDVIKTCAQETPSDDACKT

AKRSALCLFAKAG

>51557810-1

SYVPLDVEYDTEDLKKVKECEEQKNVPEEELAEWWEWKIPKNPTPCLVDC

ILTKFGWLSSDGEVVTTAIEKSYNAVGHSNPSLSACKPTKTKCAKAEELF

ECLLNTDG

>54636338-1

MNECLYFSHITLEELQAQMNISSSEEDLENLDRKYKCFAHCLVARANLLD

SRGRVDVAKIDELEPLTDEHRQALENCKRAHDDEPDNCEYAFSMFLCLSD

YLE

>54636346-1

FNPCRGQKFTTPEEQRAFLDNWLKNLEGRDLDRTFKCYATCMLFDMNIMD

GSGELHMEKYLETEALEKSWPKSVANCRYEFADEMDICEYGFGMANCLMS

LRA

>54636612-1

FLLSKGVSLQSQSAADLAAFKQNQESCIKELKIGAAEAALLTTDKEVANP

SEAVKCYHSCLYKKLGMLTADSKPINDMIMKFAQLRFSSLASDKVKALLT

SCGASKAATTCDFVYNFERCVVKGAK

>54636616-1

CLTASFVKATDVKNPEEIMKKCVTDLGVTEQDVLDLKLGKVKPADVKDNV

KCTAQCVMVASGYMDAKGNLLKDKIKKQYADHPQKDVINKALEVCGGVKG

ANACDTAFQIVYCV

>54637494-1

CVLISLASAEYVLKNRDNMLAYRDECVKELGVPADLVEQYTKWQYPNDAK

TQCYIKCVFTKWGLFDVQTGFSVENIHQQLELGHADHNEAMHSSIAACVD

KNEQGSNACEWAYRGATCLLKEHL

>54637500-1

CLLLLAVPSTRAASDWQLPSPEQVYKDLDSCRLQSLSDGQDAETLRCLVE

KLGLWSDESGYNSKRIAKIFAGHNQMEELQLVVGYCNRREQRLNQPNEWA

YQAYKCATSGRF

>54641710-1

GLLPHQGLAVTMEQMMQSMDMLRGACQPKFKVTTETLDRIRAADFSMEHT

QDLMCYTRCIAQMAGTVTKKGEFSAAKAYAQLPIILPPEMLEAGKASVDA

CRDVQKPYKDSCEKIFYTTKCMSEVDP

>54642148-1

ELSPTMKKQVGKLKDRCIKQTGASAERMLQAKKDQVLPDDPAFKCFLHCM

FDMLGLIDSHNVMQLESLIEVLPEEIHPEINALVGNCGTQKGIDGCDTAF

LTVKCYLSVNK

>54643936-1

CLVAVSESESKPQDASNEEHVAEAANECKAETGATDEDVELMMKHEPSVN

EEGKCLRACMMKKFKIIEEDGKPNTEHTIEMMKLMSENAEEKEDAITEIA

DTCAAIDLPEDHCDSADGYLKCIIEHME

>54643937-1

WTVMVRQLPHVQDMIDTGRRECIKELKLPKDQRPLMRVSNPSEKEKCLIE

CVLKKTKIMDDKNKLNLAQVEKLTGLVTQDNKMAIALSCSLAQTCNRSIS

AKNPCEAAHQLNQCISRQ

>56417448-1

AILFCDGKSAEKYYKDKGKNVKQKGESIFVHCEEIHYPVGSPQRNELCKV

RKYELGTGKPFENLMECIFKGVRYFNDKNELNIDEIARDFTQVGKKPDAV

KAAMENCKSKTKETDPGKKAVEYYKCLLADSK

>56417450-1

LALVTAKGPFDPEEMHFIFTRCMEDNLKDGPDRVKTLLKWKEWVTEPKDD

PATHCFAKCVLEMSGLYDAASGKFDASVIEAQHKAYPNSEDKGKVDAFVK

AVQALPPTKNDCTAVFRAFGPVHMAHK

>56417522-1

ILLTLCMCNAESHFKNCAEEQLSDDKPLQCKIKSLQVDGNMPKVKDYMTC

AFEASGWMPKGSNKLDTSKIAEDMTPNGFSIKNNLDEVAKECEGEFGAEI

SAIDYLACLLIDEK

>56462374-1

VLSPEVTAFLKGVIEECIEETGVVPNILELLKADNYVADDKNKSFLACGY

RKAGALDSEGKLHPHKIASYFPDELNVLEYFQKCNKHEDEVKETAYQSYE

CTKVTLP

>56805551-1

AILCVFGAVLVKGFDKKAAIAAFLAKMDDCKAEVGASDSDVEELVGKKPS

STMEGKCLRYCLMKKYEVMDDNGKFVKDIALTHAQKYTDGSEERMKTATE

IIDTCSNLEVADDNCEAAEQYGKCFKEQVI

>57907214-1

RLDLVCLLAIVLLVHSCRNEFEIEPSVFESLRAGNFSVRNSLVECFGECF

VKRAGFMNDNFTFNRDTIMRFTNRFVSKEISEKVYNICTDNVTPTYCVTA

FDVYQCIYENVY

>58618234-1

IFALVAFASASRNSAKKIGSQYDHYQTCLTELGVTEDDLFSIGEVTSGQH

KTKHEDTKLHRNGCVMQCLLEKAGLMTGADFDEEKMRENYIEEKGLQPGD

QRIDFLNSCMEQTKDIEDKCDKSLIFIGCVLMNEVS

>61742027-1

ATILIFSVFVASIKAKGIYQSCADEKIKPGNEYKDYILCKASAFLVERPG

DSIYPGMKEFMDCTFIKAGWMDKTSHALNVSKIANDLKTSEYPDRQNQIE

ELNKLCKNIYDPPLNAMNYLDCIALRP

>62530473-1

MTSQKVINNFSKGFKDVVDHCKAELNMGEHIMQDVNNFWREEYQLVNRDF

GCMVPVHGQQARAPKRGPEVHHDKAEEYAKKHGADDATAKQVVAIIFECE

NNSSGMDDECNRALEIAKCFRTKMH

>6272606-1

IDSGVDSSQEVMKNLTMNFNKALDVCKKELDLPDSINADFYNFWKPDYEV

TNRLTGCAIMCLSTKLELFDPDGKLHHGNAKEFAMKHGADDSMAQQLVDI

VHKCENDIPNNEDPCLKVLDIAKCFKTEVH

>6272646-1

RLDWAMASQDIMKKLTVGFSKALDQCKTELAIQENVLQDFYNFWREDYTL

VNREMGCVLMCMASKFDLITEDMKVHHKNAHEFAKTHGADDEMAKQLVSM

IHECEKTHEGVVDDCGRVLEMAKCFKTKIH

>6521353-1

KEHGQKVLEQIIDYATSCADSLGVSPEDMKLLMEKKFPTSREGQCMPSCV

NKKFGLQKADGTLNKEYRYSEMENVKAIDEEIYNKMNSVWDKCVINGADG

TDECDTGMKVVTCMKEESE

>6560639-1

LTLLAAGALALDEEQAELARMVRENCVDEIGVDEGLLAKVDDGADLMPDP

KLKCYLKCTMEMAGMISDGVVDVEAVLGLLPDDVKLRTTDIVRACDTQKG

ADDCDTAFLTQTCWQQANR

>6560649-1

AVKEIAPSSDAMRHIANGFLKVLDQCKHELGLTDQIVVDLYQFWKLQYAL

LNRDTGCAIICMSKKLDLLDSTGRMHHGNTQEFAVSHGATDEVASKVVVI

IRDCEKQQEGEEDDCVRVLEVAKCFRTAIH

>6560665-1

TMEMVSASQEVLKQMSVGFSKVLQTCKTELSVGDHIIQDFYNYWREDYDL

LNRDFGCMVICMAVKHDLINDQLTMHHGNAHAFAKTHGADDDTAQQLVTI

LRECEAKHQSVEDVCNRALEMAKCFRTKIH

>6560671-1

AVSVGAVSENERNQISQSILPHIVKCSQEYGVSEGQIKDAKESVNPLGLN

PCFLGCVLKSAGIIDKNGLFDVEATKEKSKKYISSEKDVTNFDKIIKDCT

EVNQKNVSDGNKGCDRAKELVTCFLAKRG

>66772982-1

CLSGILAQANIDSSVSKELVTDCLKENGVTPQDLADLQSGKVKAEDAKDN

VKCSSQCILVKSGFMDSTGILVKSGFMDSTGKLLTDKIKSYYANSNFKDV

IEKDLDRCSAVKGANACDTAFKILSCFQAAN

>6682279-1

AVVLAQAQDDWKIKSANEVNDIRRECHKEHLFNEELQKHEEVLPLPDEDV

VRNYEVCVFTKWEFSNARNRFKKDRLVRQFEPVLKREEIDEIIGRCADKN

EQGSPVDVWVYRFQQCVSRSEIP

>7529760-1

TNTAVDSSQDVMKSMTLTFTKGLDACKKEMDLPDTIDVDFNNFWKEDYVV

TNRNAGCAIMCLASKVDLVDSMGILIHGSSHEFAKQHGADDNMAKQLSDT

LHSCEKTIGTLNDECLRALNVANCFKVEIH

>77166572-1

LLLLLAAARAWDVNMRLTGRIMDAAKEVDHTCRSSTGVPRDMLHRYAEGQ

TVDDDDFKCYLKCIMVEFNSLSDDGVFVLEEELENVPPEIKEEGHRVVHS

CKHINHDEACETAYQIHQCYKQSDP

>81170832-1

CFVGAFSESLSNEEAEKLMEYKESCTAETGVDEAVLMQPYDDKEELVQDE

KLNCYFACILKKMDMMDSDGTINMETARSQLLRDLCPKKIDESVECLSQV

GDSPCNTAGKIFGCIMKIRT

>87248601-1

VFNCGADNVHLTETQKEKAKQYTSECVKESGVSTEVINAAKTGQYSEDKA

FKKFVLCFFNKSAILNSDGTLNMDVALAKLPPGVNKSEAQSVLEQCKDKT

GQDAADKAFEIFQCYYKGTK

>90194174-1

FTLCIVSYMMVRCDDITLCLKQENLNLDDIDSLLEDESERMLRKRGCIEA

CLFHRLALMNDNVFDVSKFDVYLNDTDMDMDLKDSIRKIIRQCVDNAKNE

DKCLTAQKFSRCVIDYVK

>90194176-1

SVAVIIRANGINEILKIMAVSMKDIRYCIIHMGLTFKDFIKMQELLQEED

ISEGNIKKYLTNYSCFITCALEKSHIIQNDEIQLDKLVEMANRKNISIDV

KMLSECINANKSTDKCENGLNFIICFSKL

>90194178-1

TLIFLYFGEADIKKDCRKESKVSWAALKKMKAGDMEQDDQNLKCYLKCFM

TKHGILDKNAEVDVQKALRHLPRSMQDSTKKLFNKCKSIQNEDPCEKAYQ

LVKCYVEFHP

>90194180-1

SVHCGTRPSFVSDEMIATAASVVNACQTQTGVATVDIEAVRNGQWPETRQ

LKCYMYCLWEQFGLVDDKRELSLNGMLTFFQRIPAYRAEVQKAISECKGI

AKGDNCEYAYRFNKCYAELSP

>90194182-1

IALVYGEISDIDEFREMTSKYRKKCIGETKTTIEDVEATEYGEFPEDEKL

KCYFNCVLEKFNVMDKKNGKIRYNLLKKVIPEAFKEIGVEMIDSCSNVDS

SDKCEKSFMFMKCMYEVNP

>90194186-1

FAFCLVGILAVSEESINKLRKIESVCAEENGIDLKKADDVKKGIFDKNDE

KLACYVDCMLKKVGFVNADTTFNEEKFRERTTKLDSEQVNRLVNNCKDIT

ESNSCKKSSKLLQCFIDNNL

>90194188-1

FGFCVCVGALTIEELKTRLHTEQSVCKTETGIDQQKANDVIEGNIDVEDK

KVQLYCECILKNFNILDKNNVFKPQGIKAVMELLIDENSVKQLVSDCSTI

SEENPHLKASKLVQCVSKYKT

>90194190-1

SAICICVGALSIKDFQNAIRMGQSICMAKTGINKQIINDVNDGKINIEDE

NVQLYIECAMKKFSFVDKDGNFNEHVSREIAKIFLNENEINQLITECSAI

SDTNVHLKITKIFQCITKFKT

>90194192-1

SAICVCVSAMTLDELKSGLHTVQSVCMKEIGTAQQIIDDINEGKINMDDE

NVLLFIECTMKKFNVVDENANFNEKISSDIVRAVLNDNEADQLLAECSPI

SDPNALIKISKILECFFKYKT

>90194194-1

FAICVCVGAMTHEELKTGIQTLQPICVGETGTSQKIIDEVYNGNVNVEDE

NVQSYVECMMKKFNVVDENGNFNEKNTRDIVQAVLDDNETDQLIVECSPI

SDANVHIKISKIFQCFMKYKT

>90194196-1

SAICVCVGALTLEEFQIGLRAVVPICRIETSIDQQKEDDFRDGNIDVEDE

KVQLFSECLIKKFNGYDDGGNFNEVVIREIAEIFLDENGVNKLITECSAI

SDADLAVKSAKLLKCIGKYKT

>90194198-1

FAFCICVNAMTIEELKIQLRDVQEICKAESGIDQQTVDDINEVNFDVEDE

KPQRYNECILKQFNIVDESGNFKENIVQELTSIYLDENVIKKLVAECSVI

SDANIYIRFNKLVKCFGKYKT

>91983605-1

VLLIAILASALAADNDDPFESTRFKCQKEYGFSDEELLAGEENLEPMKCF

LFCFLKDLQIADDTGNFDPAVATMMLDDDIRDTAKSAIYKCHADYLTVSE

PCQHSYEVVKCFKETLP

>91983603-1

IYGVTALSEADVAELMKYQDACIAESGVDPVLIENAKKGDVAPDENLACF

ASCMLQKLGMMNDQGVLNLDNIRAKIPDNVDKAKAEEVINKCKDVPGNHH

CLKAGNFVQCFMQHKE

>91083403-1

CLVEIFAIEMDDDMKELINNLHNTCTGETGATDDQIENARKGNFAEDDSF

KCYFKCVFDQMGCMTDDGKVDSEAVIAVMPPELADKIASTVRGCTEVGAN

PCETAWLANKCYQKSNP

>91083401-1

CQGISEEMQELVNQLHSTCVAETGVSEDLINKVNSDKVMIDDEKLKCYIK

CLLTETGCISDDGVVDVEATIALLPEDMKAKTTPVIRSCGAKMGANPCES

AWLTHKCYLETSP

>108884755-1

LSLHTSESKSTMEQLAKASEMMRGVCVGKTKAPLDLIDGLGRGEFVENKD

LKCYANCVLEMMQAMRKGKVNADSAIKQVDLLIPPEIGEPTKKAFDMCRN

SADGIKNNCEAAWALVKCLHQKNP

>108884754-1

MEKTGKLFRQVCQPKHKLSDDILEAGKNGVFPDTKNFKCYISCLLDMMQV

TKRGKISYEKSLKQIDQLLPDDMKPDFRKGLEACKDVASGIKDQCDSAFV

LLNCFYENNP

>66541157-1

IYVEGFYNISGDLSQKFQLHGYGPFKGNIHDFQLYINTILGYSRGVYLKT

FDLDFTLKFIDINLKNLMDDQELNDIMNKVVQELTPKVLDIIKPDILPDI

ENYLSAKINETIHHLTMKDIIN

>73992790-1

VYLKGKYKASGKLLILPITGDGDTTIKLKNLRIQMIYPFNLVKNSEGKDV

IDLSSYRYSYDVKDNAHFHMTNLFNGNKQLGDVMLTFMNQNWKALTQEFG

KPLLEIPMEMVYNTVKTYLKSQPLEDIAN

>73992792-1

LDVNNTYDLSGRILVIPIKSSGDSFIKLKNTLLNINFYYEDIEDADGKVH

WKIFNHDVEYEVEKAVFRLENLLNDKNLGEQINKILNGLSQQIVDEVGPT

ICGSLMKAVVENLGILLEQVSFDELMP

>73992794-1

VTLTGDYKLSGKLLVLPIEGEGKYNIKIRDIVIKTANDLVTVTGADGKPH

WHIESWKHTYEVKTGAHFQFENLFNGNKVLATPVEEFVNSNWKDVMQEVA

PPIVRSIVSEVVAAVDALYKAVPAEELYL

>73992796-1

SIKGHYTAGGSILVLPITGDGQMKLKLKNMQIHFYIEYDVEKAEDGKDHI

VLKKYDFDFDVRENAHFELSNLFNGNKELSKIIHSFLNENWKQVVTEFGR

PIMDATAKKFFKNINIFFEKNSLEDIAI

>73992798-1

IVIRGDFKGEGQYNALKVKAYGDFNTSMSDLIFTWKLDGVPEKNGTDTYV

RIKSFYMRPDVGNMISHLNNENPETRELTNLGTSFLNQNWRVLYRELLPY

AQSNWDKIGTNVANKIFSKVPYDQIFP

>73992800-1

ISARARYRSSGVLLLVRASGGGEYWGEYHGVKAKVYFRGAPYERDGRTYL

KLQQLKLDFSVKDIKMGVENLDNSNAVLQAALNLFISTNAQELLKEMKPE

LKRDLADKMSRYLDHILQHIPYDELVV

>73992802-1

MSLTAEYEADGKLLILQIKGKGDALINCVNVDVEIESKLNQVKDNNGKDH

LKLGTPSYKYKIEKTTFDLKNLFNGNKELADTTLQFANENWQQLMDDLAP

PAIKQIVKTVVKAINKFFASVTTQQIIK

>73992804-1

IDIEGKYKVFSESPLIKNLLGGTTVHGEGNGKVQLEKLQISMKFPVYAQK

RDDGEIYMKCDYSKIKYDYKILGKTKFYADNLFLGEQEASKLVTTFLNEN

WKFVMDTFGRSFFDKAMDIFYSYTSKFFGTVPAKHYLT

>73992806-1

VTLVGEYTLGGQLLILPIEGTGKYRIRIRDIIMKIILDVEERIVEGDRYW

HVSDWKHSAEDVSKVEYQFQNLFNGNRDLAKTIHDFANSNWREIFQEVAP

PMVKAIVSKIIHETFKLFDKVPIKDLAL

>78707282-1

NYTGKYSLHLNLLLPDIKGKGNMQGYCENAKAFVKIRGSRYLRNGKDYVK

FSKMTTLIDFKDFKLKLANLFSGDRFLGDVGNSLINNNQELYLKDIAPSL

EHGLSKHFLDVADKILASATFDEMFP

>19921300-1

KYDTDTLIDALRQLGLSVEYEGSGELLFDLVNLRIAGTLKYKLPMLWGSA

KITSLKTTISLESVTSDITGFMGNGKINRAINSQLENIVVKGINGNQDAI

SETIENAIVPRVNKMLKGKDFWT

>39939980-1

KHSGEIKVAGVQMDLRDVSYYVKRKKGFPSLTDVGLMDVFMGGNGFSFKI

KVSTPDKKDAQNFFKVDKVDVEVNNLSIKIHQSNHKFLFSIAKPIMLKVM

RPAIEKALGKVIKDRFNEFDQILF

>61819907-1

TGDWHSILMAADNIQKIEEGGPLRAYIRQLECTDRCSSLSVNFYAKFPSQ

CTFLNVVAEREGDVYHVGYMGSNFFELIPVSENTLAVYGENFDGVKSTKV

TQLLAKGDHATQEDIQQYEELNRERGIPIEHIEDLTQTD

>61820389-1

AGEWYSILLASDNREKIEENGSMRVFVEYIDVLENSSLLFKFHTKVNGVC

TELSLVSDSTGEDGVFTISYDGENKFRILQVNYSQHIIFYLENFSDSFKL

LELYAREPDTSPELKSEFVEICQKYGVVKENVIDLTKVD

>62297956-1

SGVWYSIFMASDDLNRIKENGDLRVFVRNIEHLKNGSLIFDFEYMVQGEC

VAVVVVCEKTEKNGEYSINYEGQNTVAVSETDYRLFITFHLQNFRNGTET

HTLALYGTSALEPSFLSRFEETCEKYGLGSQNIIDLTNKD

>62644883-1

SGKWFNKAMVSEDEFPISKVSPIFILVLNNGDLEFSVTFMMYGQCFEVTI

ILEKTDVPGQYTAWTPTFEGKTHLKVQVQPSSVKGHYMLYCEGDVEEMSF

TMTQLMGKDPQEDLEALEEFKEFTQLKRLVPENLLIPEQME

>63746579-1

EGPWKTVAIAADRVDKIERGGKLRIYCRSLTCEKECKEMKVTFYVLENGQ

CSLTTITGYLQEDGKTYKTQYQGDNHYELVKETPENLVFYSENVDRADRK

TKLIFVLGNKPLTSEENERLVKYAVSSHIPPENIRHVLGTD

>73967584-1

SGVWYSVSMASDDMKRIEKDGDLRVFIQNIESLEDGSLKFNFQFMVLGEC

VKVAVVCEKTDRNGEYTVNYEGDNRVLLSETDYKLYITFHLRNMRNGTET

NVLALYVCRKYGLGPQNILSLSDQM

>73971966-1

SGDWYSILLASDIKEKIEENGSMRVFVKDIEVLSNSSLIFTMHTKVNGKC

TKISLICNKTEKDGEYDVVHDGYNLFRIIETAYEDYIIFHLNNVNQEQEF

QLMELYGRKPDVSPKVKEKFVRYCQGMEIPKENILDLTQVD

>76631142-1

AGTWYVKAMVTDENLPKEMRPRKVSPVTVTALGGGDLELTFTFLQEARCH

EKRSRMQPTGEPGKYSSNGGKKQVHILELPVEGHYILYCEGQRQGKSFHV

GKLIGRNPDVNPEALEAFKKFVQRKGFSLEDVFTPEQTE

>76666513-1

TGRWLTHFIAAENIDKITEGAPFHIFMRYIEFDEENGTIHFHFYIKKNGE

CIEKYVSGLKEENFYAVDYSGHNEFQVISGDKNTLITHNLNVDEDGRETE

MVGLFGLSDVVDPNRIEEFKNVVREKGIPEENIRNFIYND

>76677407-1

MIRGEYQRHRRLVHHLHTADNKERVVEGGPLRCYCYQTECNNDCTYLSPT

FYVKIDGRCRLSTQVLKRQE AGIYLIEYADTNVLQLIHVPDNMLVIYFE

NDDGQKITKITIGTAKGDNFTQEELQKYQEPNNERGIPNEN IENVIEAD

SCPP

>76686423-1

VGEWRTIYAAADNKEKIVEGGPLRCYNRHIECINNCEQLSLSFYIKFDGT

CQFFSGVLQRQEGGVYFIEFEGKIYLQIIHVTDNILVFYYENDDGEKITK

VTEGSAKGTSFTPEEFQKYQQLNNERGIPNENIENIIETD

>76688940-1

TGNWYTIYMAANNKEKIEEGGPLRTYFRQFECIDNCEKMSITFIVTHYDS

CTLLTVVAQRAEGHVYHVDFMGKNSVQLIPVSESMLVFYAENFDGEKTTK

LTYALGKGDSLSQEDIQKYEEINNERGIPNENTEDGAQT

>82799960-1

TGPWHTLKLASTDRSLIEEGGAYRCFMTDIVLLDNGNLNVTYFHRKDGKC

VKEFYIAEKTDTPGQYTFEYQGRNSLTFVHVTEDFAIMDLENQSEGGTTI

VIEFHGRSLSTDELGWERYLVHTRRRGIAPENIVDLSLSR

>94407587-1

DGPWKTIAIAADNVDKIEISGEDKIEISGELRLYFHQITCEKECKKMNVT

FYVNENGQCSLTTITGYLQDDGNTYRSQFQGDNHYATVRTTPENIVFYSE

NVDRAGRKTKLVYVVGSGHPLTLEQKEKIVEYAKENNIPPGNIREVLTTG

>94420123-1

MLLEAAEGEQRISEPCPIFSTDDGFNTFTLLKTEYDNYIMFHLINEMNGE

TFQLMLLYDLELDLSSDIKEEFEKLCEEHGIVRENSIDLSNAN

>77415628-1

YMKRFDKLNVEQVLNNDRVLASHLKCFLNEGPCVQQSRDLKRVIPVIANN

GCNGCTERQMTTIKKSLNFLRTKKPTEWARLVKIYDPSGTKLNKF

>77415630-1

YLHKYDNVNIDEILNNDRLVASYFKCLMETGKCTPEGEEITRWLPEAVEN

KCENCSEKQKMGSEKIIKFLFEKKNDMWKQLEAKYDPQGTYRQRY

>77415642-1

YTTKYDNVDLDEIIKSDRLMKNYVSCLLEKGNCSPDGSELKKHLPDALHT

DCSKCSDTQKNGSKKIIRHLIDNKKDWWKELEAKYDPEGTYRKKY

>77415644-1

YPTKYDNVDIDAILHNKRLFDNYLQCLLKKGKCNEEAAILRDVIPDALIT

GCRKCNDHQKVSVEKVIRFLIKERNSDWQQLISVYDPKGEYQTQY

>77415654-1

INLDEILQSNRLLXNYVNCLLDKGSCTAEGKELKKVLPDALSNECAKCSE

KQREGAEKVIRFFVNNKPEEWKKLSAVYDPTG

>77415666-1

YTTKYDNINYKEILENKPLLHNYIKCTLDKGRCTAEGNELKSKIKDALQT

GCIKCSDKQKQGARDVIQHLEKHEPEYFAELRAKYDPNNEFESTM

>77415668-1

YTTEYDGFDIREVMRNERLLTSYVNCLLDKGPCTAEGKELKKNLPDAAQN

DCKKCTHRQKENADLMIQYMEENRPADWNKLELKYDANETYGTIL

>77415670-1

DEKYETINEDFDVAQVLENERLLNSYAKCLLNKGPCTPEVKKVKDKLPEA

LETHCAKCTDRQKAMGKQLAQEVQKRYPDLWKELVALYDPEGKYQDAF

>77415672-1

YSSKYDDFDVQPLLENDRILLSYTKCFLDEGPCTPDAKDFKKVIPEALET

SCGKCTPKQKKLIKQVIRAVIDRHPESWDKLVHKYDEDNKYKDSF

>77415676-1

YTEENDDLDIEGVIKDADTMKAFTGCFMDTADCDHVSGDFKKDLPEAIQT

ACAKCTDKQKHITKRYFEGLEEKYPELYQAFKNKYDPENKYFAAL

>77415678-1

YTSKYDGVNVDEILANDRLMMPYIKCALDHGRCSPEAKELKSHIKEALEN

NCAKCTDKQKPAVRKVIAHLINHKPAEWRQLSDKYDPAGKYTAQY

>77415680-1

YDAKYDSFNAHELVQNQRLLKSYGKCFLSKGPCTAEGSDFKRVIPEALKT

TCGKCTRKQRELVRVVVKGFQEQLPQVWTEIVSKEDPKGEYKDSF

>77415690-1

YSTKYDNIDLDSILKNDRLLQNYVNCLLDKGTCTPEGTDLKKVLPDALEN

ACAKCSEAQKRGAEKKLSDTFLKTKRM

>77415692-1

YADTYDHIEPMEILNNDELRNQYYNCVMNTGPCMSDEQRFLKEHVAEAMA

TRCRRCTERQKDGLEKVVVWYTENRPEEWSALVVH

>77415694-1

NIDVDSLLKNKKYVQTQIKCILNEGKCDKTGRDMKDLLPEVLQRNCRKCS

EVQKVNADKIINYMKQNHPS

>77415700-1

YVTKYDNIDLEEIFSSKRLMDNYMNCLKNVGPCTPDGRELKDNLPDALMS

DCVKCSEKQRIGSDKVIKFIVANRPDDFAILEQLYDPTGEYRRKY

>77415704-1

YSTRYDNLDIDTILASNRLVTNYVDCLLSRKPCPPEGKDLKRILPEALRT

KCARCSPIQKENALKIITRLYYDYPDQYRALRERWDPSGEYHRRF

>91983607-1

YTGKWNDLNTHDIVDNARLFKKYKQCIMAETNTGCPQEVIELKKVLPEAL

ETVCSKCSPVQVEKIRDTLKYVCEKRKTDFDDILKHIDPEGTHRPKF

>13236828-1

DYDALFANDEQRKIVFDCLLDKGDCGAYKQLADLSMKLIINNCAECSPTQ

KTKYEHVLKQLHDNYEPVYNDILKK

>25013078-1

DERNINKLLNNQVVVSRQIMCILGKSECDQLGLQLKAALPEVITRKCRNC

SPQQAQKAQKLTTFLQTRYPDVWAMLLRKYD

>52000993-1

NIARPEIRKFSNVLIKYNVVDKSLKADIRKIMHECAKKVKKQAREDSHWL

NCRTTINYY

>56435218-1

YPSELDDLDVVALLADAAWRQQSDDCFLNKGPCSEEQKYLNDLFREAVRT

DCERCTDKQRQIMNTITEWYEQNEADVWKIILE

>56462368-1

DQVDPTPILKNKELTNKAFQCLMDKAPCGEFKLWRDMVPEVFKTKCSDCT

PSQKDKFNLYVEVLKTSHPDIYHALLSKY

>6560637-1

DLYDMFDAEMILEDDKLRSKAIDCLLDRGVCDDYQPIRDKGPRLIKTRCE

DCTPEQKAVFEESMKILEEKFNNDFKEIIAKY

>77415552-1

LSDQRYLRRQLKCALGEAPCDPVGRRLKSLAPLVLRGACPQCSPEETRQI

KKVLSHIQRTYPKEWSKIVQQY

>77415578-1

YSSENDDLDIEALVGNIDSLKAFIGCFLETSPCDAVSGDFKKDIPEAVAE

ACGKCTPAQKHLFKRFLEVVKDKLPQEYEAFKTKYDPQGKH

>108884753-1

VHLGGVMSSMTFEDMQETAKMMRGICQPKYGIPDDVAENASSGVFPDSRE

FKCYASCLMDLTHTAKRGKLNYEAAVKQITMLPDDFREPFRVGLDSCRNA

ADGIDDYCEVAYTLLKCFFKASP

>108884739-1

VTELQCANSDEEKKAQAKEMMRGMAEECKKKEGATDEDVEALLEDKTPET

EVQKCFLSCFQHQFQISDGKRFNKDGFMQLSAMMFGEDQEKMATAEEIAE

ECSSVENADRCQLSVDIKECVEKAMD

>108882837-1

SLLAVGSQAFFTPEQHEVAKRLTMACATEIGEGLPDNVGNRFREGDLTLT

DDKSKCFMKCVFGKVGFIDDAGTVNKEVLVEKLSKGNTQAKAEMFAEKCN

MFEGANGCEKAHGLFECYWKNKE

>108882836-1

LTVVALCKADYSDKQKQKLDEFTSKCIEDLDLPKDSDLGKKFKYGQLKEK

DDATKKFISCSMQKLSFMNETGSILEESIIEFLADKYDRTMAMNVITKCS

KLKNESMEDKAAEFYDCFFMQKS

>108881986-1

GVALLMLASSIVPGCAQDFKGAIDECTKEFEMDMDVVVSLKYGDFSERDP

LIECFTECLMKRSGFMFDDFSYNKTLIIGFAGRYLEPEGAQYVYDNCVDK

FGTTVCVTGFEMYQCIHETAV

>108881985-1

MDTFNAIRNGDFSIRTPFIECFGDCLVKKAGFMNDDLSFNKDVIVKFASR

FIKPEDAETVYSQCTADVAPVLCATAYDVYQCIYENAL

>108881983-1

SFALIVGCMAVTEEQKEAARQLAGKCMQQTGTSEESVQRLRNGDTSGADD

NTKCFVQCFFQGAGVVDGEGNMQEAFVTEKLASEYGQAKAEEVVQRCRNN

SGANACERSFSLLQCYIANRA

>108881981-1

LVAALIGVNALTEEQLKKADEFASECLEKSNGLSKETVGKLRSGDFANVD

QDSKCFVKCFLERAGFMSTDGNLVADYAIERLSLDREKSKVEALVMKCSM

QMDDPCETAFRAFECYYNGKA

>108881980-1

FALVGAALSVPQQANLEDIGKIRNGETYALECLLASGLDVSSLKSLQTGD

FSNGDRVKCLVKCFFEKTGFMDAEGNLNEEAIVTQLSQFMPKDQVETLVK

NCKIEGTDACDTAYQATECYFKNKA

>108881978-1

LLIAIVAVNAWPSYKRAEVRAHVRNCVKKTGIPGKNALKVLKGNFNDDSS

EVKKFMKCMFQEVGFINEKDELLDNLLIAKIKENLEEDEADELIEKCSIV

GDDINDTAFQIYKCYYENHD

>108881977-1

VTIALNQIKAFTLQQRQQGDIYAIECIAETGVNPASVALLRVGDFSSNDK

RSKCFIRCFFEKEGFMDSKGNLHTEKIADALAGDFNREKVETVLANCLTK

EKTACETAFRMYECFYNHRE

>108880056-1

AVLLLIFSVLVHSQSIKDLVEECKQTVPISEELEKSFLKLEFPPEEKTTH

CLLDCIGKSLKVMDEKSGINLAVVTKLLQEVEPEGVIGEEQVRCATEAAT

SKEDQCTMAFKLYQCFEKEFL

>108878499-1

TSLIAAASAGWRLQTVDDLLRNRNKCVKILNMQDDLQEEFGLFDFPDQDS

AKCVFKCIMNRMGLFSDKRGPHVGRLVKQMKFASMSSTKAIRDEILNCAY

QDMEMDPEDVCDRAYALYQCIQNSNL

>108877717-1

EILSTSDAAMTMKQLKNSLEMMRKACAPKFNVVEASLDELKAGRFANEAD

KELKCYTMCIAQMAGTLTKKGELSLSKTTAQIEAMLPQEIKAAAKEALNA

CKDIQSGFKDPCEKVYFSAKCAAEYNP

>108876458-1

GPISQCASQNSNVAKQIDDYRKQCVELSDVSVDSAIKVHSGQVIENPDWS

TKRYVQCFFQKMQFMDENGVMLKDAVVEFFSRIQDESRAKAMVENCDIQK

ENPLDTAYAVLVCYQGNKN

>108876018-1

SMGIEMTASQHGECVTETGVSEESIARFNGPEIFEDDDKLKCYMDCMFRK

FGATKPDGEVDMIEVYHKIPKDFNSVALIVNNKCRDAIQGANQCERAFSH

HKCWKQMAP

>108871958-1

LVIIGVFHTIPADAGQLLNKLITVCTQGQNPPADLVQRYRNGEFPNDRNT

HCMMRCIALNLGVYDDLNGIHMHDTWQMFRRGRPASHEKAFAEQHRQCIT

QQTKDVPLDDYCGRVYAVYQCYKDEYE

>108871227-1

LGGVGVEGKATVEQMTKTGEMIRNVCIGKLKVAEDLVNMLGDKQFPDNKE

LKCYVNCIFEMMQVVKKGKLNYDAAMKQIDTIMPDELAEPMRIALNACRT

ASDGIKNNCDASYAIAQCVAKNNP

>108869414-1

ETLRPSDAAMTMKQIKESMETMRKACAPKFDVPETTLNDLKAGNFRPDAS

KDEKCYAKCIAQMAGTLTKKGEISFSKTTAQIEALLPTELKAPAKEALKA

CKEVHTDYKDSCDKVYYSVKCAADFNR

>108867388-1

KPIPRRDAEYPPPFVLEISKKPHKMCVASTGVSEAAIKRFSDEDIFEDDE

KLKCYMQCLFEKLRYTDDKGELHLGKVMDSVPEEYEDIALKMGSKCLKPK

GKTQCERAFWYHKCWKTSDP

>108866736-1

WDCCNMPNLVNQDIRADCHQKYGEQTMKQMKLEGTPRGCCIAECQLNATG

LYADGMIKRDEMTTMFMDSVKDTPEWQPMVRDLLDECFREAEANKDIIA

>108872189-1

CIEGPPVNKSPSECCTTPALIDPPLMMKCFQKWGEQTKRQSKMDGIPRGC

CVADCAMEGTKLISKGKFNREKARKVFMAVVKDQPQWQPIVNETLDACFK

QADENMAEIE

>108872191-1

NVDPFECCKTPKLLDEGTVKECVHSFPPPQNAQDEIKPDCMSECVMNSTR

IFDRRQNVNDAKAMETFLEKLNGKSVWAEIVQKAVKQCLDDADNRKEEFS

RDMKALQQKFP

>108872193-1

KDCCKAPNVIPPKDQFAECMQKYPKPSEPPTPGSMPPNHNCLAQCMFEQQ

GIMADGAVSKDAAISKTVAVMGGSSEWEATTKNVVEACFQKVSALGAQKD

SQGCSVMAGSFMDCMP

>108872194-1

DANGCCELPRFVAREINAKCDEEFKPLSPRLPPEVQAYEGSCVIECLFNV

TGMFKDGKLQQDKIAQQLKKTIGADRNFAPLLGGVVTDCYRLVMDNPANS

FK

>108872200-1

CLLPKFYDSQMVSDCLTSISKSTNDVEKYQCLVECIAKKLNLFKGNTLDR

EATMQLYKARIGSVPHFAPIMDNIFQQCYDGMAVYAAQDRSDPTKCSAL

>108872201-1

SFAVTTHNSTWDKSCFELKTSKRADDCCDIPGSFDEALLKRCYDEQKASK

NEQEAIKCIAECVARELGAYKNHTLIRENSRLVFESTIGSDPNFRPVLGD

VFEKCFNRITAIEAQETYKNATCHFAP

>108872202-1

SILKLSCNDGPVDKSCFELRTTKRADDCCKIPDILVESDESMVRRCFAQQ

NKTLDEHETAKCAAECIARELGTFKNGALDKELAKKVLLGRLDKDKNFKP

IVGGVLDKCLGRINAVIEKESKRNGTCNATANFLFDCAEQGLF

>14423995-1

LLVFTQIIYAVDIKELKIMNRILEKCIRTVPKGENDPINPLKNVNVLYCA

FSKRGIFTPKGVNTKQYINYCEKTIINPADIKQCKKLISKCIKKVYDRPG

PIIERSKNLLSCVLKKGV

>15963505-1

AVFFIGICQALNPSRKCRLDYKDKVISESCILHCEYKAYGFANDKYDIKR

KQIDQFVDVLINGKAVASDKRQKLENLLRGCANKARGKNPKLGCHTSIDY

YRCIVADQK

>15963507-1

VIIPLLYAEIAFGFEHPEAFCIKKHKDTDFECILHCKFKYYNFVDDKYNI

KDYHIRNLADFLINYNVVPANKRRNVEAHLRSCVAKSIKKHRTPSCDSIF

SYYTCITDEKL

>15963509-1

AVFLIGTCQAENPSKKCEEKFKNDASKMACIPHCKYQYYGFVAMDNNIAK

PEIRTFSNVLIKYNVVDKSLKADIRKIMHECAKKVKKQAREDSHWLNCRT

TINYYRCILTDKR

>15963511-1

SNKWYAQNPDVKPKGTKISKFCKAKNREQGESNCKHACSAYYYRLVDEDF

EPIHFRLLEIKGFSNEDIDECTKQTSGGQGCQRSDALYDCLKNKKS

>15963513-1

SILGLGYSWRFPRNGDQTYWAFNTCQRQTTDIESVKLWDQWLLPNNAATH

CYIKCVFIHLGFYNEQEKAINIDAVKKQFKSRGLEIPKDIKSLSGRTDGS

CKALYEKTIPFFKNNF

>16225983-1

SVRDAIYKKNPQIKPKGISIFRFCGKQFYQDGEAAYCNVRKHGFSDDPKF

IKHSNCTTRGMRWMKKNGEMDESAILRGLHAVNENGKDDVVKKSLQNCKA

KDESKARDYYKCIYDGLG

>16225986-1

VAFFAGGNAAWKPFSPEETLFTYTRCMEDNAKGDLALAKKWMAWKLEADQ

KSACYAKCVLVGLELFDESSKTFKGDHILEQYQKYKSYTSQDEAGVKKFQ

QAVQALGTIDSADCLKVLQKYGPVHAQFT

>16225992-1

SVFMHCEALNYPKGSPQRKDLCEIRKYQMGSGIVFGRHMECIFKGLRYMT

SKNELDVDEIARDFIEVKKKPDALKAMMKTCKANLKEKNAGKIAVHYYKC

LMNDSK

>16225999-1

SLLCLCGLGYSWQDVRNADQTLWAYRSCQKNPEDKDHVPQWRKFELPDDE

KTHCYVKCVWTRLGAYNENENVFKIDVITKQFNERGLEVPAGLDQELGGS

TDGTCKAVYDKSMKFFKSHF

>18140727-1

WMMLLATAADPDCENLKNRREEMEQCCQVNMIIPLDGAEDCSSSVDETSE

PHDKMMCTLECKLKSLGLLNGDHLVEAKVQEYIDRLEGDWKGTAKTIATE

CITTITEMKKKIQERDH

>18140733-1

VEKPHECCKVEPFFEASDFTECGYKNSNEDVGFKRGPPDCSKQLCLMKKY

NLAKDDQVDFEALKKFLDDYAEKYPAFKSAVEKAKECVKEDLPGPPSVCL

ANRIVFCIG

>18378603-1

VVIALICPLIIVETLAVSDCVRHVSESARNTVCDVRQYRVTKGVEADRYV

QCFMTALGFADESGSIQRSNVLTALDAVETHDGVYTDAVDVCLSKAKKLP

GTERSGYFFSCMLRTES

>21630076-1

LLLIVVSLSQAKESQPFDFFEGTYDDFIDCLRINNITIEEYEKFDDTDNL

DNVLKENVELKHKCNIKCQLEREPTKWLNARGEVDLKSMKATSETAVSIS

KCMEKAPQETCAYVYKLVICAFKSGH

>24640769-1

PAIPVPMRSSPQSLALLRARDQCGRELTAAQRLQLDRMQFEDAAHVRHYL

HCFWSRLQLWLDETGFQAQRIVQSFGGERRLNVEQALPAINGCNAKTSSR

GSGAQTVVDWCFRAFVCVLATPV

>24643507-1

PIWAVIDRNLPQVQELVTAARMECIQKLQLPRDQRPLGKVTNPSEKEKCL

VECVLKKIKLVSSRTFVTTRTKRSLILLIRTQMDADNKLNVGQVEKLTSL

VTQDNKMAIAVSSSMAQACSRGISSKNPCEVAHLFNQCISRQ

>24644507-1

VSLFLICSQALADLSGDAQTLEKCLRQLSSPESIAGDLRKLERYSSWTRE

EVPCLMRCLAREKGWFDVEENKWRLKQLTEDLGADVYNYCRFELRRMGSD

GCSFAYRGLRCLKQAEMH

>24652686-1

PALCCKDGGRDQVAEQCAQRILGTANGQKAGGPPSLDTAACLAECILTSS

KYIDEPQKLNLANIRSDLSAKFSNDTLYVETMTMAFSKCEPQSQRRLAMI

MQQQQQVQQQKT

>24658429-1

LVAVRVHCRHMERIHEENIHHCCKHQDGHDDVTESCAKQTNFRLPSPNEE

AIVDVTVDQAMVGTCWAKCVFDHYNLMENNTLDMDKVRSYYKRYHQTDPE

YATEMLNAYEKCHTQSEEATEKFL

>24658441-1

TTAVAISSGDLTEDKCNTSRAGCCSELYIGEEEDLVKCFVIHSPKLPVDG

DADIGKTLRFLSCFVECLYKQKKYIGKSDTINMKMVKLDAEKTFVDRPKE

KDYHIAMFEFCRKDAVGVYNLLKASPGAKVLLKGACRP

>2735108-1

YLKTYLSWLTEEQKEKLKEMKEAGQTKAEIQHEVMHYYDQLHGEEKQQAT

EKLKVGCKMLLKGIIGEEKVVELRNVKEAGADIQELQQKVEKMLSEVTDE

KQKEKVHEYGPACKKIFG

>27372915-1

AHLMVEASPSWKALDPEQTLNAFKHCLDHHLPSGSDRETYKSLWLAWKME

PNDSITHCYLRCALTGLQIYDPQENAFKSERIPVQYHAYKNVSRAQQNEV

TEYQQALAAANPKTGSCVDVYNA

>27414065-1

TGEGPSADDVKRIVRTCMNKITNAGGGNFSSSSSSSTIERDRACLMQCFF

EEMKATNADGFPEKHKVLHVITKDIREHELREFYVDSIQECFHMLGLDNR

LKDKCDYSMRFVTCLSDRFE

>27414073-1

SPAFRPASFLEVMEVVLDCFNTLRIPLQRFPSYLSGIFPEDPETKCFLRC

VAIKLGVYCDEKGADLDRHCVQFGLGECCENFSNRHLVCLQQNSLPCPDR

CTAAYKQELCFQEPIA

>27414085-1

CALILGAPATGHGYDTKSFAQAYLECLRYLNISRQSLYAYDSAAVPLNCG

SNCLLRCIGLNARWWHDETGLSERALVRFFRQAPADSLLQARACVAELPA

PPADSCAGAYWSFRCYSDA

>27414099-1

ANIFGGKLYQKAQQDCILFMGINPLRLDQYKKFVYPPDRDTMCLIRCIGI

SLDFWDDILGFDVDLAEQEFSPLVDATFKKYLAGNITLKLELLDPLDNCA

RAYYAFRTFRAQIL

>28573615-1

GYGMDHDQEEQDRNPGNRGGYGNRRQRGLRQSDGRNHTSNDGGQCVAQCF

FEEMNMVDGNGMPDRRKVSYLLTKDLRDRELRNFFTDTVQQCFRYLESNG

RGRHHKCSAARELVKCMSEYAK

>30175921-1

SIKVASCCQLEAFLTLPTYGNCLQTIAEKYPDALWQGTVCAFDCTYREMG

ILTGVDDINVEQISTNQAGYDQAYQEAIAKAVTACMAQKDKIREEADVVQ

SECSMFAVKFHACVSLETMR

>30178721-1

LVLCGSLLVTGAPNTCGKLDLKTDPFTCCTIPKLLDVTICMSECILNSTG

IYNRRGDVDEKKLNSVFTDSLPANSPWLNVVRKAIKECTAKADKKDKEFQ

KDVADQKK

>31747525-1

CPGDGRHDVGRQVDIEAVMALLPPEMAEHNGPALKSCGTQRGADDCDTAW

KTQVCWQNANK

>33355867-1

FFSKHPKQFPPSKKQSELPYCCQTEPLIPEHVSTKCKEREAANHNPGTEL

FEVCYQQCIYEELEAVDGLEIRVEKLYALAEGFPADYRHAVHLAIDECVK

RLRKTRHMFEQMN

>33355869-1

CTIPKLLDVTIVSSCFEKFPIDKDAADKGAASMPKTEVTDCMSECILNST

GIYNRRGDVDEKKLNSVFTDSLPANSPWLNVVRKAIKECTAKADKKDKEF

QKDVADQKK

>33355875-1

LTLHLLPQSVADDCIDMDLHSMEVARCCRYEPISTEEVAEKCYQELAPNI

PPNSSDFPVCFIDCSYRQMGYITNEANEIDQSKYGQFLAGFDTAYKIAVE

RAVAACATVQEDIRRDVANVPSKCNAFALLFHVCVTQITL

>33355879-1

FTAFSSFLQTTKVASCCQLEEFLTLKTYGNCLNTMAEKYPNSTLDYLVCG

LDCTYREMGILTGVDDINVEQISTNQAVYGEAYQEAIGKAVDACLAQRDE

FREQEKFTKSECGMFALKFQGCIMVESMR

>33355881-1

RALWLFLKFEVPHCCQMEELIPRPSRTKCQEKAAIDHNPGFQAYFVVNCL

AQCQLEELEVIDGEELHLEKLYPLTAKFPADYRHAVRQAIDECDAWLQGK

KKERRRPDGKAHCPLIGMEVENCLHRTTF

>33355885-1

TVLWVTVIVLVKVMVKSDAQVCCMVEHTFPQEPYRVCHEQHATPQMDNGT

VMCIHQCYYKAIGMFAADGKVNTDAYIKYRDELDPTLRDAFSYSMVVCAK

IIAKRMNNNIAEVNR

>33355887-1

FGRFRRSASEVQDDKCKRKYKCCNDANTENMEKIHEIKKQCFMEMEVICA

MECVGRKKEVVNEDGTLIEPKLMEFVKSNFAADDWQQPLLAGHIETCVKE

AKEKAAKMPREAGQCSSETSN

>38350597-1

SQIFDASGADQKPYDKQHWERTNQLCSRLLRTPQEARDLISQKKFDDDAN

EAMHCVIRCTGIVSGTYDGERGTVMEMMEVQAQGKTGFAEYRSAAEDCYG

GFGPEDYGDDWCKKSYLYFKCDWK

>38350687-1

AIDLTSANGVVVSKIDEIFVGKCEKNIKTKVPAQLCQIRKLLIDVATAET

KAFGDCILKEFGYFDSNGKIDKSALAKDYSEQGFIGKDAELSSMIDDCER

EFGTGINSVNYLLCITMEKDF

>38350693-1

TLLALASIITLSTATSEQPNWGEVSSTCHKLLRVSPEVGAPHGQDHFSPD

PKSACITRCVGIITGMYDDETGISMEQLRTWWVDEDTDADFQEFKRRYLA

CAGSIVPEQYGDDYCKKSSKLYECFMQSGMT

>40019411-1

TVDECCAIPMLANKTVIEKCKAAHPFKPPQNTDDKGPRGHPGECIAECIM

KGMGALKNEKVDGPAFRKAIEPVVKANPAFAKLLDDTVKQCHESINVDSE

FTRYVTKPVCKADAKAFINCVYGTLF

>40019415-1

IGKAVDACLAQRDEFREQEKFTKSECVNIRNNFHLPKSNRNCRTAARRNH

SSRNTCRRNCYQQCIYEELEAVDGLEIRVEKLYALAEGFPADYRHAVHLA

IDECVKRLRKTRHMFEQMN

>40019421-1

ATMRCCNDGFEKSEVHAKFAEVRTACMEELGLGETTHEELIKNREHLNCI

TECIAKKEGIADENGALLHTDLAKVVLEHMSTIEWKVPLAEGFIQQCFDE

VELTDGAFVPSDEAKCNPEGFDFVFCLWRQFT

>40019425-1

CCNDANTENMEKIHEIKKQCFMEVRNKNKADGAYEPVDFFSCERLNKTKM

EVICAMECVGRKKEVVNEDGTLIEPKLMEFVKSNFAADDWQQPLLAGHIE

TCVKEAKEKAAKMPREAGQCSSETSN

>40204874-1

SFIPFTALEHRRIVRECVDILQIGPQARQAILDEGLMEVPEGRCLLRCVL

LREGLYNDWRGPRLGSLWVQTEGHEDRFFDTAQKCYPLLKMQTLEPCELA

ARFAAECLPSRVP

>40204880-1

FKAFQKQCGRYMPESGPRVSPALKNCCKMPLLELSKYTSQCGQYLVNGAH

ITPCSFECLFEAAKALNGTSLVMENIRKMMETLLESHQEFVDIYTEGFHH

CSGEEQAMIKSLKRRRMPVTGKCSSMS

>4103771-1

AAVFKAYDPVHKAHKDTSKNLFHGNKELTKGLYEKLVEEVKRDFKLVNKD

TKALEKVLNDCKSKEPSNAKEKSWHYYKCLVESSV

>41323026-1

IFSAIFFLADPALVKCSEDCENIFHDNAYLLKLDCEAGRVDPVEYDDISD

EEIYEITVDVGVSSEDQEKVAKIIRECIAQVSTQDCTKFSEIYDCYMKKK

I

>42491541-1

GFYRVEGSSQCEEDLKEEAEAFFKDCNEAKANPGEYENLTKEEMFEELKE

YGVADTDMETVYKLVEECWNELTTTDCKRFLEEAECFKKKNI

>45550427-1

TCCRTPELDMGDVPQKCHKYVSGLKSANSKYPSYAHLCYPDCIYRETGAM

VNGKIKVNRVKQYLEEHVHRRDQEIVSHIVQSFESCLSNVKGHMKSLNIE

SYKVLP

>45551098-1

FRAADPICSQRPDVTALRNCCKLPNLDFSSFNSKCSQYLVNGVHISPCSF

ECIFRAANALNGTHLVMENIEKMMKTILGSDEFVHVYLDGFRSCGNQEKV

LIKAMKRRRVPITGKCGSMA

>45552617-1

VQNVHVCCSAPLPNWGVFNRECHKSAIQASVSINRISKSKVNLANFLIKC

RLDCDFNASSVLQGNRLIQAKVRPMLERAFSNEPTIDAYESNFAKCSTVV

RSKYQELSPLSRQSDACDRHALFYSLCAYARLI

>4887114-1

IIIALFFLVQQSLAEHPEEKCIRELARTDENCILHCTYSYYGFVDKNFRI

AKKHVQKFKKILVTFDAVPKKEKKKLLEHIEACADSANADQPQTKDEKCT

KINKYYRCVVDGKI

>48994206-1

AVTAEDCCKIPKPIDNAIMEKCRAENPKPGQMPAPGVPRTEGCCIVQCAM

METGGFVNNALNTDAIKRSMASTLGADSNFGSLVNGAVDTCARQIQNDPA

YSVAP

>54635667-1

HYCCKHPDGHDEVTEMCAKQTSFRLPSTEEEAMEDVTVDQAMVGTCWGKC

VFDHYKLLDSNDTLDMIAVHTHYKKYHKTDPEYETEMLNAFHKCHSNTED

AASQFLSLP

>54635706-1

FNAVRGCCTIPTFHFKAFQKQCGRYMPESGPRVSPCLYDCIFNATGVLTG

SEVDAENARSMLKRLLGNNQDFVDVYLDGMLQCPGAVDAMLRSKR

>54636445-1

FVDPKTCCPMPDLITAELKEKCKSFDMTPPPRPTDASGSFESKRRHHHPH

PPPCLMECIFNETGIYQNRHLDETKLESYINVVFADSTDLQNVATQAFVT

CAEKVADFEAKLSTERP

>54638945-1

MSSCCDVQRDDKAIKTCRKSLLTRNSTTTNNGDTRNLKSDKVALHACIAE

CAFKANGYLLTNGSVNVAALQKTYQQRYKNDANMSQLMVRSLNSCVDYAQ

KRTQQYQWMHTKDECDYYPATLLACILEQVY

>55239132-1

ADAPKKRCLTKPNVSKKVDMVIHQCQEEIKSSLIEDALKIFTAEHGQWHD

RRKRDEGGLDFSHPTIVSHEDKWIAGCLMQCVYRKNNAIDKNGWPTLDGL

VSLYTDGVNEQGYFMATLRGVDRCLKGTSKKYQIKRNDAAENFEQCEVAF

DVFDCISDMIT

>56417446-1

LVFFLLAISVSLSLGYDVSHFYICSTDYVKKERNFLCQVSKFNMNVPLPS

QGDEFFNCCMETSEWMNRSNKALLVDRLAKDMKKYGFDNRNSAIEKVGSD

CRREMGSKINGRGYLLCFLTDKR

>56417520-1

VALFLAAKAAEDVYESCAKDTIESGNHWEHHILCKVGTFNMQDHDSQSLM

DGTIKFMNCVFTKMAWMDGSKKELKVEKIISDLSLETDKKKKEQIGKCKS

TDPEQQNGMKYLECLLRRPN

>56417600-1

LALHVTGAPFWNAKNPDELQSIAARCMDEWSPKAKDPKAALKNWKEWRLQ

PSNDEATKCYTKCMLENIGFYEPAEKRLKGVRIMQQWETFSRYQSADREK

VHDLTDTFNFIRPLKSSSCTDVFNAYKDVHARHL

>61744153-1

WAWRSCQKGNYDPELVKKWMAFEIPDDEVTHCYIKCVWTHLGMYDETSQT

IRADRVKQQFKARGLSVPAEISHLEGSTGGSCVTIYKKTRAFLETQMP

>61807162-1

IVLVVICHAERPSQKCRRELKTEEECILHCEYKHYRFTDDQFRLNADQRG

DFRNIMRRYGAIRVDQESQLDKHLKKCANKVAKTPATSRKDKCRKISRYY

HCAVDNKL

>61807164-1

CFLGVANSLQFPRNPDQTRWAEKTCLKESWAPPNLINKWKQLEFPSTNLT

YCYVKCFVMYLGVYNETTKKFNVDGIRSQFTSQGLRPPNGLESLQKTSKG

TCKDVFRMSAGLIKKYKLEFVKAFH

>61807166-1

LTVLIVTCQAEHPGTKCRREFAIEEECINHCEYKHFGFTDDQFRIKKHHR

ENFKNAMSHYGAIRKDQEGELDKLLNRCAKKAKESPATSKRDKCYRIINY

YRCVVVDNN

>6634103-1

FPFNKDLQKKEEGLSYPDEDIVRKYEVCVFTKWGVFDEKEGFNHDRLVNH

FEPALNREEIERIIGTCA

>74099915-1

TLWAYRTCQREGKDPALVSKWMNWVLPDDPETHCYVKCVWTNLGSYDDNT

GSIMINTVATQFITRGMKVPAEVNNLSGSTSGSCSDIYKKTIGFFKSQKA

NIQ

>77415708-1

VALALVAAVAAQDKYTTKYDGVDLDEILKSDRLFNNYYKCLMDTGRCTPD

GNELKRILPDALKTDCAKCSEKQKSGTEKVINYLIDNRK

>81170834-1

LTIGISSIAAKPEVPIKSVANRQKFCYINSGFNYDKLEKIKKWRVFPSDM

SYKCYVGCFCTEMEFPMPDTRVMNEKIMWDALNRSLPQKKVNEIVDKCIN

VPVTNDVCETGEKLINCFIK

>85816138-1

FNIVKDCCVYPTFRFDQFKSQCGKYMPVGAPRISPCLYECIFNKTNTVVD

GAIHPDNARLMLEKLFGNQDFEEAYFNGLMGCSDSVQEMISNRRSRPQRK

TEQCSPFSLFYGICAQRYVF

>89214069-1

VVTFLVTIGVLLMQIAHQCAAKVGMGKYVDRSKANCFNLCQLEMHKVIVN

GSVHIPHIQNAQKNCVKLKEDDKCVLANRLKRCLQVSLN

>89214083-1

TFLLTMGLLSIRIQFDCINMLMMTIENFKSRTNCYHHCLFEGHGVIVDGT

VRIPHIQNAQNXCAKLKDDDPCMLAFRLKLCLFLSLNP
