# Supplementary material for: A machine learning approach for the identification of odorant binding proteins from sequence-derived properties
Source: BMC Bioinformatics. 2007 Sep 19;8:351. doi: 10.1186/1471-2105-8-351 (PMC2216042; doi:10.1186/1471-2105-8-351)
Supplement: Additional file 2 — Negative training dataset. This data provides 2157 protein sequences that are used for training. [file 1471-2105-8-351-S2.doc]

>21KD_DAUCA/23-183

APANQFIKTSCTLTTYPAVCEQSLSAYAKTIQNNPQELASTALQVSLTRT

QQAQTFMKRLNKFKGLKARQYAAIHDCLEEVEDSLDRVSRSCDEMKNLSH

AKGNDFTFRMSNVETWVSAALTDETTCMDGFAGKGMDGKIKESVRAQVVA

VARVTSNALAL

>A4GCT_HUMAN/195-327

FGFLPHHPFLWECMENFVEHYNSAIWGNQGPELMTRMLRVWCKLEDFQEV

SDLRCLNISFLHPQRFYPISYREWRRYYEVWDTEPSFNVSYALHLWNHMN

QEGRAVIRGSNTLVENLYRKHCPRTYRDLIKGP

>AANT_HDVP1/1-194

MSQTVARLTSKEREEILEQWVEERKNRRKLEKDLRRANKKIKKLEDENPW

LGNVVGLLRRKKDEDGAPPAKRPRQETMEVDSGPGRKPKARGFTDQERRD

HRRRKALENKKKQLAGGGKHLSQEEEEELRRLARDDDERERRTAGPRPGG

VNPMDGPPRGAPGGGFVPSLQGVPESPFSRTGEGIDIRGTQQFP

>ABEC1_MONDO/16-120

RRIKPWEFVAFFNPQELRKETCLLYEIKWGNQNIWRHSNQNTSQHAEINF

MEKFTAERHFNSSVRCSITWFLSWSPCWECSKAIRKFLDHYPNVTLAIFI

SRLYW

>ABFA_BACST/292-492

DEWNVWYHSNEADKLIEPWTVAPPLLEDIYNFEDALLVGCMLITLMKHAD

RVKIACLAQLVNVIAPIMTEKNGPAWKQTIYYPFMHASVYGRGVALHPVI

SSPKYDSKDFTDVPYLESIAVYNEEKEEVTIFAVNRDMEDALLLECDVRS

FEDYRVIEHIVLEHDNVKQTNSAQSSPVVPHRNGDAQLSDRKVSATLPKL

S

>ACA4_ARATH/866-1010

RAPIARTASFITKTMWRNIAGQSVYQLIVLGILNFAGKSLLKLDGPDSTA

VLNTVIFNSFVFCQVFNEINSREIEKINVFKGMFNSWVFTWVMTVTVVFQ

VIIVEFLGAFASTVPLSWQHWLLSILIGSLNMIVAVILKCVPVES

>ACSF_CYACA/82-215

EFLERSCTAEFSGFLLYKELARKLKEKMPVLAEGFLLMSRDEARHAGFLN

KAMADFNLTLDLGFMTKNRRYTFFKPKFIFYATYLSEKIGYWRYITIYRH

LEKHPEHRIYPIFKFFESWCQDENRHGDFFAAIV

>AGE1_CAEEL/62-141

LANVRTSLEIKLSDFKHQLFELIAPMKWGTYSVKPQDYVFRQLNNFGEIE

VIFNDDQPLSKLELHGTFPMLFLYQPDGIN

>AGLK_RHIME/292-352

EADDFLFEGTVSIVEALGEVTLLYIEGLVENEPIIAKMPGIARVGRGDKV

RFTADKAKLHL

>AGRB_BACHD/4-180

RLALTLAHQVKALNAEETESVEVLTFGFTIILHYLFTLLLVLAVGLLHGE

IWLFLQIALSFTFMRVLTGGAHLDHSIGCTLLSVLFITAISWVPFANNYA

WILYGISGGLLIWKYAPYYEAHQVVHTEHWERRKKRIAYILIVLFIILAM

LMSTQGLVLGVLLQGVLLTPIGLKVTR

>ALLC_PSEAE/20-164

GSQVVAVSDEWFAPASRMLQAGEPVWKEGVFDDSGKWMDGWETRRKRFEG

HDQAVIRLGVPGVLKGVDIDTRFFTGNHPPAASLDGCFCAEGDPDDSTSW

SEVLAAVGLQGDSHHYHPIDDERPWTHLRLNIYPDGGIARLRLYG

>AMN_ECOLI/225-460

DSPYIALSCAGGNWITAETEAPEEAISDLAWKKHQMPAWHLITADGQGIT

LVNIGVGPSNAKTICDHLAVLRPDVWLMIGHCGGLRESQAIGDYVLAHAY

LRDDHVLDAVLPPDIPIPSIAEVQRALYDATKLVSGRPGEEVKQRLRTGT

VVTTDDRNWELRYSASALRFNLSRAVAIDMESATIAAQGYRFRVPYGTLL

CVSDKPLHGEIKLPGQANRFYEGAISEHLQIGIRAI

>APLP2_RAT/42-204

GTGFAVAEPQIAMFCGKLNMHVNIQTGKWEPDPTGTKSCLGTKEEVLQYC

QEIYPELQITNVMEANQPVNIDSWCRRDKKQCRSHIVIPFKCLVGEFVSD

VLLVPENCQFFHQERMEVCEKHQRWHTVVKEACLTEGMTLYSYGMLLPCG

VDQFHGTEYVCCP

>APOC1_MOUSE/27-87

APDLSGTLESIPDKLKEFGNTLEDKARAAIEHIKQKEILTKTRAWFSEAF

GKVKEKLKTTF

>APOC3_CAVPO/1-91

MQPRVLLAVTLLALLVSARAEEIQESSLLGVMKDYMQQASKTANEMLTKV

QESQVAENAREWMTESLDSMKGYWTSLIGRLSGFLDSTPSS

>APOEB_BRARE/67-273

TLITDTMAELSSYSENLQTQMTPYASDAAGQLSKDLQLLAGKLQTDMTDA

KERSTQYLQELKTMMEQNADDVKNRVGTYTRKLKKRLNKDTEEIRNTVAT

YMSEMQSRASQNADAVKDRFQPYMSQAQDGATQKLGAISELMKAQAQEVS

EQLEVQAGALKEKLEETAENLRTSLEGRVDELTSLLAPYSQKIREQLQEV

MDKIKEA

>ARG56_CANAL/319-486

DLQKELFTDSGAGTLIRRGYRLINRNSLRDFGNPDLLRNALLRDPEIKTG

KVSVASYLKFLDSVQFKSYGDEPLEVLAIVVEQNDKIPKLDEFLSSKTGW

LNNVTDNIFNAIKKDYSQLCWVVNENDANLPWYFSKSDGSFAKNGQILFW

YGLNIDEASKLIKEFDSS

>AROK_CHLPN/9-171

PTSGKSSLGKALAKFLNLPFYDLDDLIVSNYSSALYSSSAEIYKAYGDQK

FSECEARILETLPPEDALISLGGGTLMYEASYRAIQTRGALVFLSVELPL

IYERLEKRGLPERLKEAMKTKPLSEILTERIDRMKEIADYIFPVDHVDHS

SKSSLEQASQDLI

>AROM_SHIFL/4-225

SLAILTIGIVPMQEVLPLLTEYIDEDNISHHSLLGKLSREEVMAEYAPEA

GEDTILTLLNDNQLAHVSRRKVERDLQGVVEVLDNQGYDVILLMSTANIS

SMTARNTIFLEPSRILPPLVSSIVEDHQVGVIVPVEEMLPVQAQKWQILQ

KSPVFSLGNPIHDSEQKIIDAGKELLAKGADVIMLDCLGFHQRHRDLLQK

QLDVPVLLSNVLIARLAAELLV

>ARPC5_CAEEL/4-146

TDYKKYNVDIFHPAHFEDIQEESADCGPNVQEVRQFLESNRLEYALQSVL

LNPPFGHSEQELKNRAVLLVAEVIHAFRQTDIEESVHKLSNENGDILMKY

IYKAMQLCSDSATCLSLLLWHSQLVSKFGQGSIVRVLSNRQRL

>ARS2_CRIGR/66-290

MPNRCGIIHVRGPMPPNRISHGEVLEWQKTFEEKLTPLLSVRESFLRKRP

RRWVVKTEQEVEKFVTSNTQELGKDKWLCPLSGKKFKGPEFVRKHIFNKH

AEKIEEVKKEVAFFNNFLTDAKRPALPEMKPAQPPGPAQILPPGLTPGLP

YPHQTPQGLMPYGQPALPIFGLWSWCCPPCRSQQEGLHIPMVRMALGAGT

MTLFEAKEDILGNLGTGWFEETRGP

>ASF1_YEAST/1-155

MSIVSLLGIKVLNNPAKFTDPYEFEITFECLESLKHDLEWKLTYVGSSRS

LDHDQELDSILVGPVPVGVNKFVFSADPPSAELIPASELVSVTVILLSCS

YDGREFVRVGYYVNNEYDEEELRENPPAKVQVDHIVRNILAEKPRVTRFN

IVWDN

>ASNA_UREPA/6-240

KAKINQTQKAIVEIKNSFQKHFAKNLNLSRVTAPLFVEGQSGLNDHLDHK

QKAVSFYAKKLDKTLEIVQSLAKWKRLALLDYGFSLYEGLYTDMNAIRAD

DDIDEIHSIYVDQWDWEILINNQDCNLDFLKSIVNKIYSTIRIVQLEIDQ

LYNPKQIILPDSITFISSQELEDLYPHLSPSRREYEFAKIHKAIFIYQIG

YPLKSGYIQSIRSPEYDNWNLNGDLIVYHKLNDQA

>ASNS1_PEA/1-160

CGILAVLGCSDDSQAKRVRILELSRRLKHRGPDWSGLHQHGDNYLAHQRL

AIVDPASGDQPLFNEDKSIIVTVNGEIYNHEELRKQLPNHKFFTQCDCDV

IAHLYEEHGENFVDMLDGIFSFVLLDTRDNSFIVARDAIGVTSLYIGWGL

DGSVWIASEL

>ATKC_MYCTU/2-189

RRQLLPALTMLLVFTVITGIVYPLAVTGVGQLFFGDQANGALLERDGQVI

GSAHIGQQFTAAKYFHPRPSSAGDGYDAAASSGSNLGPTNEKLLAAVAER

VTAYRKENNLPADTLVPVDAVTGSGSGLDPAISVVNAKLQAPRVAQARNI

SIRQVERLIEDHTDARGLGFLGERAVNVLRLNLALDRL

>ATP6_PARLI/21-230

IPMNLFSIVFALSWIAFIYPTNWAPSRFQSIWASFRANVLEMIFQNTSPN

TAPWAGLITTVFIVILSANVLGLFPYAFTATSHISLTYSLGFPIWMAVNI

LGFYLAFNSRLSHLVPQGTPSALIPLMVWIETLSLFAQPIALGLRLAANL

TAGHLLIFLLSTAIWLLSSSLMVSSIPIFVIFVLLFILEIGVACIEAYVF

TALVHFYLQQ

>ATPO_YEAST/32-208

VEGTYATALYQAAAKNSSIDAAFQSLQKVESTVKKNPKLGHLLLNPALSL

KDRNSVIDAIVETHKNLDGYVVNLLKVLSENNRLGCFEKIASDFGVLNDA

HNGLLKGTVTSAEPLDPKSFKRIEKALSASKLVGQGKSLKLENVVKPEIK

GGLIVELGDKTVDLSISTKIQKLNKVL

>ATR_HUMAN/1119-1225

MADYLQPKLLGILAFFNMQLLSSSVGIEDKKMALNSLMSLMKLMGPKHVS

SVRVKMMTTLRTGLRFKDDFPELCCRAWDCFVRCLDHACLGSLLSHVIVA

LLPLIHI

>ATX3_RAT/1-198

MESIFHEKQEGSLCAQHCLNNLLQGEYFSPVELSSIAHQLDEEERLRMAE

GGVTSEDYRTFLQQPSGNMDDSGFFSIQVISNALKVWGLELILFNSPEYQ

RLRIDPINERSFICNYKEHWFTVRKLGKQWFNLNSLLTGPELISDTYLAL

FLAQLQQEGYSIFVVKGDLPDCEADQLLQMIKVQQMHRPKLIGEELAH

>AZUP_ACHCY/33-121

VHMLNKGKDGAMVFEPASLKVAPGDTVTFIPTDKGHNVETIKGMIPDGAE

AFKSKINENYKVTFTAPGVYGVKCTPHYGMGMVGVVQVG

>B1_USTMA/185-410

ILKKFAREDRSRMKHLVRAKLSSSNQSTPPSSTYDSLSNNLDDVLSDNLG

RPLTPVDKQQFEDDWASMISWIKYGVKEKVGDWVYDLCAASKKTPKPGMP

RPVTTVANRHPARKTKPAAKPKSRTANPRASTTPSIDSTLDSSKLESTPE

LSMCSTADTSFSTFGSSLSMSHYNPFQDGNDILQSPTVKARGNRKVKALP

KRAGKQQPDEVDNGKIPFLCLSVAFV

>BAAT_HUMAN/2-155

IQLTATPVSALVDEPVHIRATGLIPFQMVSFQASLEDENGDMFYSQAHYR

ANEFGEVDLNHASSLGGDYMGVHPMGLFWSLKPEKLLTRLLKRDVMNRPF

QVQVKLYDLELIVNNKVASAPKASLTLERWYVAPGVTRIKVREGRLRGAL

FLPP

>BAG2_HUMAN/109-189

SLKHATRIIDEVVNKFLDDLGNAKSHLMSLYSACSSEVPHGPVDQKFQSI

VIGCALEDQKKIKRRLETLLRNIENSDKAIK

>BAK2_HUMAN/78-177

LAIIGDDINRRYDSEFQTMLQHLQPTAENAYEYFTKIASSLFESGINWGR

VVALLGFSYRLALHIYQRGLTGFLGQVTRFVVDFMLHHCIARWIAQRGGW

>BAMBI_HUMAN/4-111

HSSYIFIWLQLELCAMAVLLTKGEIRCYCDAAHCVATGYMCKSELSACFS

RLLDPQNSNSPLTHGCLDSLASTTDICQAKQARNHSGTTIPTLECCHEDM

CNYRGLHD

>BB1_RAT/1-57

MLSIDLQLSSICVPRMQLKTCYVEEIRGVVLEHSRHLWNDSHPLKLGGYR

PASLRSY

>BCSD3_ACEXY/10-153

FSLFLQALSWEIDDQAGIEVRNDLLREVGRGMAGRLQPPLCNTIHQLQIE

LNALLGMINWGYVKLELLAEEQAMRIVHEDLPQVGSAGEPSGTWLAPVLE

GLYGRWITSQPGAFGDYVVTRDVDAEDLNSVPTQTIILYMRTRS

>BEM3_YEAST/513-624

GTIQIEVLSTLYRDNEDDLSILIAIIDRKSGKEMFKFSKSIHKVRELDVY

MKSHVPDLPLPTLPDRQLFQTLSPTKVDTRKNILNQYYTSIFSVPEFPKN

VGLKIAQFISTD

>BGAL_BACME/55-231

SVYYQSLNGSWYFHFAENADGRVKNFFAPEFSYEKWDSISVPSHWQLQGY

DYPQYTNVTYPWVENEELEPPFAPTKYNPVGQYVRTFTPKSEWKDQPVYI

SFQGVESAFYVWINGEFVGYSEDSFTPAEFDITSYLQEGENTIAVEVYRW

SDASWLEDQDFWRMSGIFRDVYLYSTP

>BGLR_MOUSE/225-323

TYIDDITVITNVEQDIGLVTYWISVQGSEHFQLEVQLLDEDGKVVAHGTG

NQGQLQVPSANLWWPYLMHEHPAYMYSLEVKVTTTESVTDYYTLPVGIR

>BIND_ARBPU/252-479

AGGMQGGYGYPQAGGAQYGGQPVQGYMNQGPPMGQRPAAAGPAGGFGAPQ

GQPPVGQPIGEAAGGGEFLGEPGVGGESEFAEYSSSIGEGETINAEVMEK

IKAVLGATKIDLPVDINDPYDLGLLLRHLRHHSNLLANIGDPEVRNQVLT

AMQEEEEEEEQDAANGVRDNVLNNLNEGPGAGAVAGAAMAAGMPPYPGGA

QGGMRVGGQPQNPMGGNAYNPMTGYRQQ

>BIOW_AQUAE/1-239

MDLFSVRMRAQKNGKHVSGAERIVKKEELETAVKELLNRPKEFDFMNVKV

EKVKDFEVVKFNLKISTYSFKSPEEAREFAVKKLTQEGIKEEVAKKAVEI

LSKGANPKGGNMRGAVLMDIETGERLEEDKERGVRTIHFDWKDRKKVTEK

LLKEGYTLRTVDALALTFKNLFCGVVAELCWSDDPDYVTGYVSGKEIGYV

RITPLKEKGDPLGGRVYFVSRKELSEIIECLTQKVVLIE

>BNIP1_HUMAN/176-267

QTSSTITESLMGISRMMAQQVQQSEEAMQSLVTSSRTILDANEEFKSMSG

TIQLGRKLITKYNRRELTDKLLIFLALRLFLATVLYIVKKRL

>BT1A1_MOUSE/149-230

PQISMTVQENGEMELECTSSGWYPEPQVQWRTGNREMLPSTSESKKHNEE

GLFTVAVSMMIRDSSIKNMSCCIQNILLGQGK

>BXG_CLOBO/876-1073

GGRLIDSSGYGATMNVGSDVIFNDIGNGQFKLNNSENSNITAHQSKFVVY

DSMFDNFSINFWVRTPKYNNNDIQTYLQNEYTIISCIKNDSGWKVSIKGN

RIIWTLIDVNAKSKSIFFEYSIKDNISDYINKWFSITITNDRLGNANIYI

NGSLKKSEKILNLDRINSSNDIDFKLINCTDTTKFVWIKDFNIFGREL

>C5621_YERPE/22-127

ASLQDDMNILIANLGIVSSSTDTKVITSSLEKMRNAALDAQKAIPPKLEG

KAEDSPEIKDYRHGFDLLIEQIDKTKQWAEEGNIQEVKKSVGEVINIRNT

YHSRYR

>CATV_NPVBS/31-87

FETFLANYNKMYNDTSEKERRFSIFQQTLEEINYKNRLNDSAVYQINKFA

DLSKNEI

>CAV3_MOUSE/1-150

MMTEEHTDLEARIIKDIHCKEIDLVNRDPKNINEDIVKVDFEDVIAEPEG

TYSFDGVWKVSFTTFTVSKYWCYRLLSTLLGVPLALLWGFLFACISFCHI

WAVVPCIKSYLIEIQCISHIYSLCIRTFCNPLFAALGQVCSNIKVVLRRE

>CBL10_ARATH/245-425

YLAQVTIDNDNPLGRLDRWNLTFEWMRGEFINTMRGAYTHKKDPSECLYS

KAGQYYKDLDFSQVMNCQRKPAISDLPPEKKEDNMTGKLPFCCKNGTLLP

PIMDPSKSRSMFQLQVFKLPPDLNRTALYPPQHWKIDGVLNPQYKCGPPV

RVDPSQFPDPSGLLAVTYAISSWQVVCNITK

>CCG2_MOUSE/6-197

RGVQMLLTTVGAFAAFSLMTIAVGTDYWLYSRGVCKTKSVSENETSKKNE

EVMTHSGLWRTCCLEGNFKGLCKQIDHFPEDADYEADTAEYFLRAVRASS

IFPILSVILLFMGGLCIAASEFYKTRHNIILSAGIFFVSAGLSNIIGIIV

YISANAGDPSKSDSKKNSYSYGWSFYFGALSFIIAEMVGVLA

>CCMH_HAEIN/4-150

TWLFLTALLFSSVAFSAIDALNFSSPQQESDYHQLTQSLRCPQCQNNNIA

DSNATIAVDMRGKVFELLQEGKSKNDVVDYMVARYGNFVTYDPPITASTL

VLWIAPLLLVLLGVVFLLRRKPKTQSAVKSQEILTDEDNARLAELLN

>CCP2_HUMAN/20-191

VEVFTNQEVKEKFGGLFRTYDDCVTFQLFKSFRRVRINFSNPKSAARARI

ELHETQFRGKKLKLYFAQVQTPETDGDKLHLAPPQPAKQFLISPPSSPPV

SWQPINDATPVLNYDLLYAVAKLGPGEKYELHAGTESTPSVVVHVCDSDI

EEEEDPKTSPKPKIIQTRRPGL

>CD4_DELLE/314-387

MRVTKSPNSLTCEVLGPTSPRLILSLKKENQSMRVSDQQKLVTVLGPEAG

MWQCLLSDKGKVLLESKVKILPPV

>CDH_HELPJ/1-244

MKKAGFLFLAAMAIIVVSLNAKDPNVLRKIVFEKCLPNYEKNQNPSPCIE

VKPDAGYVVLKDINGPLQYLLMPTTHISGIENPLLLDPSTPNFFYLSWQA

RDFMSKKYGKPIPDYAISLTINSKKGRSQNHFHIHISCISLDVRKQLDNN

LKNINSRWSPLSGGLNGHKYLARRVTESELAQKSPFVMLAKEVPNAHKRM

GDYGLAVVQQSDNSFVLLATQFNPLTLNRASAEEIQDHECAILR

>CDTA_CAMJE/115-260

FLTILGPSGAALTVWALAQGNWIWGYTLIDSKGFGDARVWQLLLYPNDFA

MIKNAKTNTCLNAYGNGIVHYPCDASNHAQMWKLIPMSNTAVQIKNLGNG

KCIQAPITNLYGDFHKVFKIFTVECAKKDNFDQQWFLTTPPFTAKP

>CEAN_ECOLI/185-382

KEEKEKNEKEALLKASELVSGMGDKLGEYLGVKYKNVAKEVANDIKNFHG

RNIRSYNEAMASLNKVLANPKMKVNKSDKDAIVNAWKQVNAKDMANKIGN

LGKAFKVADLAIKVEKIREKSIEGYNTGNWGPLLLEVESWIIGGVVAGVA

ISLFGAVLSFLPISGLAVTALGVIGIMTISYLSSFIDANRVSNINNII

>CENPH_HUMAN/15-247

DSGGEGRAGGPPQVAGAQAACSEDRMTLLLRLRAQTKQQLLEYKSMVDAS

EEKTPEQIMQEKQIEAKIEDLENEIEEVKVAFEIKKLALDRMRLSTALKK

NLEKISRQSSVLMDNMKHLLELNKLIMKSQQESWDLEEKLLDIRKKRLQL

KQASESKLLEIQTEKNKQKIDLDSMENSERIKIIRQNLQMEIKITTVIQH

VFQNLILGSKVNWAEDPALKEIVLQLEKNVDMM

>CHD3_CAEEL/1509-1687

GKEYEIWHRRHDFWLLAAVAVYGYGRYQINFQDIMNDPKFSIVNEPFKQT

GADPATNFADVKNKFLARRFKLLEQSLVIEEQLRRAAHINKQQSPDQVGQ

LAQHFSELEHTADAHVNIARESNNGNRNANAILHKCLAQLDDLLSDLKTD

VARLPATISQVRPVTERLQMSERQILSRL

>CHEB_ECOLI/4-120

IRVLSVDDSALMRQIMTEIINSHSDMEMVATAPDPLVARDLIKKFNPDVL

TLDVEMPRMDGLDFLEKLMRLRPMPVVMVSSLTGKGSEVTLRALELGAID

FVTKPQLGIREGMLAYN

>CHSG_PETHY/1-231

MATVEEIRKAQRAEGPATVLAIGTANPSNCVDQSAYPDFLFRITTSDHKT

ELKEKFKHMCEGSMIKKRYLHLTEEILKNNPNICEHKAPSLNARQEIAVA

EAPKLGKRAAQKAIEEWSQSKSKITHLVFCTTTSVELPGADYQLTKLLGL

SPSVKRSMMYQQGCYGGGTALRLAKDLAENNKGARVLVVCVEITVMSFQA

PSRNDTDELDVLVGQALFADGASAVIIGSDP

>CHUR_HUMAN/1-112

MCGDCVEKEYPNRGNTCLENGSFLLNFTGCAVCSKRDFMLITNKSLKEED

GEEIVTYDHLCKNCHHVIARHEYTFSIMDEFQEYTMLCLLCGKAEDTISI

LPDDPRQMTLLF

>CI016_DROME/1-83

MSPKNNHDPSSSGDSGNTNVQEADLQEMEDVNNSLDALSCALDAVEQRTD

DIMSQLRELLNSNREIRRLIAEENDNAPESGDD

>CISA_BACSU/2-146

IAIYVRVSTEEQAIKGSSIDSQIEACIKKAGTKDVLKYADEGFSGELLER

PALNRLREDASKGLISQVICYDPDRLSRKLMNQLIIDDELRKRNIPLIFV

NGEYANSPEGQLFFAMRGAISEFEKAKIKERTSSGRLQKMKKGMI

>CITD_LACLA/1-95

MEIKQHALAGTLESSDVQIMIAPANNGISIDLISDVKKQFGKQIEATVRQ

VLAAYAIENADVQVIDKGALDLVIKARAIAVVERAIEAKDLNWEV

>CITXG_WEIPA/8-179

EAVDLLTVLDNREWRSRLQDKLKVTNSDKIVISAKLNIPGPIKNNDILQK

IFMDGWQTFVAGFECNSQYEMLFAERATGPEAFITVDGNLAAVKKTAILF

EETYALGRLFDIDVMANGQADYQLSREDLGFGPRLCLICGKPAKVCAKEQ

NHTLDEGYEVINQMYQGATSKE

>CKS1_SCHPO/20-101

FIDQIHYSPRYADDEYEYRHVMLPKAMLKAIPTDYFNPETGTLRILQEEE

WRGLGITQSLGWEMYEVHVPEPHILLFKREKD

>CLCB_BOVIN/1-227

MADDFGFFSSSESGAPEAAEEDPAAAFLAQQESEIAGIENDEGFGAPAGS

QGGLAQPGPASGASEDMGATVNGDVFQEANGPADGYAAIAQADRLTQEPE

SIRKWREEQRKRLQELDAASKVMEQEWREKAKKDLEEWNQRQSEQVEKNK

INNRIADKAFYQQPDADIIGYVASEEAFVKESKEETPGTEWEKVAQLCDF

NPKSSKQCKDVSRLRSVLMSLKQTPLS

>CLH_RAT/1423-1566

LMVLSPRLAHTRAVNYFSKVKQLPLVKPYLRSVQNHNNKSVNESLNNLFI

TEEDYQALRTSIDAYDNFDNISLAQRLEKHELIEFRRIAAYLFKGNNRWK

QSVELCKKDSLYKDAMQYASESKDTELAEELLQWFLQEEKRECF

>CNTF_CHICK/1-195

MAAADTPSATLRHHDLCSRGIRLARKMRSDVTDLLDIYVERQGLDASISV

AAVDGVPTAAVERWAEQTGTQRLLDNLAAYRAFRTLLAQMLEEQRELLGD

TDAELGPALAAMLLQVSAFVYHLEELLELESRGAPAEEGSEPPAPPRLSL

FEQKLRGLRVLRELAQWAVRSVRDLRQLSKHGPGSGAALGLPESQ

>COAE_MYCLE/185-399

RARDLWYHRILPFAYNLSQRQAVYAPAGLVTSDPIWLGQAKRIVARLKTT

CGHKALRVDHIGSTAVPHYPGFPDFQAKDIIDIQITVESLAMADELADPL

LSAGYPRLEHVTGDAAKTNARSTVDRYEHSSDPNLWHKRFHASADPGRPT

YVHIRVAGWPNQQFGLLFVDWLKANPGVRADYLDVKRTADRLAAGDMGRY

ADAKEPWLLDAYRRA

>COAG_LIMPO/23-195

PNVPTCLCEEPTLLGRKVIVSQETKDKIEEAVQAITDKDEISGRGFSIFG

GHPAFKECGKYECRTVTSEDSRCYNFFPFHHFPSECPVSVSACEPTFGYT

TSNELRIIVQAPKAGFRQCVWQHKCRAYGSNFCQRTGRCTQQRSVVRLVT

YDLEKGVFFCENVRTCCGCPCRS

>COATA_BPIF1/1-61

MKKIIIALFFAPFFTHATTDAECLSKPAFDGTLSNVWKEGDSRYANFENC

IYELSGIGIGY

>COAT_BBMV/2-190

TTSATGKALNRKQRRALNRSNRLRKEFQPVIVEPLASGQAVSLKTRTGYC

VTQFVSNNPEVKAKEVVSVSVKLPDHLAVEANRALKVGRISILLGLLPTV

AGTVKVCLTEKQDSPAESFKRALAVADSSKEVASAFYVDGFKDVSLGDLE

KDLSIYLYSEAALAANSIRIRMEVEHVMPKFITRFSPFA

>COAT_CLVN/45-234

WMNRPMYRKPMMYRMYRSPDIPRGCEGPCKVQSFEQRDDVKHLGICKVIS

DVTRGPGLTHRVGKRFCIKSIYILGKIWMDENIKKQNHTNNVMFYLLRDR

RPYGNTPQDFGQIFNMFDNEPSTATIENDLRDRFQVLRKFHATVIGGPSG

MKEQALVKRFYRLNHHVTYNHQEAGKYENHTENALLLYMA

>COAT_SPMV/5-157

RSRRSNRRAGSRAAATSLVYDTCYVTLTERATTSFQRQSFPTLKGMGDRA

FQVVAFTIQGVSAAPLMYNARLYNPGDTDSVHATGVQLMGTVPRTVRLTP

RVGQNNWFFGNTEEAETILAIDGLVSTKGANAPSNTVIVTGCFRLAPSEL

QSS

>COAT_STNV1/1-196

MAKQQNNRRKSATMRAVKRMINTHLEHKRFALINSGNTNATAGTVQNLSN

GIIQGDDINQRSGDQVRIVSHKLHVRGTAITVSQTFRFIWFRDNMNRGTT

PTVLEVLNTANFMSQYNPITLQQKRFTILKDVTLNCSLTGESIKDRIINL

PGQLVNYNGATAVAASNGPGAIFMLQIGDSLVGLWDSSYEAVYTDA

>COAT_WCMVM/24-163

TVSVASPAEIEAITKTWAETFKIPNDVLPLACWDLARAFADVGASSKSEL

TGDSAALAGVSRKQLAQAIKIHCTIRQFCMYFANIVWNIMLDTKTPPASW

SKLGYKEESKFAGFDFFDGVNHPAALMPADGLIRGPSDAE

>COBQ_THETN/282-451

KGERIGDCDVLIIPGTKNTIGDLKVLKDYGLDKEILNLREKGKFIVGICG

GFQMLGKVIKDPYHIESDTEEMEGLGLLSIETVIEREKTTSETKAFLGEE

LPDTLSSLKGLFVTGYEIHMGESYILGKGKHFSIVVERNKEKVKVLDGAV

SEDGRVFGTYIHGIFENSLF

>COMB_CLOAB/6-235

IISADDIKEEKVKNKTAVVIDMLRATSVITTALNNGCKRVVPVLTVEEAL

KKVKEYGKDAILGGERKGLKIEGFDFSNSPMEYTEDVVKGKTLIMTTTNG

TRAIKGSETARDILIGSVLNGEAVAEKIVELNNDVVIVNAGTYGEFSIDD

FICSGYIINCVMDRMKKLELTDAATTAQYVYKTNEDIKGFVKYAKHYKRI

MELGLKKDFEYCCKKDIVKLVPQYTNGEIL

>COQ4_SCHPO/48-267

PGHVPLSPLQRIFLVAGSAIMGLKAPWRGGDMISVLGDASGQPFFLHRLL

NKMLVDKTGREILKDKPRMTSKSLNLPFLRTLPPNTLGKIYVDWIDKEHV

GPDTRSPTRFVDDPEEAYVMQRYRESHDFYHAICNMPTNIEGELAIKWLE

FVNMGLPVGALSALFGPLRLNCEQASRFRRVYIPWSIRNGLNAKTLINVY

WEKELTNDIEDVRRRIRIEA

>CORA_HPBGS/32-217

DIDPYKEFGSSYQLLNFLPLDFFPDLNALVDTAAALYEEELTGREHCSPH

HTAIRQALVCWEELTRLITWMSENTTEEVRRIIVDHVNNTWGLKVRQTLW

FHLSCLTFGQHTVQEFLVSFGVWIRTPAPYRPPNAPILSTLPEHTVIRRR

GGSRAARSPRRRTPSPRRRRSQSPRRRRSQSPASNC

>COX2_ASCSU/98-217

LTVKVTGHQWYWSYEFSDIPGLEFDSYMKSLDQLELGEPRLLEVDNRCVV

PCDVNIRFCITSGDVIHSWALPSMSIKLDAMSGILSTLSYSFPVVGVFYG

QCSEICGANHSFMPVALEVT

>COX5_SCHPO/24-164

QPSGEAMIARPRLVDLDKRWGIMSQEEKDGLITDLYARQKQPWTTLSIEE

KKAAYWIAFGEHGPRAFSHISQKTVFWGTVAGLTIGVVLFGLIRTQAAPS

PRTMTREWQEKSNEYMKENKINPISGEASEGFKGRGQISGG

>CPG2_PORGI/25-229

QPAERGRNPQVRLLSAEQSMSKVQFRMDNLQFTGVQTSKGVAQVPTFTEG

VNISEKGTPILPILSRSLAVSETRAMKVEVVSSKFIEKKDVLIAPSKGVI

SRAENPDQIPYVYGQSYNEDKFFPGEIATLSDPFILRDVRGQVVNFAPLQ

YNPVTKTLRIYTEIVVAVSETAEAGQNTISLVKNSTFTGFEDIYKSVFMN

YEATR

>CR9EA_BACTA/301-505

ANPQLTREIYTDPIVYNPPANQGICRRWGNNPYNTFSELENAFIRPPHLF

ERLNRLTISRNRYTAPTTNSFLDYWSGHTLQSQHANNPTTYETSYGQITS

NTRLFNTTNGARAIDSRARNFGNLYANLYGVSSLNIFPTGVMSEITNAAN

TCRQDLTTTEELPLENNNFNLLSHVTFLRFNTTQGGPLATLGFVPTYVWT

REDVD

>CR9EA_BACTA/515-651

ITQLPWVKASEIGGGTTVVKGPGFTGGDILRRTDGGAVGTIRANVNAPLT

QQYRIRLRYASTTSFVVNLFVNNSAAGFTLPSTMAQNGSLTYESFNTLEV

THTIRFSQSDTTLRLNIFPSISGQEVYVDKLEIVPIN

>CR9EA_BACTA/70-296

LQTGINIVGRILGFLGVPFAGQLVTFYTFLLNQLWPTNDNAVWEAFMAQI

EELIDQKISAQVVRNALDDLTGLHDYYEEYLAALEEWLERPNGARANLVT

QRFENLHTAFVTRMPSFGTGPGSQRDAVALLTVYAQAANLHLLLLKDAEI

YGARWGLQQGQINLYFNAQQERTRIYTNHCVETYNRGLEDVRGTNTESWL

NYHRFRREMTLMAMDLVALFPFYNVRQ

>CSDE1_RAT/673-738

LRRATVECVKDQFGFINYEVGDSKKLFFHVKEVQDGIELQAGDEVEFSVI

LNQRTGKCSACNVWRV

>CSF2_MOUSE/18-136

APTRSPITVTRPWKHVEAIKEALNLLDDMPVTLNEEVEVVSNEFSFKKLT

CVQTRLKIFEQGLRGNFTKLKGALNMTASYYQTYCPPTPETDCETQVTTY

ADFIDSLKTFLTDIPFECK

>CSK2C_YEAST/38-213

WIDLFLGRKGHEYFCDVDPEYITDRFNLMNLQKTVSKFSYVVQYIVDDLD

DSILENMTHARLEQLESDSRKLYGLIHARYIITIKGLQKMYAKYKEADFG

RCPRVYCNLQQLLPVGLHDIPGIDCVKLYCPSCEDLYIPKSSRHSSIDGA

YFGTSFPGMFLQAFPDMVPKHPTKRY

>CUTC_XYLFA/26-227

AGLEVAAGSVASALAAQEGGAMRVELCHGLGGGGLTPSYGMLAVVRERLH

IPLYVLIRPRGGDFVFSEEEMEVMCCDVECCVRLGCDGVVLGALDPAGEV

DMGMMRVLIAVAGSLGVTFHRAIDVSADPGRTLEDVIALGCERVLTSGGR

SSALEGAETIAALVAQAAGRVVVMPGAGVSAGNVLELRVRTGAHEFHASA

RS

>CY24A_PIG/1-191

GQIEWAMWANEQALASGLILMTGGIVATAGQFTQWYLGTYSIAAGVLVCL

LEYPRGRRTKGSTMERCEQKYMTKVVKAFGPLSRNYYIRAFLHLGLSVPA

GFLLATILGTACLAIASGIYLLAAIRGEQWTPIEPKPKERPQVGGTIKQP

PSNPPPRPPPEARKKPGEEAVAGVPRGAPRKTPCPVTDEVV

>CYC3_DESSA/4-102

PADMVLKAPAGAKMTKAPVDFSHKGHAALDCTKCHHKWDGKAEVKKCSAE

GCHVBTSKKGKKSTPKFYSAFHSKSDISCVGCHKALKKATGPTKCGDCH

>CYF_PHOLA/213-330

NNAVYNASAAGVITAIAKADDGSAEVKIRTEDGTTIVDKIPAGPELIVSE

GEEVAAGAALTNNPNVGGFGQKDTEIVLQSPNRVKGRIAFLAAITLTQIL

LVLKKKQVERVQAGRDDL

>CYNS_AQUAE/77-149

PQQPVPPTDPFVYRLYEVVILYGPALKDVAHEMFGDGIMSAIDMSVELEK

VEQEGAERMVLTFNGKWLKYRKF

>CYSH_SYNP7/34-208

GLVLSTSFGIQSAVMLHLATQVQPDIPVIWIDTGYLPTETYRFAAELTER

LKLNLKVYQSEISPARMEALYGRLWESESVEDFNRYDQMRKVEPMNRALQ

ELGATAWLSGVRRQQTAHRQSMEIVELKRDRYAIRPILGWHSRDVYQYLT

AHDLPYHPLFDQGYVTVGDWHSSRP

>CYT2_YEAST/7-224

QGKCPVDEETKKLWLREHGNEAHPGATAPGNQLECSANPQDNDKTPEYHT

TVDLSQSREVSTIPRTNSDRNWIYPSEKQFYEAMMKKNWDPNSDDMKVVV

PLHNSINERVWNYIKSWEDKQGGEACGGIKLTNFKGDSKKLTPRAWFRSR

ILHLAKPFDRHDWQIDRCGKTVDYVIDFYSTDLNDANSQQQPLIYLDVRP

KLNSFEGFRLRFWKSLGF

>D1IP_RAT/1-218

MVKLGCSFSGKPGKETGDQDGAAMDSVPLISPLDVSQLQPSFPDQVVIKT

QTEYQLTSADQPKKFADLEGQRLACSHPEEGRRLPTARMIAFAMALLGCV

LIMYKAIWYDQFTCPDGFLLRHKICTPLTLEMYYTEMDPERHRSILAAIG

AYPLSRKHGTEMPAIWGNSYRAGKEEHKGTTPAAMTVSTAAAAAAAEGNE

PSGKPLDMREKEDPQKAE

>DAPB_CLOTE/7-117

VRIMLVGCNGKMGRIITHCSKDFNDIEIVAGVDKSSTSNLDFPVFENIHS

SSVECDVVLDFSRPSSLKSLISYCTEKKLPLVLCTTGYSKEELNLIEETS

KNIPIFKSANM

>DCK1_FOWPV/67-213

LQTHYCMKRVRMHLECFVPSRVNILERSIFSDRYVFAEAATALGYMDDPE

WALYCKQHDWYTDKLEIQFDGIIYLRTIPESCKERINEKSITEKNYPNIS

IDYLKTLHEKHELWLTQCKKVPVLIIDGEEDFIFDPCAKKKLINEVT

>DCR1_SCHPO/537-628

AVSLLYNFCNTLSRDVYTRYYPTFTAQPCLSGWYCFEVELPKACKVPAAQ

GSPAKSIRKAKQNAAFIMCLDLIRMGLIDKHLKPLDFRRKIA

>DCTD_YEAST/161-279

PSWDSYFMKLATLAASRSNCMKRRVGCVIVRECRVIATGYNGTPRHLTNC

FNGGCPRCNDGDSRNLHTCLCLHAEENALLEAGRDRVGQNATLYCDTCPC

LTCSVKIVQTGISEVVYSQ

>DDL_LEUME/4-134

KRVALIFGGNSSEHDVSKRSAQNFYNAIEATDKYEIIVFAIAQNGFFLDT

ESSKKILALEDEQPIVDAFMKTVDTSDPLARIHALKSAGDFDIFFPVVHG

NLGEDGTLQGLFKLLDKPYVGAPLRGHAVSF

>DEF_MYCPN/30-198

KAWLVLDDVKEINEPTKPVQFPLDQASLDCIAKMMAYVDASYNGDAEKYG

IIPGIGIAANQIGYWKQMFYIHLMDGGVEHKCLLINPKIINLSANKSFLK

SGEGCLSVPKMHQGYVIRHEWITITGFDWLQQKEITITATGLFGMCLQHE

FDHLQGRFYYHRINPLNPL

>DEGS_BACSU/12-170

DSILMKMLKTVDGSKDEVFQIGEQSRQQYEQLVEELKQIKQQVYEVIELG

DKLEVQTRHARNRLSEVSRNFHRFSEEEIRNAYEKAHKLQVELTMIQQRE

KQLRERRDDLERRLLGLQEIIERSESLVSQITVVLNYLNQDLREVGLLLA

DAQAKQDFG

>DEOC_MYCPI/2-215

NYNSLFDHTLLRADASVEEIKQLCDEAVKFNFFSVCVNPSYVPYVKEQLH

NSSVKICTVVGFPLGQTSTKQKVYETKIAIKEGADEIDMVLNISEFKENC

ACVVNEIRKYKKVCKKKILKVIVETALLSENEIEKATLVVIDGGADFIKT

STGFSSRGASIKDIEIMKNVIEKNNSKLKIKASGGIKTLTFVEELIKAGA

ERIGSSKSVEIIKE

>DGCR6_CHICK/1-197

MERFGGAGYEVAAAELSRQQERHYRLLSELQELVKALPSSCQQRLSYTTL

SDLALALLDGTVFEIVQGLLEIQHLTEKNLYSQRLKLHSEHRGLKQELFH

RHKEAQQCCRPHNLPLLRAAQQREMEAVEQRIREEQRMMDEKIVLELDQK

VIDQQSTLEKAGVSGFYITTNPQELTLQMNLLELIRKLQQKESESEK

>DHA_OCEIH/148-303

LEKSEGGKGILLGGIPGVSRGKVTVIGGGVVGTHAAKIALGLGAEVTIID

LNPVRLRQLDDIFGSSIQTLMSNPYNIAEAVKDSDLVIGSVLIPGRKAPK

LVTDEMIQSMQPGSVLVDVAIDQGGNFETVDHPTTHDEPIYVKHDVLHYA

VANIPG

>DHON_METGL/137-295

PLFKAVREGLAANRIEWIAGIINGTTNFILSEMREKGLAFADVLKEAQRL

GYAEADPTFDVEGIDAAHKLMILAAMLWLFVHSLCRGITKLDAVDITKRT

DKGVELRVHPTLIPEKRLICQCEWRNECCAGQGRCCWPTLYYGAGAGAEP

TASAVADLV

>DIF_DROME/255-356

LTITRLCSCAATANGGDEIIMLCEKIAKDDIEVRFYETDKDGRETWFANA

EFQPTDVFKQMAIAFKTPRYRNTEITQSVNVELKLVRPSDGATSAPLPFE

YY

>DNAA_THEMA/98-316

LNPDYTFENFVVGPGNSFAYHAALEVAKHPGRYNPLFIYGGVGLGKTHLL

QSIGNYVVQNEPDLRVMYITSEKFLNDLVDSMKEGKLNEFREKYRKKVDI

LLIDDVQFLIGKTGVQTELFHTFNELHDSGKQIVICSDREPQKLSEFQDR

LVSRFQMGLVAKLEPPDEETRKSIARKMLEIEHGELPEEVLNFVAENVDD

NLRRLRGAIIKLLVYKETT

>DPH4_YEAST/93-164

DEFSLDDFSFDEDKLEFMMNCPRCQFVGGFHFSESLLDECIDNVDAMERS

HSGYQLLTQCSACSLWLKVNFD

>DPO3_MYCPU/404-562

YVVYDIETTGLSPMLNELIQFGASVIKNGRIIETHHFFIKPKSKLDSFTT

KLTGITQEHLEKGYELQEALEKISSIFKARIMVAHNAAFDHNFLKQKFID

NNIEFEEMISIDTLNLAKVLNPIYRSYRLGEVASKLSVVYDPSIAHRADY

DSSVLTNIF

>DPOL_HBVW2/603-845

GSWGTLPQDHIVQKIKHCFRKLPVNRPIDWKVCQRIVGLLGFAAPFTQCG

YPALMPLYACIQAKQAFTFSPTYKAFLSKQYMNLYPVARQRPGLCQVFAD

ATPTGWGLAIGHQRMRGTFVAPLPIHTAELLAACFARSRSGAKLIGTDNS

VVLSRKYTSFPWLLGCTANWILRGTSFVYVPSALNPADDPSRGRLGLSRP

LLRLPFQPTTGRTSLYAVSPSVPSHLPVRVHFASPLHVAWRPP

>DTXH_CORBE/225-404

SCINLDWDVIRDKTKTKIESLKEHGPIKNKMSESPNKTVSEEKAKQYLEE

FHQTALEHPELSELKTVTGTNRVFAGANYAAWAVNVAQVIDSETADNLEK

TTAALSILPGIGSVMGIADGAVHHNTEEIVAQSIALSSLMVAQAIPLVGE

LVDIGFAAYNFVESIINLFQVVHNSYNRSA

>DTXH_CORBE/26-212

GADDVVDSSKSFVMENFSSYHGTKPGYVDSIQKGIQKPKSGTQGNYDDDW

KGFYSTDNKYDAAGYSVDNENPLSGKAGDVVKVTYPGLTKVLALKVDNAE

TIKKELGLSLTEPLMEQVGTEEFIKRFGDGASRVVLSLPFAEGSSSVEYI

NNWEQAKALSVKLEINFETRGKRGQDAMYEYMAQACA

>DTXH_CORBE/406-559

SPGHKTQPFLHDGYAVSWNTVEDSIIRTGFQGESGHDIKITAENTPLPIA

SVLLPTIPGKLDVNKSKTHISVNGRKIRMRCRAIDGDVTFCRPKSPVYVG

NGVHANLHVAFHRSSSEKIHSNEISSDSIGVLGYQKTVDHTKVNSKLSLF

FEIK

>DXR_CHLPN/4-129

LAVLGSTGSIGRQTLEIVRRYPSEFKIISMASYGNNLRLFFQQLEEFAPL

AAAVYNEEVYNEACQRFPHMQFFLGQEGLTQLCIMDTVTTVVAASSGIEA

LPAILESMKKGKALALANKEILVCAG

>DYL2_SCHMA/1-89

MGERKAVIKNADMHEDMQETAVHTAAAALDKYEIEKDVAAYIKKEFDRKY

NPNWHCIVGKHFGSYVTHETQHFIYFYLQERAFLLFKSG

>DYR_SCHPO/1-231

MSKPLKVLCLHGWIQSGPVFSKKMGSVQKYLSKYAELHFPTGPVVADEEA

DPNDEEEKKRLAALGGEQNGGKFGWFEVEDFKNTYGSWDESLECINQYMQ

EKGPFDGLIGFSQGAGIGAMLAQMLQPGQPPNPYVQHPPFKFVVFVGGFR

AEKPEFDHFYNPKLTTPSLHIAGTSDTLVPLARSKQLVERCENAHVLLHP

GQHIVPQQAVYKTGIRDFMFSAPTKEPTKHP

>E434_ADEM1/130-283

RELVVQKFLLGTRFNEYYPQYRVHANRYVNPGLEYVGSVWCGKHFIYVRA

DGAEFARLKGLRARLGQGVLFCESLLSCYVVIVCQQCACPPTDAQVDHCM

RLLSFTLRRWQNLLLGRSGSSPLIPGFDIPRNRTERLRQRMLHRFYSYRT

PIYR

>ECTC_CHRSL/1-130

MIVRNLEECRKTERFVEAENGNWDSTRLVLADDNVGFSFNITRIHPGTET

HIHYKHHFEAVFCYEGEGEVETLADGKIHPIKAGDMYLLDQHDEHLLRGK

EKGMTVACVFNPALTGREVHREDGSYAPVD

>EF1B_METTH/1-88

MGDVVATIKVMPESPDVDLEALKKEIQERIPEGTELHKIDEEPIAFGLVA

LNVMVVVGDAEGGTEAAEESLSGIEGVSNIEVTDVRRL

>EFP_MYCGE/6-63

EAKNLRNGQTIFGPNKEILLVLENTFNKTAMRQGIVKTKVKNLRTGAIVW

LEFTGDKL

>ELOA1_DROME/439-559

SLFDLCTRVLQKNIDALEYTGGVPFEVLRPVLERATPQQLLNFEEYNPYL

MDDSDVLWQQHVQRHCRSQRREEMETWREMFLRCQEEKDRKLSILAESIK

ASQKISEAPVRKTQLAFVDSM

>ENAH_MOUSE/1-108

MSEQSICQARAAVMVYDDANKKWVPAGGSTGFSRVHIYHHTGNNTFRVVG

RKIQDHQVVINCAIPKGLKYNQATQTFHQWRDARQVYGLNFGSKEDANVF

ASAMMHAL

>END4_CHLPN/26-247

IYEGRDIGASTVQIFTANQRQWQRRALKEEVIEDFKAALKETDLSYIMSH

AGYLINPGAPDPVILEKSRIGIYQEILDCITLGISFVNFHPGAALKSSKE

DCMNKIVSSFSQSAPLFDSSPPLVVLLETTAGQGTLIGSNFEELGYLVQN

LKNQIPIGVCVDTCHIFAAGYDITSPQGWEDVLNEFDEYVGLSYLRAFHL

NDSMFPLGANKDRHAPLGEGYI

>ENO_ZYMMO/3-134

AIVSIHGRQVVDSRGNPTVEVDVTLEDGSFGRAAVPSGASTGVHEAVELR

DGDKTRWGGKGVTKAVHAVNNEIANAIIGLEAEDQELIDQTMIKLDGTPN

KGKFGANAILGVSLAVAKAAAEARGLPLYRYV

>ENV_RSVSA/157-402

LNVSMWDEPHELQLLGSQSLPNITNIAQISGITGGCVGFRPQGVPWYLGW

SRQEATRFLLRHPSFSKSTEPFTVVTADRHNLFMGSEYCGAYGYRFWNMY

NCSQVGRQYRCGNARSPRPGLPEIQCTRRGGKWVNQSQEINESEPFSFTV

NCTASSLGNASGCCGKAGTILPGKWVDSTQGSFTKPKALPPAIFLICGDR

AWQGIPSRPVGGPCYLGKLTMLAPKHTDILKVLVNSSRTGIRRKRS

>EPHA1_HUMAN/27-204

EVTLMDTSKAQGELGWLLDPPKDGWSEQQQILNGTPLYMYQDCPMQGRRD

TDHWLRSNWIYRGEEASRVHVELQFTVRDCKSFPGGAGPLGCKETFNLLY

MESDQDVGIQLRRPLFQKVTTVAADQSFTIRDLASGSVKLNVERCSLGRL

TRRGLYLAFHNPGACVALVSVRVFYQRC

>EPN1_HUMAN/17-140

YSEAEIKVREATSNDPWGPSSSLMSEIADLTYNVVAFSEIMSMIWKRLND

HGKNWRHVYKAMTLMEYLIKTGSERVSQQCKENMYAVQTLKDFQYVDRDG

KDQGVNVREKAKQLVALLRDEDRL

>ESSQ_ECOLI/4-71

MDKLTTGVAYGTSAGNAGFWALQLLDKVTPSQWAAIGVLGSLVFGLLTYL

TNLYFKIKEDRRKAARGE

>ETS2A_XENLA/87-170

FNGFAKKRFRLGILSNPWLWDENNVFQWLWWAAKEFSLQNVNFQKFLMNG

HELCSLGKERFLALAPDFVGDILWEHLEEMMKEH

>EVG1_DROME/35-249

KLPMWPSERIPPGGAGAFHSAKVQYSKETADLIRLLVKESKMSMLVRKQI

DESLRNGEPLPLPEPPRPNTNNDPDKETLAILDRARNAKRKNLRQIEASG

AYKQSYYRPPADNRMHGEKAKSQLQFTMAGTHLPDPAIKPRRRPREEQLV

TEEDLINELLDQINERAEWLTEMESMGQGKKYRPEIRDQIAERLRRIQAL

ESKMKMKSNGGFRFV

>EVI2A_HUMAN/1-227

MEHTGHYLHLAFLMTTVFSLSPGTKANYTRLWANSTSSWDSVIQNKTGRN

QNENINTNPITPEVDYKGNSTNMPETSHIVALTSKSEQELYIPSVVSNSP

STVQSIENTSKSHGEIFKKDVCAENNNNMAMLICLIIIAVLFLICTFLFL

STVVLANKVSSLRRSKQVGKRQPRSNGDFLASGLWPAESDTWKRTKQLTG

PNLVMQSTGVLTATRERKDEEGTEKLT

>EX7L_RICPR/203-374

PSVIIVARGGGSIEDLWSFNDEILVRAAYNSKIPIISAVGHEADYTLIDL

AADKRAPTPTAAAEFAVPVRSILNNTIQSYEKILFNNTNRLIKYHEQSIV

NYDKIHSYFSYYINNRQQLLDEIGFNLLDVLIRFIALKETKIKSFSKERI

NYAKIINYKILELTHQTAYLFK

>FABA_ERWCT/29-159

LPAPNMLMMDRVVKMTEDGGKYGKGFVEAELDITPDQWFFGCHFIGDPVM

PGCLGLDAMWQLVGFYLGWLGGEGKGRALGVGEVKFSGQVLPTAKKVTYR

IHFKRVINRRLVMGIADGEVLVDGQHIYAAD

>FADR_VIBCH/72-279

NQFMETSGLHILDTLMTLDAENATSIVEDLLAARTNISPIFMRYAFKLNK

ESAERIMINVIESCEALVNAPSWDAFIAASPYAEKIQQHVKEDSEKDELK

RQEILIAKTFNFYDYMLFQRLAFHSGNQIYGLIFNGLKKLYDRVGSYYFS

NPQARELAMEFYRQLLAVCQSGEREHLPQVIRQYGIASGHIWNQMKMTLP

SNFTEDDC

>FANCA_HUMAN/1253-1316

REELLVFLFFFSLMGLLSSHLTSNSTTDLPKAFHVCAAILECLEKRKISW

LALFQLTESDLRLG

>FAS1_CANAL/1001-1062

KPVPFVPVLDERFEFFFKKDSLWQSEDLESVVDEDVQRTCILHGPVASQY

TSKVDEPIGDIL

>FASC_STRPU/392-495

PHGFVGMKEGKAEVACNRSNFDVFTVTYKEGGYTIQDSCGKYWSCDDSSR

IVLGEAAGTFFFEFHELSKFAIRAESNGMLIKGEQSGLFTANGSEVSKDT

LWEF

>FBP3_STRPU/445-559

CDLEGVWFNECNDQITIIKTSTGMMLGDHMTFTERELGVAAPTVMVGYPS

NNYDFPSFGITVVRDNGRTTTSWTGQCHLCDGQEVLYTTWIESSMVSTCE

EIKRANKVGQDKWTR

>FCERB_MOUSE/59-203

LVGLICLCFGTIVCSVLYVSDFDEEVLLLYKLGYPFWGAVLFVLSGFLSI

ISERKNTLYLVRGSLGANIVSSIAAGTGIAMLILNLTNNFAYMNNCKNVT

EDDGCFVASFTTELVLMMLFLTILAFCSAVLFTIYRIGQELESKK

>FDHD_METTH/14-245

DERRRVPEKVVNDIEVRIRINGGMEQRFTASPQALEEFATGYLLGEGLVD

SVDDIVSIEISDNIIDAEIESGDLDIRRELVMGSDCFGGWRQRVEMVGPV

DSDLRVRADDIFLAFKRMVKSAVVWRMTGGTHVAALVTGDEFRVFEDVSR

HVAVDKVIGSGAMDGVNFRESFIVYSGRMPADMLIKVVRAGVPIIASNAA

PTSSGYDAAQRTGLTMLGFVRGKRFNIYSHPE

>FEN_METJA/140-228

SLMGIPYVEAPSEGEAQASYMAKKGDVWAVVSQDYDALLYGAPRVVRNLT

TTKEMPELIELNEVLEDLRISLDDLIDIAIFMGTDYNPG

>FET5_YEAST/157-305

ERVITLSDHYHENYKTVTKEFLSRYNPTGAEPIPQNILFNNTMNVTLDFT

PGETYLFRFLNVGLFVSQYIILEDHEMSIVEVDGVYVKPNFTDSIYLSAG

QRMSVLIKAKDKMPTRNYAMMQIMDETMLDVVPPELQLNQTIQMRYGHS

>FIBP_ADEM1/388-612

LWTGLPIGNNGTFHTKQDCKIFLSLTRLGPMVHGTFMLQAPQYELTTNGM

REITFSFNSTGGLEQPAPVTYWGALDPPPTAKAAEIENQKRVKKRAAPDP

PVEPPPKRRGDLAVLFAKVAEQAMELAKEQAVQAQPPEHVNTDWADHMNL

LRFMPNTLVYPTAATIAANLQFHDTRLSLRRATLKIRLNGSPDSAYQLGF

MLELVGTQSASIVTDTISFWYYAED

>FLAA_SPIAU/37-275

NTGLHAPTTIDYSRQAGSAYSAEDKAAMKISLAIPSWEIELASSSQTVEN

QTLSLVTAAPVKQDAARYGGETVMGVRIHFPSFGINSFAVIKPPFTIPAY

ATLGDATAQNAVAGGQFDGFGVLKNVGVIKSIQINILGRNYLNRLSLLLE

DQNGDEREIVMGYLNFDGWKSLQWNNPNYQTEVRNRDLQIVPLYPRSAPL

IKLKGIKIHRDGSQEGGDIVSYIKDIKVIYDQAVVDRNS

>FLAV_HELPJ/6-155

IFFGTDSGNAEAIAEKISKAIGNAEVIDVAKASKEQFNGFTKVILVAPTA

GAGDLQTDWEDFLGTLEASDFANKTIGLVGLGDQDTYSETFAEGIFHIYE

KAKAGKVVGQTPTDGYHFEASKAVEGGKFVGLVIDEDNQDDLTDERIAKW

>FLGE_RHIME/171-286

SGAPVGDVSTTSLVVYDSQGNTRILDFNYEKTGANAWTLEIVDRASGDAL

TVPPVTLAFNAAGELTTSPATVVANGALIVPPPATGAVVGSITIDFSKTT

QLGYAFNADGGSIDGN

>FLGH_SALTY/22-231

CAWIPAKPLVQGATTAQPIPGPVPVANGSIFQSAQPINYGYQPLFEDRRP

RNIGDTLTIVLQENVSASKSSSANASRDGKTSFGFDTVPRYLQGLFGNSR

ADMEASGGNSFNGKGGANASNTFSGTLTVTVDQVLANGNLHVVGEKQIAI

NQGTEFIRFSGVVNPRTISGSNSVPSTQVADARIEYVGNGYINEAQNMGW

LQRFFLNLSP

>FLIA_ECOLI/90-172

PRSVRRNAREVAQAIGQLEQELGRNATETEVAERLGIDIADYRQMLLDTN

NSQLFSYDEWREEHGDSIELVTDDHQRENPLQQ

>FLIC_SERMA/29-164

IERLSSGLRINSAKDDAAGQAISNRFTANIKGLTQASRNANDGISLAQTT

EGALNEVNDNLQNIRRLTVQAQNGSNSTSDLKSIQDEITQRLSEINRISE

QTDFNGVKVLSSDQKLTIQVGANDGETTDIDLKKID

>FLID_THEMA/27-124

VSGLDTQSIINAILEAESQPLQNLTEKYEKYELMQEAYTEVKTKLREFRD

LVYSFKLQSTVVQKTAVSSSSLLSAEASSVAVTGVYHVKIVQTATYTT

>FLID_THEMA/349-593

GQKAHLQISMDGTNWADVYSDTNDVEYNGVTFHISGMTSETITVDVRVDT

DAIVEKIKEFVDKWNETMDYLNEKLTEESITDKDEEEMTEEEKMKGVLKG

DDLLEEIFSRLRGFITYKAEGDINYLWELGISTGDIGTGYENMMKGHLEV

DEEKLKQIVEEDPNKVWEFFGGENGFATQLDDYLWELVKFNGRIDQVAGI

SGRIEREQRFLATQIASWIERLSKREQELWRKFSVMEEVISQLQS

>FLIE_HELPY/11-109

LSPFSELNTDNRTKREESGSTFKEQKGGEFSKLLKQSINELNNTQEQSDK

ALADMATGQIKDLHQAAIAIGKAETSMKLMLEVRNKAISAYKELLRTQI

>FLII_TREPA/146-356

TGVRVLDSLLAVGCGQRLGIFSGSGVGKSTLMGMIARNTDADVSVIALIG

ERGREVMDFVAHDLGPEGLKRSVIVSATSDESPLARVRGAYTATAIAEYF

RDQGKQVLLLFDSLTRFAKAQREIGLASGELPATRGYTPGVFETLPKLLE

RAGSFSMGSVTAFYTVLVDGDDLDEPISDAVRGIVDGHIVLSRALAQRNH

YPAIDVLQSVS

>FLIM_BORBU/49-240

YDFKRPDKFSKEQVRTVSSFHEAFARYTTTSLSALLRKMVHVHVASVDQL

TYEEFIRSIPNPTTLAIINMDPLKGSAIFEVDPTIAFAIVDRLFGGDGDT

IKDKSRDLTEIEQSVMESVIIRILANMREAWSQVVDLRPRFGHIEVNPQF

AQIVPPTEMIILVTLEVKIGKVEGLMNFCLPYITIEPIVSKL

>FLIT_ECOLI/1-121

MNHAPHLYFAWQQLVEKSQLMLRLATEEQWDELIASEMAYVNAVQEIAHL

TEEVDPSTTMQEQLRPMLRLILDNESKVKQLLQIRMDELAKLVGQSSVQK

SVLSAYGDQGGFVLAPQDNLF

>FLPA_METVO/1-227

MAKIKEKFDNVFELDLGDGIKRIGTKSLVPNKRVYGEKLVNVKNTEYRVW

NPNKSKLGASIINGLKEMPIKKGSKVLYLGASAGTTPSHVADVAEDSPVY

AVEFAPRIMREFIESCEGRKNLFPILGDANKPEEYANIVEKVDVIFEDVA

QPNQAEILIKNAKWFLKKGGYGMISIKARSVDVTENPRVIFEAQKEIMEQ

NGFKIVDAINIEPFEKDHMLFVGIWNG

>FLP_ZYGBA/135-380

LSNNVGAEISKLAETKDSTWSFIERTMDLIEARTRQPTTRVAYRFLLQLT

FMNCCRANDLKNADPSTFQIIADPHLGRILRAFVPETKTSIERFIYFFPC

KGRCDPLLALDSYLLWVGPVPKTQTTDEETQYDYQLLQDTLLISYDRFIA

KESKENIFKIPNGPKAHLGRHLMASYLGNNSLKSEATLYGNWSVERQEGV

SKMADSRYMHTVKKSPPSYLFAFLSGYYKKSNQGEYVLAETLYNPL

>FMF4_ECOLI/29-277

QPGDIIIGGEITSPSVKWLWKTGEGLSSFSNTTNEIVKRKLNISVPTDEL

FLAAKMSDGIKGVFVGNTLIPKIEMASYDGSVITPSFTSNTAMDIAVKVK

NSGDNTELGTLSVPLSFGAAVATIFDGDTTDSAVAHIIGGSAGTVFEGLV

NPGRFTDQNIAYKWNGLSKAEMAGYVEKLMPGQSASTSYSGFHNWDDLSH

SNYTSANKASYLSYGSGVSAGSTLVMNLNKDVAGRLEWVAPVTITVIYS

>FRDC_PASMU/5-132

TSKRKKYVREMKPTWWKKLDFYKLYIAREATAIPTLWFCLVLLYGVISLG

SLDSFGNFISFLKNPIVIILNIITLGAMLLNTVTYYVMTPKVLNIIVKNE

RINPNIITMALWAVTAFISLVILVFMYV

>FRDC_WOLSU/1-243

MTNESILESYSGVTPERKKSRMPAKLDWWQSATGLFLGLFMIGHMFFVST

ILLGDNVMLWVTKKFELDFIFEGGKPIVVSFLAAFVFAVFIAHAFLAMRK

FPINYRQYLTFKTHKDLMRHGDTTLWWIQAMTGFAMFFLGSVHLYIMMTQ

PQTIGPVSSSFRMVSEWMWPLYLVLLFAVELHGSVGLYRLAVKWGWFDGE

TPDKTRANLKKLKTLMSAFLIVLGLLTFGAYVKKGLEQTDPNI

>FRDD_HAEIN/2-113

VDQNPKRSGEPPVWLMFGAGGTVSAIFLPVVILIIGLLLPFGLVDVHNLI

TFAYSWIGKLVILVLTIFPMWCGLHRIHHGMHDLKVHVPAGGFIFYGLAT

IYTVWVLFAVIN

>FRE6_YEAST/550-692

FDHLLLLSGGTGLPGPLDHAIKLSRNPDKPKSIDLIMAIKNPSFLNGYKS

EILELKNSRSHVNVQVYLTQKTAVTKAANARDQLIHFDDIMTELTSFAHI

GNARPNFSNVIENAIKSTPPGDSLAVVCCGPPVLVDDVRNTVS

>FRE7_YEAST/323-417

FMASTIANVSIVGEGCVELIVKDVEMAYSPGQHIFVRTIDKGIISNHPFS

IFPSAKYPGGIKMLIRAQKGFSKRLYESNDDMKKILIDGPYGGIE

>FRG1_DROME/72-260

RSYLKAMDNGLFTLGAPHNAGDGPDPEEIFTAFPINDRKVAFKSGYGKYL

KIEKDGMVTGRSEAVGGMEQWEPVFEEQRMALLSETGHFMSIDPQDDACV

ALRKKVGQHEICKVRSNASRDVVIDTEPKEEKGDLGEVEKNYVKKFQKFQ

DKKMRINQNDVKELEQAKAQGSLHETLLDRRSKMKADRY

>FRHB_METTH/6-86

KEIVSARSTDREIQKLAQDGGIVTGLLAYALDEGIIEGAVVAGPGKEFWK

PEPMVAMTSDELKAAAGTKYTFSPNVLMLKK

>FSPM_LYCES/1-96

MAAKNSEMKFAIFFVVLLTTTLVDMSGISKMQVMALRDIPPQETLLKMKL

LPTNILGLCNEPCSSNSDCIGITLCQFCKEKTDQYGLTYRTCNLLP

>FTRL_METTH/1-145

MEINGTLIEDTFSEAFTGRCVRATITARDMETVRRAALDATATPGAVIGR

VEGGVESFLGGEDTPDGRPGAVVQFYYALDDMEKFQVELSYRIRQDILVK

PFTALYSSTPDPDGYLDMMKHVGHCGDGYEWLEEFNGREMINVPV

>FTRL_METTH/147-295

VPDFKIESRMGYREAIMGANFWYMCRDPDTVLEAGRAAIRAIEEVEGVVT

PFDICSAASKPETNYPWIGPTTNHPYCPSLKEVLGDESRVPEGVGYIPEI

VINGLTMEALEEAMRAGIEAVCRYDGVLRVSAGNYDGKLGDHRIDLHGV

>FTRV_SPIOL/95-168

VGCKVKVKSPLKVYHVPKLPEVELTPDMVGVIKQYVGFWKGKYISPNYPF

KVEYRIDVPDRGSVKLVVHLKEEE

>FUCI_SHIFL/175-355

KSYLSLGGVSMGIAGSIVDHNFFESWLGMKVQAVDMTELRRRIDQKIYDE

AELEMALAWADKNFRYGEDENNKQYQRNAEQSRAVLRESLLMAMCIRDMM

QGNSKLADIGRVEESLGYNAIAAGFQGQRHWTDQYPNGDTAEAILNSSFD

WNGVREPFVVATENDSLNGVAMLMGHQLTGT

>FUCI_SHIFL/4-174

ISLPKIGIRPVIDGRRMGVRESLEEQTMNMAKATAALLTEKLRHACGAAV

ECVISDTCIAGMAEAAACEEKFSSQNVGLTITVTPCWCYGSETIDMDPTR

PKAIWGFNGTERPGAVYLAAALAAHSQKGIPAFSIYGHDVQDADDTSIPA

DVEEKLLRFARAGLAVASMKG

>GAGD3_HUMAN/1-111

MSWRGRSTYRPRPRRSLQPPELIGAMLEPTDEEPKEEKPPTKSRNPTPDQ

KREDDQGAAEIQVPDLEADLQELCQTKTGDGCEGGTDVKGKILPKAEHFK

MPEAGEGKSQV

>GAG_JSRV/272-477

FKQLKELKIACSQYGPTAPFTIAMIESLGTQALPPNDWKQTARACLSGGD

YLLWKSEFFEQCARIADVNRQQGIQTSYEMLIGEGPYQATDTQLNFLPGA

YAQISNAARQAWKKLPSSSTKTEDLSKVRQGPDEPYQDFVARLLDTIGKI

MSDEKAGMVLAKQLAFENANSACQAALRPYRKKGDLSDFIRICADIGPSY

MQGIAM

>GAG_SIVCZ/2-140

GARASVLTGGKLDRWEKVRLRPGGRKRYMMKHLVWASRELERFACDPGLM

ESKEGCTKLLQQLEPALKTGSEGLRSLFNTLAVLWCIHSDITVEDTQKAL

EQLKRHHGEQQSKTESNSGSREGGASQGASASAGISGNY

>GAG_SMRVH/2-92

QASSHSENDLFISHLKESLKVRRIRVRKKDLVSFFSFIFKTCPWFPQEGS

IDSRVWGRVGDCLNDYYRVFGPETIPITTFNYYNLIRDVLT

>GAL7_YEAST/193-365

EVSQELKSFDKYKREHNTDLFADYVKLESREKSRVVVENESFIVVVPYWA

IWPFETLVISKKKLASISQFNQMVKEDLASILKQLTIKYDNLFETSFPYS

MGIHQAPLNATGDELSNSWFHMHFYPPLLRSATVRKFLVGFELLGEPQRD

LTSEQAAEKLRNLDGQIHYLQRL

>GAL7_YEAST/3-191

EEFDFSSHSHRRYNPLTDSWILVSPHRAKRPWLGQQEAAYKPTAPLYDPK

CYLCPGNKRATGNLNPRYESTYIFPNDYAAVRLDQPILPQNDSNEDNLKN

RLLKVQSVRGNCFVICFSPNHNLTIPQMKQSDLVHIVNSWQALTDDLSRE

ARENHKPFKYVQIFENKGTAMGCSNLHPHGQAWCLESIP

>GALT2_HUMAN/139-318

SVVITFHNEARSALLRTVVSVLKKSPPHLIKEIILVDDYSNDPEDGALLG

KIEKVRVLRNDRREGLMRSRVRGADAAQAKVLTFLDSHCECNEHWLEPLL

ERVAEDRTRVVSPIIDVINMDNFQYVGASADLKGGFDWNLVFKWDYMTPE

QRRSRQGNPVAPIKTPMIAGGLFVMDKFYF

>GATA4_XENLA/1-175

MYQSIAMATNHGPSGYEGTGSFMHSATAATSPVYVPTTRVSSMIHSLPYL

QTSGSSQQGSPVSGHNMWAQAGVESSAYNPGTSHPPVSPRFTFSSSPPIT

APSSREVSYSSPLGISANGREQYSRGLGATYASPYPAYMSPDMGAAWTAS

PFDSSMLHNLQNRAVTSRHPNIEFF

>GATB_CHLPN/336-485

TFFEVACKDCKNFRSLSNWVTVEFGGRCKTLGVKLPSSGIFPEGVAQLVN

AIDQGVITGKIAKEIADLMMESPGKNPEEILKEKPELLPMSDEGELQKII

AEVVLANPESIVDYKNGKTKALGFLVGQIMKRTAGKAPPKRVNELLLLEL

>GATE_AERPE/299-394

IKKVLSSGGKVIAVKLPRMKGIIGMEIMPGRRFGTELADYARFWGGVGGI

IHSDELPGYGITGDEVEKIYEAVGGDPGVDAFALVADKPSNALRAA

>GCH1_HAEIN/96-200

EMVQVNDITLTSTCEHHFVTIDGKVCVAYYPKDWVIGLSKINRIVSFFAQ

RPQVQERLTEQLLTAFQTILETDDVAVYVKATHFCVKARGIRDTNSYTVT

SAYGG

>GCM2_MOUSE/21-123

WDINDPQMPQEPTHFDHFREWPDGYVRFIYSSQEKKAQRHLSGWAMRNTN

NHNGHILKKSCLGVVVCARACALKDGSHLQLRPAICDKARLKQQKKACPN

CHS

>GCYDB_DROME/1-170

MYGMLYESVQHYIQQEYGMETWRKVCQIVDCKHQSFKTHQIYPDKLMPDF

AAALSASTGESFDFCMNFFGRCFVRFFSNFGYDKMIRSTGRYFCDFLQSI

DNIHVQMRFTYPKMKSPSMQLTNMDDDGAVILYRSGRTGMSKYLIGQMTE

VAKEFYGLDMTAYVLESQND

>GDIR_HUMAN/1-204

MAEQEPTAEQLAQIAAENEEDEHSVNYKPPAQKSIQEIQELDKDDESLRK

YKEALLGRVAVSADPNVPNVVVTGLTLVCSSAPGPLELDLTGDLESFKKQ

SFVLKEGVEYRIKISFRVNREIVSGMKYIQHTYRKGVKIDKTDYMVGSYG

PRAEEYEFLTPVEEAPKGMLARGSYSIKSRFTDDDKTDHLSWEWNLTIKK

DWKD

>GEF1_SCHPO/315-506

LIKELVTTEAAYLNDLIAIQQSYGLRVKECSALNPVDAQTVFGDIESLLT

FTVEFHSRLYQAGEGSWRVNLDTQLIDPLPCNLGLIFLESLSEIGQIYTG

YCNRQDSVFKIITKWREKPATASWIMEGDKIVQKYTNAWDLGSLIIKPLQ

RLLKYPLLLQKIIDVTPESSSERPDLVLSYQLLQELISGINQ

>GELA_DICDI/648-744

APSAEHSYAEGEGLVKVFDNAPAEFTIFAVDTKGVARTDGGDPFEVAING

PDGLVVDAKVTDNNDGTYGVVYDAPVEGNYNVNVTLRGNPIKNMPID

>GEMI6_MOUSE/1-166

MSEWMKKSPLEWEDYVYKEVRVIACEKEYKGWLLTTDPVSANIVLVNFLE

DGRLSVTGIMGHSVQTVETISEGDHRVREKLMHVFASGDCKGYSPEDLEE

KRTSLKKWLEKNHIPVTEQGDAQRTLCVAGVLTIDPPYAPENCSSSNEII

LSRIQDLIQGHLSASQ

>GFPL_DISST/5-223

KSVIKEEMLIDLHLEGTFNGHYFEIKGKGKGQPNEGTNTVTLEVTKGGPL

PFGWHILCPQFQYGNKAFVHHPDNIHDYLKLSFPEGYTWERSMHFEDGGL

CCITNDISLTGNCFYYDIKFTGLNFPPNGPVVQKKTTGWEPSTERLYPRD

GVLIGDIHHALTVEGGGHYACDIKTVYRAKKAALKMPGYHYVDTKLVIWN

NDKEFMKVEEHEIAVARHH

>GGA2_YEAST/20-165

QALGNPLLRKIQRACRMSLAEPDLALNLDIADYINEKQGAAPRDAAIALA

KLINNRESHVAIFALSLLDVLVKNCGYPFHLQISRKEFLNELVKRFPGHP

PLRYSKIQRLILTAIEEWYQTICKHSSYKNDMGYIRDMHRLLKYKG

>GLGB_CHLPN/621-718

WVDFHDIENNVIAYYRFAGSNRSSALLCVHHFSASTFPSYVLRCEGVKHC

ELLLNTDDESFGGSGKGNRAPVVCQDQGVAWGLDIELPPLATVIYLVT

>GLGB_SYNY3/24-103

VLGCHPLGDHGKVNQWVIRAYLPTAEAVTVLLPTDRREVIMTTVHHPNFF

ECVLELEEPKNYQLRITENGHERVIYDPYG

>GLNB1_METTL/4-101

IKAIVRPDKVDDIVDSLENAGYPAFTKINSVGRGKQGGLKVGEIFYDELP

KTILLIAVNDDEVDEVVGLIKSSASTGNFGDGKIFIQPITEAYTIRTG

>GLPP_BACSU/8-182

PILPAIRNMKQFDEFLNSSFSYGVILDIHLGQLKGVIKEAQKHGKNMMVH

VDLIQGIKHDEYGAEFICQDIKPAGIISTRSNVIAKAKQKKIYAIQRLFL

LDTSAMEKSMEFIGKHKPDFIEVLPGIVPSLIQEIKEKTGIPIFAGGFIR

TEEDVEQALKAGAVAVTTSNTKLWK

>GNPI_HUMAN/15-250

WAAKYIRNRIIQFNPGPEKYFTLGLPTGSTPLGCYKKLIEYYKNGDLSFK

YVKTFNMDEYVGLPRDHPESYHSFMWNNFFKHIDIHPENTHILDGNAVDL

QAECDAFEEKIKAAGGIELFVGGIGPDGHIAFNEPGSSLVSRTRVKTLAM

DTILANARFFDGELTKVPTMALTVGVGTVMDAREVMILITGAHKAFALYK

AIEEGVNHMWTVSAFQQHPRTVFVCDEDATLELKVK

>GOS1_SCHPO/27-163

AKILLEERRKLLEEANSSADENDSHSMATIKSHFERLKRDEQLLNGVLKK

YDAKQEVLSPEELRDAQNFLEMQEANSLDNSIRGTNELLERAYATREDFD

YQNSVLGNVTNRINGAAMSIPFINQILRKTSIRRRRD

>GP6R_CHLPN/1-249

MGNLKTLLESRFKKNTPTKMEALARKRMEGDPSPLAVRLSNPTLSSKEKE

QLRHLLQHYNFREQIEEPDLTQLCTLSAEVKQIHHQSVLLHGERITKVRD

LLKSYREGAFSSWLLLTYGNRQTPYNFLVYYELFTLLPEPLKIEMEKMPR

QAVYTLASRQGPQEKKEEIIRNYRGERKSELLDRIRKEFPLVETDCRKTS

PVKQALAMLTKGSQILTKCTSLSSDEQIILEKLIKKLEKVKSNLFPDTK

>GPDA_STRCO/182-326

TDVVGCELGGAVKNVIGLAVGIADGMGLGDNAKGSLITRGLAETTRLGVA

LGADPLTFSGLAGLGDLVATCSSPLSRNHTFGTNLGKGMTLEETNAVTKQ

TAEGVKSCESVLDLARRHGVDMPITETVVAIVHEGKSPVVAVKEL

>GPMI_PORPU/371-486

DMSAELVTQKSISAINKGIYSCIVINYANADMLGHTGKLKETIRSIETVD

NCIALLFDAVSQSNGTLIITADHGNAECMLTNEGTSCTSHTTNLVPFILI

EGEQATISGHGGQVEF

>GRCA_ECO57/3-108

TGIQITKAANDDLLNSFWLLDSEKGEARCIVAKAGYAEDEVVAVSKLGDI

EYREVPVEVKPEVRVEGGQHLNVNVLRRETLEDAVKHPEKYPQLTIRVSG

YAVRFN

>GRDA_CLOST/1-150

SRFTGKKIVIIGDRDGIPGPAIEECLKPIDCEVIFSSTECFVCTAAGAMD

LENQKRIKEATEKFGAENLVVLIGAAEAEAAGLAAETVTAGDPTFAGPLA

GVELGLRVYHAVEPEFKDEVDAQIFDDQVGMMEMVLNVDEIIEEMQSIRS

>GROU_DROME/1-137

MYPSPVRHPAAGGPPPQGPIKFTIADTLERIKEEFNFLQAQYHSIKLECE

KLSNEKTEMQRHYVMYYEMSYGLNVEMHKQTEIAKRLNTLINQLLPFLQA

DHQQQVLQAVERAKQVTMQELNLIIGQQIHAQQVPGG

>GT2D1_HUMAN/705-780

LREQVQDLFNKKYGEALGIKYPVQVPYKRIKSNPGSVIIEGLPPGIPFRK

PCTFGSQNLERILAVADKIKFTVTRP

>GUC2A_HUMAN/1-115

MNAFLLFALCLLGAWAALAGGVTVQDGNFSFSLESVKKLKDLQEPQEPRV

GKLRNFAPIPGEPVVPILCSNPNFPEELKPLCKEPNAQEILQRLEEIAED

PGTCEICAYAACTGC

>GUC2C_HUMAN/815-1002

VEPELYEEVTIYFSDIVGFTTICKYSTPMEVVDMLNDIYKSFDHIVDHHD

VYKVETIGDAYMVASGLPKRNGNRHAIDIAKMALEILSFMGTFELEHLPG

LPIWIRIGVHSGPCAAGVVGIKMPRYCLFGDTVNTASRMESTGLPLRIHV

SGSTIAILKRTECQFLYEVRGETYLKGRGNETTYWLTG

>GVPD_HALME/48-294

FTIRGLDVLERDGDVLYVSTRVDQDTVHEMYFREHSSLDKTHILDLSQDP

FELPLDVDVPFEKLGLDSLLEWIQQIKAASKRLTIAFDSWELIYEYLASR

HDDSPDIETVTTQLVSLARQENIRLLLVSETADSSPLEYIVDGVVTLQVA

EDERGRTRRYLRLEKLRGVRIGNRLQPITLADGQFQAITPVELPTVRTGA

NNGTWEPRTNTKAKFSTGIGDLDPILSGGYNRGSVIHLDLGTDLSRD

>GWIN3_POPSP/32-195

AVLDFYGREVQAGASYLIDQEDFRVVNATINPICNSDVILSTGIEGLPVT

FSPVINSTDGVIREGTLITVSFDASTCGMAGVTPMWKIGFNSTAKGYIVT

TGGVDRLNLFKITKFESDSSFYQLSYCPNSEPFCECPCVPVGANSDKYLA

PNVSYADFRFKPDA

>HA1T_MOUSE/27-205

GSHSLRYFYTALSRPAISEPWYIAVGYLDDTQFVRFNSSGETATYKLSAP

WVEQEGPEYWARETEIVTSNAQFFRENLQTMLDYYNLSQNGSHTIQVMYG

CEVEFFGSLFRAYEQHGYDGPDYIALNEDLKTWTAADTAAEITRSKWEQA

GYTELRRTYLEGPCKDSLLRYLENRKKTQ

>HCT1_CHLPN/2-117

ALKDTAKKMKDLLDSIQHDLAKAEKGNKAAAQRVRTDSIKLEKVAKLYRK

ESIKAEKSGLLKRKPSTKAPAKVKKTAEKKAPKKSSAAAAKTSKAVKASK

PASKKTAAKKVKKPSK

>HCT2_CHLPN/1-172

MIGAQKKQSGKKTASRAVRKPAKKVAAKRTVKKATVRKTAVKKPAVRKTA

AKKTVAKKTTAKRTVRKTVAKKPAVKKVAAKRVVKKTVAKKTTAKRAVRK

TVAKKPVARKTTVAKGSPKKAAACALACHKNHKHTSSCKRVCSSTATRKH

GSKSRVRTAHGWRHQLIKMMSR

>HEM3_PAEMA/5-221

TIVVGTRQSQLALTQTEQVIGDLKELCRAHGLPFEFEIKKIVTKGDRILD

VTLSKIGGKGLFVKEIEQAMLDGEIDMAVHSMKDMPSVLPEGLINGGVPL

RKDPRDALISRSGLHLHELPQGARVGTSTLRRSSQLLAYRPDLVLEPVRG

NIDSRLRKLEEEGFDAIILAAAGLQRMGWENRVTAYLSADVCLPAVGQGA

LGIECRENDAELRDVLS

>HEX_VIBVU/184-313

QYSKVKDLGADAVSAHILPTPLETSVHEGSLNIAQGINIVSDALPADQVE

ALNFRFETLGVNTGTGVPVNVTIKADSSKKSGSYTLDVTSSGIRIVGVDK

AGAFYGVQSLAGLVTVGKDTINQVSINDEP

>HFQ_CLOPE/6-66

NNLQDIFLNNARKERIPVTIFLVNGVQLKGIVKGFDSFTVVLDSDGKQQL

VYKHAISTVSP

>HGH1_YEAST/303-363

HLESILLLCTTHAGREYLRDKSVYPLVRELHKNVENEDIGELCYRIVNML

MRGEPGAGAVE

>HIS4_BRAJA/5-234

ILFPAIDLKNGQCVRLEQGDMARATVFNLNPAAQAQSFAEQGFEYLHVVD

LDGAFAGKPVNAQAVEAMLKTIKIPVQLGGGIRDLATVEAWLEKGITRVI

IGTAAVRDPDLVKAAAKKFPGRVAVGLDARDGKVAVEGWAETSQVTALEI

AQRFEDAGVAAIIFTDIARDGLLKGLNLDATIALADSISIPVIASGGLAS

IDDVKAMLTPRAKKLAGAIAGRALYDGRLD

>HIS5_YEAST/5-212

HVIDVESGNLQSLTNAIEHLGYEVQLVKSPKDFNISGTSRLILPGVGNYG

HFVDNLFNRGFEKPIREYIESGKPIMGICVGLQALFAGSVESPKSTGLNY

IDFKLSRFDDSEKPVPEIGWNSCIPSENLFFGLDPYKRYYFVHSFAAILN

SEKKKNLENDGWKIAKAKYGSEEFIAAVNKNNIFATQFHPEKSGKAGLNV

IENFLKQQ

>HIS7_TRIHA/61-205

TGIGFLDHMLHALAKHAGWSMALNCKGDLHIDDHHTAEDCCIAVGTTFAK

ALGALTGVARFGYAYAPLDEALSRAVVDLSNRPYTVVDLGLKREKLGELS

CEMIPHCLQSFAQAARITLHVDCLRGDNDHHRAESAFKALAVAVR

>HISQ_SALTY/9-221

ILQGAIVTLELALSSVVLAVLIGLVGAGAKLSQNRVTGLIFEGYTTLIRG

VPDLVLMLLIFYGLQIALNVVTDSLGIDQIDIDPMVAGIITLGFIYGAYF

TETFRGAFMAVPKGHIEAATAFGFTHGQTFRRIMFPAMMRYALPGIGNNW

QVILKATALVSLLGLEDVVKATQLAGKSTWEPFYFAVVCGLIYLVFTTVS

NGVLLLLERRYSV

>HMCS2_RAT/50-223

WPKDVGILALEVYFPAQYVDQTDLEKFNNVEAGKYTVGLGQTRMGFCSVQ

EDINSLCLTVVQRLMERTKLPWDAVGRLEVGTETIIDKSKAVKTVLMELF

QDSGNTDIEGIDTTNACYGGTASLFNAANWMESSYWDGRYALVVCGDIAV

YPSGNPRPTGGAGAVAMLIGPKAP

>HMGYA_SOYBN/11-78

SLPPYPEMIVKTLEALNEPNGSNKSAISKYIETTYGELPDATVLGSHLNK

MKDSGELSFKQNNYMKAD

>HMOX_FUGRU/15-219

RDLSEQIKKVTKDVHVRAESTELMLSFQRGQVTLQQYKLLLCSLYEIYLA

LEEEMDRNCDHPSVAPIYFPAELARLATIEKDLEFFFGPDWREKIVVPAA

TERYCHRIRQIGQENPEYLIAHAYTRYLGDLSGGQVLGRIAQKSMKLGGS

EGLSFFAFPGVSSPNLFKRLYRSRMNSVELTEEQRSAVLQEALGAFEFNI

QVFED

>HMP_ECOLI/263-373

TLISAGVGQTPMLAMLDTLAKAGHTAQVNWFHAAENGDVHAFADEVKELG

QSLPRFTAHTWYRQPSEADRAKGQFDSEGLMDLSKLEGAFSDPTMQFYLC

GPVGFMQFTAK

>HNT1_YEAST/32-125

KSEIPSFKLIETKYSYAFLDIQPTAEGHALIIPKYHGAKLHDIPDEFLTD

AMPIAKRLAKAMKLDTYNVLQNNGKIAHQEVDHVHFHLIPKRDE

>HRCA_MYCCA/104-324

IDKTINYASEIISELTKMTAVVIKSKNIKNIKLKIELILLSEFLASVLFI

FSDGDVQNKMFNLKDISLSDLKIAIKLFSDFLVDVKLDEIDQYLNDLKHQ

LSLSIKQYDYVLNTFINTILESKNEQKETHGMRYMLENPEFNDTNKLKNA

VKLVEQLSPFDWFNIAYESNKNMNKIAIKIGNEIDQINDDISMIATELKI

GNSSTVLTLVGPKRGRLQPSK

>HTR1_HALSA/250-495

DSAADDVQQVSASAEEIAATIDDLASRSEDVATASDAARDSSKSALDEMS

SIETEVDDAVGQVEQLRDQVAEITDIVDVITDIGEQTNMLALNASIEAAR

AGGNADGDGFSVVADEVKDLAEETQDRANEIAAVVEKVTAQTEDVTASIQ

QTRTRVESGSETVESTLRDIRTIADSIAEVSNSIDEIQRTTSEQAETVQS

TATSVERVAGLSDDTTALASDAESAVIGQRESAEEIAASLEQFQNT

>HXA9B_BRARE/1-174

MSTLGTLSYYADSHLPHENDDHLAPRFSSGPVVQQQSRELTLLEYSEQEP

YTFQAKSSIFGASWSPVQPTGASIAYHPYIHHPCSTGDSDGASVRPWALE

PLPALPFTGLSTDTHQDIKLEPLVGSGECTTHTLLVAETDNNTTQTERKV

PDDAVSNGSHDEKIPAETKLDLDP

>HXK_DEBOC/21-222

EYLLKELTELEGLLTVSGETLRKITDHFISELEKGLSKQGGNIPMIPGWV

MDFPTGKEMGDYLAIDLGGTNLRVVLVKLGGNRDFDTTQSKFALPENMRT

AKSEELWEFIAECLQKFVEEEFRNGVLSNLPLGFTFSYPASQGSINEGYL

QRWTKGFDIEGVEGHDVVPMLQAAIEKRKVPIEVVALINDTTGTLVASMY

TD

>HXK_DEBOC/224-469

EAKMGLFSGTGCNGAYYDVVDNIPKLEGKVPDDIKSSSPMAINCEYGAFD

NEHIILPRTKYDIQIDEESPRPGQQAFEKMISGYYLGEVLRLILLDLTSK

QLIFKDQDLSKLQVPFILDTSIPARIEEDPFENLSDVQELFQEILGIQTT

SPERKIIRRLAELIGERSARLSICGIAAICKKRGYKTAHCAADGSVYNKY

PGFKERAAKGLRDIFQWESEEDPIVIVPAEDGLGAGAAIIAALTEK

>HYPA_DEIRA/1-113

MHEASIALALIDVAGDVLREHGAARASALTVRVGQWSSVVPEALAAAFPA

CAEGTPLAGARLSIERVPGVGECPQHGPVELEVWRGLRCPLCGAPTPRLL

QGDELELDQLELD

>IF2B_ARCFU/11-133

RAFEKLADKDVVRRERFEIPRVSIQREGARTILKNFSQIAKTLNRSEDHL

YKYIVKSLGTAGFIDNGRLVLQGKFTESELQKEVDDYVRLYVLCRECNSP

DTEFIKEERVLMLRCLACGAKHP

>IF2_DEIRA/1-53

MSKVRIYTLAKDLGVDNHKMLEILDGLGVSYKSVSSTIDEENVEIIKQIL

ADE

>IF5A_SULAC/69-131

IIEKHVGQILADKGDNLTIMDLESYETFDLEKPTENEIVSKIRPGAEIEY

WSVMGRRKIVRVK

>IF6_YEAST/3-204

TRTQFENSNEIGVFSKLTNTYCLVAVGGSENFYSAFEAELGDAIPIVHTT

IAGTRIIGRMTAGNRRGLLVPTQTTDQELQHLRNSLPDSVKIQRVEERLS

ALGNVICCNDYVALVHPDIDRETEELISDVLGVEVFRQTISGNILVGSYC

SLSNQGGLVHPQTSVQDQEELSSLLQVPLVAGTVNRGSSVVGAGMVVNDY

LA

>IFI4_MOUSE/245-415

LHSEPLTVMVLTATDPFEYESPEHEVKNMLHATVATVSQYFHVKVFNINL

KEKFTKKNFIIISNYFESKGILEINETSSVLEAAPDQMIEVPNSIIRNAN

ASPKICDIQKGTSGAVFYGVFTLHKKTVNRKNTIYEIKDGSGSIEVVGSG

KWHNINCKEGDKLHLFCFHLK

>IGF2_HUMAN/112-166

KFFQYDTWKQSTQRLRRGLPALLRARRGHVLAKELEAFREAKRHRPLIAL

PTQDP

>IHH_BRARE/24-184

CGPGRGYGKRRTPRKLTPLAYKQFSPNVAEKTLGASGRYEGKVTPSSERF

KELTPNYNPDIIFKDEENTGADRMMTQRCKDKLNSLAISVMNLWPGVRLR

VTEGWDEDGLHSEESLHYEGRAVDITTSDRDRNKYRMLARLAVEAGFDWV

YYESKGHVHCS

>IL12A_MOUSE/1-215

MCQSRYLLFLATLALLNHLSLARVIPVSGPARCLSQSRNLLKTTDDMVKT

AREKLKHYSCTAEDIDHEDITRDQTSTLKTCLPLELHKNESCLATRETSS

TTRGSCLPPQKTSLMMTLCLGSIYEDLKMYQTEFQAINAALQNHNHQQII

LDKGMLVAIDELMQSLNHNGETLRQKPPVGEADPYRVKMKLCILLHAFST

RVVTINRVMGYLSSA

>IL17C_HUMAN/1-193

MTLLPGLLFLTWLHTCLAHHDPSLRGHPHSHGTPHCYSAEELPLGQAPPH

LLARGAKWGQALPVALVSSLEAASHRGRHERPSATTQCPVLRPEEVLEAD

THQRSISPWRYRVDTDEDRYPQKLAFAECLCRGCIDARTGRETAALNSVR

LLQSLLVLRRRPCSRDGSGLPTPGAFAFHTEFIHVPVGCTCVL

>IL2_PIG/7-151

LCCIALTLALMANGAPTSSSTKNTKKQLEPLLLDLQLLLKEVKNYENADL

SRMLTFKFYMPKQATELKHLQCLVEELKALEGVLNLGQSKNSDSANIKES

MNNINVTVLELKGSETSFKCEYDDETVTAVEFLNKWITFCQSIYS

>IL3_BOVIN/18-132

APQAKGLPVVTSRTPYSMLMKEIMDDLKKITPSPEGSLNSDEKNFLTKES

LLQANLKVFMTFATDTFGSDSKIMKNLKEFQPVLPTATPTEDPIFIENKN

LGDFRMKLEEYLVII

>IL4_RAT/2-140

GLSPHLAVTLFCFLICTGNGIHGCNDSPLREIINTLNQVTEKGTPCTEMF

VPDVLTATRNTTENELICRASRVLRKFYFPRDVPPCLKNKSGVLGELRKL

CRGVSGLNSLRSCTVNESTLTTLKDFLESLKSILRGKYL

>IL5_MERUN/19-131

ALEIPMSAVVKETLIQLSTHRALLTSNETVRLPVPTHKNHQLCIGEIFQG

LDILKNQTARGGAVETLFQNLSLIKKYIDRQKEKCGEERRRARQFLDYLQ

EFLGVMSTEWTME

>IL9_HUMAN/1-142

MLLAMVLTSALLLCSVAGQGCPTLAGILDINFLINKMQEDPASKCHCSAN

VTSCLCLGIPSDNCTRPCFSERLSQMTNTTMQTRYPLIFSRVKKSVEVLK

NNKCPYFSCEQPCNQTTAGNALTFLKSLLEIFQKEKMRGMRG

>ILF2_DROME/105-345

QVGSFKKGTILTGNNVADVVVILKTLPTKEAVDALAKKVEADLKASMKTE

VLTKGDQHTVQIHERGFDIANVHAKVRILIATLPQNLRKLEPEIHLDHKL

MQSHLAAIRHTRWFEENAHHSSIKVLIRILKDLTRRFDAFSPLSAWMLDL

IAHLAIMNNPSRQALPINLAFRRVFQLLSAGLFLPGSAGITDPTEPGHIR

VHTAMTLEQQDVCCYTSQTLLRVLAHGGYKHILGLEGNTSV

>ILVC_LEPIC/55-219

SLKGKTIAVIGYGSQGHAQAQNMKDSGLKVIIGLKEGSKSIQDAKNAGFE

VYSVSEASQKADIIQILAPDTIQADLYKKDIEPNLKKGDALVFSHGFNIH

YDFIKPPKEVDVYMVAPKGPGHLVRRVYTEGGGVPCLIAIYQDSSGEAKK

RALAHAAGVGGGRAG

>ILVI_ECOLI/195-354

QIKRALQTLVAAKKPVVYVGGGAITAGCHQQLKETVEALNLPVVCSLMGL

GAFPATHRQALGMLGMHGTYEANMTMHNADVIFAVGVRFDDRTTNNLAKY

CPNATVLHIDIDPTSISKTVTADIPIVGDARQVLEQMLELLSQESAHQPL

DEIRDWWQQI

>IMA2_CAEEL/13-106

EEGKDEGGRLQQYKNLTKHEELRRRRTECSVEIRKQKGADMMMKRRNIVD

VDEGGNSESELEEPEKISHQQSSTRLSNDEIRAILSNNPSEDDM

>IMM3_ECOLI/1-82

GLKLDLTWFDKSTEDFKGEEYSKDFGDDGSVMESLGVPFKDNVNNGCFDV

IAEWVPLLQPYFNHQIDISDNEYFVSFDYRDG

>IMP3_YEAST/1-108

MVRKLKHHEQKLLKKVDFLEWKQDQGHRDTQVMRTYHIQNREDYHKYNRI

CGDIRRLANKLSLLPPTDPFRRKHEQLLLDKLYAMGVLTTKSKISDLENK

VTVSAICR

>IOD3_RANCA/11-256

LLQQLLACCLLLPRFLLTVLLLWLLDFPCVRRRVIRGAKEEDPGAPERED

PPLCVSDTNRMCTLESLKAVWYGQKLDFFKSAHLGGGAPNTEVVTLEGQR

LCRILDFSKGHRPLVLNFGSCTCPPFMARLQAYQRLAAQRLDFADFLLVY

IEEAHPCDGWLSTDAAYQIPTHQCLQDRLRAAQLMLQGAPGCRVVADTMT

NASNAAYGAYFERLYVILDGKVVYQGGRGPEGYKIGELRNWLDQYQ

>IPT_RHOFA/20-252

PGVYAIVGATGIGKSAEASKLALSHSAPIVVADRIQCYSDLLVTSGRAFD

AKVEGLNRVWLDNRTIHQGNFDPDEAFDRLIKVLTSYVDRGEAVVMEGGS

ISLILRFAQTISNLPFPAVVNVMPIPDRQHYFAQQCARARQMLRGDSTGR

NLLTELAEAWVLGDQHNFIASVAGLDCVLDWCATHSVTPEELANRDLTTE

VLDELAASMGGRYVEHGVLQQEIFLRTFGAPGV

>ISCX_SHIFL/3-66

LKWTDSREIGEALYDAYPDLDPKTVRFTDMHQWICDLEDFDDDPQASNEK

ILEAILLVWLDEAE

>IST1_YEAST/18-147

MCIQRLRYAQEKQQAIAKQSRRQVAQLLLTNKEQKAHYRVETLIHDDIHI

ELLEILELYCELLLARVQVINDISTEEQLVKEHMDDGINEAIRSLIYAIL

FVDEVKELSQLKDLMAWKINVEFVNGVIAD

>ITR1_NICSY/31-94

KETWPELIGVPAKFAREIIQKENSKLTNVPSVLNGSPVTKDFRCERVRLF

VNVLDFVVQIPRVG

>KAD_MICLU/6-165

LMGPPGSGKGTQATRIADKLGIVPISTGDIFRHNVKSMTPLGVEAKRYID

NGDFVPDEVTNRMVADRIAQADAEHGFLLDGYPRTKGQVEALDAMLAEAG

QSLSAVVELEVPDEELVERLLKRAEIEGRADDTQEVIEHRLDLYHRETES

VIQEYVERGI

>KCNE1_HUMAN/1-129

MILSNTTAVTPFLTKLWQETVQQGGNMSGLARRSPRSSDGKLEALYVLMV

LGFFGFFTLGIMLSYIRSKKLEHSNDPFNVYIESDAWQEKDKAYVQARVL

ESYRSCYVVENHLAIEQPNTHLPETKPSP

>KCNN3_RAT/270-388

KLGHRRALFEKRKRLSDYALIFGMFGIVVMVIETELSWGLYSKDSMFSLA

LKCLISLSTIILLGLIIAYHTREVQLFVIDNGADDWRIAMTYERILYISL

EMLVCAIHPIPGEYKFFWT

>KCY_MYCTU/62-220

ETIASTVQMSLGYDPDGDSCYLAGEDVSVEIRGDAVTRAVSAVSSVPAVR

TRLVELQRTMAEGPGSIVVEGRDIGTVVFPDAPVKIFLTASAETRARRRN

AQNVAAGLADDYDGVLADVRRRDHLDSTRAVSPLQAAGDAVIVDTSDMTE

AEVVAHLLE

>KDKA_XYLFA/49-251

PAWWGGRAHAVSEGGRGSAWFVEASFGNAVLRQYRRGGMIAMLNRDRYFW

CGGHRTRSVLEFRLMRELISRGLPVPTPLAACYVRYGVQYRAAILMERLE

GVSSLAMCVRGNSKETHWEQIGRMISRFHREGLDHADLNAHNILLDQAGQ

CWLIDFDRGALRIPATKWRERNLARLLRSLLKIRGERSVDAVYRDFERLR

RAY

>KITH_SWPVK/4-175

GFIHLILGPMFSGKSTELIRLVNRYQIATYNCRVIKYSKDNRYGNDAVYT

HDKCYISAVSTDSLFDIKDTLDDVDIVGIDEGQFFNDIVEFCEYIANKGK

IVIVAALDGTYERKPFGNILNLIPLSEKVTKLNAICMICHRDASFSKRLS

DEKEIELIGGKEKYLSVCRSCY

>KM11_TRYBR/1-90

MATTYEEFAAKLDRLDAEFAKKMEEQNKRFFADKPDEATLSPEMKEHYEK

FEKMIQEHTDKFNKKMREHSEHFKAKFAELLEQQKNAQFP

>KRH2_DROME/24-270

QQQEQPQQSQSQNVPAKLLQHFQTNRIDSALWALRLLVIFFTVSYVLPIF

TSQQSAFSKVMLANAAISALRLHQRLPAFAFSREFLARLFAEDSCHYMMY

SLIFFNIRPSLLVLIPVLLYSVLHASSYSLKLLDLIGQNSWWGARFIISI

VEFQAANILKATAFCEIFIMPYAIVLAFMNHAGLMTPVIYYHYLVMRYSS

RRNPYPRNAFAELRITFEALAARSPPAFAKIIRGGIGFVNRLAPQLQ

>KTHY_RICPR/12-199

FEGVDGIGKSTQSKMLYEYLKSQKIPVILTREVGGTTVAEKMREILVNEE

LLPMSELLQAMAARYDHMARKIIPALKDGYIVICDRFIDSTACYQGLELE

NGIDLVYSLHKTLMPSLMPDITFFIDVEPHTAIKRVNARNMSNKFDIRSI

DFYKKIYTCFKELSNRFPERIKTIKASHLSPLEVHELI

>LAMA4_MOUSE/727-854

SKLFIEEANKTTAAVQQVTTPMANNLSNWSQNLQTFDSSAYNTAVDSARD

AVRNLTEVVPQLLDQLRTVEQKRPASNISASIQRIRELIAQTRSVASKIQ

VSMMFDGQSAVEVHPKVSVDDLKAFTSI

>LAMC1_MOUSE/48-282

CMPEFVNAAFNVTVVATNTCGTPPEEYCVQTGVTGVTKSCHLCDAGQQHL

QHGAAFLTDYNNQADTTWWQSQTMLAGVQYPNSINLTLHLGKAFDITYVR

LKFHTSRPESFAIYKRTREDGPWIPYQYYSGSCENTYSKANRGFIRTGGD

EQQALCTDEFSDISPLTGGNVAFSTLEGRPSAYNFDNSPVLQEWVTATDI

RVTLNRLNTFGDEVFNEPKVLKSYYYAISDFAVGG

>LAMC1_MOUSE/556-686

YFIAPVKFLGNQVLSYGQNLSFSFRVDRRDTRLSAEDLVLEGAGLRVSVP

LIAQGNSYPSETTVKYIFRLHEATDYPWRPALSPFEFQKLLNNLTSIKIR

GTYSERTAGYLDDVTLQSARPGPGVPATWVE

>LAPM5_MOUSE/28-260

HIVMSVLLFIEHVVEVARGKVSCRFFKMPYLRMADLLSSFLLIGVLFIIS

ISLLFGVVKNREKYLIPFLSLQIMDFLLCLLTLLGSYIELPAYLKLARPR

PGPSKVPLMTLQLLDFCLSILTLCSSYMEVPTYLNFKSMNHMNYLPSQEG

VPHSQFINMMLIFSVAFITVLILKVYMFKCVYTCYKFLKHMNSAMEDSSS

KMFLKVALPSYEEALSLPPKTPEGDPAPPPYSE

>LBD32_ARATH/5-105

RCAVCKILNETCAPMCIYAPHFPSNDASFKVIIQIFGAVNVCNILDNLEF

PEQREIAANCLRYAAEARIRNPISGCHDMILQYKNILNNVEQDIESAVNE

L

>LCN3_LACLA/490-669

GEKIAIVGKSGSGKSTLFNILLGLISYEGEVTYGYENLRQIIGVVSQNMN

LRKGSLIENIVSNNNSEELDIQKINDVLKDVNMLELVDSLPQKIFSQLFE

NGKNLSGGQIQRLLIAKSLLNNNKFIFWDEPFSSLDNQNRIHIYKNVLEN

PDYKSQTIIMISHHLDVLKYVDRVIYIDDK

>LECG_ARAHY/25-249

ETVSFNFNSFSEGNPAINFQGDVTVLSNGNIQLTNLNKVNSVGRVLYAMP

VRIWSSATGNVASFLTSFSFEMKDIKDYDPADGIIFFIAPEDTQIPAGSI

GGGTLGVSDTKGAGHFVGVEFDTYSNSEYNDPPTDHVGIDVNSVDSVKTV

PWNSVSGAVVKVTVIYDSSTKTLSVAVTNDNGDITTIAQVVDLKAKLPER

VKFGFSASGSLGGRQIHLIRSWSFT

>LEF5_NPVOP/24-262

PRALFTVFGAFRASKDYAKLIEFLTNNFACYVKNKTFNFAGTGHLFHSLY

AFVPNVSELVKERKQIRLQIDCVMRLFKNTTNDFKMYVELFAFIDAHGGA

ECPCLLLQQSKLNAVSFVENLNCKLFDIKPPKFKKEPFDSILSKYSLNYK

ALCFKKKEKCTVGCVTKRQKKMKRRQLLSDRVIYLHNKNDVLDERTLLHG

PSGTSLAPCLHRYATVERQTRAGDEMVSFIRYCELCQMR

>LIMA_RHOER/21-144

TPDEKIVLEFMDALTSNDAAKLIEYFAEDTMYQNMPLPPAYGRDAVEQTL

AGLFTVMSIDAVETFHIGSSNGLVYTERVDVLRALPTGKSYNLSILGVFQ

LTEGKITGWRDYFDLREFEEAVDL

>LIN2_CAEEL/371-425

KVLGSLDAINSLLDPNSYKPGSTTFQKIHDDGSVRNLLRLYDKIKALPCE

PVVTE

>LOXL3_MOUSE/530-733

SDLLLHSALVQETAYIEDRPLHMLYCAAEENCLASSARSANWPYGHRRLL

RFSSQIHNLGRADFRPKAGRHSWVWHECHGHYHSMDIFTHYDILTPNGTK

VAEGHKASFCLEDTECQEDVSKRYECANFGEQGITVGCWDLYRHDIDCQW

IDITDVKPGNYILQVVINPNFEVAESDFTNNAMKCNCKYDGHRIWVHNCH

IGDA

>LYG_ANSAN/50-170

TIIKKVGEKLCVEPAVIAGIISRESHAGKVLKNGWGDRGNGFGLMQVDKR

SHKPQGTWNGEVHITQGTTILINFIKTIQKKFPSWTKDQQLKGGISAYNA

GAGNVRSYARMDIGTTHDDYA

>LYS4_YEAST/484-620

NPVVEEEVNAQTEAPKQSVEILEGFPREFSGELVLCDADNINTDGIYPGK

YTYQDDVPKEKMAQVCMENYDAEFRTKVHPGDIVVSGFNFGTGSSREQAA

TALLAKGINLVVSGSFGNIFSRNSINNALLTLEIPAL

>LYTR_STRMU/146-243

DRSIVLKMPDIVAASIEDGELTVSTKNTSYTIKKTLNWFKTRAKTNYFLQ

IHRNTVVNLEMIQEIQPWFNHTLLLVMVNGEKFPVGRSYMKELNAHLT

>M1_INBSI/158-248

SHRAHSRAARSSVPGVRREMQMVSAMNTAKTMNGMGKGEDVQKLAEELQS

NIGVLRSLGASQKNGEGIAKDVMEVLKQSSMGNSALVKKYL

>M1_INBSI/2-157

SLFGDTIAYLLSLTEDGEGKAELAEKLHCWFGGKEFDLDSALEWIKNKRC

LTDIQKALIGASICFLKPKDQERKRRFITEPLSGMGTTATKKKGLILAER

KMRRCVSFHEAFEIAEGHESSALLYCLMVMYLNRGNYSMQVKLGTLCALC

EKQASH

>M2H1_BOMMX/1-141

MNFKYIVAVSFLIASAYARSEENDEQSLSQRDVLEEESLREIRGIGTKIL

GGVKTALKGALKELASTYVNGKRTAEDHEVMKRLEAVMRDLDSLDYPEEA

AERETRGFNQEEIANLFTKKEKRILGPVISTIGGVLGGLLK

>M2_IAZI1/1-97

MSLPTEVETPTRNEWGCRCNDSSDHITIAAKFIGILHLILWILDRLFFKC

IYRRLKYGPKRGPSTEGVPDSMREEYRQKQQNAADVDDGHFVNIELE

>MANC7_ECOLI/306-459

ETENSYIYTESGLVATIGIQDLVIIHTKDSLLVSRRDSVQNVKNIVQHLD

LSGRKEHKEHREVFKSWGRCDSIDSSEKYHYQVKRITVNPSENYRCNYII

TVRNIGVVVMGIAKLTVAEEIKILKENESVYIPAGIKHSLKILDNTTCVN

RSLD

>MANS1_HUMAN/24-117

SASQNCLKKSLEDVVIDIQSSLSKGIRGNEPVYTSTQEDCINSCCSTKNI

SGDKACNLMIFDTRKTARQPNCYLFFCPNEEACPLKPAKGLMSY

>MBP1_YEAST/24-107

SIMKRKKDDWVNATHILKAANFAKAKRTRILEKEVLKETHEKVQGGFGKY

QGTWVPLNIAKQLAEKFSVYDQLKPLFDFTQTDG

>MCP3_ECOLI/47-185

DRDQRDVTAEIEIRTGLANSSDFLRSARINMIQAGAASRIAEMEAMKRNI

AQAESEIKQSQQGYRAYQNRPVKTPADEALDTELNQRFQAYITGMQPMLK

YAKNGMFEAIINHESEQIRPLDNAYTDILNKAVKIRSTR

>MCP7_SCHPO/15-204

LEAIFHDSKDFFQLKEVEKLGSKKQIVLQTVKDVLQSLVDDNIVKTEKIG

TSNYYWSFPSDAKRSRESVLGSLQAQLDDLKQKSKTLDENISFEKSKRDN

EGTENDANQYTLELLHAKESELKLLKTQLSNLNHCNPETFELKNENTKKY

MEAANLWTDQIHTLIAFCRDMGADTNQIREYCSIPEDLDD

>MDH_THETH/156-325

TRLDHNRAKAQLAKKTGTGVDRIRRMTVWGNHSSTMFPDLFHAEVDGRPA

LELVDMEWYEKVFIPTVAQRGAAIIQARGASSAASAANAAIEHIRDWALG

TPEGDWVSMAVPSQGEYGIPEGIVYSFPVTAKDGAYRVVEGLEINEFARK

RMEITAQELLDEMEQVKALG

>MDL3_PRUSE/386-537

IVNKVPGPLSHGTVTLNSSSDVRVGPNVKFNYYSNLTDLSHCVSGMKKLG

EVLSTDALEPYKVEDLPGIDGFNILGIPLPENQTDDAAFETFCRESVASY

WHYHGGCLVGKVLDDGFRVTGINALRVVDGSTFPSTPASHPQGFYLMLGR

YM

>MECA_BACPF/1-212

MDIERVNDTTIKFFITYKDIEDRGFDRDEIWYNRERGEELFFEMMNEAND

RDEFELDGPLWIQVHALDKGLEIVVTRGQVSDGNVKLEIPVSQDKENTDE

NIVDLMTGHSSEDDEGIDTDQLEIVIGFNDFEDIISLSHNFFIDDLENEL

YHFEGRYYLHVLFNDDQYNEDEQDDMLSQMLEYGYETDLSIHRMQEYGKE

IIGEYALKHLRG

>MER3_EUPOC/1-151

MKAIFIILAILMVTQAFKMTSKVNTKLQSQIQSKFQSKNKLASTFQTSSQ

LKYYCWEEPYTSSITGCSTSLACYEASDCSVTGNDQDKCNNVGQNMIDKF

FELWGVCINDYETCLQYVDRAWIHYSDSEFCGCTNPEQESAFRDAMDCLQ

F

>METJ_SALTY/1-104

AEWSGEYISPYAEHGKKSEQVKKITVSIPLKVLKILTDERTRRQVNNLRH

ATNSELLCEAFLHAFTGQPLPDDADLRKERSDEIPEAAKEIMRELGIDPE

TWEY

>METK_YEAST/239-376

IGGPQGDAGLTGRKIIVDAYGGASSVGGGAFSGKDYSKVDRSAAYAARWV

AKSLVAAGLCKRVQVQFSYAIGIAEPLSLHVDTYGTATKSDEEIIDIISK

NFDLRPGVLVKELDLARPIYLPTASYGHFTNQEYPWEK

>MEU13_SCHPO/16-185

EAEKLVYEYLRKTNRPYSATDVSANLKNVVSKQVAQKALEQLRDTGLIHG

KLYGKQSVFVCLQDDLAAATPEELAEMEKQIQELKDEVSVVKTLYKEKCI

ELQALNNSLSPAEIREKIQSIDKEIEETSSKLESLRNGTVKQISKEAMQK

TDKNYDFAKKGFSNRKKMFY

>MFN1_HUMAN/567-737

TTPATPDNASQEELMITLVTGLASVTSRTSMGIIIVGGVIWKTIGWKLLS

VSLTMYGALYLYERLSWTTHAKERAFKQQFVNYATEKLRMIVSSTSANCS

HQVKQQIATTFARLCQQVDITQKQLEEEIARLPKEIDQLEKIQNNSKLLR

NKAVQLENELENFTKQFLPSS

>MGF_CHICK/46-196

TRKIRGDVAALQRAVCDTFQLCTEEELQLVQPDPHLVQAPLDQCHKRGFQ

AEVCFTQIRAGLHAYHDSLGAVLRLLPNHTTLVETLQLDAANLSSNIQQQ

MEDLGLDTVTLPAEQRSPPPTFSGPFQQQVGGFFILANFQRFLETAYRAL

R

>MGN_SCHPO/3-145

DFYVRYYSGHHGRFGHEFLEFDYHSDGLARYANNSNYRNDSLIRKEMFVS

ELVLKEVQRIVDDSEIIKESDESWPPENKDGKQELEIRMNGKHIMFETCK

LGSLADVQNSDDPEGLKVFYYLIQDLKALCFSLISLNFKLRPV

>MGP3_MYCPN/490-715

MGSDRVPSLWYWVVGEDQESGKATWWAKTELNWGTDKQKQFVENQLGFKD

DSNSDSKNSNLKAQGLTQPAYLIAGLDVVADHLVFAAFKAGAVGYDMTTD

SSASTYNQALAWSTTAGLDSDGGYKALVENTAGLNGPINGLFTLLDTFAY

VTPVSGMKGGSQNNEEVQTTYPVKSDQKATAKIASLINASPLNSYGDDGV

TVFDALGLNFNFKLNEERLPSRTDQL

>MGSA_RHIME/18-112

DLAAFAKANEAVLSKWKIVATGTTGGRVLDVCPALDIVRLKSGPLGGDQQ

IGALIATGDVDCLIFFVDPLTAMPHDVDVKALMRLAIVYDIPMAL

>MIAA_STRCO/42-291

MQLYRGMDIGTAKLTPEERGGVPHHLLDIWDVTVTASVAEYQRLARERID

ALLAEGRWPILVGGSGLYVRGAVDNLEFPGTDPGIRARLEEELELRGPGA

LHARLAVADPEAARAILPSNGRRIVRALEVIEITGRPFTANLPGHDSVYD

TVQIGVDVARPELHERIALRVDRMWEAGLVDEVRALEAQGLREGRTASRA

LGYQQVLAALAGECTLDEARAETVRATKRFARRQDSWFRRDPRVHWLSGG

>MIH_PROCL/2-74

YVFEECPGVMGNRAVHGKVTRVCEDCYNVFRDTDVLAGCRKGCFSSEMFK

LCLLAMERVEEFPDFKRWIGILN

>MINC_PASMU/113-220

CLPVKIIHQHVASKQVIYAKNSDLIIHGNVEPGAEVAADGNVHIYGKLLG

RAMAGVNNNVGSIYTQYLDAEFIAVSSRFLYKDNLPHEYQHEAVRIFADK

DKLRFHFL

>MINC_PSEAE/7-113

LDQDPVFQLKGSMLAVTILELAHNDLARLERQLADKVAQAPNFFRDTPLV

MALDKLPEGEGRLDLPALLEVCRRHGLRTLAIRAGREEDIAAAQALDLPV

LPPSGAR

>MINE_HELPJ/1-77

MSLFDFFKNKGSAATATDRLKLILAKERTLNLPYMEEMRKEIIAVIQKYT

KSSDIHFKTLDGNQSVETIEVEIILPK

>MMPS1_MYCTU/4-142

VAKRFWIPMVIVIVVAVAAVTVSRLHSVFGSHQHAPDTGNLDPIIAFYPK

HVLYEVFGPPGTVASINYLDADAQPHEVVNAAVPWSFTIVTTLTAVVANV

VARGDGASLGCRITVNEVIREERIVNAYHAHTSCLVKSA

>MNTR_BACSU/1-61

MTTPSMEDYIEQIYMLIEEKGYARVSDIAEALAVHPSSVTKMVQKLDKDE

YLIYEKYRGLV

>MNTR_BACSU/63-133

TSKGKKIGKRLVYRHELLDQFLRIIGVDEEKIYNDVEGIEHHLSWNSIDR

IGDLVQYFEEDDARKKDLKSI

>MOAA_AERPE/183-313

FDLQVIEVHPAGRGRKVLSSFRRPIDVVEERLSSMAVAVETGRLHNRRVY

RLPSGVRVYLVDPVENPVFCMGCYRVRLTWDGRLLPCIYWKGPYPSVAEA

LKRGGSREEKVWRVMKILLEANALRRPTYLF

>MOAC_AQUAE/3-136

TVDITTKIETLREAKAYGRIRLKPETVKLIKENKVPKGNLVEATKLSGIF

GAKKTGELLPFCHPIPLDFVALEVKVNEDNLEVFSTVRGIARTGYEMEAL

TAVTTALLNVYDMCKALDDSMVIEEVKLLEKSGG

>MOCA_RHIME/4-121

FRLGLVGAGRMGQVHVRAAAESSLVEIAAVADPIAASRLNLAGNGIKTYE

TAGDMIEAGEVDGVLIATPSNTHVDTVADIAARGLPILCEKPCGVTAEEA

RKAADVAERYKVHLQIGY

>MRE11_COPCI/271-495

RIIPEPVAGKNYYITQPGSSVATSLADGEAIEKHVALLEIKGKEFQLTPI

PLRTVRPFVISEVVLEDAAEEEGLDVNDQMEITKYLKQKVNDLIDQAQAL

WEERNARSIEAGDEEIPPMLPLVRLKVDTTNVTQTSNPIRFGQEFQGRVA

NPRDLLVFHRSKKAGKRGAGKVDIDQPELSIDDPDLTVSEKLAKVRVKTL

VREYLAAQELQLLGENGMSDAIQMF

>MRR_ECOLI/175-279

VLDVLHRLGYGGHRDDLQRVGGTGDGGIDGVISLDKLGLEKVYVQAKRWQ

NTVGRPELQAFYGALAGQKAKRGVFITTSGFTSQARDFAQSVEGMVLVDG

ERLVH

>MS2A_DROMA/1-250

MNQILLCSQILLLFFTVANCDGEHQLDSSVDLKSAVLKNVAPKNVATQAE

IVKDDVALKSGKKGDYVMDIEVSDMPLDDYPINNSKSRKNSSTLPSPILT

DKLNQGSNQIALKALKHRLVMEQNNNLFLRNHSVSLMNEIEARKTDIIQA

RQLNIDLELELESLKRKLSEMNVQNARKSTKSCKKRPSKDIAPPVNQLQE

VIVKNTYRNKYLTLLTQLAQKINYEIANVNNPATDVPTGKSPSEGNPSTT

>MSA2_PLAFF/105-264

SASTSTTNNGESQTTTPTAADTPTATESISPSPPITTTESSKFWQCTNKT

DGKGEESEKQNELNESTEEGPKAPQEPQTAENENPAAPENKGTGQHGHMH

GSRNNHPQNTSDSQKECTDGNKENCGAATSLLSNSSNIASINKFVVLISA

TLVLSFAIFI

>MSCL_SYNY3/12-137

FWRDFKDFILRGNVVDLAVAVVIGGAFTSIVNAFVAWLMAVLLQPVLDQA

GVSQLQDLPLGLGELVIAIINFLIIAFVIFLIIKAIEKMQRKKAVEEEIV

AEAQPDPVLEAQTNLTDSINRLITTL

>MSF1_YEAST/15-174

DQVTAANWKKYPNEISTHVIAVDVLRRELKDQGKVLVTERLITVKQGVPK

WIMMMLGGTNMSHVREVSVVDLNKKSLTMRSCNLTMCNLLKVYETVTYSP

HPDDSANKTLFQQEAQITAYGSIRKLCNKMEDWSVQRFCENAKKGKMGFD

AVLQVFSENW

>MSH4_CANAL/512-742

YTCPEFAKEVTIMRSLHPILGGNNSNFVANNYSCNHELSRIHVITGANMS

GKSVYLRQIAYLVIMAQMGCFVPAEYARMRIFNSLYSRISSDNVDINASS

FSKEMSETAVILNDSDGDSLILIDELGRGSSLTDGFSICLAILEDLICKE

ATVITTTHFRDIAQVLANKSCVVTAHMQTVETNGQLEMKYNLVLGRNDIV

GYGIRFAEVSNLLPQELIEDSKVVANILRSR

>MSMB_RAT/1-113

MKARLGSLLVLATLVTASNAACSIQRLKRLPNEKSDECTDVDGGKHVLNT

YWQKNCEWCFCEKTAITCCTKTLIPVSYDKKRCQRQFHSENCTYSVVERT

NPGKTCPVNGWTI

>MSRAB_HAEIN/43-196

REIYLAGGCFWGMEAYMERIHGVKDAISGYANGNTEKTSYQMIGLTDHAE

TVKVTYDANQISLDKLLKYYFKVIDPTSVNKQGNDRGRQYRTGIYYQDGA

DKAVIGQALAQLQTKYKKPVQIEVQPLKNYIVAEEYHQDYLKKNPNGYCH

IDIT

>MSS4_SCHPO/11-100

CPSVVFNNKRPDVVKRPTMSAMLHSETQEDLETDDFFLLKDPFAFDNVSV

SKPLANNYKLLACADCEKGPLGYYDSKNNEYLLLCSLEKN

>MTFR1_MOUSE/20-249

MQSVLWSGKPYGSSRSIVRKIGTNLSLIQCPRVQFQLTSHATEWSPAHSG

EDAVASFADVGLVATEEGECSIRLRAEVSSKPPHEDDPPCFEKPPSRHTS

FPSLSQDKPSPERTLASEEALQKISALENELAALRAQIAKIVTLQEQQSP

SAGCLDSSTSVTVAPPPPPPPPPPPLPLVLHQSTSALDLIKERREQRLSA

GKTLATGHPKKPDMPNMLEILKDMNSVKLR

>MTRD_METJA/1-219

MDIVSAIVPLIEMTIAGAIINASVHFIPVGGAPAAMATSTGVGTGTTQLA

AGAGFTGLMGAAVMASNVGLSPIGMALIMISGAVSSMIMLGVTMLIGQLI

YVFGVGVVPAADKCEIDPITKDPQKPYVTPGTTGHGVPTVCFVSGLIGAA

LGGIGGALAYIALRKLGLDPGVAGMLAVGFFFINAVLASYNIGGTIEGFH

DPKFKKMPNGVIASTVASL

>MTRE_METFE/8-255

LGILALTTASAIIGQTIEDVETNIGSQSNPNSQVQLAPQMGNLHRFFNKA

IAGEPFAYCTFCGVSGAITVATLYLHLPAVIALAIGAAITTLIWLAYSTT

AYLGRVSGSATFNQPVFLDMLTENLGPIAGHAFIVFFCMTGVAYLMTLPV

KGFAHPFPIPVIGMIWGMTIGAIGSAVGDVYYGAEAEFVHKKFGGGIPVA

SHGDITRKGVLGARSPMEVGNFTVKYGSPITGMAFGLIVLSITRGVYN

>MTRG_METJA/4-73

DEKLPQVIMDPADYEALKKRLDELEKKVENTNAELFQLAGKKVGRDIGIL

YGLVIGIILSYILPALIKII

>MTXA_METBF/1-168

MKKIAEAWPIVKGDYTVGNPESRIAVVTLASQINSLPEAALWGSSKTENL

GVEKIIINTISNSNIRYILICGKESRGHLAGHSLLAIHANGIDEKGRIVG

SEGAIPFIENISREAVKRFQQQVVLLDRIGLTNLEEIMKIVREYKDQGEV

YPE
