# Supplementary material for: A machine learning approach for the identification of odorant binding proteins from sequence-derived properties
Source: BMC Bioinformatics. 2007 Sep 19;8:351. doi: 10.1186/1471-2105-8-351 (PMC2216042; doi:10.1186/1471-2105-8-351)
Supplement: Additional file 3 — Independent testing dataset. This data provides 414 protein sequences that are used for testing. [file 1471-2105-8-351-S3.doc]

>108870204 |Juvenile hormone binding protein

KIDAIYNLKGNVLLLPLVGDGDVTMVLKDVKTSVVTKFGVRPLPEDAIFI

EEMKVTFLVGGMRIHLDNLFQGNQVLGASLNLFLNQNANEVIAELRTDLE

YGLADIFTGLWNELFNKLPLKLWMV

>108870205 |Hypothetical proteins

YVEGRYVVDGRILLLPVKGAGKFNGNFTQGYGNVRIKGDRKVINGKNHLS

LAKLDIKIKVGDGKIKLENLFGGDRVLGEIINQTINQNFSLLSHELIPLI

EKALQRIFKRTGNKILERFPEEKVPLE

>108870207 |Hypothetical proteins

LIHAKYTSSGVLIIIPASGAGDFDAVFDGVIANVRGRVSFSEKPTGTHMR

VENLDLNLAIKKPRLSVSKIFNNNRILTEATNLFLKENGGEVLKALQPQL

QKKLSAEFAGISNQLLDNVPLNNFIID

>108870553 |Odorant binding protein

VTPRRDAEYPPPELLQALKPLRDICQKKTGVSDEAILEFSDGKVHEDEKL

KCYMNCLFHEAKVVDDTGHVHLEKLHDALPDSMRDIAMHMGKRCLYPEGE

NLCEKAFWLHKCWKESDPK

>108875704 |Hypothetical proteins

RIEGNYHMQGRILVIPLNGHGKCWFEPSDMDIIMKTTTTLYEQNNHVFYN

VTATKVDYTISGLKLHMGNLFEGVKVLEDSTNQYLNDNWRPVSEALKPII

AKTIEDILLAIMRNIFFQIPAEYFVA

>108876021 |Odorant binding protein

QEPRRDANYPPPELLEKMKPMHDACVAETGASEDAIKRFSDQEIHEDDNL

KCYMNCLFHKAGVVNDNGEFHYVKIQDFLPESMHLITLNWFKRCLYPQGD

NLCEKAFWLNKCWKERDPV

>108879356 |Hypothetical proteins

ITEGKYKGEGRFNAIKVVSKGYFNVTTSDVSATWRISGRTTQRQGEEYLL

IDKFDMTPEVGDMKVYATGLFPDPGLNQVALDFVNQYWPSLYKQMLPETR

QSWEPMMLDIVNKMFNRVPYRRLLPK

>108883363 |Hypothetical proteins

DVSGKYSLNMNILVLRIAGKGDIKAVLNDTKAILKLEYFSENVGGKQLAR

FRPIDLRLKFDKASFYLSNLFNGDPTLEKVGNDAINENPMVLLDEVKPSF

EENLSIKFTELANSLVKDAELSEIFPE

>108883365 |Hypothetical proteins

IVNGQYTLDMKVLVLRISGQGDFDLILDNTIANMRLKYYLKPGPDGKEYV

QFQPIQVKLRFDKGKFNLQNLFNGDPTLGQIGNSAINEDPHVLLDEVKPA

FEESLAKQFTAMANSAVSGATELDILPL

>108883368 |Hypothetical proteins

SLWGMSSPRNDDTIASLKVQYRLAPEGDKNFVRFDPIDLKLKFPKAKFYF

TGLFNGDPALEEFGNQAINDNPNLILDEVKPSFEKNLGRVFTDISNSVVE

GAEEFELL

>108883369 |Hypothetical proteins

YVRGKYELNMNILLLKIAGKGDFNLTLGDTQVNMMVQYYLEPKGGKNYLK

FRPVEIKIKFDKALFYLKNLFGGDPTLEEIGNQAINANPHLLLDEVKPAI

KDKLQESLTYISNSVVDGAEEDELLPR

>108883370 |Hypothetical proteins

VLKGKYNMNLRLGLLLVDGDGDSTLDLTDVKLLVKMNYFFAENSNGRKTM

QFHPIDVKIKYAGTAKFDMTNLLKDKPRLGQAANEAINESPELILEKTKH

PVEQFFSKLFTTIANGLMNDAEEEEAFPV

>108883371 |Hypothetical proteins

SFRGRYDLKIKLLLLNIAGTGDLKGVLENSRARVKLFCERYDKDGKAFMR

AKRLAVKIQIEKGQFDMKDLFNGDATLSQVGNQFINDNSRLFLDELTPGL

ERSLSETFKNTANEILKLATMDDIFPA

>108883913 |Hypothetical proteins

IMDQPKFVAPSRLLENQIFLECGTMLGFSRQRVWDVLYKGDFTHPEISCL

VRCFLIRSGLYDDKSGLHLERFYVACGGYDDAFYHNVTKCIANVEAAGLC

DKCTRAQRLALECVGSQY

>108883914 |Hypothetical proteins

IMDQPKFVAPSRLLENQIFLECGTMLGFSRQRVWEVLYKGEFTLPEISCL

VRCFLIRSGLYDDKSGLNLERFYVACGGYDDAFYHNVTKCIANVEAAGLC

DKCTRAQRLALECVGSQY

>108884243 |Hypothetical proteins

KTTGKYKAQGKALSFIPFNRSGNFGFNFNGLSLAGSIKVALNGDKLLVEE

FKLYPTVKSVNSKFEHVFFLPLTNLIFNKIVESVVPKYLRENQEDVSLFL

ESYIKPEVNELLGDYTLEDLMGL

>109473603 |Probasin

GNFNTIYLAGKFVRKTAENSQARMLMREVKYFNKYRVMSMMFYIRKNGSC

QLHTVWADKPQYKYYSMTWNLNLIKCPKKNGTDYIPGIWYFHDPLDHVPN

MYTHPIHFASKTTILFEAYFFDHRNILHKFTGALATGANITEKMQEEYMK

LNQNLKIPTENIENVYETDV

>109474987 |Vertebrate odorant binding proteins

GDWFSIVVASNKREKIEENGSMRVFMQHIDVLENSLGFKFRIKENGECRE

LYLVAYKTPEDGEYFVEYDGGNTFTILKTDYDRYVMFHLVNFKNGETFQL

MELYGRTKDLSSDFKKKFAKLCEAHGITRDNIIDLTKTDR

>109483261 |Vertebrate odorant binding proteins

GKHILLLPLGLSLLMSSLLALQCFRCISFDSTGFCYVGRHICQTYPDEIC

AWVVVTTRDGKFVYGNQSCAECNATTVEHGSLIVSTNC

>110596817 |Vertebrate odorant binding proteins

GKWYEIARIDNRFEKGIEQATAVYSLAEDGSVKVLNSGYKVKKRQWKTVT

GRGVFIDDTSKGALKVSFFGPFYASYNVIALDRANYSWAVVCGSNRSYFW

ILSRTPLMERELLEELVGKAGSMGFDTTKIHTVTQQQT

>110759398 |Juvenile hormone binding protein

LKGGYEFKGSTFNSTINNRGTFTLQLYDLVQTTTVIAKPGSKVYVRVELN

SIRDMKLHVTNLFFGIKLIENFIDIVINNTWKQFFKISTPIINELISDGY

FELFDTTFRNFPFEKIIKP

>110766380 |Juvenile hormone binding protein

IIKGEYDVDGQLLLLRIRGSGALNGQFNNCKALVKLQMEMTKGKDGQNYL

KLADLQTKIFVGSGSSLKLDNLFGGDPVLGDAINVAINSNFDSFLKEITP

IIENAISNTFTDISNSILKQFPYEKLFPE

>110766385 |Juvenile hormone binding protein

HMNGNYDVQGRLLLLPLNGAGNFKGNFTDTKVDVQAQGKEVTDAKGVQKI

EIDKLVTKIRVENGNIELKAPPNHSAAAEAAATFFNSNPRLVLDIASPII

EDTAATVSRALVARALNVLTKQELLP

>110766387 |Juvenile hormone binding protein

EMVSNYVMDGRIMMLSITGNGLGHGNFTDIDVIVTIQMERYLNERTGSIH

QSVDDIYVDFEIGHATVHLDNLFDGDEMLSGAMNLFLNDNWSTIIAEIRP

KLEETIAMLIMKFTNTIFSLFPENVLLP

>112031518 |Chemosensory protein

VDVDNVLKNEKLVKRYIDCTLERGRCEQNGRDLKVMIPRVLNEGCSGCTP

KQVENSNRIINFMKTNHPGDWAAIETKYK

>112031531 |Chemosensory protein

AAVEAAIRNPRYMRRQINCLLNESPCDNIGRTMRQLVPALIKGQCPGCSP

QQHQQAMKVMNVVSQQYPQEYSRIYYTYN

>112032057 |Chemosensory protein

PFDNINIEEIFENRRLLLGYINCILERGNCTRAGKDLKSSLKNVLEENCD

KCSEDQRKSIIKVINYLVSSEPESWNQLKSKYDPEGKYLIKYE

>112032227 |Chemosensory protein

TDKWDNINVDEILESNRLLKGYVDCLLGKGRCTPDGKALKETLPDALEHE

CVKCTGKQKSGADKVIRHLVNKRPDLWKELAVKYDPDNIYQARYK

>112032244 |Chemosensory protein

SSRYDDFDVKPLVENDRILQSYTNCFLDKGPCTPDAKEFKKVIPEALETT

CGKCSPKQKQLIKTVIKAVIERHPEAWEELVNKYDKDRKFRPSFD

>112032265 |Chemosensory protein

TTQYDEVDIKEIMGNERLLVAYIGCLLDKNPCTPEGKELKRNIPDALQSD

CSKCSDKQRENADAWIEFMIDNRPEDWTKLEER

>112032318 |Chemosensory protein

DSRYDYYDIDHLVQNPRLLKKYLDCFLGKGPCTPIGRLFKQVMPEVITTA

CAKCTPTQKRFARKTFNAFRRYFPETLMELRRKFDPESKYYDAFE

>11277082 |Vertebrate odorant binding proteins

GEWRIIYAAADNKDKIVEGGPLRNYYRRIECINDCESLSITFYLKDQGTC

LLLTEVAKRQEGYVYVLEFYGTNTLEVIHVSENMLVTYAENYDGERITKM

TEGLAKGTSFTPEELEKYQQLNSERGVPNENIENLIKTDN

>11277083 |Vertebrate odorant binding proteins

GEWRIIYAAADNKDKIVEGGPLRNYYRRIECINDCESLSITFYLKDQGTC

LLLTEVAKRQEGYVYVLEFDGTNTLEVIHVSENMLVTYVENYDGERITKM

TEGLAKGTSFTPEELEKYQQLNSERGVPNENIENLIKTDN

>112820887 |Odorant binding protein

VSVMKDVTLGFGQALDKCRQESDLTEEKMEEFFHFWRDDFKFEHRELGCA

IQCMSRHFNLLTDSSRMHHDNTEQFIQSFPNGEVLARQMVSLIHGCEKQF

DHEEDHCWRILHVAECFKHACVA

>112820889 |Odorant binding protein

DVSVMKDVTLGFGQALDKCRQESDLTEEKMEEFFHFWRDDFKFEHRELGC

AIQCMSRHFNLLTDSSRMHHVNTEEFIQSFPNGEVLARQMVALIHGCEKQ

FDHEDDHCWRILHVAECFKHACVA

>112983042 |Chemosensory protein

DKKYDNFNVDEIIDNPRLLKAYTFCFNDKGKCTAEGNDFKKWIPESLQTS

CGKCSEKQKYLVAKFVHAIKDKMPDEFDILRKLHDPKGEYTENLD

>112983048 |Chemosensory protein

SSQYDNFDVEQLVGNLRLLKNYAKCFLDQGPCTAEGTEFKKRIPEALRTK

CAKCNPKQRHLIRTVVKAFQTKLPDLWEELAIKEDPKGQYKHEFT

>112983050 |Chemosensory protein

TDKYDNIDVDEILENRKLLVPYIKCVLDEGRCTPDGKELKAHIKDGMQTA

CAKCTDKQKVSARKIVKHIKQHEADYWEQMKAKYDPKDEFKEIYE

>112983052 |Chemosensory protein

SSENDDLDIEALVGNIDSLKAFIGCFLETSPCDAVSGDFKKDIPEAVAEA

CGKCTPAQKHLFKRFLEVVKDKLPQEYEAFKTKYDPQGKHF

>112983054 |Chemosensory protein

TDKYDTVDLDQLISNRRLLIPYVHCILEKGQCTAEGKELKSHIKEALETN

CAKCTKAQKGGTEKMIGHLINHEAEFWEELKAKYDPTNEFTKKYE

>112983058 |Chemosensory protein

TDRYDNVNLDEVLSNSRLLQPYIKCILDKDRCAPDAKELKEHIREALETE

CAKCTEAQKKGTRRVIGHLINNESKSWNELTAKYDPENKFTAKYE

>112983082 |Juvenile hormone binding protein

LKRAQTNNWPIKEHNGLKYVEAPGGYKFYIVDKPQPVDKDPVVKVSLASS

NLAKSIAYWNGLLTLKLYEKTDKTALLGYSDDQAKLELVDIGGPINRAKA

YGRIAFSCPFDERPVIDKKIQDA

>112983094 |Chemosensory protein

TDKYDKINLQEILENKRLLESYMDCVLGKGKCTPEGKELKDHLQEALETG

CEKCTEAQEKGAETSIDYLIKNELEIWKELTAHFDPDGKWRKKYE

>112983166 |Hypothetical proteins

YLKGKYKASGKLLILPITGDGDTTIKLKNLRIQMIYPFNLVKNSEGKDVI

DLSSYRYSYDVKDNAHFHMTNLFNGNKQLGDVMLTFMNQNWKALTQEFGK

PLLEIPMEMVYNTVKTYLKSQPLEDIANL

>112983172 |Hypothetical proteins

IKGHYTAGGSILVLPITGDGQMKLKLKNMQIHFYIEYDVEKAEDGKDHIV

LKKYDFDFDVRENAHFELSNLFNGNKELSKIIHSFLNENWKQVVTEFGRP

IMDATAKKFFKNINIFFEKNSLEDIAIL

>112983174 |Hypothetical proteins

TLVGEYTLGGQLLILPIEGTGKYRIRIRDIIMKIILDVEERIVEGDRYWH

VSDWKHSAEDVSKVEYQFQNLFNGNRDLAKTIHDFANSNWREIFQEVAPP

MVKAIVSKIIHETFKLFDKVPIKDLALE

>112983410 |Hypothetical proteins

SARARYRSSGVLLLVRASGGGEYWGEYHGVKAKVYFRGAPYERDGRTYLK

LQQLKLDFSVKDIKMGVENLDNSNAVLQAALNLFISTNAQELLKEMKPEL

KRDLADKMSRYLDHILQHIPYDELVVD

>112984426 |Odorant binding protein

LQGALTDVYVMKDVTLGFGQALEQCREESQLTEEKMEEFFHFWNDDFKFE

HRELGCAIQCMSRHFNLLTDSSRMHHENTDKFIKSFPNGEILSQKMIDMI

HTCEKTFDSEPDHCWRILRVAECFKDACNK

>112984474 |Chemosensory protein

KYEPIDDSFDASEVLSNERLLKSYTKCLLNQGPCTAELKKIKDKIPEALE

THCAKCTDKQKQMAKQLAQGIKKTHPELWDEFITFYDPQGKYQTSFK

>113205954 |Juvenile hormone binding protein

DVIAPNDAGVVIRFKNLNITGLKNQQISDFQMDTKAKTVLLKTKADLHIV

GDIVIELTEQSKSFTGLYTADTNVIGAVRYGYNLKNDDNGVQHFEVQPET

FTCESIGEP

>11387218 |Vomeromodulin

SQPYATGETKLFISHASKILNSKLVPDVKLTRSEHSVVPPETKEEVEGIM

AEVTRKAWSRFNELYKKMSIPDGVSSNTLTNSDVKLLRSNDL

>113947522 |Vertebrate odorant binding proteins

GKWYEIARLDHSFERGLSQVSAEYSLKDDGGVMVINSGFSAAKNEWKEAE

GKAYFVNGDSEGYLKVSFFGPFYGSYVVFELDHENYQYAFISGPDTDYLW

LLAKTPTVPPEVLQKFVEMSKARGFDTDSLIYVQQESA

>113951689 |Chemosensory protein

TTKYDNVDLDEIIKSDRLLKNYVNCLLEKGKCTPDGAELKRHLPDALHTE

CSKCSETQKNGSKKIMRHLIDHKRDWWNELEEKYDKEGEYRKKYE

>113951691 |Chemosensory protein

TTKYDNIDLNQILKSDRLLKNYVNCLLDRGKCSPDGQELKNNLADALQTS

CSKCSQRQKDGSRTIIRYLIKNKRDWWNELEAKYDPTGIYKNKYA

>113951693 |Chemosensory protein

TTKYDNIDLEEILKSDRLLKNYFNCLMERGTCSPDGEELKKALPDALHSG

CSKCTEKQKEGSRKIIHYLIDNKRDWWNELEAKYDKDGVYRQKYK

>113951697 |Chemosensory protein

TNQYNDELDAALKSERLMKSYFECLLGTGKCTPSGEELKKDIPDALKNEC

AKCNDKHKEGIRKVIHYLVKQKPEWWEQLQKKFDPQGIYKKRYQ

>113951699 |Chemosensory protein

PTKYDNVDIDAILHNKRLFDNYLQCLLKKGKCNEEAAILRDVIPDALITG

CRKCNDHQKVSVEKVIRFLIKERNSDWQQLISVYDPKGEYQTQYA

>113951701 |Chemosensory protein

TTKYDNIDLENVVKNERLLKSYVDCLLEKGRCSPDGLELKKNMPDAIETD

CSKCSEKQKEGSDFIMRYLIDNKPDYWKALEAKYDPDGTYKKRYF

>113951703 |Chemosensory protein

IDVDEILKNDRLTRNYLDCVLGKGKCTPEGEELKKDIPEALQNGCAKCNE

KHKEGVRKVIHHLIENKPNWWQELESKFDPQGEYKKKYD

>113951705 |Chemosensory protein

IDIDEILKNDRLTKNYLDCILEKGKCTPEGEELKKDIPDALQNECAKCNE

KHKEGVRKVIRHLIKNKPSWWQELQEKYDPKGEYKSRYN

>113951707 |Chemosensory protein

IDLDEILKNDRLTRNYIDCILGKGKCTPEGEELKRDIPEALQNECAKCNE

KHKEGVRKVLHHLIKNKPNWWQELEAKFDPKGEYKQKYN

>113951709 |Chemosensory protein

ESRYDHLDVESILNNRRMVNYYAACLLSKGPCPPQGVDLKRVLPEALQTN

CAKCTEKQRTAAYRSIKRLKKEYPKIWEQLRAVWDPDDVFIRKFE

>113951711 |Chemosensory protein

NIDIDDILSNERLLKNYVNCLLDKGRCTPEGKKLKSTIPEALSTDCAKCN

EKVKANVRKVLHHLIDNKPDMWKQLEAKYDPSGEYRSKYK

>113951715 |Chemosensory protein

TTRYDNVDVDRILHSKRLLLNYINCLLEKGPCSPEGRELKKILPDALVTN

CSKCSEVQKKQAGKILTFVLLNYRNEWNQLVAKYDPDGIYRKQYE

>113951719 |Chemosensory protein

TNKYDNVDVDKILNNDRVLTNYIKCLMDEGPCTSEGRELKKTLPDALSSG

CTKCNQKQKETAEKVIRHLTQKRARDWERLSKKYDPQGQYKKRYE

>113951721 |Chemosensory protein

IDTILKNDQMTRNYLDCVLDKGKCTKEAEKLKKGITETMKNGCVKCEQKQ

KEDVHKVFQHLMIHRPNWWHELETKFNPHHEIKLQHL

>113970920 |Vertebrate odorant binding proteins

GKWYEIARLDHSFERGLTQVTAEYSLKADGGVKVINRGYSADTQQWKEAE

GKAYFVNGDEEAYLKVSFFGPFYGSYVVFGLDQQDYQYAFISGPDTDYLW

LLARTPTVSPEVMKQFVEMASARGFDTNSLIYVEQKAE

>114048144 |Vertebrate odorant binding proteins

GKWYEITRLDHSFERGLTQVTAEYSLKPDGGVKVINRGYSADTQQWKEAE

GKAYFVNGDGEAYLKVSFFGPFYGSYVVFGLDQQDYQYAFISGPDTDYLW

LLARTPTVSPEVMKQFVEMASARGFDTNSLIYVEQKAE

>116806264 |Juvenile hormone binding protein

YDTDTLIDALRQLGLSVEYEGSGELLFDLVNLRIAGTLKYKLPMLWGSAK

ITSLKTTISLESVTSDITGFMGNGKINRAINSQLENIVVKGINGNQDAIS

ETIENAIVPRVNKMLKGKDFWTV

>116806284 |Juvenile hormone binding protein

YDTDTLIDALRQLGLSVEYEGSGELLFDLVNLRIAGTLKYKLPMLWGSAK

ITSLKTTISLESVTSDITGFMGNGKINRAINSQLENIVVKGINNNQDVIS

ETIENAIVPRVNKMLKGKDFWTL

>1168469 |Aphrodisin

GKWYTIVIAADNLEKIEEGGPLRFYFRHIDCYKNCSEMEITFYVITNNQC

SKTTVIGYLKGNGTYQTQFEGNNIFQPLYITSDKIFFTNKNMDRAGQETN

MIVVAGKGNALTPEENEILVQFAHEKKIPVENILNILATDT

>117169194 |Odorant binding protein

TSKFDDINVDEILHSDRLLNNYFKCLMDEGRCTAEGNELKRVLPDALATD

CKKCTDKQREVIKKVIKFLVENKPELWDSLANKYDPDKKYRVKFE

>117574114 |Pheromone binding protein

QDVMKNLAINFAKPLDDCKKEMDLPDSVTTDFYNFWKEGYELTNRQTGCA

ILCLSSKLEILDQELNLHHGRAQEFAMKHGADETMAKQIVDMIHTCAQST

PDVAADPCMKTLNVAKCFKLKIHG

>118404322 |Chemosensory protein

NVKAQDDISKFLKDRPYVQKQLHCILDRGHCDVIGKKIKELLPEVLNNHC

NRCTSRQIGIANTLIPFMQQNYPYEWQLILRRYKIMKYY

>118778235 |Odorant binding protein

AECCVTPFLVEPSAFMTCHSKWIGQTKRQMAMEGIPRGCCVAECVMNSTS

LYSNGKIDREALTKLYLASTKSMAPEWNKITLDAIDGCFKMADTIKDEIE

AGAKL

>118780302 |Juvenile hormone binding protein

SFKGKYDLKIKLLLLNIAGVGDLSGVIENNQARVKLLCEKYAKDGKEYVR

VKKLVVRIQIEKGRFDMKDLFRGDPVLSQAGNQFINENSRLFLDELTPGL

ERSLSDTFKATANEIMQQATFDEIFPA

>118781254 |Odorant binding protein

TGCDASLDVPHLTLSKSFSRALQDCMEYLQVPGYRYAEYAANSFPDDPET

KCLLRCVGLNLRWWNDTTGMQTAVIEGFFHPDPLDELYENRTAECLRKEL

SHADTTDCCCLAYDSFRCYLQHYGN

>118791068 |Odorant binding protein

TTPRRDAEYPPPELLEALKPLHDICLGKTGVTEEAIKKFSDEEIHEDEKL

KCYMNCLFHEAKVVDDNGDVHLEKLHDSLPSSMHDIAMHMGKRCLYPEGE

TLCDKAFWLHKCWKQSDPK

>118793789 |Juvenile hormone binding protein

TIHAKYTSSGVLIIIPASGSGDFDAVFDGVTADVKGLVSTNEKPTGMHLR

VEKLDLNLSIKKPRLSVSKIFNNNRILTEATNLFLKENGHEVLRALQPQL

QKKLSSEFTGIANQLLDNVPIHFFIVD

>118793791 |Juvenile hormone binding protein

YVEGRYVVDGRILLLPVKGSGKFTGNFTQGIGSVRIKGDRKRINGKDHLS

LAKLDIKIRVSDGRVKLENLFGGDRVLGEIINETINQNFNLLSTELIPLI

EKALQRIFKRTGNKILERFPEEVLFP

>118794779 |Odorant binding protein

DEPRRDANYPPPELLEKMKPMHDACVAETGASEDAIKRFSDQEIHEDDKL

KCYMNCLFHQAGVVNDKGEFHYVKIQDFLPESMHLITLNWFKRCLYPEGE

NGCEKAFWLNKCWKTRDPV

>119113249 |Juvenile hormone binding protein

QFAGNYTMDGRILVLPITGKGLANITLHRLKTHHELIGELVERNGEQYMH

IRKYLVHFEPKLVTFQFGNLFNGDERLGKTMHQVLNDNWEVVFRELRSSY

EDTFGYIFKKISNQIFLKVPMNKIFPQ

>119508352 |Juvenile hormone binding protein

HFKSLYTAKGSLAYILNLAGDGNAETSITNFSILISFRLRSVSPLAISSL

QIELRLGGLWINFDNLMEEDRINDFIHALVNEMGVELLGDVWDYEQGTVV

SKVQAAVNNFLGQYSLSDIIQI

>119608456 |Odorant binding protein

GTWYVKAMVVDKDFPEDRRPRKVSPVKVTALGGGKLEATFTFMREDRCIQ

KKILMRKTEEPGKYSAYGGRKLMYLQELPRRDHYIFYCKDQHHGGLLHMG

KLVASAPCRAVPLSPRRLTWPPHLQV

>119608458 |Odorant binding protein

CPGGTTTSFTAKTSTMGACSTWESLWVGILIPTGRPWKNLRNWCSARDSR

RRTFSRPCRREAAFPNTRQPPGLHLQSPPYHQTQSPDHLDLPSSHDP

>119888034 |Odorant binding protein

TMTQIRNAMKPLGKTCLGKTGLSKEVQAGQHNGEFPEDEALMCYHSCLLK

LAKISDKSGNINLDTVHKQIDLMMPEDLIARAKAVTTDCFGEIKSTEICR

MSFEFVKCYFIKGPE

>119888036 |Odorant binding protein

LISNKAEAKSVQKRECPFKKPFEANAPKCMDKISEENMGRMMQGNMDNDE

IRCFVGCVFENAGFVKDNKVQMDKVREAVDNFVDDYKYSKEVGDQVYGVV

SDCAPQAEKGANNCEVSSNLLICFKTNNKF

>119888038 |Odorant binding protein

ACALVVGVLGDDDMKEKHKEIFKKCAEETGVTKEDLHNHKRGEEPETKIK

CFHACIAKADGAMVDGKLNKDKVIEKIPADLPDRERIIEAVTKCSEQTAA

DECETAHLVFKCLRENKAL

>119888040 |Odorant binding protein

EETFKACKDKLTPENFALLNKDPHADNQEIKCFKACGMNHAGIMADGKIQ

IEKMEEKLNSLLGEDKKDFSKIIIGRAKPCVEEANKGENECDVAAGFEAC

VQKTINT

>119888042 |Odorant binding protein

TATTESLQEIMNTFQKARLEVRAPCLHLLSNETLTTLKTRRHLDNPEIRC

FKACLMERQGYLKDNKIFIDEYEKLIDVNLKRIKELNMKFARACVNEAEK

SENKCELAHNYNRCILHQTRK

>119888044 |Pheromone binding protein

AQGEKGRCMSEHGTTEDMINMVNEGNIPNDPKLTCYMFCLFESFSIIDED

GVLEYGMLTEMFPDDIKAKAESVLSGCAEQPGADNCEKVYKIATCVQSKS

PD

>119925160 |Odorant binding protein

GSWRTVYIGSTNPEKIQENGPFRTYFRELVFDDEKGTVDFYFSVKRDGKW

KNVHVKATKQDDGTYVADYEGQNVFKIVSLSRTHLVAHNINVDKHGQKTE

LTGLFVKLNVEDEDLEKFWKLTEDKGIDKKNVVNFLENED

>119925395 |Vertebrate odorant binding proteins

KVRRMRTVSSSARKWMASKECLVCWFCSVDGTCLLLTEVAERQEGYVDEL

KYEGTNTFKLIHVSDNMLVTYIENDDGSKKTKITDGVVKGDSFTPEELQK

YQEVNTERGIPNENIENVIETGK

>120599491 |Vertebrate odorant binding proteins

GKWYEIARLDHSFERGLTQVTAEYSLKPDGGVKVINRGYSAAKQEWKEAE

GKAYFVNGDNEAYLKVSFFGPFYGAYVVFGLDQQDYQYAFISGPDTDYLW

LLARTPTVSPEVIQQFIKMAKAKGFDTDSLIYVEQKSA

>121485827 |Odorant binding protein

VLPLRADVNVMKDVTLGFGQALDKCRQESQLTEEKMEEFFHFWREDFKFE

HRELGCAIQCMSRHFNLLTDTSRMHHENTEQFIQSFPNGEVLARQMVELI

HACEKQHDHEEDHCWRILHVAECFKQACVQ

>121525756 |Vertebrate odorant binding proteins

WLLTIGDGRLGRLLGVEGQDVEQAIRQSLGGSLDWLVNILTSVGSQGLQI

VALLSLIVVTPVVAFYLLLDWDRMVERVDALLPRDHAETIRRLAREINEV

LAGFIRGQVIVCLVLGT

>122894080 |Chemosensory protein

TTKYDNIDLDEILSSERLLTGYVNCLLDQGPCTPDGKELKHTLPDAIDND

CRKCTQKQKEGSDRVMGYIIEYRPNDWAKLEKKYLSDGSYKKKYL

>122894082 |Chemosensory protein

TDRYDNVNLDELISNRRLLVPYVKCVLDQGKCSPDGKELKEHIQEALENN

CGKCTDKQREGTRKMIGHLINHEQEFWDQLIAKYDPERKYVSKYE

>122894084 |Chemosensory protein

TDRYDNINLDEILGNRRLLTPYIKCILEEGKCTPDGKELKSHIREALEQN

CAKCTDAQRNGTRRVLGHLINNEEESWNRLKAKYDPQSKYTVKYE

>122894086 |Chemosensory protein

TDRYDNVDLDEILSNRRLLVPYIKCILDQGKCAPDAKELKEHIREALENE

CGKCTETQKKGTRRVIEYLINNEEEYWNELTAKYDPERKYTTKYE

>122894088 |Chemosensory protein

TDKYDNIDLDEILNNKKILTSYINCCLDLGKCTPDGKELKSHIREALENK

CGKCTEAQKNGTRKVMTHLINFEPDYWNQLCAKYDPEGKYKAMYE

>123121834 |Odorant binding protein

GQWKTTAIMADNIDKIETSGPLELFVREITCDEGCQKMKVTFYVKQNGQC

SLTTVTGYKQEDGKTFKNQYEGENNYKLLKATSENLVFYDENVDRASRKT

KLLYILGKGEALTHEQKERLTELATQKGIPAGNLRELAHEDT

>123226369 |Probasin

GPWQTIYLAASTMEKINEGSPLRTYFRHILCGRRCNQVYLYFFIKKGTKC

QLYKVIGRKKQEVYYAQSKGKQLNKEEMTEFMNLVEEMGIEEENVQRIMD

TDN

>123230127 |Odorant binding protein

GQWKTTAIMADNIDKIETSGPLELFVREITCDEGCQKMKVTFYVKQNGQC

SLTTVTGYKQEDGKTFKNQYEGENNYKLLKATSENLVFYDENVDRASRKT

KLLYILGKGEALTHEQKERLTELATQKGIPAGNLKELAHEDT

>124246513 |Chemosensory protein

TSKYDGVNVDEILANDRLMMPYIKCALDHGRCSPEAKELKSHIKEALENN

CAKCTDKQKPAVRKVIAHLINHKPAEWRQLSDKYDPAGKYTAQYE

>124246515 |Chemosensory protein

DAKYDSFNAHELVQNQRLLKSYGKCFLSKGPCTAEGSDFKRVIPEALKTT

CGKCTRKQRELVRVVVKGFQEQLPQVWTEIVSKEDPKGEYKDSFA

>125778466 |Juvenile hormone binding protein

AFKGPYSVDGKVLILPIVGKGRAEIVLKHCKIHSLITLRPISKGGHHTFA

EVTDIKLQVDPSHVSYKLEGLFHGQKDLSENMHILINENWQEIFNELKPS

ISEAMGLIVKSVLNKTFGKTPLEELFIV

>125778468 |Juvenile hormone binding protein

VLRGKYTADGRILILPIRGDGDAEITLHSPKFSVKFKPGTQVRDGRTYLT

VDKLKVLVEPQRMNIKLTNLFNGDQALGANLNQFLNENWVEVWSELHPSI

HLAIAEIMKNILSNLFKRFAYEDLYLE

>125778614 |Juvenile hormone binding protein

IHKGRYMSQGRVWIVELNSTGEQLSDFQNFRFVLKLKVIMEYRNNKRYLK

IYELNPIVNMDRWVFWLDNFFSENTDLTIAINQVFNIHWVEFWNELEPTN

LRIFASVFRDLIEDILYKVSYDDMFLP

>125778616 |Juvenile hormone binding protein

LHEATYDMQGRFLLFVANTTGKLQSDFQNFRLTLTIKVILEYRNNKRYLK

IYDLVPVVDIDRWIVWFDNLYRENEDVTIALNRSFNKNWLEFWNDLEPGL

LKTFSTVFTVLLNTVFEQVAYDDMFLP

>125807118 |Insect pheromone binding protein

TSKFDNVNVDDVLGNQRVLNNYLKCLMDKGPCTAEGRELKRLLPDALQSD

CSKCTAAQRRNSEKVINILRSKYPGEWKQLLDKYDSKGIYRSKYE

>125808901 |Insect pheromone binding protein

IGLASAADEYKLRTAEDLQTVRKECGEANKVTEALVAKYKTFEYPDDEIT

RSYIQCIFHKFDLFDDTKGFKIDNLVAQLGQGKEDKAALKADIEKCADKN

EQKSSANAWAFRGFKCFLGKNLP

>125809549 |Insect pheromone binding protein

NECLYFSHITLEELQAQMNISSSEEDLENLDRKYKCFAHCLVARANLLDS

RGRVDVAKIDELEPLTDEHRQALENCKRAHDDEPDNCEYAFSMFLCLSDY

LEA

>125976718 |Odorant binding protein

KLKGDYSLFGRILLIPLNGRGQVFLDADNMTVTMHTKTRLYSKGGFTFYN

VTNCRVDFKMDGLRSYFSNLFNGNKQLEDSTNKFFNDNWRMLADALYTVI

TQTIEDILLDVLKKIFHYIPANFFVS

>125984734 |Juvenile hormone binding protein

NIVGSYKADMQVNQLQLKPKGEFNVTLMDVETTTLTEGELYEKDGHRFLR

LTGIETKPKIGDLTIKANGIFPDPELDQIALNVANQYWRDIYGIMLPETR

QYWQPLLLRMFNESLELVPIDQFIKE

>125984936 |Juvenile hormone binding protein

SVRSSKYMLDILARLFGSDFALWGDGVFSLELIDFRAHGSFVIRPTTASS

GVYVKSWQVNWQLGEARSQTTGIMNSRLYTKFVNDLVKDYLELLVNDNPA

EVSQFMEGLIVPPLNAVLENVAWYEI

>125984938 |Juvenile hormone binding protein

YSTDTLIDALRQLGLSVEYEGTGDLLFDLNNLRIAGTLKYKLPMLWGSAK

ITSLKTTISLESVESDISGFMGHGKINRAINRQLENVVVKAINENQQAIS

DTIEDTIVPPVNKMLKGKDFWTL

>125984940 |Juvenile hormone binding protein

NTDTFVDMLRQLGLSVRYESTGPMSFALENLSIQGQFKYKMPFLFGSIKI

SKFQCTVGLGGVSSNIGGVMGNGRINEFINDMIDYEVPAFINGQQEAISK

QIEEVFVPIINQKLKGHKIWYLL

>125985295 |Juvenile hormone binding protein

LVKGKYNLASWISRAQGPFTVILKNVYAEATAFLAVERDGHLTTDRIKID

ITFRDMAMDFQNLGFMGSLFQGMVNSAPNLVFDAMKPFMLQEADKQLRSE

IDTMIKT

>126002371 |Insect pheromone binding protein

NEKFDNVDLDEILIQERLLNNYIKCLESAGPCTPDAKMLKDILPDAVLTD

CTKCTEKQKIGAEKVTRHLIDNRPNDWERLEKIYDPEGTYRFKYL

>126173745 |Vertebrate odorant binding proteins

GKWYEIARLDHSFERGLSQVSAEYSLKDDGGVMVINRGFSAAKNEWKEAE

GKAYFVNGDSEGYLKVSFFGPFYGSYVVFELDHENYQYAFISGPDTDYLW

LLAKTPTVPPEVLQKFVEMSKARGFDTDSLIYVQQESA

>126463195 |Vertebrate odorant binding proteins

AAPTIFRQLHSFLLDHFPQLSDETSTARQTLLSIGDTIKERGGEVANTLL

TSAYGVINAMIFMVVVPVVSFYLLLDWDPMVAKIDSWLPRDHAPTLRRLA

REIDEVLAGFVRGQISVCLVLGT

>126508766 |Odorant binding protein

NIPHVESRMSMAQTINTMKPLGKTCAAKTGLSKEMQDGQHEGQFPEEEAL

MCYHTCLLKMAKVADKTGKLNIDAMVKQIDMLMPEDLVDKAKTACSGCAD

EVTATEGCRPSWEFMKCWYGRAPE

>126723762 |Vertebrate odorant binding proteins

GEWYSIFLASDVKEKIEENGSMRVFVDVIRALDNSSLYAEYQTKVNGECT

EFPMVFDKTEEDGVYSLNYDGYNVFRISEFENDEHIILYLVNFDKDRPFQ

LFEFYAREPDVSPEIKEEFVKIVQKRGIVKENIIDLTKIDR

>12860858 |Vertebrate odorant binding proteins

GEWFVLGLADNTFRREHRALLNFFTTLFELKEKSQFQVTNSMTRGKHCNT

WSYTLIPATKPGQFTRDNRGSGPGADRENIQVIETDYITFALVLSLRQTS

SQNITRVSLLGRNWRLSHKTIDKFICLTRTQNLTKDNFLFPDLSDW

>129022 |Odorant binding protein

GPWRTVYIGSTNPEKIQENGPFRTYFRELVFDDEKGTVDFYFSVKRDGKW

KNVHVKATKQDDGTYVADYEGQNVFKIVSLSRTHLVAHNINVDKHGQTTE

LTELFVKLNVEDEDLEKFWKLTEDKGIDKKNVVNFLENED

>129673 |Pheromone binding protein

INLVHSSPEIMKNLSNNFGKAMDQCKDELSLPDSVVADLYNFWKDDYVMT

DRLAGCAINCLATKLDVVDPDGNLHHGNAKDFAMKHGADETMAQQLVDII

HGCEKSAPPNDDKCMKTIDVAMCFKKEIHK

>130701 |Vertebrate odorant binding proteins

GTWHSMAMATNNISLMATLKAPLRVHITSLLPTPEDNLEIVLHRWENNSC

VEKKVLGEKTENPKKFKINYTVANEATLLDTDYDNFLFLCLQDTTTPIQS

MMCQYLARVLVEDDEIMQGFIRAFRPLPRHLWYLLDLKQMEE

>13124669 |Vertebrate odorant binding proteins

GKWHSLYIAADNKSKVSEGGPLRVYVKHLECSDECQTFTIKFYTKVENVC

QEHRVVGRKGKDGKYITDFSGQNYFHVVEKADDTMTFHNVNVDDSGKTNV

ILVVGKGESSSIEQKQRFEKTAEKYDIPKENIEHLVTTDT

>133919124 |Pheromone binding protein

EMVCGSRDVMTNLSIQFAKPLEACKKEMGLTETVLKDFYNFWIEDYEFTD

RNTGCAILCMSKKLELMDGDYNLHHGKAHEFARKHGADETMAKQLVDLIH

GCSQSVATMPDECERTLKVAKCFIAEIHK

>134270321 |Pheromone binding protein

MGSAMSSKELLTKMTGGFTKVVDHCKTELNVGDHIMQDMYNFWREEYQLV

NRDLGCMIMCMTAKLDLVGDDQKMHHGKAEEFAKSHGADDALAKQLVGLI

HGCETQHQAIEDHCSRTLEVAKCFRTKIHE

>13430369 |Vertebrate odorant binding proteins

GKWYSLYIAADNKEKVSEGGPLRAYIKNVECIDECQTLKITFYTKVEGVC

QEHTIVGRKGEDGKYITDFSGQNYFHIVEKSDDTMTFHNVNVDDSGKTNV

ILVVGRGESSSIEQKQRFEKTAEEYDIPKENIEDLVPTDN

>134948553 |Pheromone binding protein

VVGARGSSEAMRHIATGFIRVLDECKQELGLTDHILTDMYHFWKLDYSMM

TRETGCAIICMSKKLDLIDGDGKLHHGNAQAYALKHGAATEVAAKLVEVI

HGCEKLHESIDDQCSRVLEVAKCFRTGVHE

>137823 |Vertebrate odorant binding proteins

GTWYLKAAAWDKEIPDKKFGSVSVTPMKIKTLEGGNLQVKFTVLIAGRCK

EMSTVLEKTDEPAKYTAYSGKQVLYIIPSSVEDHYIFYYEGKIHRHHFQI

AKLVGRDPEINQEALEDFQSVVRAGGLNPDNIFIPKQSET

>13959427 |Juvenile hormone binding protein

TATGIQTTDAEIMGIPLKGSGPFTISLANPSLAVTLTGAPSAGPNGQTYL

RLTSASAAIEPGTPTADIKGFFPQFPPLEAAASAFASVVAPDVVQSLKPT

LDKWLGGVALQRAQAVFSSVSYDALFPG

>14091480 |Chemosensory protein

TDKYDNINLDEILENKRLLLAYVNCVMERGKCSPEGKELKEHLQDAIETG

CSKCTEAQEKGAYKVIEHLIKNELDIWRELAAKYDPKGDWRKKYE

>15826041 |Pheromone binding protein

GKWYTIVIAADNLEKIEEGGPLRFYFRHIDCYKNCSEXEITFYVITNNQC

SKTTVIGYLKGNGTYETQFEGNNIFQPLYITSDKIFFTNKNXDRAGQETN

XIVVAGKGNALTPEENEILVQFAHEKKIPVENILNILATDT

>15983753 |Odorant binding protein

LADINVMKDVTLGFGQALDKCREESQLTEEKMEEFFHFWRDDFKFEHREL

GCAIQCMSRHFNLLTDSSRMLHDNAEKFIQSFPNGEVLARQMVELIHSCE

KQFDHEDDHCWRILHVAECFKGSCVQ

>16225961 |Vertebrate odorant binding proteins

ACGLFVIAQANTVKKCEKKMPASLKSQLCEIRKYKLLDTPDMDSHMDCVM

KALDFVRPDGTGDYHKLIKPLNAIEKDRKHDFNLEKCGGQTQHLPVGKRA

NAYYKCLVESTSG

>1710156 |Juvenile hormone binding protein

LMRNLLARYDLERLSNHMLPVTPVDLWEPVSEGYDPQLRLLSGKEAAARP

EGLRPTHADVISLDDVISWERRVRDAAATALFLNEKKL

>1718160 |Vertebrate odorant binding proteins

GRWYLKAMTSDPEIPGKKPESVTPLILKALEGGDLEAQITFLIDGQCQDV

TLVLKKTNQPFTFTAYDGKRVVYILPSKVKDHYILYCEGELDGQEVRMAK

LVGRDPENNPEALEEFKEVARAKGLNPDIVRPQQSET

>17647793 |Pheromone binding protein

VLLGAALVRAFDEKEALAKLMESAESCMPEVGATDADLQEMVKKQPASTY

AGKCLRACVMKNIGILDANGKLDTEAGHEKAKQYTGNDPAKLKIALEIGD

TCAAITVPDDHCEAAEAYGTCFRGEAKK

>17738189 |Juvenile hormone binding protein

SLVGPYNIQGKVLILPISGTGQSNMTMVNVRAIVSFSGKPLVKNGETYLD

VTDLKITMKPESSHYHFSNLFNGDKALGDNMNVFLNENSEAIYKETAKAI

DRSFGKLYLGVVKGVFSKLPYAKFFAD

>17943132 |Odorant binding protein

GPWRTVYIGSTNPEKIQENGPFRTYFRELVFDDEKGTVDFYFSVKRDGKW

KNVHVKATKQDDGTYVADYEGQNVFKIVSLSRTHLVAHNINVDKHGQTTE

LTELFVKLNVEDEDLEKFWKLTEDKGIDKKNVVNFLENEN

>17981809 |Odorant binding protein

FALVAFASASRDSAKKIGSQYEHYATCLTENDAAADDIFTILDITSGHHK

NENEHDKQHKNGCVMHCLLEKDGLMTGADYHEEKIREDYIKETGAQPGDK

RLEALDTCMNETKDMTDKCDKSLLLVACVLIAEDS

>18140737 |Antennal binding protein

ILADGVDSMSKQQLKNSGKMFKKQCMGKNKVTEDEIGEIDKGRFVEQQNV

MCYIACIYQMSQVVKNNKLNYEASLKQIDIMYPPELKDTAKGALEACKDI

AKKNKDLCEASFKTAKCMYEYSPK

>1827612 |Odorant binding protein

GPWRTVYIGSTNPEKIQENGPFRTYFRELVFDDEKGTVDFYFSVKRDGKW

KNVHVKATKQDDGTYVADYEGQNVFKIVSLSRTHLVAHNINVDKHGQTTE

LTGLFVKLNVEDEDLEKFWKLTEDKGIDKKNVVNFLENED

>19071280 |Odorant binding protein

PFPSVECAMTRKQLINSMDMMRSACAPKFKVSTEMLDNLRGGIFAEDREL

KCYTMCIAQMAGTMNKKGEINVQKTLAQMDAMLPPDMRDKAKEAIHSCRD

VQGRYKDSCDKTFYSTKCLAEYDRD

>19224143 |Odorant binding protein

VTPRRDAEYPPPELLEALKPLHDICAKKTGVTDEAIIEFSDGKIHEDEKL

KCYMNCLFHEAKVVDDNGDVHLEKLHDSLPNSMHDIAMHMGKRCLYPEGE

NLCEKAFWLHKCWKQADPK

>19922608 |Odorant binding protein

TLAVGSSLNLSDEQKDLAKQHREQCAEEVKLTEEEKAKVNAKDFNNPTEN

IKCFANCFFEKVGTLKDGELQESVVLEKLGALIGEEKTKAALEKCRTIKG

ENKCDTASKLYDCFESFKPA

>19922956 |Chemosensory protein

TNKYDSVNVDEVLGNNRVLGNYLKCLMDKGPCTAEGRELKRLLPDALHSD

CSKCTEVQRKNSQKVINYLRANKAGEWKLLLNKYDPQGIYRAKHE

>20129495 |Juvenile hormone binding protein

NIVGSYKADMQVNQLQLKPKGEFNVTLLDVEAITVTDGEVYEKDGHRFFR

LKNIDSKPKIKDLVIKANGIFADPELDKIALNVANQYWRDIYGIMLPETR

QFWQPLMLRMFNEAFELVPIDQFLKE

>21314941|Insect pheromone/odorant binding protein

VALVTCGLLIIVQAAKKVEQCEKRIPDSLKHKLCQIRQYQLLEGADMEKH

IDCVMRALGFVHPDGSGNYHALIEPLNAIDKDRKHGFNLETCGGNRNNLP

KRKRAYAFYKCMLKSTSA

>21355505 |Juvenile hormone binding protein

EMIGDYEMSGRILLLPITGHGKANVTLINTKIEHRLIGEPFEKDGVKYMR

LKDYRVSFDPKRVYMNFENLFNDKTLSDGMNRFLNENWETVFNELKVGYA

KSFGIIFRELSNKLFEKVPFDNIFLS

>21730166 |Odorant binding protein

GPWRTVYIGSTNPEKIQENGPFRTYFRELVFDDEKGTVDFYFSVKRDGKW

KNVHVKATKQDDGTYVADYEGQNVFKIVSLSRTHLVAHNINVDKHGQTTE

LTGLFVKLNVEDEDLEKFWKLTEDKGIDKKNVVNFLENEN

>21898556 |Chemosensory protein

TDKYDNVDLDEILSNRRLLVPYVKCILDQGKCAPDAKELKEHIIEALENE

CGKCTEAQKKGTRRVIGHLINNEADYWNELTAKFDPEKKYVQKYE

>21898574 |Chemosensory protein

TDKWDNINVDEILESQRLLKAYVDCLLDRGRCTPDGKALKETLPDALENE

CSKCTEKQKAGSDKVIRYLVNKRQDLWKELSAKYDPNNIYQDRYK

>21898673 |Chemosensory protein

TTKYDNIDLDEILGSKRLLNNYFNCLLDKGPCTPDGKELRDHIPDALETG

CDKCSDKQKNGTRRVLKFLIDNEPDRYKELENKFDPEGTYRKKYE

>22094831 |Juvenile hormone binding protein

AAYDDIGLIFHFKNLNITGLKNQKISDFRMDTTRKSVLLKTQADLNVVAD

VVIELSKQSKSFAGVMNIQASIIGGAKYSYDLQDDSKGVKHFEVGQETIS

CESIGEP

>22770450 |Juvenile hormone binding protein

KTKVNFTAEGKLVIELPKSSKTYTGEVTIEASAEGGAAYSYSVKTDDKGV

EHYEAGPETVSCEIFGEPTLSVSSTLEDALKLDSDFKKIFTGYGKQLTEG

RKQTACRIVETVYAVSVHNIRAA

>23320747 |Odorant binding protein

LGVADLASGLTGRAFERAKEVDEKCRSENNVERAYFEKFIKARIDEIDPP

DNYKCFVKCVMVELMALNDEGDFNVDEELQNVPPEIVEEGHRIVKTCHGT

PGKDPCDKAYQVHKCYHKENPE

>2392495 |Odorant binding protein

GPWRTVYIGSTNPEKIQENGPFRTYFRELVFDDEKGTVDFYFSVKRDGKW

KNVHVKATKQDDGTYVADYEGQNVFKIVSLSRTHLVAHNINVDKHGQKTE

LTGLFVKLNVEDEDLEKFWKLTEDKGIDKKNVVNFLENED

>2444185 |Pheromone binding protein

VMRVDCSKEVMKQMTINFAKPMEACKQELNVPDAVMQDFFNFWKEGYQIT

NREAGCVILCLAKKLELLDQDMNLHHGKAMEFAMKHGADEAMAKQLLDIK

HSCEKVITIVADDPCQTMLNLAMCFKAEIHK

>24643509 |Pheromone binding protein

TSAKPHEEINRDHAAELANECKAETGATDEDVEQLMSHDLPERHEAKCLR

ACVMKKLQIMDESGKLNKEHAIELVKVMSKHDAEKEDAPAEVVAKCEAIE

TPEDHCDAAFAYEECIYEQMKE

>24644477 |Odorant binding protein

QEPRRDGEWPPPAILKLGKHFHDICAPKTGVTDEAIKEFSDGQIHEDEAL

KCYMNCLFHEFEVVDDNGDVHMEKVLNAIPGEKLRNIMMEASKGCIHPEG

DTLCHKAWWFHQCWKKADPV

>24648633 |Odorant binding protein

SSCCDVQKNDKAINSCRKSLLGNNSTNSNGEVRNLKSDKVALHACIAECS

FRTNGFLLSNGTVNTQALQKSYQQRYKNDPNMSQLMLKSLNSCTDYARKR

VQEFQWMPKKGDCDFYPATLLACVMEKVYI

>24649971 |Juvenile hormone binding protein

VLKGKYVADGRILILPIRGDGDAEIVLHNPKFSVKFKPGTQQRNGRTYLS

VDKLKVLVEPQKMNIRLENLFNGDQALGTNLNQFLNDNWTEVWNELHPSI

HVAIAEIMKSVLSQLFKRFAYEDLFLE

>24652406 |Odorant binding protein

LGRDRRSLNLNEVKEHLESDLVNDADIKLLYDTYVKCDKHALSLMPHKGV

KQLSKRLSRLGCHPYPGLVLECVANEMILHCPTKRFRQTAQCEETRNHL

>24653178 |Odorant binding protein

VNPKTCCPMPDFVTAELKQKCIKFDMTPPPPPDGEASGSFESKRRHHHPH

PPPCFFSCIFNETGIYQNRKLDEAKLNAYLQEVFEDSSDLQTTATQAFTT

CATKVADFEANLP

>24653631 |Odorant binding protein

PFRAAKCRAAPKSVQNVHVCCSAPLPNWGVFNRECHKSAIQASCRLDCDF

NASSVLQGNRLIQAKVRPMLERAFSNEPTIDAYESNFAKCSTVVRSKYQE

LSPLSRQSDACDRHALFYSLCAYARLIF

>24653633 |Odorant binding protein

PLLVYSVSNDMGGLQKCTELLNTHKLVYCCGKSFLDKFPFVGSNCTPFWD

DYGPCRYECLYRHWDLLDQDNKIKKPELYLMITSLYSPLNGYDKYGAAFK

AAHETCEALGSRHADFLLLYSNQVADKMGM

>24656242 |Odorant binding protein

LAKARHPFDIFHWNWQDFQECLQVNNITIGEYEKYARHETLDYLLNEKVD

LRYKCNIKCQLERDSTKWLNAQGRMDLDLMNTTDKASKSITKCMEKAPEE

LCAYSFRLVMCAFKAGHP

>24658422 |Odorant binding protein

VAQCFFEEMNMVGDPRDSFQSRNPSEKQELIPFNSHPQVDGNGMPDRRKV

SYLLTKDLRDRELRNFFTDTVQQCFRYLESNGRGRHHKCSAARELVKCMS

EYAKA

>24658435 |Odorant binding protein

YCCKHPDGHNDLIEGCARETNFTLPNQNEEALVDITADRAIRGTCFGKCV

FSKLNLMKDNNLDMDAVRSLFTERFPDDPEYAKEMINAFDHCHGKSEENT

SMFLSKPLFKQMSK

>24665733 |Antennal binding protein

DDKFDNVDLDEILNQERLLINYIKCLEGTGPCTPDAKMLKEILPDAIQTD

CTKCTEKQRYGAEKVTRHLIDNRPTDWERLEKIYDPEGTYRIKYQ

>24668608 |Odorant binding protein

KLQADYSLFGRILLIPLNGKGQVFLDAENMTVTMHTKTRLYSKGGFTFYN

VTNLHVDFKMDGLKSYFSNLFNGNKQLEDSTNKFFNDNWRMLADALYTVI

TQTIEDILLDVLKKIFHFIPANFFVS

>24762502 |Hypothetical proteins

TTKYDNIDVDEILKSDRLFGNYFKCLVDNGKCTPEGRELKKSLPDALKTE

CSKCSEKQRQNTDKVIRYIIENKPEEWKQLQAKYDPDEIYIKRYR

>2494867 |Pheromone binding protein

INLVHSSPEIIKNLSQNFCKAMDQCKQELNIPDSVIADLYNFWKDDYVMT

DRLAGCAINCMATKLDVVDPDGNLHHGNAKEFAMKHGADASMAQQLVDII

HGCEKSAPPNDDKCMKTIDVAMCFKKEIHK

>2494870 |Odorant binding protein

LHPILADVNVMKDVTLGFGQALEKCREESQLTEEKMEEFFHFWSEDFKFE

HRELGCAILCMSRHFNLLTDSSRMHHENTDKFIKSFPNNEVLSKHMVNLI

HSCEQQHDADLDHCWRILRVAECFKRSCQE

>2494872 |Odorant binding protein

AAPLLADVNVMKDVTLGFGQALDKCREESQLTEEKMEEFFHFWRDDFKFE

HRELGCAIQCMSRHFNLLTDSSRMHHDNTEKFIQSFPNGEVLARQMVELI

HSCEKQFDHEEDHCWRISHLADCFKSSCVQ

>25990272 |Pheromone binding protein

ASSVMASKELITKMSSGFTKVVDQCKNELNVGEHIMQDMYNFWREEYELL

NRDLGCMVMCMANKLDLIGEDQKMHHGKAADFAKSHGADDDQAKQLVGIV

HDCENTHQGVEDACSRALEVAKCFRSKMHE

>26007502 |Pheromone binding protein

FITVECSQEIMKNLSMNFAKPLEDCKKEMDLPDSVTTDFYNFWKEGYEFT

NRQTGCAILCLSSKLELLDQELKLHHGKAQEFAQKHGADEAMAKQLVDLI

HGCTQSTPDVAADPCMKALNVAMCSKTKVHE

>26007526 |Chemosensory protein

TDRYDSVDLDEILGNRRLMVPYIKCILDQGKCAPDAKELKEHIREALENE

CGKCTETQKNGTRRVIGHLINHEDAYWKELTAKYDPQSKFTAKYE

>266472 |Vertebrate odorant binding proteins

GKWYGIGLASNSNWFQSKKQQLKMCTTVITPTADGNLDVVATFPKLDRCE

KKSMTYIKTEQPGRFLSKSPRYGSDHVIRVVESNYDEYTLMHTIKTKGNE

VNTIVSLFGRRKTLSPELLDKFQQFAKEQGLTDDNILILPQTDS

>27065152 |Chemosensory protein

TDKYDNINLDEILANKRLLVAYVNCVMERGKCSPEGKELKEHLQDAIENG

CKKCTENQEKGAYRVIEHLIKNEIEIWRELTAKYDPTGNWRKKYE

>27414057 |Odorant binding protein

AAPPPDLEDVSKIANGEAFALECLIESGLKLDSLAALSAKELDTNGSKIK

CLVKCFFEKTGFMNKDGQLQEETITEQLSKFMPRERIESLVKNCNFQEAD

ACETAYKVTECYFQNKAG

>27414089 |Odorant binding protein

PLPHYFVRKSFPEAQAECAVYLQVPDDRLQRYMREGYPDEPEVHCLVLCV

LENLRAWENGTLHENVLANYFVPATEDCDNAKRTERCLVNLPQECNGEPC

VQAYRAFQCYYQNYGT

>27414091 |Odorant binding protein

PLPHYFVRKSFPEAQAECAVYLQVPDDRLQRYMREGYPDEPEVHCLVLCV

LENLRAWENGTLHENVLANYFVPATEDCDNAKRTERCLVYLPQECNGEPC

VQAYRAFQCYYQNYGT

>27464446 |Pheromone binding protein

VRKAEPSKDAMQYITSGFVKVLEECKHELDLNEQILADLFHFWKLEYSLL

GRDTGCAIICMSKKLDLLDANGRMHHGNAAEFAKKHGAGDEVASKIVTII

HECEKKHEQDGDECLRVLEVAKCFRTGIHE

>27465627 |Odorant binding protein

GKWYTKATVCDRNHTDGKRPMKVFPMTVTALEGGDLEVRITFRGKGHCHL

RRITMHKTDEPGKYTTFKGKKTFYTKEIPVKDHYIFYIKGQRHGKSYLKG

KLVGRDSKDNPEAMEEFKKFVKSKGFREENITVPELLDE

>27543486 |Chemosensory protein

TTKYDNIDLDEILNNERLLKKYYECLMSDSDASCTPDGKELKVSIPDALV

TDCSKCNEKQKEGSNKVIRFLIQKKEDLWKPLQAKYDPEGTYLKKHP

>27543488 |Chemosensory protein

TTKYDNIDLDEILNNERLLKKYHECLMSDSDASCTPDGKELKVSIPDALV

TDCSKCNEKQKEGSNKVIRFLIQKKEDLWEPLQAKYDPEGTYLKKHP

>27543490 |Chemosensory protein

TTKYDNIDLDEILNNERLLKKYYECLMSDSDASCTPDGKELKVSIPDALV

TDCSKCNEKQKEGSNKVIRFLIQKKEDLWKSLQAKYDPEGTYLKKHP

>27543492 |Chemosensory protein

TTKYDNIDLDEILHNDRLLKKYHECLLSDDDASCTPDGKELKAAIPDALT

NECSKCNEKQKNGAEKVIRFLIKEKPDLWTPLETKYDPSGSYRQKYA

>27543494 |Chemosensory protein

TTKYDNIDLDDVLHNDRLLKKYHECLLSDSDASCTPDGKELKAAIPDALT

NECAQCNEKQKAGAEKVIKFLIKEKPDLWEPLEKKYDPTGSFRQKYD

>27543498 |Chemosensory protein

TTKYDNIDLDDVLHNDRLLKKYHECLLSDSDASCTPDGKELKAAIPDALT

NECSKCNEKQKAGAEKVIRFLIKEKPDLWTPLENKYVPSGSYKQKYD

>27543500 |Chemosensory protein

TTKYDNIDLDEILHNDRLLKKYHECLLADDDASCTPDGKELKAAIPDALT

NECAQCNEKQKNGAEKVIRFLIKEKPDLWTPLENKYDPSGSYRQKYD

>27543502 |Chemosensory protein

TTKYDNIDLDDVLHNDRLLKKYHECLLSDSDASCTPDGKELKXAIPDALT

NECSKCNEKQKAGAEKVIRFLIKEKPDLWTPLENKYDPSGSYRQKYG

>28396154 |Antennal binding protein

AFGRNEIVPGLIDVAPEQTIKITYPQSDVEVSLGNQLTPTQVKARPKLCW

EVEPSALYTLLMADPDAPSRSNPEMRSWKHWLVGNIPGADVDAGDVLADY

VGSGPPQGTGLHRYVFLVY

>28396160 |Antennal binding protein

TVEGAALNRSPRQLSSLLTLSGESNARIENGTIICDTLKCPAESFKCVIV

KNSTKDDVNKVQVTRECLDPAGKATAKTVTTE

>28572146 |Juvenile hormone binding protein

VHEATYDMSGRVLLFFFNTTGRLISDFQNFRITLTIKALVEYRNDKRYLK

IYNLVPSLDLDRWIIWLDGLYKENTDVTIFMNKLFNENWVEFWNDLQPGL

VKAFTNAFTVLLNRVFDNVAYDDMFLP

>29377203 |Pheromone binding protein

SLDKVDVQVVKEVNTGKNLFEGKELDVVKISGEIVAQEQGNAALKIREIP

GTYYIQLNTQKDLLANKNARRAIALSLNSERLAKNVLNDGSKKALGFVPT

GFTNQETQKDFAEELGDLN

>29377538 |Pheromone binding protein

FLAVVSIAWLAPQNQKFVEAQGKDYALDSEHLLYSGPFTLANWDATSDTW

TLKKNPEYYDADQVKLEEVAVSTIKEDNTGINLYQVNEL

>29377877 |Pheromone binding protein

LSLTAYYPVQQKAIKEYGKDYGTSQKSIVTNGAFNLTNLEGVGTSDKWTI

SKNKEYWDQKDVSMDKINFQVVKEINTGINLYNDGQLDDAPLAGEYAKQY

KKDKEY

>30984068 |Juvenile hormone binding protein

QMDTKAKTVLLKTKADLHIVGDIVIELTEQSKSFTGLYTADTNVIGAVRY

GYNLKNDDDGVQHFEVQPETFTCESIGEPKVTLSSDLSSALEKDSGNNSL

EPDMEPLKTLRQAAICKIAEACYISVVHNIRASAKILPASSFFEN

>31239625 |Odorant binding protein

FLLVASVHAFTLRQQKMVSIFALECMAETGIGAESLTKLRDGDLTANDRT

AKCFMKCFFEKENFMDAEGKLQLEAIATALEKDYERAKIDEMLEKCGEQK

EDACETAFNAYACYHDHY

>31241197 |Odorant binding protein

PAFRPASFLEVMEVVLDCFNTLRIPLQRFPSYLSGIFPEDPETKCFLRCV

AIKLGVYCDEKGADLDRHCVQFGLGECCENFSNRHLVCLQQNSLPCPDRC

TAAYKQELCFQEPIAK

>31322218 |Odorant binding protein

EMKELAQQLHNTCVSETGTTEDAITNARAGTFTDDEKFKCYLKCLLDQMA

IVDEEGRIDVEAMIAVLPEEFQDSLPPVIRECDTIIGANACDNVWLTQQC

YYKENPE

>31442896 |Chemosensory protein

YDYIDPMEIVNNDRLRDQYYNCFMNTGPCVTPDAIYFKEHFPEAVVTKCK

KCTEIQKTNFEKLAIWYNENRPDEWTALIKKFM

>31747328 |Chemosensory protein

YDNIDLDEILSNRRLLLSYFNCVMGKGKCTAEGKELKDNLEDAIKTGCAK

CTENQEKGSYRVIEHLIKNELDLWRELCAKFDPTGEWRQKYE

>31747523 |Antennal binding protein

VVMDEDMAELARMVRESCVDETGADVKLVEKVNGGADLMEDDKLKCYIKC

TMETAGMMSDGEVDIEAVMALLPPEMAEHNGPALKSCGTQRGADDCDTAW

KTQVCWQNANKA

>31747525 |Antennal binding protein

PGDGRHDVGRQVDIEAVMALLPPEMAEHNGPALKSCGTQRGADDCDTAWK

TQVCWQNANKA

>31747533 |Antennal binding protein

PGDGRHDVGRQVDIEAVMALLPPEMAEHNGPALKSCGTQRGADDCGTAWK

TQVCWQTANKA

>31747535 |Pheromone binding protein

FITVECSQEIIKNLSLQFAKPLEDCKKEMDLSDTVITDFYNFWKEGYEFT

NRQFGCAILCLSSKLELLDQDLKLHHGKAQEFAKKHGADEAMAKQLVDMI

HSCSQSTPDVADDPCMKTLNVAKCFVAKIHD

>31747543 |Pheromone binding protein

QEVVASFSKGFTNVVEHCKAEVNAGEHIMQDIYNFWREEYQLVNRDLGCM

VLCMANKLGLIGEDQKMHHAKAEEFAKSHGADEAVAKQLVAILYECETST

RPVEDECGWRLEIAKCFRTKMHE

>31747545 |Pheromone binding protein

QKVVASFSKGFKDVVDHCKAELNMGEHIMQDVYNFWREEYQLVNRDFGCM

VLCTANKLGLLKEDQKMHHDKAEEYAKKHGADDATAKQVVAIIFECENNN

SGMDDECNRALEIAKCFRTKMHE

>31747547 |Pheromone binding protein

HIMQDMYNFWREEYQLVNRDLGCMIMCMTAKLDLVGDDQKMHHGKAEEFA

KSHGADDALAKQLVGLIHACETQHQAIEDHCSRTLEVAKCFRTKIHE

>31982543 |Hypothetical proteins

LGPVISLLQFEAKMDVMTTIAVASNNTQCVNLDAQDTHMHVKEMKIQLVE

TVTGKVPLPVPLPLDQIIPAIVTAKINENLEKSNSCAIVLNDFNNCKNNT

GL

>32135154 |Odorant binding protein

QEPRRDGEWPPPAILKLAKHFHDICAPKTGVTDEAIKEFSDGQIHEDEAL

KCYMNCLFHEIEVVDDNGDVHMEKLFNAIPGEKLRNILMEASKGCMHPEG

DTLCHKAWWFHQCWKKADPV

>32135155 |Odorant binding protein

AAAQRDENYPPPGILKMAKPFHDACVEKTGVSEAAIKEFSDGEIHEDEKL

KCYMNCFFHEIEVVDDNGDVHLEKLFATVPLSMRDKLMEMSKGCVHPEGD

TLCHKAWWFHQCWKKADPK

>33328843 |Juvenile hormone binding protein

YNTDTLIDALRQLGLSVEYEGSGELLFDLVNLRIAGTLKYKLPMLWGSAK

ITSLKTTISLESVTSDITGFMGNGKINRAINSQLENIVVKAINGNQDTIS

ETIENAIVPRVNKMLKGKDFWTI

>33328933 |Juvenile hormone binding protein

VLKGKYVADGRILILPIRGDGDAEIVLHNPKFSVKFKPGTQLRNGRTYLS

VDKLKVLVEPQKMNIRLENLFNGDQALGSNLNQFLNDNWTEVWNELHPSI

HVAIAEIMKSVLSQLFKRFAYEDLFLE

>33328935 |Juvenile hormone binding protein

NIVGSYKADMQVNQLQLKPKGEFNVTLMDVEATTLTDGEIYEKDGHRYFR

MKNIETKPKIRDLVIKANGIFADPELDQIALNVANQYWRDIYGIMLPETR

QYWQPLLLRMFNEAFELVPIDQFLKE

>33355889 |Odorant binding protein

TMRCCNDGFEKSEVHAKFAEVRTACMEELEHLNCITECIAKKEGIADENG

ALLHTDLAKVVLEHMSTIEWKVPLAEGFIQQCFDEVELTDGAFVPSDEAK

CNPEGFDFVFCLWRQFTL

>33356152 |Odorant binding protein

QALTDEQIQKRNKISKECQQVSGVSQETIDKVRTGVLVDDPKMKKHVLCF

SKKTGVATEAGDTNVEVLKAKLKHVASDEEVDKIVQKCVVKKATPEETAY

DTFKCIYDSKPD

>33413583 |Chemosensory protein

ADTYDHIEPMEILNNDELRNQYYNCVMNTGPCMSDEQRFLKEHVAEAMAT

RCRRCTERQKDGLEKVVVWYTENRPEEWSALVVHL

>36020870 |Chemosensory protein

TTKYDNVNLEEILSNDRLRNKYVECLTSTSDEHCTPEGKELKSVVSDALT

TDCAKCNEKQKNGTKYVVDTLLDKYPDDYAKLEKVYDADGAYRKKYE

>3639083 |Pheromone binding protein

FIRVDASQDVIKNLSMNFAKPLEDCKKEMDLPDSVTTDFYNFWKEGYEFT

NRQTGCAILCLSSKLELLDQELKLHHGKAQEFAKKHGADDAMAKQLVDLI

HGCAQSTPEVVDDPCMKTLNVAKCFKAKIHE

>37537977 |Pheromone binding protein

YQAVEPSQDVVKDMSLNFRKGLDACKKELNLPDTINADFNRFWNDDHVVT

NRDTGCAIMCLSSKLELVTDTGLHHGNTLEYAKQHGADETVAQQIVDLLH

TCAQAVPDLQDTCMKVLEWAKCFKAEIHK

>37537978 |Pheromone binding protein

YQAVEPSQDVVKDMSLNFRKGLDACKKELNLPDTINSDFNRFWNDDHVVT

NRDTGCAIMCLSSKLELVSDTGLHHGNTLEYAKQHGADDTVAQQIVDLLH

SCAQAVPDLEDPCLKVLEWAKCFKAEIHK

>37537980 |Odorant binding protein

VWQVESSADVMKKLTTGFATALEKCRDELNLPDAVMQDFFNFWREDYELV

NRDMGCAIMCMATKFDLVTEEQKLHHGNAHEFAKSHGADDSMAKQLVTML

HECETQTASISDDCGRTLEIAKCFRTKIHG

>37674500 |Odorant binding protein

EMKELAQQLHNTCVSETGTTEDAITNARAGTFTDDEKFKCYLKCLLDQMA

IVDEEGRIDVEAMIAVLPEEFQDSLPPVIRKCDTIIGANACDNVWLTQQC

YYKENPE

>37778927 |Odorant binding protein

VTPRRDAEYPPPEFLEAMKPLREICIKKTGVTEEAIIEFSDGKVHEDENL

KCYMNCLFHEAKVVDDTGHVHLEKLHDALPDSMHDIALHMGKRCLYPEGE

NLCEKAFWLHKCWKESDPK

>37779208 |Odorant binding protein

VTPRRDAEYPPPELLEALKPLHDICVKKTGVTDEAIIEFSDGKIHEDEKL

KCYMNCLFHEAKVVDDNGDVHLEKLHDSLPNSMHDIAMHMGKRCLYPEGE

NLCEKAFWLHKCWKQADPK

>38454302 |Vertebrate odorant binding proteins

GDWFSIVLASDKREKIEENGSMRVFMQHIDVLENSLGFKFHIKKNGECRE

VYLVAYKTPKDGEYFVEHDGGNTFTILKTDYDRYVMIHLVNVKNGETFQL

MLLYGRTKDLSSDIKEKFEKLCVAHGITRDNIIDLTKTDR

>39579207 |Odorant binding protein

YLLIALKVANGETLRESLRPVIVACSKEHGVTDEEIQAAKEAGSPASIKP

CFIACVFKKAGFLDDQGQIDIETGLKNLRQFVKDDEQYKKLEEVSKLCSF

VKDKVVSDGAAGCEKGALLAGCFLDHKTR

>39840705 |Odorant binding protein

QEPRRDGEWPPPATLKLAKHFHDICAPKTGVTDEAIKEFSDGQIHEDEAL

KWYMNCLFHEFEVVDDNGDVHMEKLFNAIPGEKLRNLLMEASKGCTHPEG

DTLCHKAWWFHQCWKKADPV

>39840707 |Odorant binding protein

AAAQRDENYPPPGILKMAKPFHDACVEKTGVSEAAIKEFSDGEIHEDEKL

KCYMNCFFHEIEVVDDKGDVHLEKLFATVPLSLRDKLVEMSKGCVHPEGD

TLCHKAWWFHQCWKKADPK

>400656 |Odorant binding protein

VGAVSADVQVMKDVTLGFGQALEQCREESQLTEEKMEEFFHFWREDFKFE

HRELGCALQCMSRHFNLLTDSSRMHHENTDKFIKSFPNGAVLSKTMVELI

HNCELQHDAEEDHCWRILRVAECFKISCTK

>401346 |Vertebrate odorant binding proteins

GTWYLKAMTVDREFPEMNLESVTPMTLTTLEGGNLEAKVTMLISGRCQEV

KAVLEKTDEPGKYTADGGKHVAYIIRSHVKDHYIFYCEGELHGKPVRGVK

LVGRDPKNNLEALEDFEKAAGARGLSTESILIPRQSET

>40204882 |Odorant binding protein

DVPQKCHKYVNQLKSANSKYPSYTHLCYPDCIYRETGALVNGKLRVDRVK

QYLEQHVHQRDQDMVAYIMRSFDSCLGNIKSHMKAANIE

>408475 |Insect pheromone binding protein

LQMVTQTQARPQDVITVAGEETEVVIKREGDDDGDDDDSSSEETVEDSEE

SRRRRREVNTDNTPSARAVIPGEQVVPILLEAILPSVDAAGDRFARSVQF

LKNLTPG

>42627893 |Vertebrate odorant binding proteins

GDWFSIVMASDKREKIEENGSMRVFVQHIDVLENSLGFKFHIKVNGKCRE

LYLVAYKTPKDGEYFVEYDGGNTFNILKTDYDRYVMFHLVNFKDGETFQL

MNLLGRTKDLSSDIKEKFAKLCVAHGITRENIIDVTKTDR

>43439928 |Pheromone binding protein

TSAALGSQELMMKMTKGFTKVVDECKAELNAGEHIMQDMYNYWREDYQLI

NRDLGCMILCMAKKLDLMEDQKMHHGKTEEFAKSHGADDEVAKKLVSIIH

ECEQQHAGIADDCMRVLEISKCFRTKIHE

>44976947 |Pheromone binding protein

TSSVMSSKELVSKMSSGFSKVLDQCKAELNVGEHIMQDMYNFWREEYELV

NRDLGCMVMCMASKLDLVGDDQKMHHGKAEEFAKSHGADDELAKQLVGII

HACETQHQAIEDPCSRTLEVAKCFRSKMHE

>45550714 |Odorant binding protein

GSVVFSMFLTRPSLDKGNSECRKSLNLPAHRKFNFAELYTINMCIEECNF

IGCGYIEIDPPFRLDLANIRTNLQTIAPQPQNESIPFLVDAYRKCELFRS

SHGRRFTLHLPDIEFIEEPCNPFALQITICVRIHA

>45551098 |Odorant binding protein

RAADPICSQRPDVTALRNCCKLPNLDFSSFNSKCSQYLVNGVHISPCSFE

CIFRAANALNGTHLVMENIEKMMKTILGSDEFVHVYLDGFRSCGNQEKVL

IKAMKRRRVPITGKCGSMAI

>47568391 |Pheromone binding protein

AYGLVPSDFVKGPDKQDFRKENGKLSKVDVKEAKKFWEAGKKELGKDKIE

LEFLNFDNEESKKIGEYVKGELEKNLPGLTVTIKMQPFAQKLKLE

>47933944 |Chemosensory protein

KLDSFNVDEVLNNERLLKSYIQCMLDADEGRCTNEGKEIKKRLPKFVANG

CLDCTPSQLERAIKTLRHVTEKYPEEWTKLKAKFDPTGEYAKKHA

>48109165 |Juvenile hormone binding protein

ILDAHYSSSGVLIILPASGNGTFHARFDDVTAVVKGTVSTRVKDGKTYLN

VDNLDVVLDVKKVRMGVHKIFNNNRILTEATNLFLRENGQEVLKVMEPQL

KRKLSVLFAGIVNQLLRHVPVEVFLLP

>48139424 |Chemosensory protein

TTKYDNVDIDVVLNTERLLNAYVNCLLDQGPCTPDAAELKRNLPDALENE

CSPCSEKQKKIADKVVQFLIDNKPEIWVLLEAKYDPTGAYKQHYL

>4836777 |Chemosensory protein

KYDNVNLEEVFGNERLLESYRKCLMDEGLCAPDAEELKKAIPDALENECA

KCSEKQKAGVETTIVFLIKNKPEIWESFKKKYDPTHKYEKIYE

>4836779 |Chemosensory protein

KYDNVDVPSLLQNERSANSYYNCLMSLGLCTPEGQFFKELLPDALATGCS

KCSDRQKAIVKAIVEFLKKNKPDDLQKLVNKFDPDGSYRAKYG

>4836781 |Chemosensory protein

KYDNVNLKEIFENERLFASYKECLLGNRPCPPDGQELKDAIPDALENECA

KCSEKQKAGVETTIVFLIKNKPEVWESFKKKYDPTHKYQTFYD

>48557901 |Pheromone binding protein

FIVVKCSQEVMKNLTHHFSKPLEDCKKEMDLPDSVITDFYNFWKEGYEFT

SRHTGCAILCLSSKLELLDPDLKLHHGKAQEFAQKHGADEAMAKQLVGLI

HGCMETIREPADDPCVRAQNVVMCFKAKIHE

>48994214 |Chemosensory protein

TDKFDNIDVDRVLSNDRILNNYLKCLLDKGPCTQEGRELKTLPDALKTNC

EKCSEKQRTSSRKVIAHLEERKPQEWKKLLDKYDPEGIYKSKFE

>48994222 |Chemosensory protein

STRYDNLDIDTILASNRLVTNYVDCLLSRKPCPPEGKDLKRILPEALRTK

CARCSPIQKENALKIITRLYYDYPDQYRALRERWDPSGEYHRRFE

>50812920 |Chemosensory protein

TDKWDNINIDEILESQRLLKAYIDCLLDKGRCTPDAKTLKDTLPDALENE

CNKCTEKQKSGSDKVIRHLVNKRPEMWKELSVKYDPDHIYEGRYK

>5081563 |Pheromone binding protein

ILGAECSQDVMKQMTINFGKALDTCRKELDLPDSINADFYNFWKEGYELS

NRQTGCAIMCLSSKLDLVDPEGKLHHGNTHEFAKKHGADDSMAKQLVELI

HKCEGSVADDPDACMKVLNIAKCFKAEIHK

>5081565 |Pheromone binding protein

ILGAECSQDVMKQMTINFGKALDTCRKELDLPDSINADFYNFWKEGYELS

NRHTGCAIMCLSSKLDLVDPEGKLHHGNTHEFAKKHGADDSMAKQLVELI

HKCEGSVADDPDACMKVLNIAKCFKAEIHK

>5081579 |Pheromone binding protein

XXGAEXSQDVMXQMTINFGKALDTCRKELDLPDSINADFYNFWKEGYELS

NRQTGCAIMCLSSKLDLVDPEGKLHHGNTHEFAKKHGADDSMAKQLVELI

HKCEGSVADDPDACMKVLNIAKCFKAEIHK

>5081585 |Pheromone binding protein

XLGAECSQDVMXQMTINFGKALDTCRKELDLPDSINADFYNFWKEGYELS

NRHTGCAIMCLSSKLDLVDPEGKLHHGNTHEFAKKHGADDSMAKQLVELI

HKCEGSVADDPDACMKVLNIAKCFKAEIHK

>5257315 |Pheromone binding protein

FITVECSQEIMKNLSLQFAKPLEDCKKEMELSETVITDFYNFWKEGYEFT

NRQFGCAILCLSSKLELLDQDLKLHHGKAQEFAKKHGADEAMAKQLVDMI

HGCSQSTPDVADDPCMKTLNVAKCFVAKIHD

>52783245 |Vertebrate odorant binding proteins

ASQFYQGXWYVTHETSAXTLSECNILTTSNDNGKFTVKHKYTKDGXVGEL

ICEGQASANNKFTYDCKFXGZTMEQVTRTAMDTDYNDYALYYLCTTYKXG

P

>52854755 |Odorant binding protein

AAAQREDNYPPPGNLKMAKPLHDACVEKTGVIEAAIKEFSEGEIHEDENL

KCYMNCFFHEIEVVDDKGDVHLEKLFATVPLPMRDNLIEMSKGCVHPEGD

TLCH

>52854757 |Odorant binding protein

HDPRSDGEWPPPAILHLGNHFHDICAPNTGVTDEAIKEFSEGQIHEDEAL

KCYMNCLFHDFEVVDDRGDVHMEKVLNAIPGEKLRNIMMEASKGCIHPEG

DTLCHKAWWF

>53851291 |Pheromone binding protein

TSAALGSQELMMKMTKGFTKVVDDCKAELNAGEHIMQDMYNYWREDYQLI

NRDLGCMILCMAKKLDLMEDQKMHHGKTEEFAKSHGADDEVAKKLVSIIH

ECEQQHAGIADDCMRVLEISKCFRTKIHE

>53851293 |Pheromone binding protein

LVVVQASQDVMKNLAINFAKPLDDCKKEMDLPDSVTTDFYNFWKEGYELT

NRQTGCAILCLSSKLEILDQELNLHHGRAQEFAMKHGADETMAKQIVDMI

HTCAQSTPDVAADPCMKTLNVAKCFKLKIHE

>5442215 |Pheromone binding protein

QEIMKNLSLQFAKPLEDCKKEMELSETVITDFYNFWKEGYEFTNRQFGCA

ILCLSSKLELPDQDLKLHHGKAQEFAKKHGADEAMAKQLVDMIHGCSQST

LDVADDPCMKTLNV

>5442221 |Pheromone binding protein

QEIMKNLSLQFAKPLEDCKKEMELSETVITDFYNFWKEGYEFTNRQFGCA

ILCLSSKLELLDQDLKLHHGRAQEFAKKHGADEAMAKQLVDMIHGCSQST

PDVADDPCMKTLNV

>55978944 |Chemosensory protein

TTKFDNINVQEILHNDRLLNNYVKCLLDQGRCTADAIELKKSLPDALETE

CSKCSPKQKEFAEEAMKFLSHNKKDIWEKLLAKYDPEKKYRSKFE

>56126263 |Odorant binding protein

ATAQRDDNYPQPVKLKMAKPLHDACVENTGVIEAAIKEFSDGEIHEDENL

KCYMNCFFHEIEVVDDHGDVHLEKLFATVPLPMRDNLMEMSKGCVHPEGD

TLCHKVWWFHQCWKKADPK

>56462366 |Chemosensory protein

TNKYDNTNLDEILGNDRLFNAHMECIMGEGKCTPEGRELKEHIGESLENE

CEKCTDDQKKGAKKAIDYIIKHRPEAWKRLTDKFDPSGKYKQQYE

>56713962 |Chemosensory protein

NSKYDNFDVETLISNDRLLKSYVNCFLDKGRCTPEGTDFKKTLPDAVETT

CAKCTDKQKTNIKKVIKAIQTRHPRQWDELVKKNDPTGKHIVNFN

>56805549 |Chemosensory protein

SDMFDHINPDDILPNDELRNQYYNCFMDTGPCVTEDQKYFKEHAAEAFAT

KCRKCTEVQKKNVEKIVVWYTENRPQEWQAMVQKL

>57163775 |Vertebrate odorant binding proteins

GEWYSILLASDVKEKIEENGSMRVFVEHIKALDNSSLSFVFHTKENGKCT

EIFLVADKTKDGVYTVVYDGYNVFSIVETVYDEYILLHLLNFDKTRPFQL

VEFYAREPDVSQKLKEKFVKYCQEHGIVNILDLTEVDR

>57907220 |Odorant binding protein

VALIAGTFALTIDQKKKAEGYAAECVKTTGVPPETAAKLKGGDFAGADDK

TKCFAKCFLEKAGFMTDKGEIDEKTVIEKLSVDHDRAKVEGLVKKCNHKE

ANPCETAFKAYQCIYAAKGA

>58045519 |Odorant binding protein

DINVMKDVTLGFGQALDKCREESQLTEEKMEEFFHFWSDDFKFEYRELGC

AIQCMSRHFNLLTDSSRMHHDNTEKFIQSFPNGEVLARQMVELIHSCEKQ

FDHEDDHCWRILHVAECFKGSCVQ

>58378570 |Odorant binding protein

AECCPTPMLVDGTIMMDCYKKYGEQTKKQLQMDGIPRGCCIAECAMNATN

MYADGMLKRDDLSKMFMDAVKDKPEWMSLVRDATNACFELAEKKMDEIEA

GAKL

>58382521 |Odorant binding protein

CRVQAGSAEELEQAKEMLRGLAAECKTKEGATDEDVEGFVNDKMPESRTQ

KCLAGCMQEQFGVSNGKAFQEDGFIEIAKMLMKGDETKIELAKEIAADCK

AVANDDRCELAVDIMNCLKESAEK

>58389966 |Insect pheromone binding protein

TDKFDNIDVDRVLSNDRILNNYLKCLLDKGPCTQEGRELKKTLPDALKTN

CEKCSEKQRTSSRKVIAHLEERKPQEWKKLLDKYDPEGIYKSKFE

>58389970 |Chemosensory protein

VTKYDNIDLEEIFSSKRLMDNYMNCLKNVGPCTPDGRELKDNLPDALMSD

CVKCSEKQRIGSDKVIKFIVANRPDDFAILEQLYDPTGEYRRKYM

>58391533 |Odorant binding protein

PFPSVECAMTRKQLINSMDMMRSACAPKFKVSTEMLDNLRGGIFAEDREL

KCYTMCIAQMAGTMNKKGEINVPKTLAQMDAMLPPDMRDKAKEAIHSCRD

VQGRYKDSCDKTFYSTKCLAEYDRD

>58395871 |Odorant binding protein

YIVLSAPFEIPDRYKKPAKMLHEICIAESGASEEQLRTCLDGTVPTAPAA

KCYIHCLFDKIDVVDEATGRILLDRLLYIIPDDVKAAVDHLTRECSHIVT

PDKCETAYETVKCYFNAHDE

>58585106 |Chemosensory protein

TSKFDNINVDEILHSDRLLNNYFKCLMDEGRCTAEGNELKRVLPDALATD

CKKCTDKQREVIKKVIKFLVENKPELWDSLANKYDPDKKYRVKFE

>61819907 |Vertebrate odorant binding proteins

GDWHSILMAADNIQKIEEGGPLRAYIRQLECTDRCSSLSVNFYAKFPSQC

TFLNVVAEREGDVYHVGYMGSNFFELIPVSENTLAVYGENFDGVKSTKVT

QLLAKGDHATQEDIQQYEELNRERGIPIEHIEDLTQTDD

>6226255 |Vertebrate odorant binding proteins

RHWHTVVLASSDRSLIEEEGPFRNFIQNITVESGNLNGFFLTRKNGQCIP

LYLTAFKTEEARQFKLNYYGTNDVYYGSSKPNEYAKFIFYNYHDGKVNVV

ANLFGRTPNLSNEIKKRFEEDFMNRGFRRENILDISEVDH

>62286940 |Vertebrate odorant binding proteins

GIWYAKAMVHNGTLPSHKIPSIVFPVRIIALEEGDLETTVVFWNNGHCRE

FKFVMKKTEEPGKYTAFHNTKVIHVEKTSVNEHYIFYCEGRHNGTSSFGM

GKLMGRDSGENPEAMEEFKNFIKRMNLRLENMFVPEIGDK

>62297956 |Vertebrate odorant binding proteins

GVWYSIFMASDDLNRIKENGDLRVFVRNIEHLKNGSLIFDFEYMVQGECV

AVVVVCEKTEKNGEYSINYEGQNTVAVSETDYRLFITFHLQNFRNGTETH

TLALYGTSALEPSFLSRFEETCEKYGLGSQNIIDLTNKDP

>62530469 |Pheromone binding protein

QEIMKNLSLQFAKPLEDCKKEMDLSDTVITDFYNFWKEGYEFTNRQFGCA

ILCLSSKLELLDQDLKLHHGKAQEFAKKHGADEAMAKQLVDMIHSCTQST

PDVADDPCMKTLNVAKCFVAKIHD

>62530471 |Pheromone binding protein

QEVVASFSKGFTNVVEHCKAEVNAGEHIMQDIYNFWREEYQLVNRDLGCM

VLCMANKLGLIGEDQKMHHAKAEEFAKSHGADEAVAKQLVAILYECETKH

AAIEDECGMALEIAKCFRTKMHE

>62530473 |Pheromone binding protein

TSQKVINNFSKGFKDVVDHCKAELNMGEHIMQDVNNFWREEYQLVNRDFG

CMVPVHGQQARAPKRGPEVHHDKAEEYAKKHGADDATAKQVVAIIFECEN

NSSGMDDECNRALEIAKCFRTKMHE

>62653494 |Vertebrate odorant binding proteins

STLSSPLLISSLKINLTAMGKHILLLLLGLCLLVNSLQALTCVTCERFNS

QGICERGEGCCQAKPGQKCASLITYRDGKFLLGSQRCADVCFKGTVENGG

LTAKMKC

>6272608 |Pheromone binding protein

QQKVESSQDVIKGMTLNFRKGLDECKKEMNLPDSINADFYNFWKDDHVLS

NRDTGCAIMCLSSKLELVSDGKLHHGNTFDYAKQHGADETVAQQLVDLVH

SCEKSLPDLEDPCMKVLEWAKCFKTEIHK

>6272610 |Pheromone binding protein

QQKVESSQDVIKGMTLNFRKGLDECKKEMNLPDSINADFYNFWKDDHVLS

NRDTGCAIMCLSSKLELVSDGKLHHGNTFDYAKQHGADETVAQQLVDLXH

SCEKSLPDLEDPCMKVLEWAKCFKTEIHK

>6272612 |Pheromone binding protein

QQKVESSQDVIKGMTLNFRKGLDECKKEMNLPDSINADFYNFWKDDHVLS

NRDTGCAIMCLSSKLELVSDGKLHHGNTFDYAKQHGADETVAQQLVDLIH

SCEKSLPDLEDPCMKVLEWAKCFKTEIHK

>6272614 |Pheromone binding protein

TQMVEXSQDVIKGMCLNFGKGLEECKKEMNLPDTVDADFYNFWKDDYVLT

NRDTGCAIMCLSNKLELVSDGKLHHGNTLDFAKQHGADETVAQQLVDLIH

TCEKALPDLEDPCLKVLEWAKCFKIEIHK

>6272616 |Pheromone binding protein

TQMVEPSQDVIKGMCLNFGKGLEECKKEMNLPDTVDADFYNFWKDDYVLT

NRDTGCAIMCLSNKLELVSDGKLHHGNTLDFAKQHGADETVAQQLVDLIH

TCEKALPDLEDPCLKVLEWAKCFKIEIHK

>6272618 |Pheromone binding protein

TQMVEPSQDVIKGMCLNFGKGLEECKKEMNLPDTVDADFYNFWKDXYVLT

NRDTGCAIMCLSNKLELVSDGKLHHGNTLDFAKQHGADETVAQQLVDLIH

TCEKALPDLEDPCLKVLEWAKCFKIEIHK

>6272620 |Pheromone binding protein

TQMVEPSQDVLKGMTLNFRKGLEECKKEMNLPDTVDAEFYNFWKEDIVLT

NRDTGCAIMCLSSKLELVSDGKLHHGKTFDYAKQHGADDTVAKQMVDLIH

TCEKSLPDLDDPCLKVLEWAKCFKIEIHK

>6272624 |Pheromone binding protein

TQMVEPSQDVLKGMTLNFRKGLEECKKEMXLPDTVDAEFYNFWKEDIVLT

NRDTXCAIMCLSSKLELVSDGKLHHGKTFDYAKQHGADDTVAKQMVDLIH

TCEKSLPDLDDPCLKVLEWAKCFKIEIHK

>6272626 |Pheromone binding protein

TQMVEPSADVVKGMTLNFGKGLEECKKEMNLPDTINADFYNFWKDDHVLT

NRDTGCAIMCLSSKLELVSEGKLHHGNTLEYAKQHGADDTVAQQLVDIIH

TCEKALPDLEDPCLKVLEWAKCFKIEIHK

>6272630 |Pheromone binding protein

TQVVEPSQDVIKGMSLNFGKGLEECKKEMNLPDTVNADFYNFWKDDHVLT

NRDTGCAIMCLSSKLELVSDGKLHHGNTLEYAKQHGADETVAQQLVEIIH

TCEKAMPDLEDPCLKVLEWAKCFKIEIHK

>6272634 |Pheromone binding protein

TQMVEPSADVVKGMTLNFGKGLEECKKEMNLPDSINADFYNFWKDDHVLT

NRDTGCAIMCLSSKLELVSDGKLHHGNTLEYAKQHGADDTVAQQLVDLIH

NCEKALPDLEDPCMKVLEWAKCFKIEIHK

>6272636 |Pheromone binding protein

TQXVEPSQDVIKGMSLNFGKGLEECKKEMNLPDTVNADFYNFWKDDHVLT

NRDTGCAIMCLSSKLELVSDGKLHHGNTLEYAKQHGADETVAQQLVEIIH

TCEKAMPDLEDPCLKVLEWAKCFKIEIHK

>6272646 |Pheromone binding protein

LDWAMASQDIMKKLTVGFSKALDQCKTELAIQENVLQDFYNFWREDYTLV

NREMGCVLMCMASKFDLITEDMKVHHKNAHEFAKTHGADDEMAKQLVSMI

HECEKTHEGVVDDCGRVLEMAKCFKTKIHE

>62911148 |Pheromone binding protein

TSAVLGSQDLMVKMTKGFTRVVDDCKTELNVGDHIMQDMYNYWREDYQLI

NRDMGCMLLCMAKKLDLMDDQTMHHGKTEDFAKSHGADDDVAKKLVSVIH

ECEQQHAGIADDCMRVLEVAKCFRTKIHE

>63020522 |Chemosensory protein

TDKWDNIDLDEILNNKKILASYVKCCLDQGKCTPDAKELKSHIKEALENR

CGKCTPAQKDGTRKVLTHLINHEPEMWNQLCEKYDAEGKYRKMYE

>63746579 |Odorant binding protein

GPWKTVAIAADRVDKIERGGKLRIYCRSLTCEKECKEMKVTFYVLENGQC

SLTTITGYLQEDGKTYKTQYQGDNHYELVKETPENLVFYSENVDRADRKT

KLIFVLGNKPLTSEENERLVKYAVSSHIPPENIRHVLGTDT

>6521353 |Odorant binding protein

EHGQKVLEQIIDYATSCADSLGVSPEDMKLLMEKKFPTSREGQCMPSCVN

KKFGLQKADGTLNKEYRYSEMENVKAIDEEIYNKMNSVWDKCVINGADGT

DECDTGMKVVTCMKEESEK

>6560639 |Antennal binding protein

TLLAAGALALDEEQAELARMVRENCVDEIGVDEGLLAKVDDGADLMPDPK

LKCYLKCTMEMAGMISDGVVDVEAVLGLLPDDVKLRTTDIVRACDTQKGA

DDCDTAFLTQTCWQQANRA

>6560643 |Antennal binding protein

TLLAVGALALDEEQAELARMVRENCVHEIGVDEGLLAKVDDGADLMPDPK

LKCYLKCTMEMAGMISDGVVDVEAVLGLLPDDVKLRTTDIVRACDTQKGA

DDCDTAFLTQTCWQQANRA

>6560649 |Pheromone binding protein

VKEIAPSSDAMRHIANGFLKVLDQCKHELGLTDQIVVDLYQFWKLQYALL

NRDTGCAIICMSKKLDLLDSTGRMHHGNTQEFAVSHGATDEVASKVVVII

RDCEKQQEGEEDDCVRVLEVAKCFRTAIHE

>6560651 |Pheromone binding protein

VKEIAPSSDAMRHIANGFLKVLDQCKHELGLTDQIVVDLYQFWKLQYALL

NRDTGCAIICMSKKLDLLDSTGRMHHGNTQEFAVSHGATDEVASKVVVII

RDCEKQQEGEQDDCVRVLEVAKCFRTAIHE

>6560665 |Pheromone binding protein

MEMVSASQEVLKQMSVGFSKVLQTCKTELSVGDHIIQDFYNYWREDYDLL

NRDFGCMVICMAVKHDLINDQLTMHHGNAHAFAKTHGADDDTAQQLVTIL

RECEAKHQSVEDVCNRALEMAKCFRTKIHE

>6560667 |Pheromone binding protein

MEMVSASQEVLKQMSVGFSKVLQTCKTELSVGDHIIQDFYNYWREDYDLL

NRDFGCMVICMAVKHDLINDQLTMHHGNAHAFVKTHGADDDTAQQLVTIL

RECEAKHQSVEDVCNRALEMAKCFRTKIHE

>6560673 |Hypothetical proteins

TDKYDNVNVDEILANERLLKGYVDCVLERGKCTPEGKELKEHLRDAIETG

CKKCTKPQEEGATKVIDFLIKNKLEVWRELVAKFDPEGKWRKKYE

>6625564 |Juvenile hormone binding protein

QMDTKAKTVLLKTKADLHIVGDIVIELTEQSKSFTGLYTADTNVIGAVRY

GYNLKNDDNGVQHFEVQPETFTCESIGEPKITLSSDLSSALEKDSGNNSL

EPDMEPLKTLRQAAICKIAEACYISVVHNIRASAKILPA

>6631007 |Chemosensory protein

TDKYDNINLDEILANKRLLVAYVNCVMERGKCSPEGKELKEHLQDAIENG

CKKCAENQEKGAYRVIEHLIKNEIEIWRELTAKYDPTGNWRKKYE

>6631009 |Chemosensory protein

TDKYDNINLDEILANKRLLVAYVNCVMERGKCSPEGKELKEHLQDAIENG

CKKCAENQEKGAYRVIEHLIENEIEIWRELTAKYDPTGNWRKKYE

>6631015 |Chemosensory protein

TDKYDNINLDEILANKRLLVAYVNCVMERGKCSPEGKELKEHLQDAIENG

CKKCTENQEKGAYRAIEHLIKNEIEIWRELTAKYDPTGNWRKKYE

>6631017 |Chemosensory protein

TDRYDNVDLDEILGNRRLMVPYIKCILDQGKCAPDAKELKEHIREALENE

CGKCTETQKNGTRRVIGHLINHEDAYWKELTAKYDPQSKFTAKYE

>6631019 |Chemosensory protein

TDRYDSVDLDEILGNRRLLVPYVKCILDQGKCAPDGKELKEHIKEALENE

CGKCTDAQKKGTRRVIAHLINHEEDFWNELTAKFDPERKFTAKYE

>6634103 |Odorant binding protein

PFNKDLQKKEEGLSYPDEDIVRKYEVCVFTKWGVFDEKEGFNHDRLVNHF

EPALNREEIERIIGTCA

>6634105 |Odorant binding protein

NEELQKKEEGLRFPDEEVVAKYEVCVFTKWGVFDEKEGFSHDRLVSQFEP

VLKREEIECIISPCVDKNEQGSLVDEWVYRFQQCVSKIDIA

>66530058 |Juvenile hormone binding protein

RAAAKYRSSGTLLLVKASGAGDYWGEYEGVKAKVFIRAKPFLVQDRRYLR

LQQLKMDFSVQNIKMGVENVRDSNAIILAALNLFINTNSQELLKEMKPDL

RRKLVQVMTTFVERIFAQVPYDAWILD

>66730407 |Vertebrate odorant binding proteins

GDWFSIVVASNKREKIEENGSMRVFMQHIDVLENSLGFKLCIKENGECRK

LYSVAYKTPKIGEYFLEYDGGNTFTILKTDYERYVMFHLVNVNNGEAFQL

MELYGRTKDLSSDIKEKFAKLCEAHGITRDNIIDLTKTDR

>66840183 |Insect pheromone binding protein

TTKFDNFDVEKVLNNDRILTSYIKCLLDQGNCTNEGRELKKVLPDALKTD

CSKCTNVQKDRSERVIKFLIKNRSAEFDKLTAKYDPSGEYKKKIE

>66840185 |Insect pheromone binding protein

TTKYDNIDIDQILASKRLVNNYVQCLLDKKPCTPEGAELRKILPDALKTQ

CSKCNPGQKNAALKVVDRLQKDYDKEWKLLLDKWDPKREQFQKFQ

>66840187 |Insect pheromone binding protein

TTKYDNIDIDQILASKRLVNNYVQCLLDKKPCTPEGAELRKILPDALKTQ

CSKCNPGQKNAALKVVDRLQKDYDAEWKQLLDKWDPKREHFQKFQ

>66840189 |Insect pheromone binding protein

TTKYDNIDIDQILASKRLVNNYVQCLLDKKPCTPEGAELRKILPDALKTQ

CVKCNATQKNAALKVVDRLQRDYDKEWKQLLDKWDPKREYFQKFQ

>66840191 |Insect pheromone binding protein

TTKYDHIDIDQVLGSKRLVNSYVQCLLNKKPCTPEGAELRKILPDALKTQ

CVKCNATQKNAALKVVDRLQRDYDKEWKQLLDKWDPKREYFQKFQ

>66840193 |Insect pheromone binding protein

TTKYDHIDIDQVLGSKRLVNSYVQCLLDKKPCTPEGAELRKILPDALKTQ

CVKCNATQKNAALKVVDRLQRDCDKEWKQLLDKWDPKREYFQKFQ

>66840195 |Insect pheromone binding protein

TTKYDHIDIDQVLASKRLVNSYVQCLLDKKPCTPEGAELRKILPDALKTQ

CAKCNATQKNAALKVVDRLQKDYDAEWKQLLDKWDPKREHFQKFQ

>66840197 |Insect pheromone binding protein

TTKYDHIDIDQVLASKRLVNSYVQCLLDKKPCTPEGAELRKILPDALKTQ

CVKCNATQKNAALKVIDRLQRDYDKEWKQLLDKWDPKREQFQKFQ

>66840201 |Insect pheromone binding protein

TTKYDHIDIDQVLASKRLVNSYVQCLLDKKPCTPEGAELRKILPDALKTQ

CAKCNATQKNAALKVVDRLQKDYDKEWKQLLDKWDPKREQFQKFQ

>66840203 |Insect pheromone binding protein

TTKYDHIDIDQVLGSKRLVNSYVQCLLDKKPCTPEGAELRKILPDALKTQ

CVKCNATQKNAALKVVDRLQRDYDKEWKQLLDKWDPKREYFQKFQ

>66840205 |Insect pheromone binding protein

TTKYDHIDIDQVLGPKRLVNSYVQCLLDKKPCTPEGAELRKILPDALKTQ

CVKCNATQKNAALKVVDRLQRDYDKEWKQLLDKWDPKREYFQKFQ

>66840947 |Insect pheromone binding protein

TTKFDNFDVDKVLNNDRILTSYIKCLLDQGNCTNEGRELKRVLPDALKTD

CSKCTTVQKDRSEKVIKFLIKNRSTDFDHLTAKYDPSGEYKKKIE

>66840949 |Insect pheromone binding protein

TTKFDNFDVDKVLNNDRILTSYIKCLLEQGNCTNEGRELKRVLPDALKTD

CNKCTEVQKNRSGKVIKFLIKNRSNDFDRLIAKYDPTGEYKKKIE

>66840951 |Insect pheromone binding protein

TTKFDNFDVDKVLNNNRILTSYIKCLLDEGNCTNEGRELRKVLPDALKTD

CSKCTEVQKDRSEKVIKFLIKNRSTDFDRLTAKYDPSGEYKKKIE

>66840982 |Insect pheromone binding protein

TTKYDHIDVDQVLASKRLVNSYVQCLLDKKPCTPEGAELRKILPDALKTQ

CAKCNATQKNAALKVVDRLQKDYDAEWKQLLDKWDPKREHFQKFQ

>6688648 |Chemosensory protein

YDNIDLDDILHNDRLLKKYHECLVSSSDASCTPDGKELKAVIPDALTNEC

AKCNEKQKAGAEKVIKFLVKEKPDLWEPLEKKYDPSGSFRQKYG

>6688654 |Chemosensory protein

TTKYDNIDLDDVLHNDRLLKKYHECLLSDSDASCTPDGKELKAAIPDALT

NECAQCNEKQKAGAEKVIRFLIKEKPDLWEPLEKKYDPTGSFRQKYD

>68552951 |Vertebrate odorant binding proteins

GFTSVVATYSRDDDGSVKVLNKGYDMEDGEWKETRGKAKFVGDPSQGELK

VSFFGPFYSSYNIVDLDRENYSWAIVCGYKKSLFWILGREPEMDSALYEQ

LVQRAADLGFDTTALVRQEP

>70733107 |Chemosensory protein

LWRERRIPLISFESACGGRTVIGERARIVVLNALGGRPELKFMALLVQGI

PRSCKLDSQLSYVDVPLAALEKAAVQVVEQVAKVPDLLALEELLV

>71063497 |Pheromone binding protein

LVVVQASQDVMKNLAVNFAKPLDDCKKEMDLPDSVTTDFYNFWKEGYELT

NRQTGCAILCLSSKLEILDQELNLHHGRAQEFAMKHGADEAMAKQIVDMI

HTCAQSTPDEAADPCMKALNVAKCFKLKVHE

>71907511 |Vertebrate odorant binding proteins

GKWYEIGAFPMFFQRQCIGDTTAEYSLKADGEIAVDNRCRTESGFDQAIG

RAWVPETAKNAELKVSFFWPFRSDYWVIALDDSYRWAVVGNPNRKYLWLL

SRTPRLPQAEIERALNSAKAQGYDLNQFKYTRHTET

>7239259 |Juvenile hormone binding protein

AAYDDIGLIFHFKNLNVTGLKNQKISDFRMDTTRKSVLLKTQADLNVVAD

VVIELSKQSKSFAGVMNIQASIIGGAKYSYDLQDDSKGVKHFEVGQETIS

CESIGEP

>726332 |Juvenile hormone binding protein

KMDTDKKSVVLKTKAILNIVADLKIEFTKQNKVFNGPYIAKATALGSSQY

GYSFTKKDDKEYFVVGSEENACEIIGEPDVEIGEELQKALLNDADAKAMK

PDYEANKVALRKKTLCHIVEAAYVTVIHNIRAVAKLFPK

>731102 |Vertebrate odorant binding proteins

GTWYLKAAAWDKEIPDKKFGSVSVTPMKIKTLEGGNLQVKFTVLISGRCQ

EMSTVLEKTDEPGKYTAYSGKQVVYSIPSAVEDHYIFYYEGKIHRHHFQI

AKLVGRNPEINQEALEDFQNAVRAGGLNPDNIFIPKQSET

>7529760 |Pheromone binding protein

NTAVDSSQDVMKSMTLTFTKGLDACKKEMDLPDTIDVDFNNFWKEDYVVT

NRNAGCAIMCLASKVDLVDSMGILIHGSSHEFAKQHGADDNMAKQLSDTL

HSCEKTIGTLNDECLRALNVANCFKVEIHK

>75860114 |Pheromone binding protein

ILGAECSQDVIKQMTINFGKALDTCRKELDLPDSINADFYNFWKEGYELS

NRHTGCAIMCLSSKLDLVDPEGK

>75860122 |Pheromone binding protein

ILGAECSQDAMKQMTINFGKALDTCRKELDLPDSINADFYNFWKEGYELS

NRHTGCAIMCLSSKLDLVDPEGK

>75860130 |Pheromone binding protein

ILGAECSQDVMKQMTINFGKALDTCGKELDLPDSINADFYNFWKEGYELS

NRQTGCAIMCLSSKLDLVDPEGK

>75860134 |Pheromone binding protein

IFSAESSQDVMKQMTINFGKALDTCRKELDLPDSINADFYNFWKEGYELS

NRQTGCAIMCLSSKLDLVDPEGK

>75860142 |Pheromone binding protein

ILGAECSQDVMKQMTLIFGKALDTCRKELDLPDSINADFYNFWKEGYELS

NRHTGCAIMCLSSKLDLVDPEGK

>75860148 |Pheromone binding protein

ILGAECSQDVMKQMTINFGKALDTCRKELDLPDSINADFYNFWREGYELS

NRQTGCAIMCLSSKLDLVDPEGK

>75860150 |Pheromone binding protein

ILGAECSQDVMKQTTINFGKALDTCRKELDLPDSINADFYNFWKEGYELS

NRHTGCAIMCLSSKLDLVDPEGK

>75860162 |Pheromone binding protein

ILGAECSQDVMKQMTIDFGKALDTCRKELDLPDSINADFYNFWKEGYELS

NRHTGCAIMCLSSKLDLVDPEGK

>76666515 |Odorant binding protein

GRWLTYYTAANNIEKITEGAPFHAFMRYLEFDEENGTILMHFYVKENGEC

IEKYASGTKEENFYAVDYAGHNEFQLIRGDANSLLTHNVNVDEDGKETEL

VQLFGKGNNVEPEYKEEYYNTVREKGIPEENILNFIDNDN

>7960308 |Chemosensory protein

TDRYDNVDLDEILGNRRLLVPYVKCILDEGKCAPDGKELKEHIKEALENE

CGKCTDAQKKGTRRVIAHLINHEEDFWNELTAKFDPERKFTAKYE

>82792657 |Pheromone binding protein

VRKAEPSKDAMQYITSGFVKVLEECKHELNLNEQILADLFHFWKLEYSLL

GRDTGCAIICMSKKLDLLDANGRMHHGNAAEFAKKHGAGDEVASKIVTII

HECEKKHEQDGDECLRVLEVAKCFRTGIHE

>82792665 |Chemosensory protein

TDKYDNINLDEILENKRLLLAYVNCVMERGKCSPEGKELKEHLQDAIETG

CTKCTEAQEKGAYKVIEHLIKNELDIWRELAAKYDPKGDWRKKYE

>82799958 |Odorant binding protein

GIWYTQAMVSDRNHTDGKRPMKVFPMTVIALEGGSLEAQLTFWDNGHCHM

KKILMHKTDEPHKYTAFKGKKTIYIQETSVKGYYILYCEGQRHGRSHRKG

KLIGTNSEKNPEAMEEFKKFAMSKGFREENIIVPEQLDQ

>87248601 |Antennal binding protein

FNCGADNVHLTETQKEKAKQYTSECVKESGVSTEVINAAKTGQYSEDKAF

KKFVLCFFNKSAILNSDGTLNMDVALAKLPPGVNKSEAQSVLEQCKDKTG

QDAADKAFEIFQCYYKGTKT

>90111830 |Pheromone binding protein

CLRVDASQDVMKNLSMNFAKPLEDCKKEMDLPDSVTTDFYNFWKEGYEFT

NRHTGCAILCLSSKLELLDQEMKLHHGKAQEFAKKHGADDAMAKQLVDMI

HGCSQSTPDATDDPCMKALNVAKCFKAKIHE

>91077704 |Juvenile hormone binding protein

MFIGQYEVDGKILFLSLQGSGPCNLTSVGGNFRFVSVLQEYTEGKETFVK

FNKPTMDYTLERAYFYLENLLDSGDQQIGIDINRILNENWEDVLKDIDVP

LKETVTTVVESVVSKILRNIPAKNIFPD

>91077882 |Odorant binding protein

KGEFEYEMHGRLLIMELNVKPNATLTFKLGEYEKRRKKHYKVTGSKLVVA

PQSVSANFDNIINGDKELSDNINKMFNDNWKGLFEEIGPTYEDACAQVFQ

GIFNRVLSKVPVDELFGG

>91081919 |Juvenile hormone binding protein

EIKGKYEVNGNVLLFPVRSKGDFWAIFLDVDAAAKIYGKEFKDKNNTRFM

KIEKLLIDFRLQKSRFRVRDIINHGNIIGEAMNQFLNSNANEIIAEMKPA

ANAAIAKHFKGFLNSAFLKLPLKVWLPD

>91082519 |Juvenile hormone binding protein

RIKSNYHVKGRILFFELDGNGPADGNFTDVQVETKLQGRRYQKGSREYIK

FCKMEVKESVSKAHFRFDGLFKGNMELTRQMNQIVNENVDEFLTELQPAI

HVAIEQTVLALVGRIFDKFSIQELFLD

>91082523 |Juvenile hormone binding protein

DLTGDYTVTGKILLAPIEGKGKFVAGIANSNITAYQKVEYVKKRGKDYVR

PVNTTTTIEVGGPKVHLDGLFDGNEELNRVTNTVINDNVNELFEDLKPVI

QQIITNIVEQWLFRALEDNVPFDKLYPV

>91082525 |Juvenile hormone binding protein

HLDMDYDVVGQLLIIPLRSKGFFSGNFTNTQIYAKGNLKLVDKNGVKYFQ

VDKYNMKIRVGDGEIKLIADNPDLQFGADLIANFYNENPRRVMDAVNPIF

VETATDLYRVILNQVLATIPAKELLPE

>91082779 |Juvenile hormone binding protein

HFEGDYDIDMKILVLEYKGVGPITGNFTNYSFDCILKGNRIQKDGEEYLK

FDKMRLRLNIGHSRITLGNLFRDDPIIGRATNDVINDNTDLFINEIKPVL

ENSLAEKFTDIANKITLKFTYKELFP

>91082781 |Juvenile hormone binding protein

SFDGDYDIDARVLVVPIKGTGKFTADISDVDGQGVLKAEISEKNGHREIK

FTSFDFAIKIADYNIHLDNLFNGDEVLSRAAMDVINDNKAEFIQAALPFI

QRKTAEILLDAANKITEDLDYDQVFPE

>91082785 |Juvenile hormone binding protein

SARANYESSGVLMLVQASGGGEYWGEYEGIKCKVYIRASPHRIGQRAFLT

LQQIKMDFSVKNIRMGVENVHNGNSVIQAALNLFINSNAQELLKEMKPDL

KKKLIVLMRNFVENLFANIPYDAWIS

>91082787 |Juvenile hormone binding protein

VGLYEIEGTFFGNKITNQGSWNLNLLDYVQTMTVTRKPRRDANGQIIPNP

PLKVQVNVQSCNKLELHIGHLAGENMLDWVINNAWQPGFVVLRPLINDLV

GTAFTEILNKNFQNFPFEKVFPN

>91082877 |Hypothetical proteins

QVDPTYETDMILLTVLPIYGNGTAKVYLNDLKLDTYLELKTSPSLHIQNF

RILITLDNIKLDIEGLLYDEEFSKFASAVASGLANTFITPYINDNAEAIS

DVLSPIIETAINEILAGNSSTSAPV

>91083195 |Juvenile hormone binding protein

SIKSQYNLKGKILVLPLVGHGACDLKLSNVQTKVTTTVDFPLREGREVVK

IDKMQVDFKVGGMKVKLYNLFNGNKVLGQTVNQFINQNALEIIGELKDSI

GDSLAGIFTDIMNNVFTKMPTDLWFLS

>91083197 |Odorant binding protein

ELTSRYAMEGRILMMPISGTGTSRGNYSNIDATVIMQGQRVHKDNETYFN

IKDFYVDFNIGHATIQLDDLFNGDKELGEAMNLFLNDNWKNVANEIKPVL

EDTIASIFKKFSNKIYHKYPLNLLLPK

>91083199 |Juvenile hormone binding protein

RIKSQYNIDGRILILTLKGQGPADGNFTNVGAKLTVDGQRFFKKGKEYIG

ITNKKLDLSLGKPVFYFDNFFRDNPELNEQTNKIINENIVDILDELRPVV

DQTVSEFVFGINMPKEKQLNR

>91084603 |Juvenile hormone binding protein

RADFDYYFNGSISILPIYGDGNGSIVLDNVKCLHTFYMKQYQKKEKTHFR

VTNSTLDVNPQHVTLQFNNLYNGDKFLESNLNVVMNENWREVYQELKPLL

EEGFTRVFAALFDSVLRRVPVSDLFEG

>91084605 |Juvenile hormone binding protein

RLEADYDMKGRVLLLPVFGNGPCNVTLVNTKINHTLIGEPFERKGRTFLK

WVDYKVTLRPEIVKFHFANLFNGDDRLGNEINRVINENWDAVFTDVRDGY

EKSFGLIFKDLANRVFTRVPLKDIFLE

>91084609 |Juvenile hormone binding protein

RMEADYKINGKLLIFTLNGEGKCNNTFWDVEGANHFKCERYTKKGKTHLR

MTEHVFKFNPKKVIFSYDNIINGNEQLSQQIMKTINENSLTVYADFGAAI

EQVMATVWSQNINEVFARVPEEELFLP

>91084627 |Juvenile hormone binding protein

QMDFDYEISGKILLLPIYGKGPGSITFLGNPRFILTFEMAEFEKRKKKFM

TVTNATLSMEPQLIQFHLENLFNGDKALGDNVVQVMNDNWREVFSDVRPS

YEEAFSQIFAAIFNNLLRKVPIVDLFDG

>91084629 |Juvenile hormone binding protein

KLVFEYEINGRILVLPISGKGPGAITMVKPKFVIDFPLEEYEKNGTKYYK

VGKETLSMQPQKMHFELENLFNGDKLLASNVLQIMNDNWKEVYGDVQASY

EEAFGKIFTSIFNNFLSKVPKSDLFDG

>91084631 |Odorant binding protein

GANTIENMKYFFEFWLQEYTRDGQKHYKVVQNRLVLEPDLIKFKFDNLFD

GDKDLSDKVNQVFNDNSKEVFGDVKPSYESAFGHIFANIFNRLLARVPIV

DLFEA

>91084641 |Juvenile hormone binding protein

RMVSDYTMTGQLLIFPINGHGKCTNYLYDYDGDYEAKCERYNKKNKTYLR

VTDFKFGFKPKRIVFDFENIIDGNEQLSKEVVKTLNENSETVYADVGPAF

NQVMGMVWKQTLNQVFLRVPEDELFLP

>91084645 |Juvenile hormone binding protein

KLVADYDINGKILLLPVYGSGPAQVTMEKVHGFEKFTLEEYEKKGKKHLK

VVSGVLSLEPSLMKFNFENLFNGDKSLGDNINQVVNENWKEVFGDIRASY

EDAFGQIIMGLFNNMLAKIPIEELLEQ

>91084721 |Juvenile hormone binding protein

KILAQYQLEGQLLLIPVHGKGDATLLLKNVTGVAFLPLEKYTKKGLTHLR

IVDGKVTLPKTDLLYMNFQNRENRSQEFSDMINKVLNDIWQAIFNDVKSG

YESFFTEITVSLAGKFFDKVPLSEMLDN

>91085487 |Juvenile hormone binding protein

TKSCHYKMDGKILLLPVKGEGPSTAVLKNVKTVCRLHYDEVKNNGKTYMK

FTKSELDIEPDFVSFNFENLFNGDKALGDNVNKVLNENWRDVFHDVKDDH

IQVVNKILLSLMNNFFAKVSIEEAFD

>91085489 |Juvenile hormone binding protein

KKLCDYELNGKVLLLPIVGTGKSTVILRDIKLGAHYNFEEVQKKGKHFMN

FKSFTVSFNPDFVSFDFENLFNGDKQLGDNINKVLNENYKEVFADVQKGY

EEGFGLVVENILNNLFAKVSIEEAFD

>91085491 |Juvenile hormone binding protein

TKKCDYKLSGRVLLLPIQGEGESTIVLKNVTIAGYFNYETVKRSGKTYLR

FVDHHLSLDVSHVYYHFDNLFNGDKALGDNINKVLNDNWSEVFEDVKGGY

SEVMNQIIQTLLNNFWSVVSLEDAFGE

>91092298 |Juvenile hormone binding protein

VANFEYEIDGKILVFPIKGKGTGVIAMDKPEFALTFIMEEYEKKGEKYYK

VSNTTMSIEPQKIQFGFSNNVFGGDKDFSDKVLEAMNENWKELYADFGFS

NNVFGGDKDSSDKVLEAMNENWKELYADVRPSYEEAYGQIFMSIFNSFLS

KVPISELFDS

>91092540 |Juvenile hormone binding protein

NLFTDYKGDLLVANLVPFYGAGKANVHILNAEIQGSAQTDLSNGISLKNL

RIQLYVESATFDIHGALNNEDFSQILSALLNDLVPSFIDNHQQVISDILS

PIIEGLINAILNGGGSST

>91093109 |Juvenile hormone binding protein

KVFCTYELNGKILLLPISGSGPSTVIIKKLKVKADYNFEQVKKKGKTYMH

FTTFTVALEPGSVFFNFENLFNGDKKLGDNINKVLNENSKQVFDDVKEGY

AQAFGSISALFNFQSLLTVVLLIIC

>94158668 |Odorant binding protein

AICICVGALSIKDFQNAIRMGQSICMAKTGINKQIINDVNDGKINIEDEN

VQLYIECAMKKFSFVDKDGNFNEHVSREIAKIFLNENEINQLITECSAIS

DTNVHLKITKIFQCITKFKTI

>94158709 |Odorant binding protein

AICVCVGAMTHEELKTGIQTLQPICVGETGTSQKIIDEVYNGNVNVEDEN

VQSYVECMMKKFNVVDENGNFNEKNTRDIVQAVLDDNETDQLIVECSPIS

DANVHIKISKIFQCFMKYKTI

>94158711 |Odorant binding protein

AICVCVSAMTLDELKSGLHTVQSVCMKEIGTAQQIIDDINEGKINMDDEN

VLLFIECTMKKFNVVDENANFNEKISSDIVRAVLNDNEADQLLAECSPIS

DPNALIKISKILECFFKYKTI

>94158718 |Odorant binding protein

AICVCVGALTLEELQIGLRAVIPVCRIDSGIDEKKEDDFRNGIIDVENEK

VQLFSECLIKKFNAYDDGGNFNEVVVREIAEIYLDENEVNKLITECSAIS

DADIHLKSSKLIKCFAKYKTL

>94158731 |Odorant binding protein

AFCICVNAMTIEELKIQLRDVQEICKAESGIDQQTVDDINEVNFDVEDEK

PQRYNECILKQFNIVDESGNFKENIVQELTSIYLDENVIKKLVAECSVIS

DANIYIRFNKLVKCFGKYKTM

>94158813 |Odorant binding protein

AFCICVNAMTIEELKIQLHDVQEICKTESGIDQQTVDDINEVNFDVEDEK

PQRYNECILKQFNIVDESGNFKENIVQELTSIYLDENVIKKLVAECSVIS

DANIYIRFNKLVKCFGKYKTM

>94158820 |Odorant binding protein

ILIMCGVQNLRARSVNIFQDIADCVDRSNMTFHELKKLRDSSEARIKLIN

EEENFRNYGCFLACIWQQTGVMNGSELSTYNIAGIIEGQYHDDEDLKTFF

HKIALTCEDDVHRKFLHVNDECDVALSFKLCMLKAMRN

>94158830 |Odorant binding protein

AICVCVGALTLEEFQIGLRAVVPICRIETSIDQQKEDDFRDGNIDVEDEK

VQLFSECLIKKFNGYDDGGNFNEVVIREIAEIFLDENGVNKLITECSAIS

DADLAVKSAKLLKCIGKYKTL

>94385729 |Vertebrate odorant binding proteins

LTCITCDRMNSRRICEGKEGCCQARPGEKCASFITLKDGKIQFGSQRCAD

LCFTGTVMIGDKTVKMNCCNNKSFCNK

>95930208 |Vertebrate odorant binding proteins

GKWYEIARLGHSFERGLERVTAEYRLRDGGGVAVVNRGYSSKNNQWKEAK

GKAYFVEGETTGYLKVSFFGPFYGSYIVFDIDRAGYQYSFVCGPDYSYLW

LLSRTPEVSQQLIEEFKIKAEGLGFDTSELIFVEHTGG

>47567425 |pheromone binding protein

KERAEVGIQFLRLNQKNETLKNQHARLAINGAMNKKAYVETILNNGAVPA

EGMVPAKFAKSPDGNDFRKENGNLVKDDVKTAKENWKKAKQELGTDKVTI

ELLTSDNALAKKTGEYLKG

>47523218 |Salivary lipocalin

GEWYSILLASDAKENIEENGSMRVFVEHIRVLDNSSLAFKFQRKVNGECT

DFYAVCDKVGDGVYTVAYYGENKFRLLEVNYSDYVILHLVNVNGDKTFQL

MEFYGRKPDVEPKLKDKFVEICQQYGIIKENIIDLTKIDR

>29375639 |pheromone binding protein

KEQGDKYATDAEHLIYNGPFKLKEWDNASSDDWTYEKNDTYWDAEKVKLT

EAKVSVIKSPTTAVNLFDSNELDVVNKLSGEFIPGYVDNPAFLSIPQFVT

Y

>29374719 |pheromone binding protein

VYNGPFVLANFEGAGSDTNWTLEKNENYWDKDNVKLDKINFDVVKEAPTA

LNLFQDGQADDVILSGELAQQMAKDPELVIEKEARTSYLEFNQRDKNSPY

NNVNLR

>2497701 |ALL2_BOVIN Allergen Bos d 2 precursor

GEWRIIYAAADNKDKIVEGGPLRNYYRRIECINDCESLSITFYLKDQGTC

LLLTEVAKRQEGYVYVLEFYGTNTLEVIHVSENMLVTYVENYDGERITKM

TEGLAKGTSFTPEELEKYQQLNSERGVPNENIENLIKTDN

>1709596 |Pheromone-binding protein-related protein

AAAQRDENYPPPGILKMAKPFHDACVEKTGVTEAAIKEFSDGEIHEDEKL

KCYMNCFFHEIEVVDDNGDVHLEKLFATVPLSMRDKLMEMSKGCVHPEGD

TLCHKAWWFHQCWKKADPK

>119923704-1|odorant binding protein

GDWRIHYAASSNTEKTSETGPLNVYLHNIQFNEEGDSVVFHHFLKADGVC

IESSFTGRKIGDNVYTLDSKYECFTDAGANRIHFILVSDDGLIISIENVD

EAGNRTRHIGLVGKEADADDHDLERFKEEVRKLGIPEENIVDFTKVVK

>119923704-2|odorant binding protein

GQWSATYIASNGLATAGQKAFVIPHRLYIVFCGKCFILMFYVYDCNWHVW

RPVFICGVWKGPYTYTYEYNGTNQIEFIHVSEKALVAINVNLDETSKKTQ

QILLFGKENEVDEEYLEIFKKEALSRKISEENIKDLTEPDL
