# Supplementary material for: A machine learning approach for the identification of odorant binding proteins from sequence-derived properties
Source: BMC Bioinformatics. 2007 Sep 19;8:351. doi: 10.1186/1471-2105-8-351 (PMC2216042; doi:10.1186/1471-2105-8-351)
Supplement: Additional file 4 — Prediction results of 414 odorant binding proteins. This table provides prediction results for 414 odorant binding proteins by our method, BLAST and HMM, where "+" represents proteins correctly predicted as odorant binding proteins, and "-" represents proteins incorrectly predicted as non odorant binding proteins. [file 1471-2105-8-351-S4.doc]

| GI code | Our method | BLAST | HMM | NCBI annotation |
| --- | --- | --- | --- | --- |
| 91077882 | + | + | + | Odorant binding protein |
| 91083197 | + | + | + | Odorant binding protein |
| 91084631 | + | - | + | Odorant binding protein |
| 125976718 | + | + | + | Odorant binding protein |
| 24668608 | + | + | + | Odorant binding protein |
| 32135154 | + | + | + | Odorant binding protein |
| 32135155 | + | + | + | Odorant binding protein |
| 33356152 | + | + | + | Odorant binding protein |
| 39840705 | + | + | + | Odorant binding protein |
| 39840707 | + | + | + | Odorant binding protein |
| 108870553 | + | + | + | Odorant binding protein |
| 108876021 | + | + | + | Odorant binding protein |
| 112820887 | + | + | + | Odorant binding protein |
| 112820889 | + | + | + | Odorant binding protein |
| 112984426 | + | + | + | Odorant binding protein |
| 117169194 | + | + | + | Odorant binding protein |
| 118778235 | + | + | - | Odorant binding protein |
| 118781254 | + | + | + | Odorant binding protein |
| 118791068 | + | + | + | Odorant binding protein |
| 118794779 | + | + | + | Odorant binding protein |
| 119608458 | - | - | - | Odorant binding protein |
| 119888034 | + | + | + | Odorant binding protein |
| 119888036 | + | + | + | Odorant binding protein |
| 119888038 | + | + | + | Odorant binding protein |
| 119888040 | + | + | + | Odorant binding protein |
| 119888042 | + | + | + | Odorant binding protein |
| 119923704 | + | + | + | Odorant binding protein |
| 119925160 | + | + | + | Odorant binding protein |
| 121485827 | + | + | + | Odorant binding protein |
| 123121834 | + | + | + | Odorant binding protein |
| 123230127 | + | + | + | Odorant binding protein |
| 126508766 | + | + | + | Odorant binding protein |
| 129022 | + | + | + | Odorant binding protein |
| 119923704 | + | + | + | Odorant binding protein |
| 15983753 | + | + | + | Odorant binding protein |
| 17737785 | + | + | + | Odorant binding protein |
| 17943132 | + | + | + | Odorant binding protein |
| 17981809 | + | + | + | Odorant binding protein |
| 1827612 | + | + | + | Odorant binding protein |
| 19071280 | + | + | + | Odorant binding protein |
| 19224143 | + | + | + | Odorant binding protein |
| 19922608 | + | + | + | Odorant binding protein |
| 21730166 | + | + | + | Odorant binding protein |
| 23320747 | + | + | + | Odorant binding protein |
| 2392495 | + | + | + | Odorant binding protein |
| 24644477 | + | + | + | Odorant binding protein |
| 24648633 | + | + | - | Odorant binding protein |
| 24652406 | + | - | - | Odorant binding protein |
| 24653178 | + | + | - | Odorant binding protein |
| 24653631 | + | + | - | Odorant binding protein |
| 24653633 | + | - | - | Odorant binding protein |
| 24656242 | + | + | + | Odorant binding protein |
| 24658422 | + | + | - | Odorant binding protein |
| 24658435 | + | + | + | Odorant binding protein |
| 2494870 | + | + | + | Odorant binding protein |
| 2494872 | + | + | + | Odorant binding protein |
| 27414057 | + | + | + | Odorant binding protein |
| 27414089 | + | + | + | Odorant binding protein |
| 27414091 | + | + | + | Odorant binding protein |
| 27465627 | + | + | + | Odorant binding protein |
| 31239625 | + | + | + | Odorant binding protein |
| 31241197 | + | + | + | Odorant binding protein |
| 31322218 | + | + | + | Odorant binding protein |
| 33355889 | + | + | - | Odorant binding protein |
| 37537980 | + | + | + | Odorant binding protein |
| 37674500 | + | + | + | Odorant binding protein |
| 37778927 | + | + | + | Odorant binding protein |
| 37779208 | + | + | + | Odorant binding protein |
| 39579207 | + | + | + | Odorant binding protein |
| 400656 | + | + | + | Odorant binding protein |
| 40204882 | + | + | - | Odorant binding protein |
| 45550714 | - | - | - | Odorant binding protein |
| 45551098 | + | + | - | Odorant binding protein |
| 52854755 | + | + | + | Odorant binding protein |
| 52854757 | + | + | + | Odorant binding protein |
| 56126263 | + | + | + | Odorant binding protein |
| 57907220 | + | + | + | Odorant binding protein |
| 58045519 | + | + | + | Odorant binding protein |
| 58378570 | + | + | - | Odorant binding protein |
| 58382521 | + | + | + | Odorant binding protein |
| 58391533 | + | + | + | Odorant binding protein |
| 58395871 | + | + | + | Odorant binding protein |
| 63746579 | + | + | + | Odorant binding protein |
| 6521353 | + | + | + | Odorant binding protein |
| 6634103 | + | + | - | Odorant binding protein |
| 6634105 | + | + | + | Odorant binding protein |
| 76666515 | + | + | + | Odorant binding protein |
| 82799958 | + | + | + | Odorant binding protein |
| 94158668 | + | + | + | Odorant binding protein |
| 94158709 | + | + | + | Odorant binding protein |
| 94158711 | + | + | + | Odorant binding protein |
| 94158718 | + | + | + | Odorant binding protein |
| 94158731 | + | + | + | Odorant binding protein |
| 94158813 | + | + | + | Odorant binding protein |
| 94158820 | + | + | + | Odorant binding protein |
| 94158830 | + | + | + | Odorant binding protein |
| 119608456 | - | + | + | Odorant binding protein |
| 117574114 | + | + | + | Pheromone binding protein |
| 119888044 | + | + | + | Pheromone binding protein |
| 129673 | + | + | + | Pheromone binding protein |
| 133919124 | + | + | + | Pheromone binding protein |
| 134270321 | + | + | + | Pheromone binding protein |
| 134948553 | + | + | + | Pheromone binding protein |
| 15826041 | + | + | + | Pheromone binding protein |
| 17647793 | + | + | + | Pheromone binding protein |
| 2444185 | + | + | + | Pheromone binding protein |
| 24643509 | + | + | + | Pheromone binding protein |
| 2494867 | + | + | + | Pheromone binding protein |
| 25990272 | + | + | + | Pheromone binding protein |
| 26007502 | + | + | + | Pheromone binding protein |
| 27464446 | + | + | + | Pheromone binding protein |
| 29374719 | + | - | - | Pheromone binding protein |
| 29375639 | + | - | - | Pheromone binding protein |
| 29377203 | + | - | - | Pheromone binding protein |
| 29377538 | + | - | - | Pheromone binding protein |
| 29377877 | - | - | - | Pheromone binding protein |
| 31747535 | + | + | + | Pheromone binding protein |
| 31747543 | + | + | + | Pheromone binding protein |
| 31747545 | + | + | + | Pheromone binding protein |
| 31747547 | + | + | + | Pheromone binding protein |
| 3639083 | + | + | + | Pheromone binding protein |
| 37537977 | + | + | + | Pheromone binding protein |
| 37537978 | + | + | + | Pheromone binding protein |
| 43439928 | + | + | + | Pheromone binding protein |
| 44976947 | + | + | + | Pheromone binding protein |
| 47567425 | - | - | - | Pheromone binding protein |
| 47568391 | - | - | - | Pheromone binding protein |
| 48557901 | + | + | + | Pheromone binding protein |
| 5081563 | + | + | + | Pheromone binding protein |
| 5081565 | + | + | + | Pheromone binding protein |
| 5081579 | + | + | + | Pheromone binding protein |
| 5081585 | + | + | + | Pheromone binding protein |
| 5257315 | + | + | + | Pheromone binding protein |
| 53851291 | + | + | + | Pheromone binding protein |
| 53851293 | + | + | + | Pheromone binding protein |
| 5442215 | + | + | + | Pheromone binding protein |
| 5442221 | + | + | + | Pheromone binding protein |
| 62530469 | + | + | + | Pheromone binding protein |
| 62530471 | + | + | + | Pheromone binding protein |
| 62530473 | + | + | + | Pheromone binding protein |
| 6272608 | + | + | + | Pheromone binding protein |
| 6272610 | + | + | + | Pheromone binding protein |
| 6272612 | + | + | + | Pheromone binding protein |
| 6272614 | + | + | + | Pheromone binding protein |
| 6272616 | + | + | + | Pheromone binding protein |
| 6272618 | + | + | + | Pheromone binding protein |
| 6272620 | + | + | + | Pheromone binding protein |
| 6272624 | + | + | + | Pheromone binding protein |
| 6272626 | + | + | + | Pheromone binding protein |
| 6272630 | + | + | + | Pheromone binding protein |
| 6272634 | + | + | + | Pheromone binding protein |
| 6272636 | + | + | + | Pheromone binding protein |
| 6272646 | + | + | + | Pheromone binding protein |
| 62911148 | + | + | + | Pheromone binding protein |
| 6560649 | + | + | + | Pheromone binding protein |
| 6560651 | + | + | + | Pheromone binding protein |
| 6560665 | + | + | + | Pheromone binding protein |
| 6560667 | + | + | + | Pheromone binding protein |
| 71063497 | + | + | + | Pheromone binding protein |
| 7529760 | + | + | + | Pheromone binding protein |
| 75860114 | + | + | + | Pheromone binding protein |
| 75860122 | + | + | + | Pheromone binding protein |
| 75860130 | + | + | + | Pheromone binding protein |
| 75860134 | + | + | + | Pheromone binding protein |
| 75860142 | + | + | + | Pheromone binding protein |
| 75860148 | + | + | + | Pheromone binding protein |
| 75860150 | + | + | + | Pheromone binding protein |
| 75860162 | + | + | + | Pheromone binding protein |
| 82792657 | + | + | + | Pheromone binding protein |
| 90111830 | + | + | + | Pheromone binding protein |
| 112031518 | + | + | + | Chemosensory protein |
| 112031531 | + | + | + | Chemosensory protein |
| 112032057 | + | + | + | Chemosensory protein |
| 112032227 | + | + | + | Chemosensory protein |
| 112032244 | + | + | + | Chemosensory protein |
| 112032265 | + | + | + | Chemosensory protein |
| 112032318 | + | + | + | Chemosensory protein |
| 112983042 | + | + | + | Chemosensory protein |
| 112983048 | + | + | + | Chemosensory protein |
| 112983050 | + | + | + | Chemosensory protein |
| 112983052 | + | + | + | Chemosensory protein |
| 112983054 | + | + | + | Chemosensory protein |
| 112983058 | + | + | + | Chemosensory protein |
| 112983094 | + | + | + | Chemosensory protein |
| 112984474 | + | + | + | Chemosensory protein |
| 113951689 | + | + | + | Chemosensory protein |
| 113951691 | + | + | + | Chemosensory protein |
| 113951693 | + | + | + | Chemosensory protein |
| 113951697 | + | + | + | Chemosensory protein |
| 113951699 | + | + | + | Chemosensory protein |
| 113951701 | + | + | + | Chemosensory protein |
| 113951703 | + | + | + | Chemosensory protein |
| 113951705 | + | + | + | Chemosensory protein |
| 113951707 | + | + | + | Chemosensory protein |
| 113951709 | + | + | + | Chemosensory protein |
| 113951711 | + | + | + | Chemosensory protein |
| 113951715 | + | + | + | Chemosensory protein |
| 113951719 | + | + | + | Chemosensory protein |
| 113951721 | + | + | + | Chemosensory protein |
| 118404322 | + | + | + | Chemosensory protein |
| 122894080 | + | + | + | Chemosensory protein |
| 122894082 | + | + | + | Chemosensory protein |
| 122894084 | + | + | + | Chemosensory protein |
| 122894086 | + | + | + | Chemosensory protein |
| 122894088 | + | + | + | Chemosensory protein |
| 124246513 | + | + | + | Chemosensory protein |
| 124246515 | + | + | + | Chemosensory protein |
| 14091480 | + | + | + | Chemosensory protein |
| 19922956 | + | + | + | Chemosensory protein |
| 21898556 | + | + | + | Chemosensory protein |
| 21898574 | + | + | + | Chemosensory protein |
| 21898673 | + | + | + | Chemosensory protein |
| 26007526 | + | + | + | Chemosensory protein |
| 27065152 | + | + | + | Chemosensory protein |
| 31442896 | + | + | + | Chemosensory protein |
| 31747328 | + | + | + | Chemosensory protein |
| 33413583 | + | + | + | Chemosensory protein |
| 36020870 | + | + | + | Chemosensory protein |
| 48139424 | + | + | + | Chemosensory protein |
| 4836777 | + | + | + | Chemosensory protein |
| 4836779 | + | + | + | Chemosensory protein |
| 4836781 | + | + | + | Chemosensory protein |
| 48994214 | + | + | + | Chemosensory protein |
| 48994222 | + | + | + | Chemosensory protein |
| 50812920 | + | + | + | Chemosensory protein |
| 55978944 | + | + | + | Chemosensory protein |
| 56713962 | + | + | + | Chemosensory protein |
| 56805549 | + | + | + | Chemosensory protein |
| 58389970 | + | + | + | Chemosensory protein |
| 58585106 | + | + | + | Chemosensory protein |
| 63020522 | + | + | + | Chemosensory protein |
| 6631007 | + | + | + | Chemosensory protein |
| 6631009 | + | + | + | Chemosensory protein |
| 6631015 | + | + | + | Chemosensory protein |
| 6631017 | + | + | + | Chemosensory protein |
| 6631019 | + | + | + | Chemosensory protein |
| 6688648 | + | + | + | Chemosensory protein |
| 6688654 | + | + | + | Chemosensory protein |
| 70733107 | - | - | - | Chemosensory protein |
| 7960308 | + | + | + | Chemosensory protein |
| 82792665 | + | + | + | Chemosensory protein |
| 27543486 | + | + | + | Chemosensory protein |
| 27543488 | + | + | + | Chemosensory protein |
| 27543490 | + | + | + | Chemosensory protein |
| 27543492 | + | + | + | Chemosensory protein |
| 27543494 | + | + | + | Chemosensory protein |
| 27543498 | + | + | + | Chemosensory protein |
| 27543500 | + | + | + | Chemosensory protein |
| 27543502 | + | + | + | Chemosensory protein |
| 47933944 | + | + | + | Chemosensory protein |
| 56462366 | + | + | + | Chemosensory protein |
| 18140737 | + | + | + | Antennal binding protein |
| 24665733 | + | + | + | Antennal binding protein |
| 28396154 | + | - | - | Antennal binding protein |
| 28396160 | + | - | - | Antennal binding protein |
| 31747523 | + | + | + | Antennal binding protein |
| 31747525 | + | + | + | Antennal binding protein |
| 31747533 | + | + | - | Antennal binding protein |
| 6560639 | + | + | + | Antennal binding protein |
| 6560643 | + | + | + | Antennal binding protein |
| 87248601 | + | + | + | Antennal binding protein |
| 58389966 | + | + | + | Insect pheromone binding protein |
| 125807118 | + | + | + | Insect pheromone binding protein |
| 125808901 | + | + | + | Insect pheromone binding protein |
| 125809549 | + | + | + | Insect pheromone binding protein |
| 126002371 | + | + | + | Insect pheromone binding protein |
| 408475 | + | - | - | Insect pheromone binding protein |
| 66840183 | + | + | + | Insect pheromone binding protein |
| 66840185 | + | + | + | Insect pheromone binding protein |
| 66840187 | + | + | + | Insect pheromone binding protein |
| 66840189 | + | + | + | Insect pheromone binding protein |
| 66840191 | + | + | + | Insect pheromone binding protein |
| 66840193 | + | + | + | Insect pheromone binding protein |
| 66840195 | + | + | + | Insect pheromone binding protein |
| 66840197 | + | + | + | Insect pheromone binding protein |
| 66840201 | + | + | + | Insect pheromone binding protein |
| 66840203 | + | + | + | Insect pheromone binding protein |
| 66840205 | + | + | + | Insect pheromone binding protein |
| 66840947 | + | + | + | Insect pheromone binding protein |
| 66840949 | + | + | + | Insect pheromone binding protein |
| 66840951 | + | + | + | Insect pheromone binding protein |
| 66840982 | + | + | + | Insect pheromone binding protein |
| 91084721 | + | + | + | Juvenile hormone binding protein |
| 91084641 | + | + | + | Juvenile hormone binding protein |
| 91084629 | + | + | + | Juvenile hormone binding protein |
| 91084609 | + | + | + | Juvenile hormone binding protein |
| 91082525 | + | + | + | Juvenile hormone binding protein |
| 108870204 | + | + | + | Juvenile hormone binding protein |
| 112983082 | + | - | - | Juvenile hormone binding protein |
| 113205954 | + | - | - | Juvenile hormone binding protein |
| 1710156 | - | - | - | Juvenile hormone binding protein |
| 22094831 | + | - | - | Juvenile hormone binding protein |
| 22770450 | - | - | - | Juvenile hormone binding protein |
| 30984068 | + | - | - | Juvenile hormone binding protein |
| 6625564 | + | - | - | Juvenile hormone binding protein |
| 7239259 | + | - | - | Juvenile hormone binding protein |
| 726332 | - | - | - | Juvenile hormone binding protein |
| 13959427 | + | + | + | Juvenile hormone binding protein |
| 17738189 | + | + | + | Juvenile hormone binding protein |
| 108870205 | + | + | + | Hypothetical proteins |
| 108870207 | + | + | + | Hypothetical proteins |
| 108875704 | + | + | + | Hypothetical proteins |
| 108879356 | + | + | + | Hypothetical proteins |
| 108883363 | + | + | + | Hypothetical proteins |
| 108883365 | + | + | + | Hypothetical proteins |
| 108883368 | + | + | + | Hypothetical proteins |
| 108883369 | + | + | + | Hypothetical proteins |
| 108883370 | + | + | + | Hypothetical proteins |
| 108883371 | + | + | + | Hypothetical proteins |
| 108883913 | + | + | + | Hypothetical proteins |
| 108883914 | + | + | + | Hypothetical proteins |
| 108884243 | + | + | + | Hypothetical proteins |
| 112983166 | + | + | + | Hypothetical proteins |
| 112983172 | + | + | + | Hypothetical proteins |
| 112983174 | + | + | + | Hypothetical proteins |
| 112983410 | + | + | + | Hypothetical proteins |
| 24762502 | + | + | + | Hypothetical proteins |
| 28195402 | + | + | + | Hypothetical proteins |
| 31982543 | + | - | - | Hypothetical proteins |
| 6560673 | + | + | + | Hypothetical proteins |
| 91082877 | + | + | + | Hypothetical proteins |
| 1168469 | + | + | + | Aphrodisin |
| 109473603 | + | + | - | Probasin |
| 123226369 | + | + | + | Probasin |
| 130701 | + | + | + | Vertebrate odorant binding proteins |
| 13124669 | + | + | + | Vertebrate odorant binding proteins |
| 126463195 | + | - | - | Vertebrate odorant binding proteins |
| 110596817 | + | - | - | Vertebrate odorant binding proteins |
| 113947522 | + | - | - | Vertebrate odorant binding proteins |
| 113970920 | + | - | - | Vertebrate odorant binding proteins |
| 114048144 | + | - | - | Vertebrate odorant binding proteins |
| 120599491 | + | - | - | Vertebrate odorant binding proteins |
| 121525756 | + | - | - | Vertebrate odorant binding proteins |
| 126173745 | + | - | - | Vertebrate odorant binding proteins |
| 12860858 | + | - | - | Vertebrate odorant binding proteins |
| 13430369 | + | + | + | Vertebrate odorant binding proteins |
| 1718160 | + | + | + | Vertebrate odorant binding proteins |
| 266472 | + | + | + | Vertebrate odorant binding proteins |
| 401346 | + | + | + | Vertebrate odorant binding proteins |
| 47523218 | + | + | + | Vertebrate odorant binding proteins |
| 62286940 | + | + | + | Vertebrate odorant binding proteins |
| 62297956 | + | + | + | Vertebrate odorant binding proteins |
| 68552951 | + | - | - | Vertebrate odorant binding proteins |
| 71907511 | + | - | - | Vertebrate odorant binding proteins |
| 95930208 | + | - | - | Vertebrate odorant binding proteins |
| 109474987 | + | + | + | Vertebrate odorant binding proteins |
| 109483261 | - | - | - | Vertebrate odorant binding proteins |
| 38454302 | + | + | + | Vertebrate odorant binding proteins |
| 42627893 | + | + | + | Vertebrate odorant binding proteins |
| 62653494 | + | - | - | Vertebrate odorant binding proteins |
| 94385729 | + | - | - | Vertebrate odorant binding proteins |
| 11277082 | + | + | + | Vertebrate odorant binding proteins |
| 11277083 | + | + | + | Vertebrate odorant binding proteins |
| 119925395 | + | + | + | Vertebrate odorant binding proteins |
| 126723762 | + | + | + | Vertebrate odorant binding proteins |
| 52783245 | + | - | - | Vertebrate odorant binding proteins |
| 57163775 | + | + | + | Vertebrate odorant binding proteins |
| 61819907 | + | + | + | Vertebrate odorant binding proteins |
| 6226255 | + | + | + | Vertebrate odorant binding proteins |
| 137823 | + | + | + | Vertebrate odorant binding proteins |
| 16225961 | + | + | + | Vertebrate odorant binding proteins |
| 66730407 | + | + | + | Vertebrate odorant binding proteins |
| 731102 | + | + | + | Vertebrate odorant binding proteins |
| 11387218 | - | - | - | Vomeromodulin |
| 110759398 | + | + | + | Juvenile hormone binding protein |
| 110766380 | + | + | + | Juvenile hormone binding protein |
| 110766385 | + | + | + | Juvenile hormone binding protein |
| 110766387 | + | + | + | Juvenile hormone binding protein |
| 116806264 | + | + | + | Juvenile hormone binding protein |
| 116806284 | + | + | + | Juvenile hormone binding protein |
| 118780302 | + | + | + | Juvenile hormone binding protein |
| 118793789 | + | + | + | Juvenile hormone binding protein |
| 118793791 | + | + | + | Juvenile hormone binding protein |
| 119113249 | + | + | + | Juvenile hormone binding protein |
| 119508352 | + | - | + | Juvenile hormone binding protein |
| 125778466 | + | + | + | Juvenile hormone binding protein |
| 125778468 | + | + | + | Juvenile hormone binding protein |
| 125778614 | + | + | + | Juvenile hormone binding protein |
| 125778616 | + | + | + | Juvenile hormone binding protein |
| 125984734 | + | + | + | Juvenile hormone binding protein |
| 125984936 | + | + | + | Juvenile hormone binding protein |
| 125984938 | + | + | + | Juvenile hormone binding protein |
| 125984940 | + | + | + | Juvenile hormone binding protein |
| 125985295 | + | - | + | Juvenile hormone binding protein |
| 20129495 | + | + | + | Juvenile hormone binding protein |
| 21355505 | + | + | + | Juvenile hormone binding protein |
| 24649971 | + | + | + | Juvenile hormone binding protein |
| 28572146 | + | + | + | Juvenile hormone binding protein |
| 33328843 | + | + | + | Juvenile hormone binding protein |
| 33328933 | + | + | + | Juvenile hormone binding protein |
| 33328935 | + | + | + | Juvenile hormone binding protein |
| 48109165 | + | + | + | Juvenile hormone binding protein |
| 66530058 | + | + | + | Juvenile hormone binding protein |
| 91077704 | + | + | + | Juvenile hormone binding protein |
| 91081919 | + | + | + | Juvenile hormone binding protein |
| 91082519 | + | + | + | Juvenile hormone binding protein |
| 91082523 | + | + | + | Juvenile hormone binding protein |
| 91082779 | + | + | + | Juvenile hormone binding protein |
| 91082781 | + | + | + | Juvenile hormone binding protein |
| 91082785 | + | + | + | Juvenile hormone binding protein |
| 91082787 | + | + | + | Juvenile hormone binding protein |
| 91083195 | + | + | + | Juvenile hormone binding protein |
| 91083199 | + | + | + | Juvenile hormone binding protein |
| 91084603 | + | + | + | Juvenile hormone binding protein |
| 91084605 | + | + | + | Juvenile hormone binding protein |
| 91084627 | + | + | + | Juvenile hormone binding protein |
| 91084645 | + | + | + | Juvenile hormone binding protein |
| 91085487 | + | + | + | Juvenile hormone binding protein |
| 91085489 | + | + | + | Juvenile hormone binding protein |
| 91085491 | + | + | + | Juvenile hormone binding protein |
| 91092298 | + | + | + | Juvenile hormone binding protein |
| 91092540 | + | + | + | Juvenile hormone binding protein |
| 91093109 | + | + | + | Juvenile hormone binding protein |
| 21314941 | + | + | + | Insect pheromone/odorant binding protein |
